# Cyclophane-based shielding strategy for singly dispersed graphene nanoribbons

**DOI:** 10.1038/s41557-026-02172-z

**Published:** 2026-06-05

**Authors:** Jin-Jiang Zhang, Jian Zhang, Guanzhao Wen, Silvio Osella, Zhenlin Qiu, Steffen Böckmann, Xu Wang, Britta Maib, Yubin Fu, Xiuling Yu, Michael Ryan Hansen, Janina Maultzsch, Michel Calame, Mickael L. Perrin, Hai I. Wang, Mischa Bonn, Ji Ma, Klaus Müllen, Xinliang Feng

**Affiliations:** 1 Max Planck Institute of Microstructure Physics, Halle, Germany; 2 Center for Advancing Electronics Dresden, Faculty of Chemistry and Food Chemistry, Technische Universität Dresden, Dresden, Germany; 3 Institute of New Concept Sensors and Molecular Materials, Shaanxi Provincial Key Laboratory of New Concept Sensors and Molecular Materials, Xi’an Jiaotong University, Xi’an, China; 4 Nanofabrication Laboratory, National Center for Nanoscience and Technology, Beijing, China; 5 Max-Planck-Institute for Polymer Research, Mainz, Germany; 6 Materials and Processes Simulation Lab, Centre of New Technologies, University of Warsaw, Warsaw, Poland; 7 Institute of Physical Chemistry, University of Münster, Münster, Germany; 8 College of Polymer Science and Engineering, State Key Laboratory of Polymer Material and Engineering, Sichuan University, Chengdu, China; 9 Department of Physics, Friedrich-Alexander-Universität Erlangen-Nürnberg, Erlangen, Germany; 10 Transport at Nanoscale Interfaces Laboratory, Empa Swiss Federal Laboratories for Materials Science and Technology, Dübendorf, Switzerland; 11 Department of Physics, University of Basel, Basel, Switzerland; 12 Swiss Nanoscience Institute, University of Basel, Basel, Switzerland; 13 Department of Information Technology and Electrical Engineering, ETH Zürich, Zurich, Switzerland; 14 Quantum Center, ETH Zürich, Zurich, Switzerland; 15 Nanophotonics, Debye Institute for Nanomaterials Science, Utrecht University, Utrecht, the Netherlands; 16 Beijing National Laboratory for Molecular Sciences, CAS Key Laboratory of Organic Solids, Institute of Chemistry, Chinese Academy of Science, Beijing, China; 17 College of Materials Science and Opto-Electronic Technology, Center of Materials Science and Optoelectronics Engineering, University of Chinese Academy of Science, Beijing, China

**Keywords:** Materials chemistry, Electronic properties and materials, Electronic devices, Electronic properties and devices, Graphene

## Abstract

Structurally precise graphene nanoribbons (GNRs) hold great promise for nanoelectronic devices owing to their tunable bandgaps and unique electronic properties. However, their practical integration into single-ribbon devices remains impeded by strong inter-ribbon aggregation. Here we introduce a cyclophane-based shielding strategy that not only sterically protects the GNR backbone but also imparts internal strain, enabling singly dispersed GNRs while simultaneously modulating their optoelectronic properties, as demonstrated by the synthesis and investigation of three cyclophane-shielded GNRs **1a–c** and model nanographenes with varying cyclic chain lengths. Terahertz spectroscopy reveals a marked increase in short-range charge carrier mobility—from 190 to 330 cm^2 ^V^−1 ^s^−1^ —as the chain length shortens, attributed to both reduced effective mass and increased scattering time. The resulting singly dispersed cyclophane-shielded GNRs enable the fabrication of single-electron transistors showing a clear Coulomb blockade behaviour at low temperatures. This work provides a generalizable strategy for engineering solution-processable GNRs compatible with quantum device applications.

**Figure:**
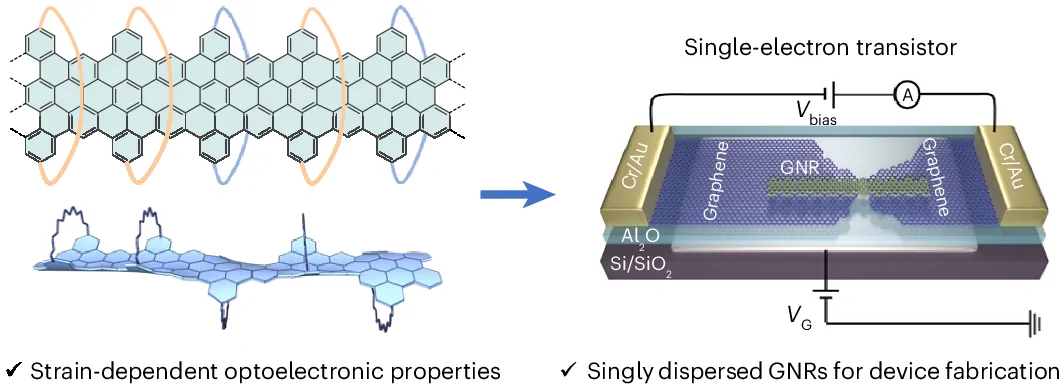

## Abstract (web summary)

The integration of graphene nanoribbons into single-ribbon devices is impeded by strong inter-ribbon aggregation. Now a cyclophane-based shielding approach has been developed to sterically protect the graphene nanoribbon backbone while imparting internal strain, enabling singly dispersed graphene nanoribbons and modulation of their optoelectronic properties.

## Main

Atomically precise graphene nanoribbons (GNRs), synthesized via bottom-up approaches, have attracted tremendous interest due to their exceptional physicochemical properties, such as tunable bandgaps and exotic edge states^1–5^. These features position GNRs as promising candidates for nanoelectronic and spintronic devices^4,6–9^. The wide variety of GNRs synthesized so far provides a robust platform for exploring their intrinsic quantum properties^10–13^, such as Coulomb blockade and discrete charge states in single-electron transistor (SET) operation within a single GNR channel. However, reliably integrating individual GNRs into devices, particularly upon solution-processing, remains a notable challenge due to the severe aggregation driven by the strong *π*–*π* interactions between adjacent ribbons^14,15^. To improve the dispersibility of GNRs, commonly used concepts include topological modifications, such as introducing curvature to the GNR backbone^16^, and side-chain engineering through the incorporation of branched side chains or bulky functional groups^17^. However, these approaches rarely yield GNRs with true single-dispersion capabilities—an essential requirement for fabricating SET devices^8,18^. A more effective strategy would involve spatially shielding the *π*-conjugated surfaces^19–21^ of the GNR backbone to suppress their aggregation and enable the formation of monodisperse GNR solutions, which are compatible with the device integrations. Despite great potential, such GNRs have yet to be realized, primarily due to the synthetic challenges.

**Fig. 1:**
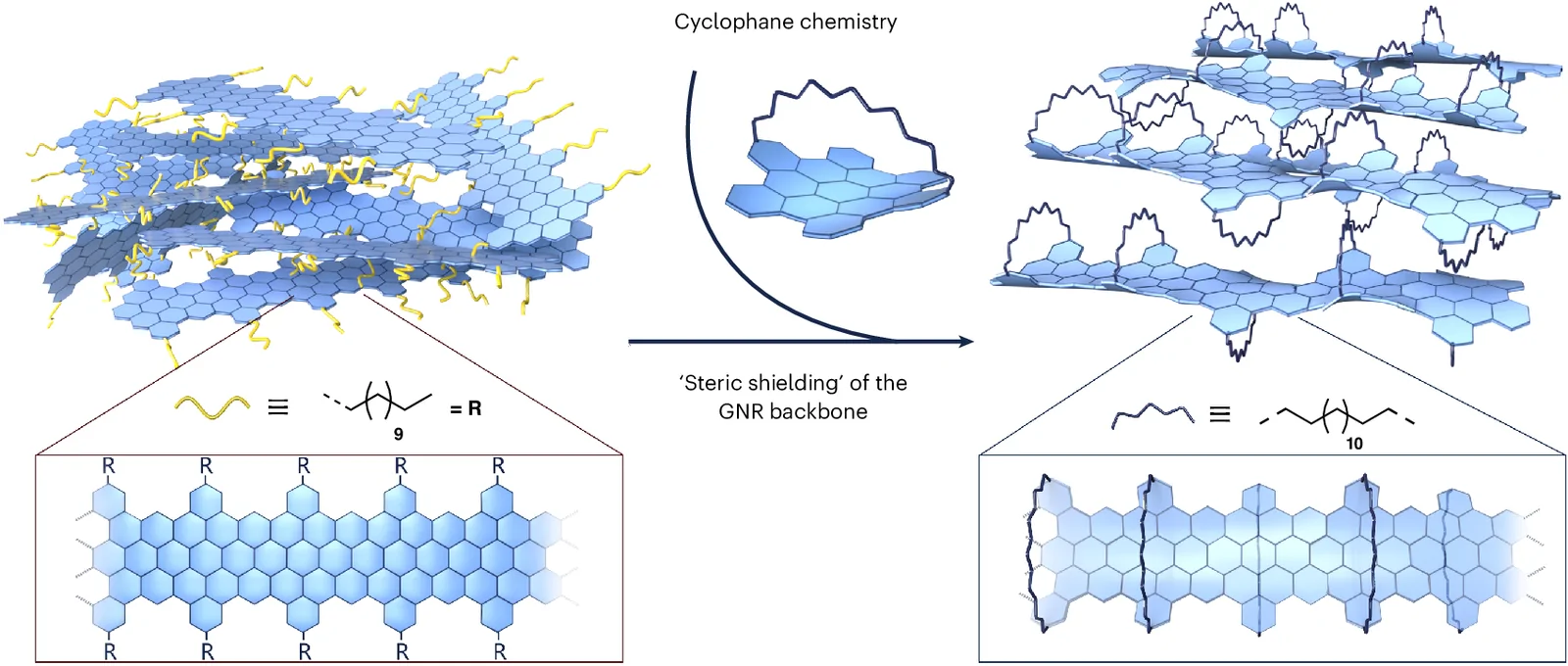
Conventional versus cyclophane-shielded GNRs. Schematic illustration of introducing cyclophane-based shielding strategy into gulf-edged GNR to effectively suppress inter-ribbon aggregation.

Spatial shielding of the *π*-conjugated backbone can offer the resultant system with unique physicochemical properties, such as enabling spin-state manipulation at magnetic centres^22^, preventing enantiomerization in chiral molecules^23,24^, stabilizing highly reactive open-shell system^25–28^ and enhancing light-emitting device performance by suppressing Dexter energy transfer^29^. In this context, strained cyclophanes represent a prototypical example, consisting of a tensioned flexible chain that connects rigid *π*-conjugated units at both ends^30–33^. Moreover, the inherent geometric mismatch in these systems makes them ideal models for studying the effects of bending strain on the optoelectronic properties^23,34–38^. Considering the unique structural features of strained cyclophanes, we envision applying cyclophane chemistry to GNR systems—by bridging the two opposite edges of GNRs with tensioned cyclic alkanediyl chains. Such an approach not only could sterically shield both *π*-conjugated surfaces of the ribbons (Fig. 1), rendering inter-ribbon aggregation energetically unfavourable and thereby yielding singly dispersed GNRs, but also provide a platform to investigate the strain effect on GNR’s optoelectronic properties.

Here we demonstrate that shielding the backbone by cyclophane-type bridges represents an efficient approach to achieve singly dispersed GNRs, enabling their successful integration into SET device fabrication. Three types of cyclophane-shielded GNR (CsGNR) with varying tethered chain lengths (benzene–C_20_–benzene for **1a**, C_20_ for **1b** and C_14_ for **1c**) are synthesized from ethynyl-substituted cyclopentadienone-based cyclophane monomers. The resulting CsGNRs provide an ideal platform to investigate how steric shielding and backbone bending, induced by the tethered chains, affect the dispersibility and optoelectronic properties of GNRs. Three model nanographene compounds (**2a**–**c**), each comprising a hexa-*peri*-hexabenzocoronene (HBC) core tethered with differently substituted alkanediyl chains, are synthesized in excellent yields, suggesting that the bridges present in this study have a negligible influence on the efficiency of the cyclodehydrogenation process. Single-crystal X-ray analysis of **2c** featuring C_14_ cyclic chain as the bridge reveals a significantly strained, bent structure with an end-to-end angle as high as 37.3° and a ~0.4 nm distance between the tethered chain and the HBC core—well beyond typical *π*–*π* stacking distances. As a consequence of the efficient suppression of *π*–*π* stacking, the corresponding CsGNR **1c** exhibits single dispersibility in *N*-methyl-2-pyrrolidone (NMP), achieving a concentration of up to 0.03 g l^−1^. Compared with **1a** and **1b**, **1c** also shows a larger optical bandgap (2.0 eV; +0.2 eV versus **1a**, +0.06 eV versus **1b**) and exhibits pronounced concentration-dependent emission. Optical pump terahertz-probe spectroscopy reveals a notable boost in the short-range charge carrier mobility from 190 for **1b** to 330 cm^2^ V^−1 ^s^−1^ for **1c**, attributed to the reduced effective mass and longer scattering time resulting from the increased structural bending strain. Moreover, SET devices based on **1c** are fabricated, exhibiting clear Coulomb blockade behaviour at low temperatures.

## Results and discussion

### Synthesis and characterization of model compounds

**Fig. 2:**
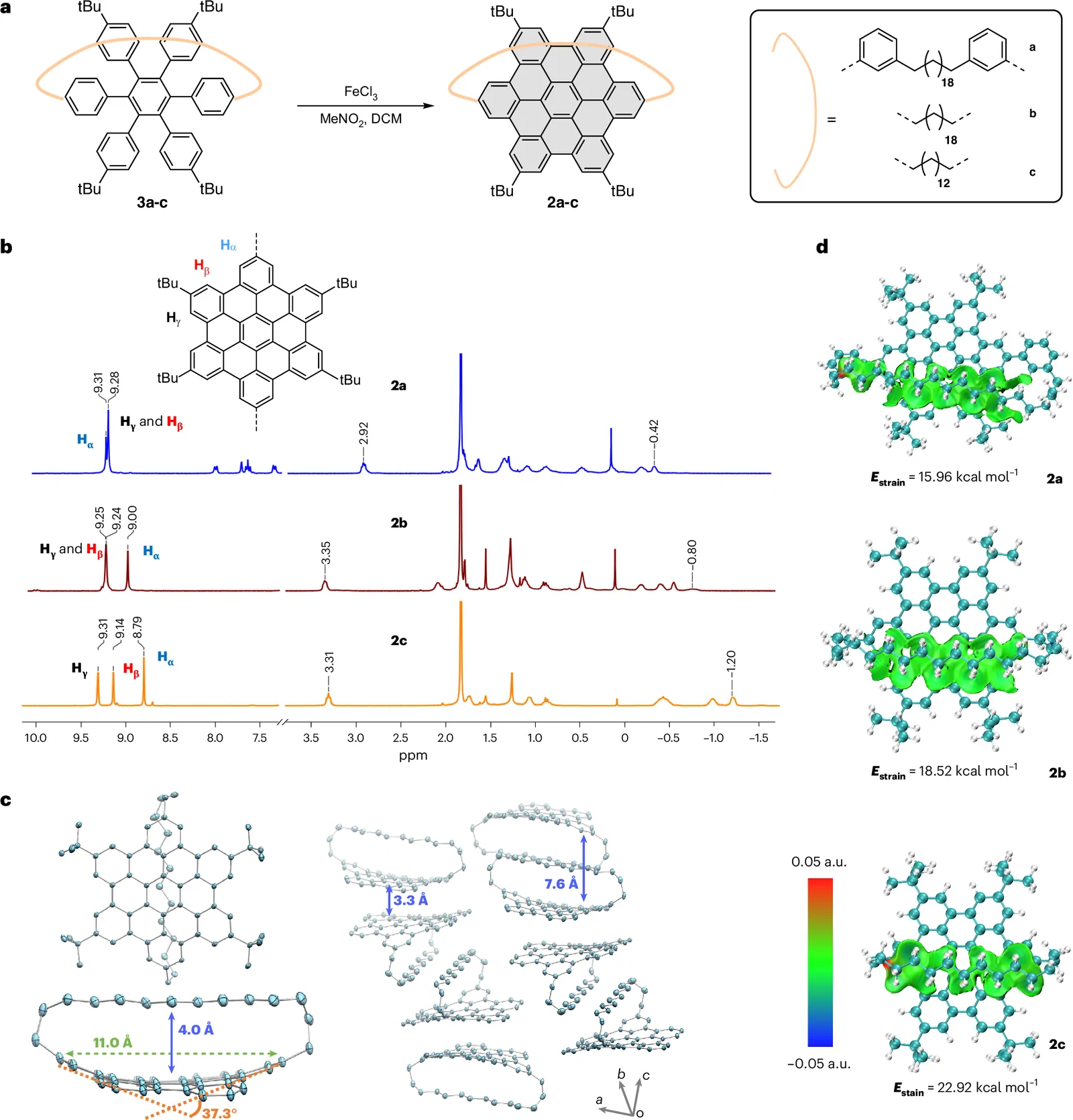
Model compounds synthesis and structural characterization. **a**, The synthesis of model compounds **2a**–**c**. **b**, ^1^H NMR spectra (300 MHz) of **2a**–**c**, recorded at 25 °C in CDCl_3_ at a concentration of 8.0 mmol l^−1^ for **2a**, 7.8 mmol l^−1^ for **2b** and 7.6 mmol l^−1^ for **2c**, respectively. **c**, Single-crystal structure analysis of **2c**; thermal ellipsoids are set at 20% probability, and hydrogen atoms are omitted for clarity. **d**, Contour plots of IGMH iso-surfaces for **2a**–**c**, with green surfaces indicating the non-covalent interactions between the tether chain and main backbone, and the theoretically calculated strain energy of **2a**–**c**.

As illustrated in Fig. 2a, model compounds **2a–c** featuring the HBC core with different cyclic substituents as short segments of the corresponding CsGNRs **1a**–**c** were synthesized. Tetraphenyl-substituted pentabenzenacyclophane (**3a**) and tribenzenacyclophanes (**3b**–**c**) precursors were obtained after multiple-step synthesis, with intramolecular Grubbs metathesis catalysed by second-generation Grubbs catalyst (G2) as the key step (Supplementary Scheme 1). Compounds **3a–c** were then subjected to the Scholl reaction in the presence of FeCl_3_, affording **2a**–**c** in excellent yields of 96%, 95% and 90%, respectively. The chemical identities of **2a**–**c** were first characterized by nuclear magnetic resonance (NMR) spectroscopy. Notably, the ^1^H NMR signals of **2c** exhibit concentration-independent behaviour even at a high concentration of 7.6 mmol l^−1^, indicating that it predominantly exists as a monomeric species in solution. By contrast, self-association behaviour^39,40^ of the HBC backbone was observed in **2a** and **2b**, although the intermolecular interactions are relatively weak (Supplementary Figs. 1–10). Furthermore, ^1^H NMR spectra of **2a**–**c** display non-negligible trends regarding the chemical shift of aromatic and bridge protons as the chain length shortens when measured at comparable concentrations (Fig. 2b). For instance, protons H_α_ in **2c** (8.79 ppm), which features a C_14_ tethered chain, exhibit a pronounced upfield chemical shift of 0.49 and 0.21 ppm compared with that of **2a** (9.28 ppm) with benzene–C_20_–benzene as the bridge and **2b** (9.0 ppm) with C_20_ as the bridge, respectively. Similarly, the signal of the benzylic protons in **2c** (3.31 ppm) is upfield-shifted by 0.04 ppm compared with that in **2b** (3.35 ppm). These results suggest an enhanced magnetic shielding effect from **2a** to **2c** as the bridge length shortens, accounting for the diminishing ring current in the HBC core.

The structures of **2a** and **2c** were further verified through single-crystal X-ray analysis (Fig. 2c and Supplementary Fig. 13), revealing a bent geometry for **2c** with an end-to-end bending angle of 37.3°, which closely matches the theoretically calculated value of 34.5°. This angle is larger than that of **2a** (3.8°) and **2b** (14°), the latter obtained from density functional theory (DFT) calculations, indicating an increased curvature as the bridge length decreases. Consistently, the bridgehead C–C distance decreases from 11.4 Å for **2a** to 11.0 Å for **2c**. Moreover, the average distance between the cyclic alkanediyl chain and the HBC backbone in **2a** is determined to be 3.6 Å; this distance increases to 4.0 Å in **2c**, which is significantly larger than the typical interlayer spacing in graphite (~3.3 Å). In the solid state, **2a** and **2c** exhibit orientation-recognition behaviour, resulting in only face-to-face or back-to-back packing motifs, which are alternatively stacked into a supercolumnar structure. Both the distances of packing dimers increase from 3.3 and 7.5 Å for **2a** to 3.5 and 7.6 Å for **2c**, respectively. Furthermore, the independent gradient model based on the Hirshfeld partition (IGMH) method^41,42^ was visualized between the alkanediyl bridge and the HBC core, which indicates a progressive increase of the van der Waals interactions (green surface) from **2a** to **2c** (Fig. 2d). This result suggests that bending strain increases markedly as the chain length shortens from C_20_ to C_14_, a conclusion further supported by the DFT calculated strain energies of 15.96 kcal mol^−1^ for **2a**, 18.52 kcal mol^−1^ for **2b** and 22.92 kcal mol^−1^ for **2c**, respectively (Supplementary Fig. 23). By contrast, IGMH isosurface analyses of the intermolecular non-covalent interactions reveal a progressive decrease in van der Waals interactions from **2a** to **2c** for both packing dimers (Supplementary Fig. 24). These results elucidate the crucial role of the spatial shielding effect imparted by the cyclic chains in modulating intermolecular interactions.

### Synthesis and characterization of the CsGNRs

**Fig. 3:**
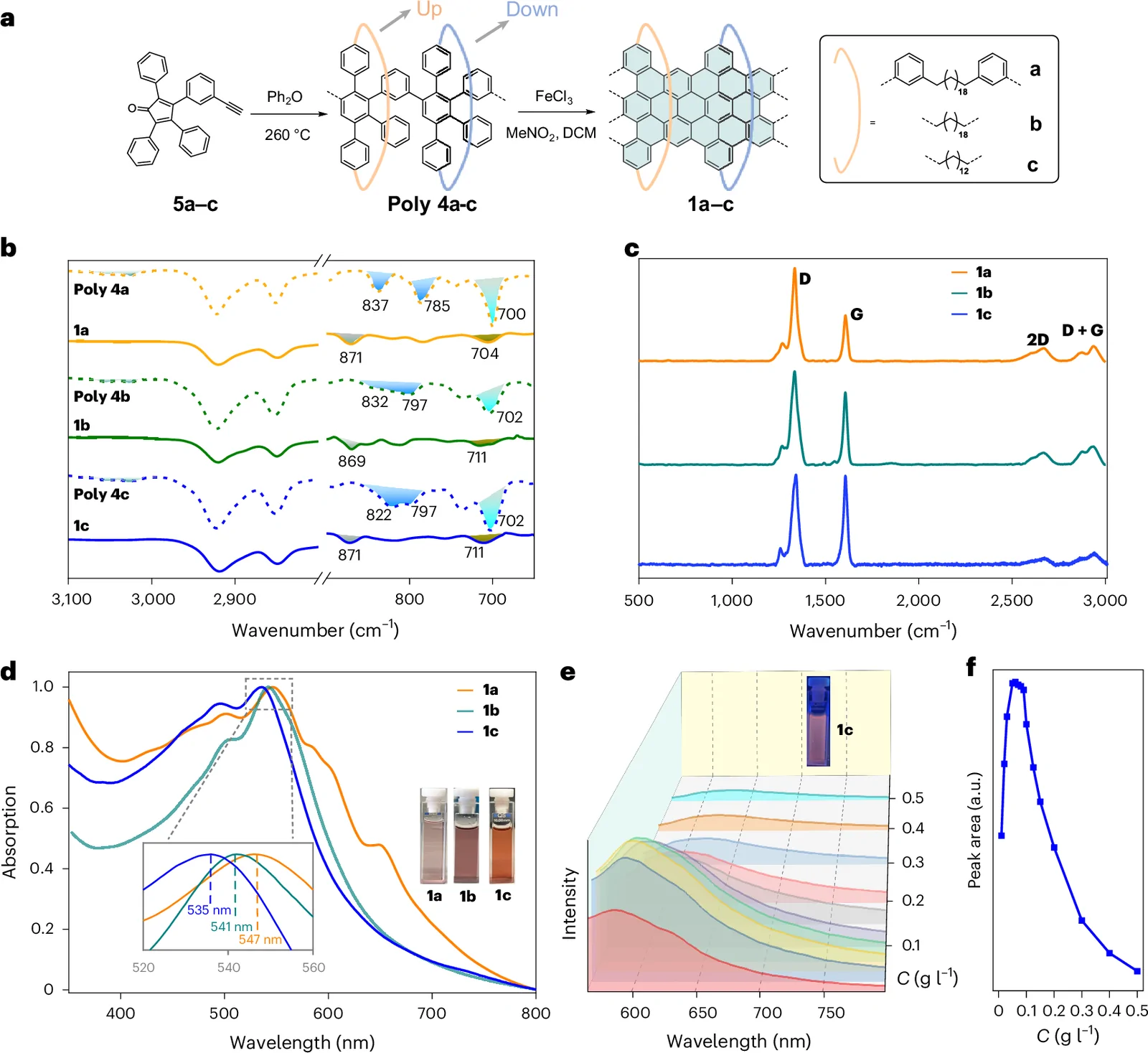
The synthesis and characterization of CsGNRs. **a**, Schematic illustration of the synthesis of CsGNRs **1a**–**c**. **b**, FTIR spectra of **poly 4a**–**c** and **1a**–**c**. **c**, Raman spectra of **1a**–**c**, exited at 532 nm. **d**, UV–visible absorption of **1a**–**c** in NMP solution (*C* ≈ 0.1 g l^−1^), inset images show the eye-visible colour of **1a**–**c** solution. **e**, Concentration-dependent photoluminescence properties of **1c**; the inset shows the emission colour of **1c** in NMP solution (*C* ≈ 0.1 g l^−1^) under 365-nm UV light. **f**, Correlation between the photoluminescence peak areas and concentration *C*. Source data

Encouraged by the successful synthesis of the model compounds, we explored the synthesis of CsGNRs (Fig. 3a). Three cyclophanes, consisting of ethynyl-substituted cyclopentadienone and different cyclic chains, benzene–C_20_–benzene for **5a**, C_20_ for **5b** and C_14_ for **5c**, respectively, were first synthesized as the monomer precursors (Supplementary Scheme 2). Subsequently, we synthesized polyphenylene precursors (**poly 4a**–**c**) featuring varying alkanediyl bridges from monomers **5a**–**c** via AB-type Diels–Alder polymerization. These polymers were probably obtained as a mixture of regioisomers and conformational isomers, given the unsymmetrical monomer structure and the two possible orientations of the tethered chains relative to the backbone, which is expected to promote effective blocking of both *π*-conjugated surfaces of the resulting GNR backbone. The analytical size-exclusion chromatography measurement, calibrated by polystyrene standards, revealed a number-average molar mass (*M*_n_) of 75 kDa for **poly 4a**, 50 kDa for **poly 4b** and 48 kDa for **poly 4c**, respectively (Supplementary Fig. 16). Finally, intramolecular oxidative cyclodehydrogenation using FeCl_3_ as an oxidant afforded the CsGNRs **1a**–**c** as dark-purple solids with high efficiency. The estimated average length of **1a**–**c** is about 50–60 nm based on the *M*_n_ of the corresponding **poly 4a**–**c**.

The successful formation of **1a**–**c** was thoroughly confirmed through a combination of Fourier transform infrared (FTIR), Raman and solid-state NMR spectroscopies. For instance, the FTIR signals from aromatic C–H stretching vibrations at 3,026, 3,054 and 3,082 cm^−1^, as well as the C–H deformation bands at 700, 785 and 837 cm^−1^ in **1a**, were attenuated compared with those in **poly 4a**. In addition, a new peak emerging at 871 cm^−1^ was observed for **1a**, attributed to the SOLO mode (wagging of an isolated aromatic C–H bond neighbored by two C–C bonds) due to the formation of the gulf edge (Fig. 3b). Similar changes were observed for CsGNRs **1b** and **1c**. In their Raman spectra, the typical *D*-like band of **1c** appears at 1,341 cm^−1^, which is slightly red-shifted to 1,336 cm^−1^ in **1a**, and 1,334 cm^−1^ in **1b**, respectively. The *G*-like mode in all of them shows no difference and is located at 1,607 cm^−1^ (Fig. 3c). These results confirm the efficient conversion of **poly 4a**–**c** into CsGNRs. Owing to the steric shielding of the cyclic chains positioned either above or below the GNR backbone, **1a**–**c** can be readily dispersed in common organic solvents. Among them, **1c** exhibits the best dispersibility, reaching a concentration exceeding 0.5 g l^−1^ in its NMP solution. Ultraviolet (UV)/visible–near-infrared absorption spectra reveal the maximum absorption peak at 547 nm for **1a**, 541 nm for **1b** and 535 nm for **1c**, respectively, and the corresponding optical transition energies are 1.8 eV (**1a**), 1.94 eV (**1b**) and 2.0 eV (**1c**) (Fig. 3d). The observed abnormal blue shift in the absorption maximum from **1a** to **1c** can be attributed to the progressively increasing strain arising from the shortening of the bridges from benzene–C_20_–benzene to C_14_. This trend is in good agreement with the findings from their model compounds, reflecting the critical role of strain in tuning optical properties. This conclusion is further corroborated by the concentration-dependent emission property in **1c**, whereas **1a** and **1b** are non-emissive due to the aggregation quenching effect (Fig. 3e). In the photoluminescence spectrum of **1c**, two fluorescence peaks were observed between 590 nm and 630 nm. The photoluminescence intensity at ~590 nm increases with concentration, reaching its maximum at 0.075 g l^−1^, accompanied by a bathochromic shift of the maximum emission peak. Further increasing the concentration causes a decrease in the intensity of both emission peaks, with the high-energy peak disappearing when the concentration exceeds 0.3 g l^−1^. By analysing the correlation between emission peak area and concentration, we determined the saturation point for the single dispersion of **1c** to be approximately 0.03 g l^−1^ (Fig. 3f). These findings highlight the critical role of backbone shielding by strained cyclic chains in enhancing the dispersibility of GNRs and modulating their optical properties.

### Electronic structure calculations and terahertz study of CsGNRs

**Fig. 4:**
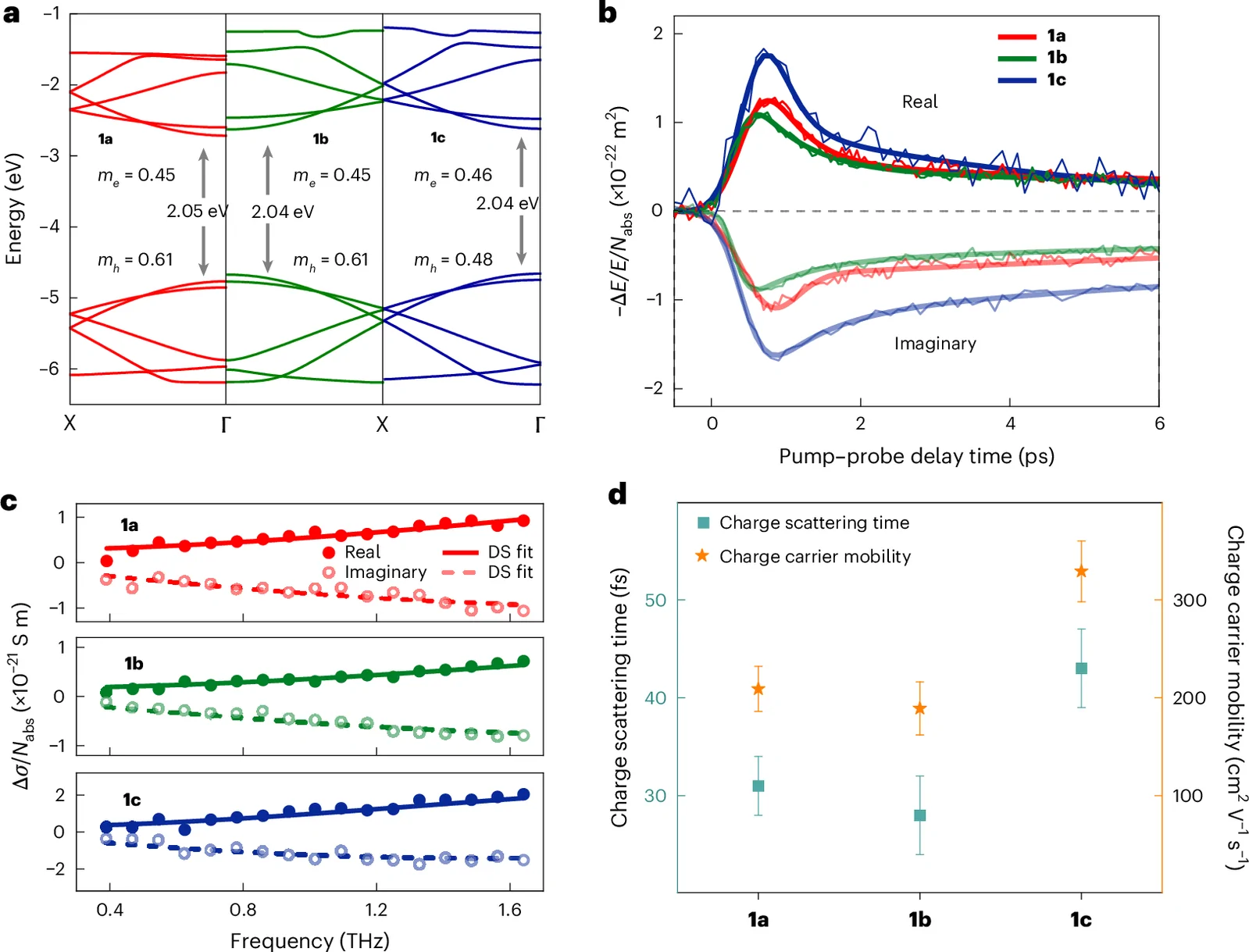
The electronic structure and photoconductivity of CsGNRs. **a**, Band structure and effective mass calculations of **1a**–**c**. **b**, Time-dependent complex (real and imaginary) terahertz photoconductivity of CsGNRs **1a**, **1b** and **1c** at a concentration of 0.5 g l^−1^. For the study, we excite samples by short laser pulses of ~50 fs with a photon energy of 3.1 eV and an absorbed photon density of 1.2 × 10^15^ cm^−2^. **c**, The frequency-resolved complex terahertz conductivity response for **1a**–**c**. The solid lines represent the Drude–Smith (DS) model fittings. **d**, The inferred charge transport parameters of **1a**–**c** from terahertz spectroscopy based on the fitted lines shown in **c**. The squares and stars represent the carrier scattering time and mobility, respectively. The error bars for the carrier scattering time indicate the fitting uncertainties extracted from the Drude–Smith model, while those for the mobility were determined through propagation of the corresponding fitting uncertainties. Source data

We then carried out DFT calculations to gain deeper insight into the impact of varying cyclic chain lengths on the electronic structure of CsGNRs. Considering that **1a**–**c** are probably obtained as a mixture of conformational isomers, where the cyclic chain located above the main backbone is defined as the U isomer and those positioned below the backbone as the D isomer, we selected the UD dimer structure as the unit cell for the infinite GNR calculations. This configuration is both energetically more favourable, as indicated by total potential energy, and in good agreement with experimental results (Supplementary Fig. 25). Geometry optimization indicates that **1c** exhibits the greatest structural distortion among them, primarily due to its high internal bending strain induced by the short-tethered chains (Supplementary Fig. 26). Although the presence of additional phenyl rings in **1a** slightly shifts its energy levels downwards, the bandgaps of **1a**–**c** remain comparable at approximately 2.04 eV (Fig. 4a). Despite the negligible influence of varying cyclic chain lengths on the bandgap, they exhibit a clear impact on band dispersion and, in turn, on the effective mass, which accounts for contributions from both electrons and holes. The reduced effective mass is calculated to be 0.23 *m*_0_ for **1c**, showing a significant decrease compared with **1a** and **1b** (both 0.26 *m*_0_), where *m*_0_ refers to the electron rest mass. This reduction is attributed to the more pronounced edge bending observed in **1c**, and the decreased effective mass is expected to facilitate more efficient charge carrier transport in CsGNRs.

We then used ultrafast terahertz spectroscopy to evaluate the charge carrier transport performance of CsGNRs in solution. In the studies, an optical laser pulse (400 nm, ~50 fs duration) is used to photoinject charge carriers into **1a**–**c** dispersed in 1,2,4-trichlorobenzene, and a terahertz pulse subsequently probes their complex photoconductivity. Figure 4b shows the time-dependent photoconductivity of **1a**–**c**. The fast rise after excitation corresponds to free-carrier generation via above-bandgap excitations, while the subsequent rapid decay in both the real and imaginary parts can be attributed to exciton formation^43,44^. In line with the effective mass trend predicted by the simulation, **1c** exhibits the highest real photoconductivity among the samples. Figure 4c presents the frequency-resolved photoconductivity of **1a**–**c** recorded at *τ* ≈ 1.5 ps after injecting charge carriers. Both absolute amplitudes of the real and imaginary conductivities increase with frequency, consistent with the Drude–Smith model. The Drude–Smith model fitting yields charge scattering times *τ* of 31 ± 3 fs for **1a**, 28 ± 4 fs for **1b** and 43 ± 4 fs for **1c**, respectively, and a shared backscattering parameter *c* of ~0.95 for **1a**–**c** (Fig. 4c). We subsequently estimated the short-range charge carrier mobility *μ*_int_ (= *eτ*/m*) to be 209 ± 23 for **1a**, 190 ± 30 for **1b** and 330 ± 30 cm^2^ V^−1^ s^−1^ for **1c**, respectively, using the fitted charge scattering time along with the reduced effective mass from DFT calculations (Fig. 4d). The intrinsic mobility obtained in **1c** exceeds those of the reported GNRs with the same *π*-conjugated backbone without non-cyclic substituents^8,9,45^, which are typically below 200 cm^2^ V^−1^ s^−1^ (Supplementary Fig. 36). This enhancement is attributed to both a reduced effective mass and an extended scattering time, arising from pronounced edge bending induced by cyclic chain rigidity. By considering the backscattering effect, the corresponding long-range mobility at the direct current limit *μ*_dc_ = *μ*(1 + *c*) was further evaluated to be 11 ± 1 for **1a**, 10 ± 2 for **1b** and 16 ± 4 cm^2^ V^−1^ s^−1^ for **1c**, respectively.

### SET fabrication

**Fig. 5:**
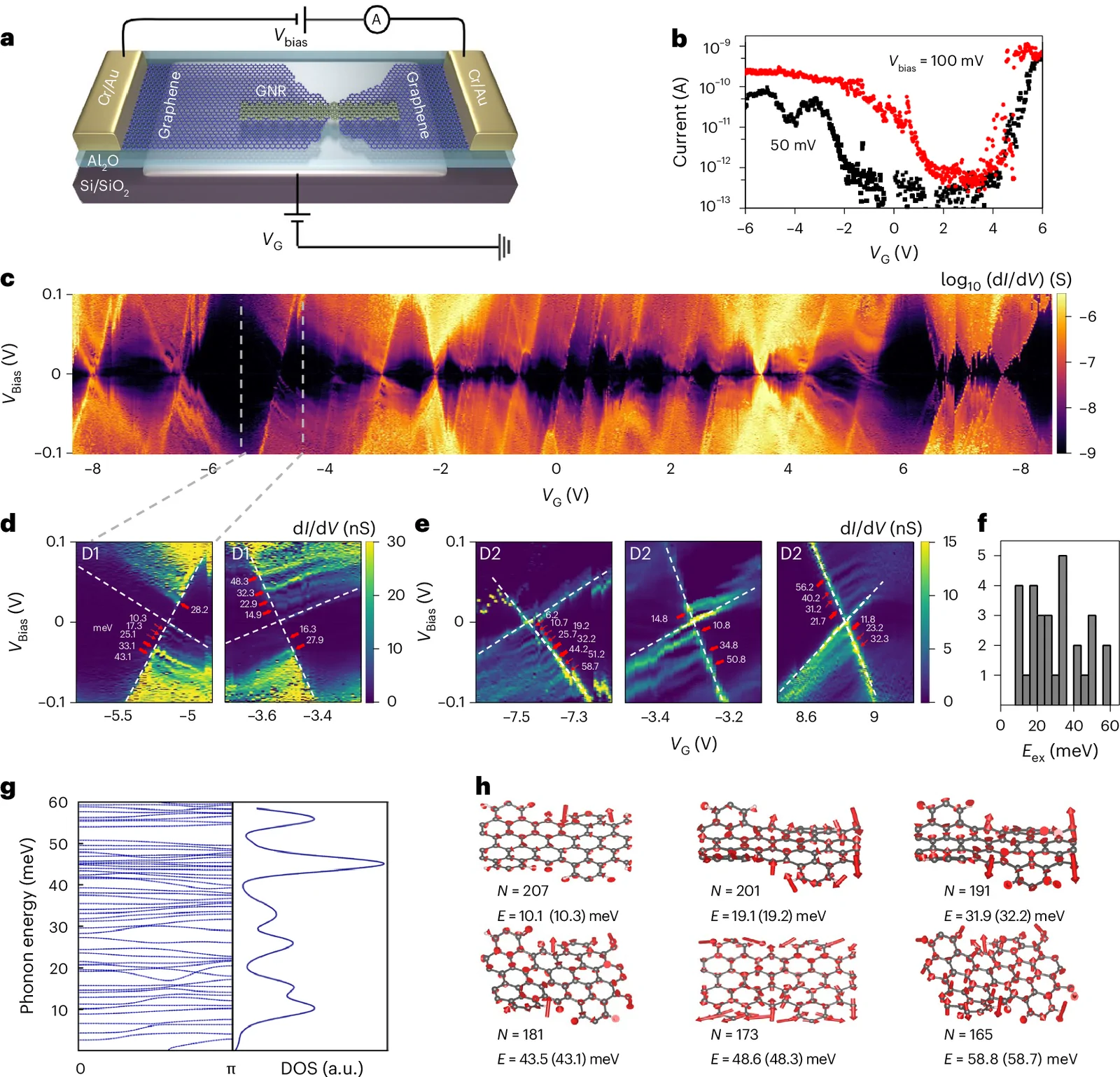
CsGNR-based SET. **a**, Scheme of the electronic devices. **b**, The characteristic curve for a field-effect transistor was obtained with a CsGNR **1c** at room temperature, measured at *V*_Bias_ values of 50 mV and 100 mV. **c**, Single-electron charging behaviour in D1. **d**, Close-up of the box in **c** (left) and additional measurements for D1(right), highlighting the excited states (red arrows). **e**, Charge transport data for D2 with well-resolved excited states. **f**, Histogram of excited-state energies measured from D1 and D2; the bin size is 4 meV. **g**, Phonon band structure of **1c** and the corresponding DOS. **h**, The calculated phonon energies of selected phonon modes (*N* indicates the mode number); the experimentally observed values are shown in brackets. Source data

Due to the singly dispersed feature of **1c**, we further used it to fabricate the three-terminal SETs, enabling detailed investigations of its charge transport behaviour. As shown in Fig. 5a, the SETs comprise a pair of graphene electrodes separated by a nanogap (3–10 nm) formed via electrical breakdown. Beneath the nanogap, a 150-nm-wide Cr/Pd strip serves as the gate electrode, insulated by a 30-nm-thick Al_2_O_3_ layer. The CsGNRs **1c** were deposited onto the substrate using a drop-casting method from its solution^8,18^, achieving a yield of approximately 7% for devices showing an electrical signal. This relatively low yield is a deliberate trade-off. We purposefully used a low-concentration solution for drop-casting to ensure single-GNR bridging rather than multiple ribbons. Attempts to fabricate comparative devices using **1a** and **1b** under identical conditions resulted in near-zero yields (0/100 for **1a**; 1/100 for **1b** with negligible gate modulation; Supplementary Fig. 38). This contrast highlights that the superior dispersibility of **1c** is a strict prerequisite for successful single-ribbon integration.

In these devices fabricated with **1c**, source–drain voltage (*V*_Bias_) and gate voltage (*V*_G_) were applied while measuring the source–drain current. Several transistors exhibited ambipolar behaviour, capable of accessing both n- and p-type states. Figure 5b shows typical transfer curves measured at *V*_Bias_ values of 50 mV and 100 mV, revealing an ON-state current of ~1 nA and an ON/OFF ratio of ~10^3^ (at *V*_Bias_ = 100 mV).

Figure 5c–e presents transport data for two representative devices D1 and D2 measured at 260 mK, in which the differential conductance (d*I*/d*V*) is recorded as a function of *V*_Bias_ and *V*_G_ (so-called stability diagram). The full stability diagrams of D2, D3 and D4 can be found in Supplementary Fig. 37. Figure 5c shows the stability diagram for device D1. Within the *V*_G_ range studied, multiple Coulomb diamonds are observed, with additional energies varying significantly from 10 meV to 100 meV. The variations in additional energies arise from strong quantum confinement effects due to the nanometre-scale dimensions of the GNRs^7,46^, which behave as quantum dots at low temperatures. In addition, we observe some Coulomb diamonds that do not have a crossing point at each of their sides. While we do not yet fully understand the origin of this effect, it may be due to the mixing of the graphene lead states with GNR states^47^, or disorder induced by the environment^48^, such as residues, charge traps in the oxide and so on. A close-up of the boxed region in Fig. 5c reveals a well-resolved crossing point with several resonances parallel to the diamond edges (Fig. 5d, left, red arrows). Notably, these excited states are predominantly observed at negative bias in this regime, probably due to asymmetric coupling between the GNR and the two electrodes. Figure 5d (right) shows additional measurements for D1, where multiple excited states are visible at both positive and negative biases. Figure 5e presents transport data for D2, displaying three distinct regimes with well-resolved excited states. Collectively, the Coulomb diamonds and excited states observed across different samples confirm the successful contacting of individual CsGNRs in our SETs. From the data in Fig. 5d,e, we plot a histogram of excited-state energies measured from D1 and D2, which range from 6 meV to 60 meV (Fig. 5f).

To explain the origin of the excited states observed in the SET regime of devices D1 and D2, we computed the phonon band structure of **1c** and the corresponding density of states (DOS) (Fig. 5g). The phonon band structure exhibits numerous vibrational modes in the 0–60 meV range, closely matching the energy scales of the excited states experimentally observed in D1 and D2, suggesting that the excited states arise from vibrational modes. To visualize the observed vibrational modes, we present six calculated phonon modes with energies between 10.1 meV and 58.8 meV (Fig. 5h). While the experimental and computed values do not exhibit a perfect 1:1 correspondence, they generally agree very well.

Finally, we evaluate the impact of the cyclophane shielding strategy on the GNR–electrode electronic coupling. By applying the Breit–Wigner model to the resonant transport features, we extracted the total tunnel coupling (*Γ*) for **1c** devices, yielding values between 0.18 meV and 2.35 meV (average ~0.93 meV; Supplementary Fig. 38). These values are comparatively lower than those typically reported for unshielded GNRs (for example, 1.3–11.5 meV)^7,46^, indicating that the cyclic chains act as a spatial barrier that partially attenuates charge injection into the *π* backbone. From the Coulomb diamond measurements, the charging energy of our SET devices is determined to be 20–50 meV, resulting in an average *Γ*/*E*_C_ ratio of ~0.05. This indicates that the coupling lies in the weak-coupling regime (*Γ* ≪ *E*_C_), where Coulomb blockade is fully established and well resolved. The corresponding single-electron dwell time *τ*, calculated via *τ* = *ħ*/*Γ*, ranges from 0.28 ps to 3.66 ps (average ~0.71 ps), providing a quantitative reference for future dynamic transport studies. As a result, while the cyclic chains act as a spatial barrier that moderately weakens the tunnel coupling, this effect is not detrimental to SET operation and represents a necessary structural trade-off.

## Conclusion

In summary, we demonstrate that the cyclophane-based shielding strategy—by providing steric protection and introducing internal bending strain to the GNR backbone—offers an effective approach to achieving singly dispersed GNRs, as indicated by the synthesis and investigation of three CsGNRs (**1a**–**c**) with varying cyclic chain lengths. The efficient steric shielding effect provided by the cyclic chains, particularly for **1c** bearing the shortest chain length, is clearly elaborated by the NMR measurements and single-crystal analysis of its model nanographene compound, thus bestowing it with single dispersibility by efficient suppression of *π*–*π* aggregation. As a consequence of the most pronounced bending strain, singly dispersed **1c** exhibits a blue-shifted absorption maximum band compared with **1a** and **1b** with longer bridges, along with an increased optical bandgap of 2.0 eV and concentration-dependent emission behaviour. In addition, the increased bending strain, as the cyclic chain length shortens, leads to a notable reduction in effective mass and a substantial increase in charge carrier mobility up to 74% from **1b** to **1c**. Moreover, singly dispersed **1c** enables the fabrication of SET devices with moderate efficiency, which exhibit the characteristic single-electron transport behaviour at cryogenic temperatures. The cyclophane-based shielding strategy presented in this work provides new insights into modulating the optoelectronic properties of GNRs. For instance, higher conductance can now be achieved either by enhancing charge carrier mobility through precise control of the bridging architecture or by tuning the contact resistance using carbon nanotube electrodes. To improve device reproducibility and scalability, future work will focus on enhancing the integration yield. This could be achieved by utilizing higher-concentration solutions combined with narrower electrodes to avoid multi-ribbon contacts. In addition, electric field-assisted alignment or side-group engineering could enable precise GNR positioning for scalable quantum devices. Most importantly, this strategy enables the formation of singly dispersed GNRs with well-defined structures and supports scalable solution-phase synthesis, both essential for the practical development of quantum electronic applications.

## Methods

### A typical procedure for the synthesis of model compounds 2a, 2b and 2c

A solution of precursor **3** (0.01 mol) in dichloromethane (DCM; 5 ml) was degassed by argon bubbling for 10 min. A suspension of FeCl_3_ (140 mg, 0.85 mmol) in nitromethane (1.0 ml) was added to the degassed solution. After stirring at room temperature for 2 h under continuous argon bubbling, the reaction was quenched by adding methanol, and the reaction mixture was concentrated under vacuum. The crude product was purified by silica gel column chromatography using a DCM/hexane (1:10) eluent, yielding the title compound.

### A typical procedure for the synthesis of poly 4a, poly 4b and poly 4c

A degassed solution of **5** (0.034 mmol) in diphenyl ether (0.2 ml, 170 mM) was refluxed for 24 h using sand heating. The purple colour vanished and the solution turned pale yellow, indicating that the polymerization had been completed. The reaction mixture was then cooled down to room temperature, and methanol (30 ml) was added. The resulting precipitate was collected by membrane filtration, yielding a crude polymer in the form of grey-white solid. This product was then purified through a preparative size-exclusion chromatography system to remove low molecular weight components, affording poly **4a** with *M*_n_ = 75 kDa (Polydispersity Index: 1.2), poly **4b** with *M*_n_ = 50 kDa (Polydispersity Index: 1.3) and poly **4c** with *M*_n_ = 48 kDa (Polydispersity Index: 1.2), respectively.

### A typical procedure for the synthesis of CsGNRs 1a, 1b and 1c

A solution of the polyphenylene precursor **poly 4a**–**c** (10 mg) in DCM (15 ml) was degassed by argon bubbling for 15 min. To the degassed solution was added a suspension of FeCl_3_ (84 equiv.) in nitromethane (1 ml). After being stirred at room temperature for 3 days under continuous bubbling with argon, the reaction was quenched by addition of methanol (30 ml), resulting in the formation of dark-purple precipitate. The precipitates were collected by vacuum filtration and washed intensively with water (50 ml), methanol (30 ml) and tetrahydrofuran (20 ml) to yield CsGNRs as dark-purple powder.

## Online content

Any methods, additional references, Nature Portfolio reporting summaries, source data, extended data, supplementary information, acknowledgements, peer review information; details of author contributions and competing interests; and statements of data and code availability are available at https://doi.org/10.1038/s41557-026-02172-z.

## Supplementary information


Supplementary InformationSupplementary Figs. 1–87, Discussion and Tables 1 and 2.
Supplementary Data 1Coordinates for the optimized structures.


## Source data


Source Data Fig. 3Source data for Fig. 3b–f.
Source Data Fig. 4Source data for Fig. 4a–d.
Source Data Fig. 5Source data for Fig. 5b,f,g.


## Data Availability

All data supporting the findings of this study are available in the Article or its Supplementary Information and are also available from the corresponding authors upon reasonable request. Crystallographic data for the structures reported in this Article have been deposited at the Cambridge Crystallographic Data Centre, under deposition numbers CCDC 2529326 (**2a**) and 2447470 (**2c**). Copies of the data can be obtained free of charge at https://www.ccdc.cam.ac.uk/structures. Source data are provided with this paper. The underlying data for Figs. 3–5 are also available via figshare at https://doi.org/10.6084/m9.figshare.32051151 (ref. ^49^).
