# Supplementary material for: Cyclophane-based shielding strategy for singly dispersed graphene nanoribbons
Source: Nat Chem. 2026 Jun 5;18(9):1602–10. doi: 10.1038/s41557-026-02172-z (PMC13524812; doi:10.1038/s41557-026-02172-z)
Supplement: Supplementary file 1 [file 41557_2026_2172_MOESM1_ESM.pdf]

nature chemistry

Supplementary information https://doi.org/10.1038/s41557-026-02172-z

Cyclophane-based shielding strategy for singly dispersed graphene nanoribbons

In the format provided by the authors and unedited

Table of contents

1. Supplementary Methods **2**

1.1 General Methods and Materials **2**

1.2 Synthestic details **4**

2. Concentration-dependent NMR measurments **23**

3. UV-Vis absorption spectrum of 2a-c **29**

4. Polymers characterization **33**

5. X-ray structure **30**

6. Solid-state NMR analysis **34**

7. DFT calculation **42**

7.1 Model compounds calculations **42**

7.2 CsGNRs calculations **45**

8. THZ spectroscopy **58**

9. Single-Electron Transistor Devices **60**

10. NMR and Maldi-TOF Mass Spectra **64**

11. Supplementary References **89**

1. Supplementary Methods

1.1 General Methods and Materials

All the reagents were obtained from Sigma Aldrich, TCI, abcr, Alfa Aesar, Strem, fluorochem, or chempur. All these chemicals were used as received without further purification. All reactions dealing with air- or moisture-sensitive compounds were carried out in oven-dried glassware under an argon atmosphere. Anhydrous dichloromethane (DCM), toluene, and tetrahydrofuran (THF) were obtained from the MBRAUN MB-SPS-5 solvent purification system. All the sensitive reactions were performed using standard vacuum-line and Schlenk techniques.

Thin layer chromatography (TLC) was performed on silica-coated aluminium sheets with a fluorescence indicator (TLC silica gel 60 F_{254}, purchased from Merck KGaA). Column chromatography was performed on silica (SiO_2, particle size 0.063-0.200 mm, purchased from VWR).

Solution nuclear magnetic resonance (NMR) spectra were recorded using Bruker 300 and Bruker 600 MHz NMR spectrometers at room temperature (23°C). The NMR spectra were recorded using the standard Bruker pulse programs and processed using MestReNova software. Chemical shifts (δ) are expressed in ppm relative to the residual of solvents, $CDCl_3$ (δ (^{1}H) = 7.26 ppm, δ (^{13}C) = 77.0 ppm), CD_2Cl_2 (δ (^{1}H) = 5.32 ppm, δ (^{13}C) = 53.8 ppm), and $C_2D_2Cl_4$ (δ (^{1}H) = 5.98 ppm, δ (^{13}C) = 73.8 ppm). The following abbreviations are used to describe peak patterns as appropriate: s = singlet, d = doublet, t = triplet, q = quartet, and m = multiplet. Coupling constants (J) were recorded in Hertz. Solid-state NMR spectra were acquired in the magnetic field of a Bruker Ascend 11.75 T wide-bore magnet (ν_L(^{1}H) = 500.39 MHz, ν_L(^{13}C) = 125.83 MHz) operated with a Bruker Avance Neo console and a commercial Bruker 1.3 mm HFXY MAS DVT probe. All samples were packed in standard 1.3 mm o.d. (0.7 mm i.d.) ZrO_2 rotors with Vespel top and bottom caps using dry MgO powder as spacer material to confine

the sample to the inner third of the rotor's height. All samples were spun at 62.5 kHz at the magic angle and at ambient temperature. Spectra are externally referenced to solid adamantane spinning at 50.0 kHz with δ (^{1}H) = 1.85 ppm and δ (^{13}C) = 29.47 ppm (low shift signal).[1] Pulse length were calibrated by finding a 180° pulse resulting in a 4 µs 180° pulse for ^{1}H (ν_{rf}(^{1}H) = 125.0 kHz) and a 10 µs 180° pulse for ^{13}C (ν_{rf}(^{13}C) = 50.0 kHz). Quantitative ^{1}H single-pulse spectra were acquired with a total of 16 scans using a 64 s recycle delay. The T_1 relaxation time was determined for each sample using a saturation recovery experiment to ensure full relaxation (T_1 was about 0.5 s to 1 s for the precursors and 20 ms to 50 ms for the GNRs). ^{1}H Hahn echo spectra were acquired using a rotor-synchronous 90°-τ-180°-τ-acq sequence with a total echo time of $2\tau = 32$ µs. ^{13}C single-pulse spectra were acquired with a recycle delay of 3.5 s or 7 s and at least 8192 scans. Low-power continuous wave (CW) ^{1}H decoupling was applied during acquisition with a r. f. power of 28 kHz. 1H-1H DQ/SQ correlation spectra were acquired with rotor-synchronous BaBaXY16 DQ excitation[2] and reconversion of varying length and a z-filter of 160 µs before acquisition. All spectra were normalized to maximum intensity and plotted with home-written Python scripts using matplotlib.

Relative molar masses of polymers were determined by gel permeation chromatography (GPC) with an Agilent Technologies 1260 Infinity LC system equipped with two Resipore columns and RI and UV-vis detection. Chloroform was used as an eluent with a flow rate of 1 mL min^{-1}. The measurements were carried out at 40 ºC. The molar masses were calculated relative to polystyrene standards with low dispersity.

The mass spectrometry analysis of polymers was performed on a Bruker Autoflex Speed MALDI TOF MS (Bruker Daltonics, Bremen, Germany) with the linear model using *trans*-2-[3-(4-*ter*t-butylphenyl)-2-methyl-2-propenylidene]malononitrile (DCTB) as matrix. The high-resolution mass spectrometry (HRMS) analyses were performed either on a Waters Xevo G2-XS

QTOF mass spectrometer or Bruker Autoflex Speed MALDI TOF MS (Bruker Daltonics, Bremen, Germany) using dithranol or *trans*-2-[3-(4-*tert*-Butylphenyl)-2-methyl-2-propenylidene]malononitrile (DCTB) as matrix. Single-crystal X-ray diffraction data were measured by Agilent Supernova Diffractometer.

UV-visible spectra were measured on an Agilent Cary 5000 UV-vis-NIR spectrophotometer by using a 10 mm optical-path quartz cell at room temperature. Fluorescence spectra were recorded at room temperature on a Perkin-Elmer Fluorescence Spectrometer LS 55 using a 10 mm fluorescence quartz cell.

Raman spectroscopy of nanoribbon powder was performed using a Horiba LabRam HR Evolution confocal Raman microscope, a 532 nm laser, a 600 lines/mm grating, an ultra-low frequency module (ULF), and a 100x long working distance objective. The laser spot was widened to 2 × 2 μm², and the power was approximately 55 μW.

1.2 Synthestic details

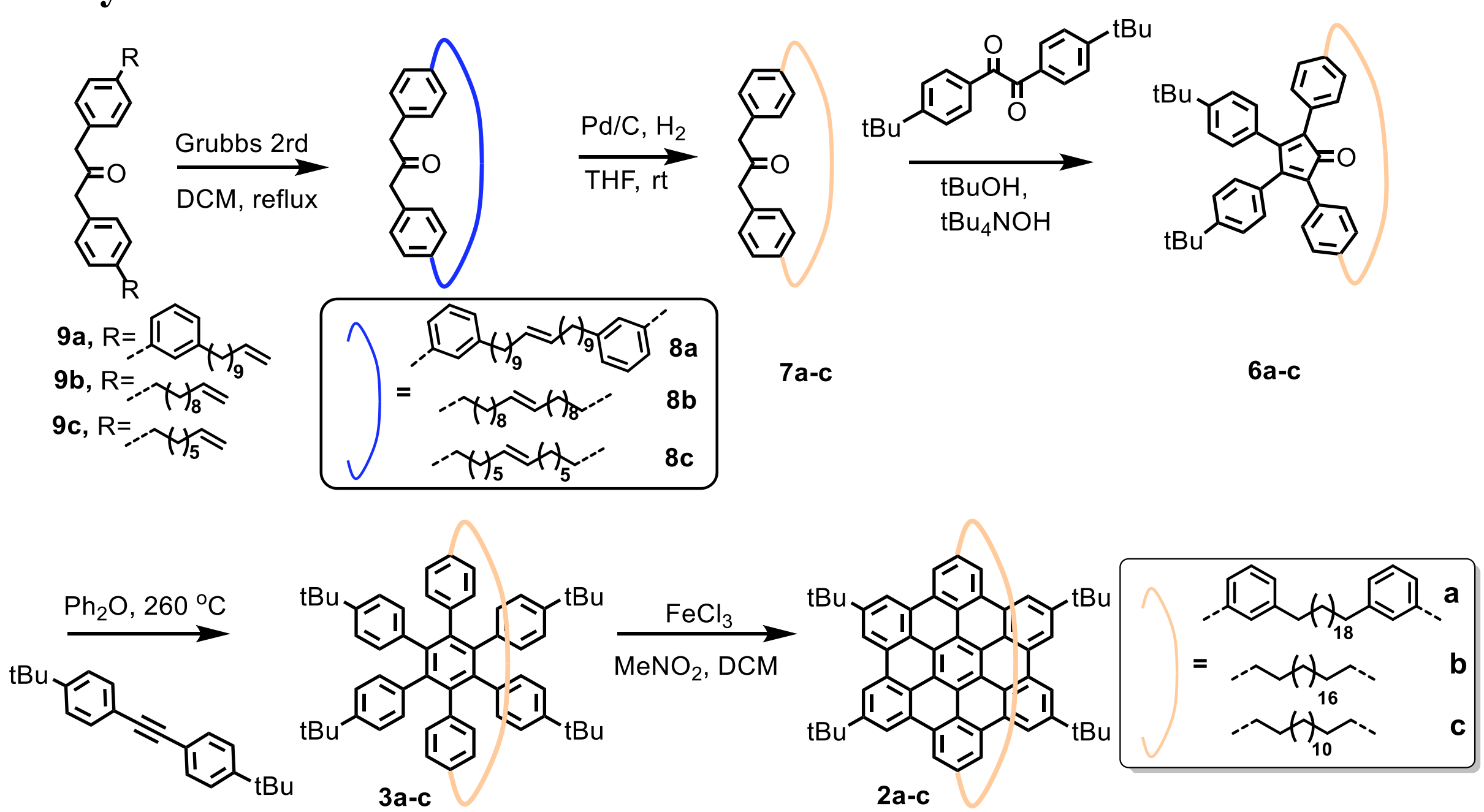

Supplementary Scheme 1. The synthesis route towards model compounds **2a-c**.

Model compounds **2a-c** with different tethered chains, which can be regarded as the short segments of target CsGNRs, were synthesized as shown in

Supplementary Scheme 1. Using compound **2a** as an example, firstly, **9a** was obtained from 1,3-bis(3'-bromo-[1,1'-biphenyl]-4-yl)propan-2-one *via* Negishi coupling reaction with 11-bromounder-1-ene in 75% yield. Subsequently, the intramolecular Grubbs metathesis reaction of **9a** using Grubbs Catalyst 2nd Generation affords **8a** in 80% yield, which was then reduced by Pd/C to provide **7a** in quantitative yield. Treatment **7a** with 1,2-bis(4-(tert-butyl)phenyl)ethane-1,2-dione in the presence of strong base afforded cyclophane-based cyclopentadienone (**6a**) in 60% yield. Diels-Alder reaction of **6a** with 1,2-bis(4-(tert-butyl)phenyl)ethyne provided precursor **3a** in 75% yield. Finally, model compound **2a** was achieved through the Scholl reaction of **3a** using iron(III) chloride ($FeCl_3$) as the oxidant in 96% yield. Following a similar process, model compounds **1b** and **1c** could also be obtained in a yield of 95% and 90%, respectively. **Note: The concentration of zinc reagents for the Negishi coupling should not exceed 1 M.**

Synthesis of 1,3-bis(3'-(undec-10-en-1-yl)-[1,1'-biphenyl]-4-yl)propan-2-one (**9a**)

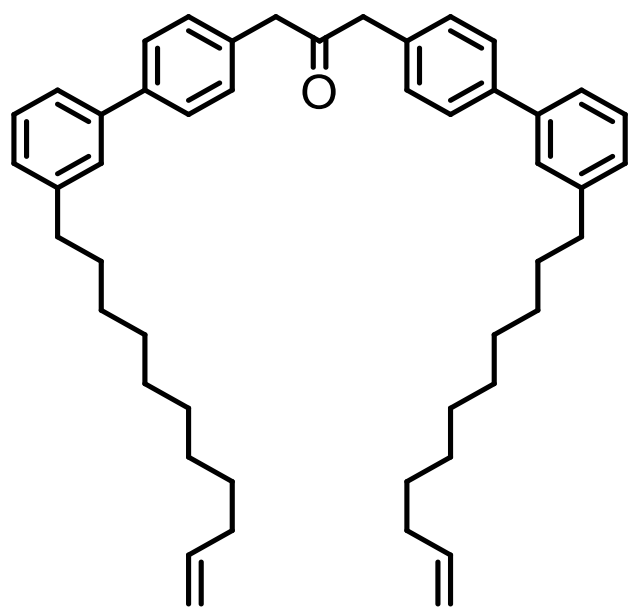

Dry N, N-dimethylacetamide (15 mL) was added to zinc (1.76 g, 26.9 mmol) and iodine (160 mg, 0.62 mmol) in a 100-mL two-necked flask equipped with a condenser, and stirred at room temperature until the purple colour of iodine disappeared. 11-bromoundec-1-ene (3.14g, 13.45 mmol) was then added to the mixture and stirred at 80 °C for 24 h to generate undec-10-en-1-ylzinc bromide. The solution of undec-10-en-1-ylzin bromide was added via cannula to a mixture of 1,3-bis(3'-bromo-[1,1'-biphenyl]-4-yl)propan-2-one (1.00 g, 1.92 mmol) and dichloro[1,1'-bis(diphenylphosphino)-ferrocene]palladium(II) (84 mg, 0.12

mmol) in a 100 mL Schlenk flask. The reaction mixture was stirred at room temperature for 24 h, and then quenched with hydrochloric acid (2 M, 10 mL). The aqueous layer was extracted three times with DCM. The combined organic layers were washed three times with water, dried over magnesium sulfate, and evaporated. The crude material was purified by silica gel column chromatography (DCM/hexane: 1/2) to give the title compound as a white solid (0.96 g, 75% yield). ^{1}H NMR (300 MHz, CD_2Cl_2) δ 7.62 – 7.54 (m, 4H), 7.41 (dt, J = 7.4, 1.6 Hz, 4H), 7.38 – 7.31 (m, 2H), 7.29 – 7.22 (m, 4H), 7.18 (dt, J = 7.3, 1.6 Hz, 2H), 5.83 (ddt, J = 16.9, 10.2, 6.7 Hz, 2H), 5.04 – 4.88 (m, 3H), 3.83 (s, 4H), 2.67 (dd, J = 8.8, 6.7 Hz, 4H), 2.04 (tdd, J = 6.5, 5.3, 1.4 Hz, 4H), 1.70 – 1.59 (m, 4H), 1.43 – 1.28 (m, 24H). ^{13}C NMR (75 MHz, CD_2Cl_2) δ 144.03, 140.93, 140.43, 139.71, 133.62, 130.36, 129.01, 127.83, 127.61, 127.49, 124.60, 114.16, 49.17, 36.37, 34.18, 32.00, 29.91, 29.87, 29.85, 29.73, 29.52, 29.34. HRMS (APCI, positive): Calcd for $C_{49}H_{63}O^+$ $[M+H]^+$ 667.4873; observed 667.4839.

Synthesis of (*Z/E*)1,7(1,3),2,6(1,4)-tetrabenzenacycloheptacosaphan-17-en-4-one (**8a**)

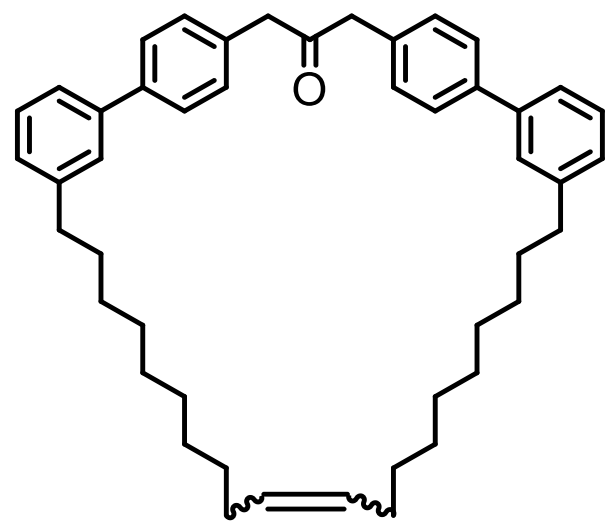

9a (500 mg, 0.749 mmol) was dissolved in 800 mL of dry DCM and bubbled with Ar for 30 min, then Grubbs Catalyst 2nd Generation (160 mg, 0.187 mmol) was added, and the solution was heated to reflux. After 60 h, the reaction was cooled to room temperature, and the solvent was removed by rotary evaporation. The residual solid was purified by silica column chromatography (DCM/hexane:1/2), which yielded the title compound as a transparent oily solid (376 mg, 80%). ^{1}H NMR (300 MHz, $CDCl_3$) δ 7.59 – 7.51 (m, 4H), 7.45 – 7.34 (m, 6H), 7.24 – 7.14

(m, 6H), 5.39 (ddt, J = 5.2, 3.2, 1.6 Hz, 2H), 3.82 (s, 4H), 2.69 (t, J = 7.6 Hz, 4H), 1.99 (d, J = 5.3 Hz, 4H), 1.69 (t, J = 7.3 Hz, 4H), 1.41 – 1.28 (m, 24H). ^{13}C NMR (75 MHz, $CDCl_3$) δ 205.8, 143.3, 140.6, 140.2, 132.8, 130.4, 129.8, 128.6, 127.5, 127.4, 127.2, 124.3, 48.9, 35.9, 32.4, 31.3, 29.4, 29.3, 29.3, 29.0, 28.7. HRMS (APCI, positive): Calcd for $C_{47}H_{58}O^+$ $[M]^+$ 638.4438; observed 638.4452.

Synthesis of 1,7(1,3),2,6(1,4)-tetrabenzenacycloheptacosaphan-4-one (**7a**)

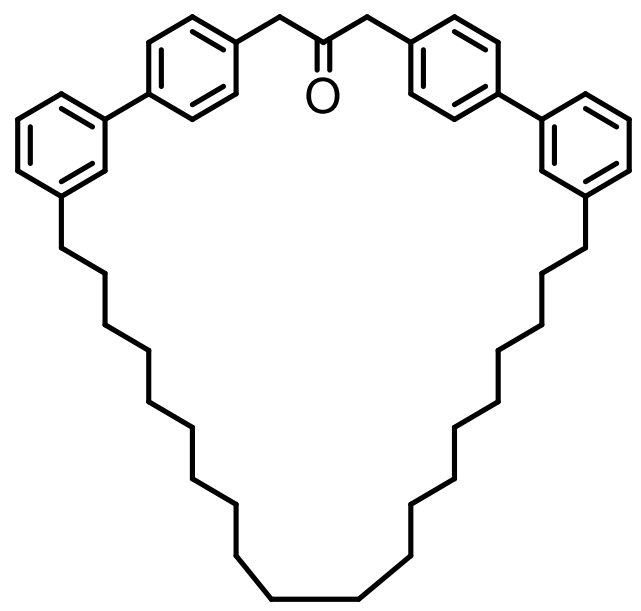

A mixture of **8a** (198 mg, 0.31 mmol) and palladium on carbon (70 mg, 10% Pd) in THF (20 mL) was stirred at room temperature for 4 hours under a hydrogen atmosphere (atmospheric pressure). After the catalyst was removed by filtration over celite, the solvent was evaporated under reduced pressure, and the crude product was purified by silica column chromatography (DCM/hexane: 1/2). The product was obtained as a transparent solid (190 mg, 96%). ^{1}H NMR (300 MHz, CD_2Cl_2) δ 7.60 – 7.54 (m, 4H), 7.41 (dt, J = 6.1, 1.7 Hz, 4H), 7.34 (dd, J = 8.4, 7.3 Hz, 2H), 7.25 – 7.14 (m, 6H), 3.83 (s, 4H), 2.67 (t, J = 7.5 Hz, 4H), 1.65 (m, J = 7.5 Hz, 4H), 1.24 (s, 32H). ^{13}C NMR (75 MHz, CD_2Cl_2) δ 143.96, 140.88, 140.43, 133.62, 130.35, 129.00, 127.95, 127.60, 127.56, 124.52, 49.13, 36.24, 31.76, 29.87, 29.83, 29.79, 29.72, 29.67, 29.63, 29.34. HRMS (APCI, positive): Calcd for $C_{47}H_{60}O^+$ $[M]^+$ 640.4644; observed 640.4638.

Synthesis of 3^4,3^5-bis(4-(*tert*-butyl)phenyl)-1,5(1,3),2,4(1,4)-tetrabenzena-3(1,3)-cyclopentanacyclopentacosaphane-3^1(3^5),3^3-dien-3^2-one (**6a**)

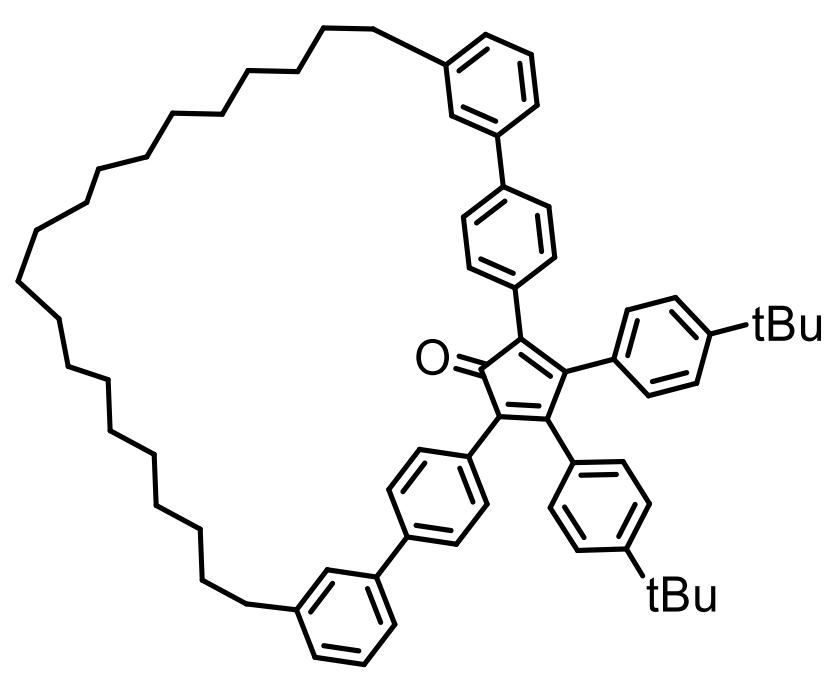

To a solution of **7a** (55 mg, 0.085 mmol) and 1,2-bis(4-(*tert*-butyl)phenyl)ethane-1,2-dione (32.2 mg, 0.1 mmol) in tert-butanol (5 mL) was added a solution of tetrabutylammonium hydroxide (1.0 M, 0.11 mL, 0.11 mmol) at 80 °C. After stirring for 40 min, the reaction was quenched by the addition of hydrochloric acid (2 M, 20 mL), and the reaction mixture was concentrated under a vacuum and then extracted twice with DCM. The combined organic layers were washed twice with water, dried over magnesium sulphate, and evaporated. The crude was purified by silica gel column chromatography (DCM/hexane:1/10) to give the product a purple colour solid (47 mg, 60% yield). ^{1}H NMR (300 MHz, CD_2Cl_2) δ 7.57 – 7.50 (m, 4H), 7.47 (d, J = 1.8 Hz, 2H), 7.42 – 7.29 (m, 8H), 7.29 – 7.21 (m, 4H), 7.18 – 7.11 (m, 2H), 7.04 – 6.90 (m, 4H), 2.69 (t, J = 7.2 Hz, 4H), 1.76 – 1.61 (m, 4H), 1.29 (d, J = 6.8 Hz, 50H). ^{13}C NMR (75 MHz, CD_2Cl_2) δ 201.1, 155.1, 152.3, 143.8, 140.7, 130.9, 130.6, 129.4, 129.0, 128.2, 127.8, 127.0, 125.2, 125.1, 124.2, 35.8, 35.0, 31.3, 31.2, 30.7, 30.60, 30.5, 30.4, 29.9, 29.7, 28.9. HRMS (APCI, positive): Calcd for $C_{69}H_{82}O^{+}$ $[M]^{+}$ 926.6360; observed 926.6352.

Synthesis of 3^2,3^3,3^5,3^6-tetrakis(4-(tert-butyl)phenyl)-1,5(1,3),2,3,4(1,4)-pentabenzenacyclopentacosaphane (**3a**)

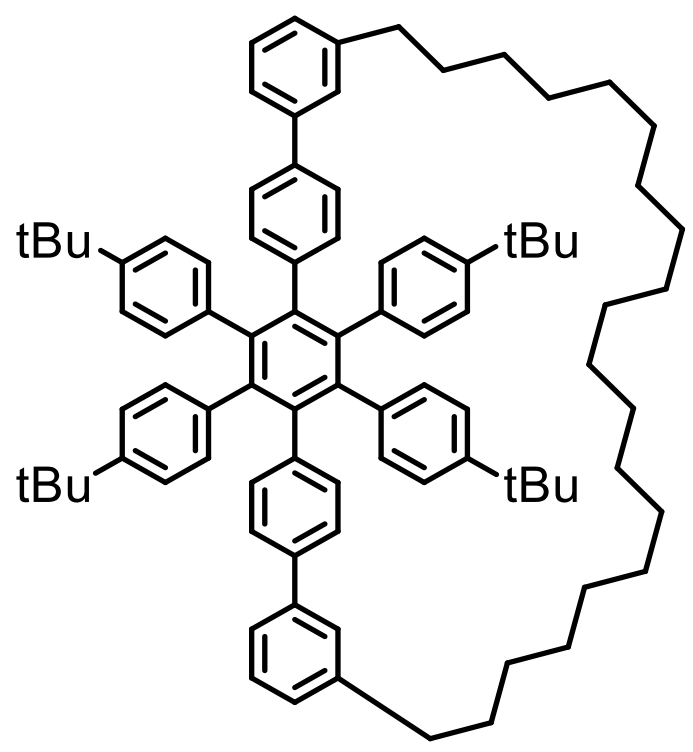

A degassed solution of **6a** (20 mg, 0.021 mmol) and 1,2-bis(4-(tert-butyl)phenyl)ethyne (9.4 mg, 0.032 mmol) in 2 mL diphenyl ether was stirred at 260 °C for 18 hours. After cooling to room temperature, methanol was added to precipitate the crude solid, which was filtered off and washed carefully with methanol to remove residual diphenyl ether. The colourless solid was purified by analytical GPC to afford the desired target as colourless solid (22 mg, 75%). ^{1}H NMR (300 MHz, CD_2Cl_2) δ 7.27 – 7.17 (m, 6H), 7.14 – 7.01 (m, 6H), 6.95 – 6.85 (m, 12H), 6.78 (d, J = 7.9 Hz, 8H), 2.67 (dd, J = 7.8, 4.9 Hz, 4H), 1.65 (t, J = 6.5 Hz, 4H), 1.36 (d, J = 12.3 Hz, 32H), 1.12 (s, 36H). ^{13}C NMR (75 MHz, CD_2Cl_2) δ 148.36, 142.77, 140.78, 138.37, 138.30, 131.47, 128.86, 127.89, 123.81, 123.67, 34.39, 34.08, 31.74, 31.71, 31.68, 31.64, 31.34, 31.18, 30.75, 29.49, 29.33, 27.47. HR-MALDI-TOF: Calcd for $C_{90}H_{108}^{+}$ $[M]^{+}$ 1188.8451; observed 1188.8460.

Synthesis of 2^{5},2^{8},2^{14},2^{17}-tetra-*tert*-butyl-2(2,11)-hexabenzo[*bc*,*ef*,*hi*,*kl*,*no*,*qr*]-coronena-1,3(1,3)-dibenzenacyclotricosaphane (**2a**)

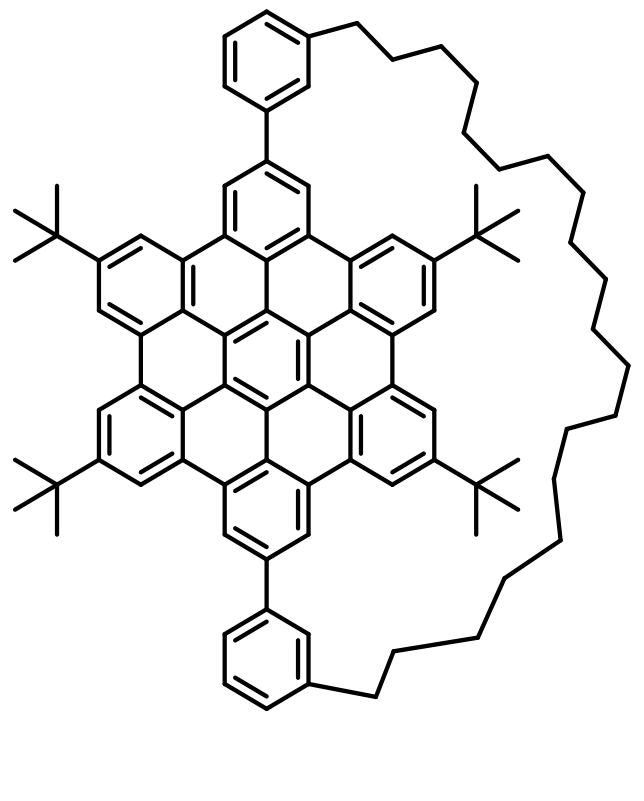

A solution of precursor **3a** (12 mg, 0.01 mol) in DCM (5 mL) was degassed by argon bubbling for 10 min. A suspension of $FeCl_3$ (137 mg, 0.847 mmol) in nitromethane (1.0 mL) was added to the degassed solution. After stirring at room temperature for 2 h under continuous argon bubbling, the reaction was quenched by adding methanol, and the reaction mixture was concentrated under vacuum. The crude was purified by silica gel column chromatography (DCM/hexane:1/10) to provide the product as a pale-yellow solid (11.4 mg, 96%). ^{1}H NMR (300 MHz, $C_2D_2Cl_4$) δ 9.35 (d, J = 12.4 Hz, 12H), 8.05 (d, J = 7.6 Hz, 2H), 7.82 – 7.59 (m, 4H), 7.38 (d, J = 7.6 Hz, 2H), 2.92 (s, 4H), 1.82 (s, 36H), 1.35 (d, J = 21.0 Hz, 8H), 1.20 – 0.74 (m, 9H), 0.30 (d, J = 98.5 Hz, 9H), -0.10 – -0.58 (m, 8H). ^{13}C NMR (75 MHz, $C_2D_2Cl_4$) δ 149.5, 142.9, 141.8, 140.0, 131.0, 130.2, 129.2, 124.7, 123.7, 121.2, 120.4, 120.2, 119.5, 119.2, 35.6, 31.9, 30.30, 30.1, 30.0, 29.5, 28.5, 26.7. HR-MALDI-TOF: Calcd for $C_{90}H_{96}^+$ $[M]^+$ 1176.7507; observed 1176.7511.

Synthesis of 1,3-bis(4-(undec-10-en-1-yl)phenyl)propan-2-one (**9b**)

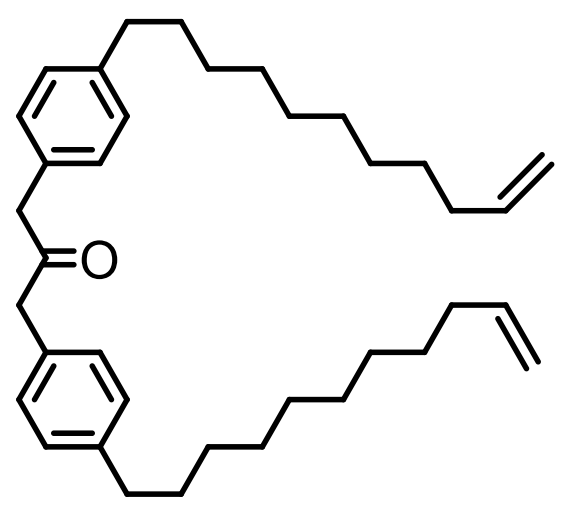

Following the synthesis conditions used for **9a**, compound **9b** was synthesized from undec-10-en-1-ylzinc bromide and 1,3-bis(4-bromophenyl)propan-2-one. Purification by silica gel column chromatography (DCM/hexane: 1/2) to give the title compound as a white solid (0.98 g, 70% yield). ^{1}H NMR (300 MHz, $CDCl_3$) δ 7.12 (d, J = 8.1 Hz, 4H), 7.05 (d, J = 8.1 Hz, 4H), 5.81 (ddt, J = 16.9, 10.2, 6.7 Hz, 2H), 5.05 – 4.87 (m, 4H), 3.67 (s, 4H), 2.57 (dd, J = 8.8, 6.7 Hz, 4H), 2.09 – 1.97 (m, 4H), 1.60 (dd, J = 10.5, 4.9 Hz, 4H), 1.29 (q, J = 4.4, 3.7 Hz, 24H). ^{13}C NMR (75 MHz, $CDCl_3$) δ 206.23, 141.69, 139.23, 131.17, 130.85, 129.33, 128.72, 123.60, 114.09, 48.65, 35.57, 33.80, 31.44, 29.54, 29.51, 29.47, 29.31, 29.27,

29.12, 28.92, 26.82. HRMS (APCI, positive): Calcd for $C_{37}H_{55}O^+$ $[M+H]^+$ 515.4247; observed 515.4233.

Synthesis of (*Z/E*)-1,5(1,4)-dibenzenacyclopentacosaphan-15-en-3-one (**8b**)

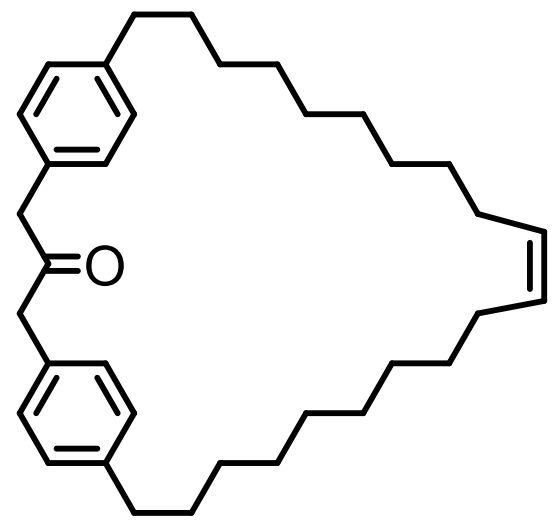

Following the synthesis conditions used for **8a**, compound **8b** was synthesized from **9b**. Purification by silica column chromatography (DCM/hexane:1/2) yielded the title compound as a transparent oily solid (270 mg, 71%). ^{1}H NMR (300 MHz, $CDCl_3$) δ 7.08 (d, J = 8.0 Hz, 4H), 6.98 (d, J = 8.1 Hz, 4H), 5.40 – 5.31 (m, 2H), 3.70 (s, 4H), 2.60 (t, J = 7.1 Hz, 4H), 1.96 (dd, J = 6.0, 2.4 Hz, 4H), 1.66 – 1.56 (m, 4H), 1.33 – 1.22 (m, 24H). ^{13}C NMR (75 MHz, $CDCl_3$) δ 206.81, 141.54, 131.26, 130.77, 130.74, 130.39, 130.04, 129.52, 129.49, 129.44, 128.92, 128.88, 128.81, 128.73, 49.39, 48.81, 35.51, 35.45, 32.47, 32.43, 32.34, 31.53, 31.47, 31.33, 31.22, 31.14, 29.67, 29.57, 29.51, 29.45, 29.37, 29.34, 29.25, 29.22, 28.93, 28.87, 28.84, 28.70, 28.50, 27.18, 22.77. HRMS (APCI, positive): Calcd for $C_{35}H_{50}O^+$ $[M]^+$ 486.3856; observed 486.3840.

Synthesis of 1,5(1,4)-dibenzenacyclopentacosaphan-3-one (**7b**)

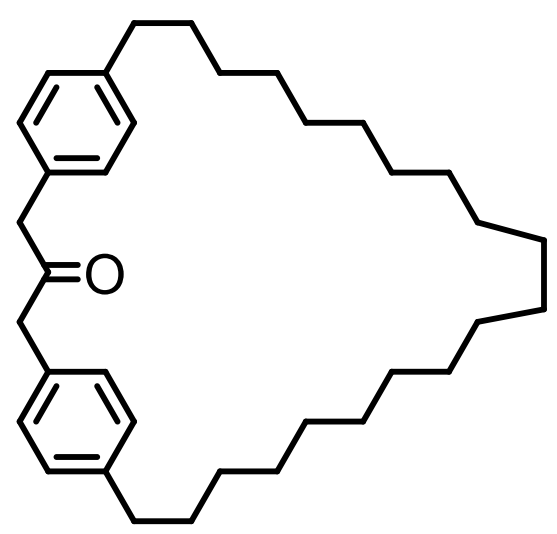

Following the synthesis conditions used for **7a**, compound **7b** was synthesized from **8b**. Purification by silica column chromatography (DCM/hexane: 1/2) to

give the target as a transparent solid (240 mg, 96%). ^{1}H NMR (300 MHz, $CDCl_3$) δ 7.10 (d, J = 8.0 Hz, 4H), 7.00 (d, J = 8.1 Hz, 4H), 3.70 (s, 4H), 2.59 (t, J = 7.2 Hz, 4H), 1.66 – 1.56 (m, 4H), 1.25 (s, 32H). ^{13}C NMR (75 MHz, $CDCl_3$) δ 141.65, 141.63, 131.31, 129.52, 128.93, 48.97, 48.93, 35.53, 35.50, 35.44, 31.22, 29.50, 29.42, 29.37, 29.29, 29.26, 29.18, 29.10, 29.08, 28.98, 28.96, 28.71, 28.68, 28.45. HRMS (APCI, positive): Calcd for $C_{35}H_{53}O^+$ $[M+H]^+$ 489.4091; observed 489.4095.

Synthesis of 2^4,2^5-bis(4-(*tert*-butyl)phenyl)-1,3(1,4)-dibenzena-2(1,3)-cyclopentanacyclotricosaphane-2^1(2^5),2^3-dien-2^2-one (**6b**)

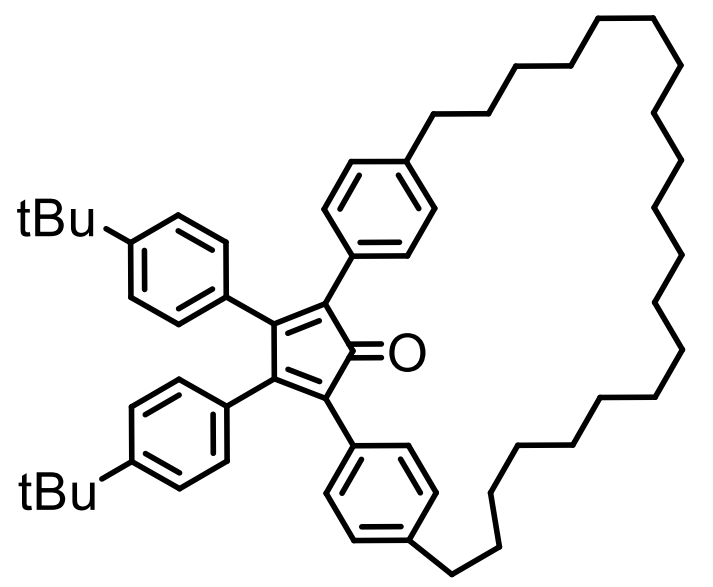

Following the synthesis conditions used for **6a**, compound **6b** was synthesized from **7b**. Purification by silica column chromatography (DCM/hexane:1/10) to give the product a purple colour solid (102 mg, 56% yield). ^{1}H NMR (300 MHz, $CDCl_3$) δ 7.23 – 7.12 (m, 8H), 7.06 (d, J = 8.3 Hz, 4H), 6.94 – 6.85 (m, 4H), 2.67 – 2.56 (m, 4H), 1.60 (s, 4H), 1.28 (s, 18H), 1.24 – 1.18 (m, 32H). ^{13}C NMR (75 MHz, $CDCl_3$) δ 151.41, 141.41, 130.36, 129.91, 129.10, 128.50, 128.36, 124.55, 34.91, 34.63, 31.21, 30.39, 30.16, 30.11, 30.07, 29.62, 29.14, 29.08, 29.01, 27.19. HRMS (APCI, positive): Calcd for $C_{57}H_{75}O^+$ $[M+H]^+$ 775.5812; observed 775.5811.

Synthesis of 2^2,2^3,2^5,2^6-tetrakis(4-(*tert*-butyl)phenyl)-1,2,3(1,4)-tribenzena-cyclotricosaphane (**3b**)

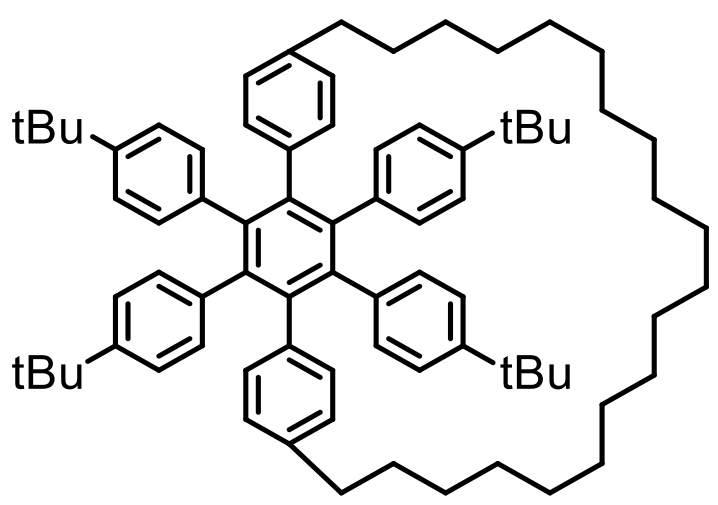

Following the synthesis conditions used for **3a**, compound **3b** was synthesized from **6b**. Purification by analytical GPC to afford the desired target as a colourless solid (19 mg, 75%). ^{1}H NMR (300 MHz, $CDCl_3$) δ 6.91 – 6.43 (m, 24H), 2.50 – 2.34 (m, 4H), 1.41 (t, J = 6.3 Hz, 4H), 1.24 (d, J = 18.2 Hz, 28H), 1.11 (s, 36H), 0.88 (d, J = 14.5 Hz, 4H). ^{13}C NMR (75 MHz, $CDCl_3$) δ 147.3, 140.6, 138.1, 131.2, 123.1, 34.1, 33.3, 31.2, 31.0, 30.6, 29.2, 29.0, 28.7, 27.1, 24.7. HR-MALDI-TOF: Calcd for $C_{78}H_{100}^{+}$ $[M]^{+}$ 1036.7820; observed 1036.7825.

Synthesis of 1^{5},1^{8},1^{14},1^{17}-tetra-*tert*-butyl-1(2,11)-hexabenzo[*bc*,*ef*,*hi*,*kl*,*no*,*qr*]-coronenacyclohenicosaphane (**2b**)

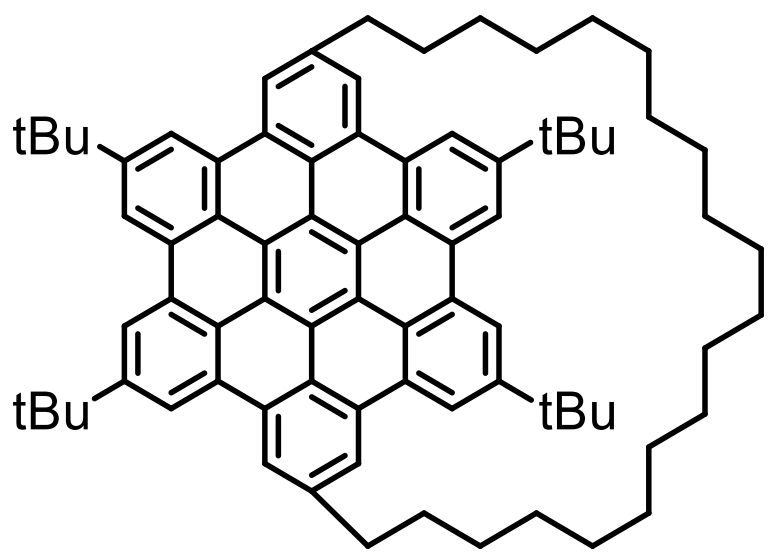

Following the synthesis conditions used for **2a**, compound **2b** was synthesized from **3b**. Purification by silica gel column chromatography (DCM/hexane:1/10) to provide the product as a pale-yellow solid (16 mg, 95%). ^{1}H NMR (300 MHz, $CDCl_3$) δ 9.25 (d, J = 2.1 Hz, 8H), 9.00 (s, 4H), 3.35 (s, 4H), 2.08 (s, 4H), 1.82 (s, 36H), 1.37 (d, J = 6.6 Hz, 8H), 1.10 (d, J = 5.2 Hz, 4H), 0.45 (s, 8H), -0.22 (s, 4H), -0.44 (s, 4H), -0.59 (s, 4H). ^{13}C NMR (75 MHz, $CDCl_3$) δ 148.8, 140.3, 130.7, 130.4, 130.2, 124.0, 122.4, 120.3, 119.0, 118.7, 35.7, 32.1, 29.7, 29.4, 29.1, 27.8, 27.7, 27.1, 25.0. HR-MALDI-TOF: Calcd for $C_{78}H_{88}^{+}$ $[M]^{+}$ 1024.6881; observed 1024.6872.

Synthesis of 1,3-bis(4-(oct-7-en-1-yl)phenyl)propan-2-one (**9c**)

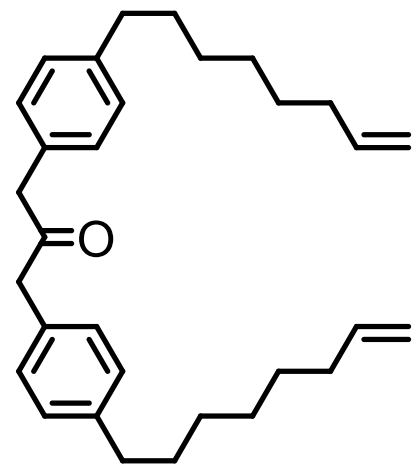

Following the synthesis conditions used for **9a**, compound **9c** was synthesized from oct-7-en-1-ylzin bromide and 1,3-bis(4-bromophenyl)propan-2-one. Purification by silica gel column chromatography (DCM/hexane: 1/2) to give the title compound as a white solid (2.52 g, 72% yield). ^{1}H NMR (300 MHz, $CDCl_3$) δ 7.17 (d, J = 8.2 Hz, 4H), 7.10 (d, J = 8.2 Hz, 4H), 5.97 – 5.75 (m, 2H), 5.13 – 4.91 (m, 4H), 3.72 (s, 4H), 2.63 (dd, J = 8.7, 6.7 Hz, 4H), 2.09 (qd, J = 6.6, 1.4 Hz, 4H), 1.73 – 1.59 (m, 4H), 1.48 – 1.33 (m, 12H).^{13}C NMR (75 MHz, $CDCl_3$) δ 206.18, 141.67, 139.17, 131.35, 129.47, 128.82, 114.33, 48.75, 35.66, 33.88, 31.48, 29.24, 29.08, 28.95. HRMS (APCI, positive): Calcd for $C_{31}H_{43}O^+$ $[M+H]^+$ 431.3308; observed 431.3320.

Synthesis of (*Z/E*)-1,5(1,4)-dibenzenacyclononadecaphan-12-en-3-one (**8c**)

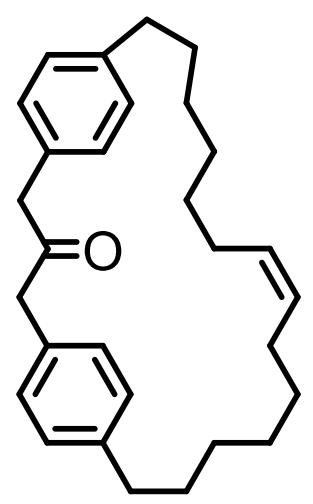

Following the synthesis conditions used for **8a**, compound **8c** was synthesized from **9c**. Purification by silica column chromatography (DCM/hexane:1/2) yielded the title compound as a transparent oily solid (190 mg, 28%). ^{1}H NMR (300 MHz, $CDCl_3$) δ 6.91 (dd, J = 8.2, 2.2 Hz, 4H), 6.79 – 6.72 (m, 4H), 5.38 – 5.29 (m, 2H), 3.64 (s, 4H), 2.51 (t, J = 7.6 Hz, 4H), 2.00 (q, J = 5.4 Hz, 4H), 1.60 – 1.51 (m, 4H), 1.40 – 1.23 (m, 12H). ^{13}C NMR (75 MHz, $CDCl_3$) δ 206.87, 141.25, 130.73, 129.24, 128.45, 49.53, 35.00, 32.17, 31.27, 28.97, 28.52, 28.02.

HRMS (APCI, positive): Calcd for $C_{29}H_{39}O^+$ $[M+H]^+$ 403.2995; observed 403.2986.

Synthesis of 1,5(1,4)-dibenzenacyclononadecaphan-3-one (**7c**)

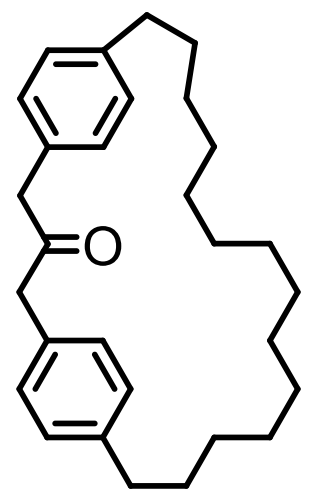

Following the synthesis conditions used for **7a**, compound **7c** was synthesized from **8c**. Purification by silica column chromatography (DCM/hexane: 1/2) provided the product as a transparent solid (176 mg, 98%). ^{1}H NMR (300 MHz, $CDCl_3$) δ 7.02 (d, J = 8.0 Hz, 4H), 6.84 (d, J = 8.0 Hz, 4H), 3.65 (s, 4H), 2.57 (t, J = 7.1 Hz, 4H), 1.60 (q, J = 7.0 Hz, 4H), 1.25 (d, J = 5.8 Hz, 20H). HRMS (APCI, positive): Calcd for $C_{29}H_{40}O$ $[M]^+$ 404.3079; observed 404.3072.

Synthesis of 2^4,2^5-bis(4-(*tert*-butyl)phenyl)-1,3(1,4)-dibenzena-2(1,3)-cyclopentanacycloheptadecaphane-2^1(2^5),2^3-dien-2^2-one (**6c**)

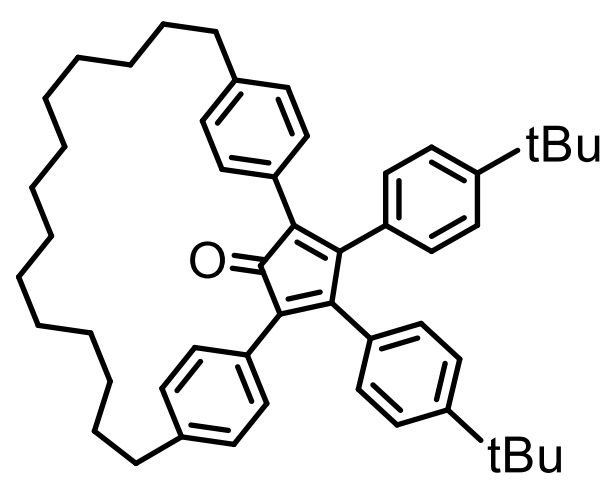

Following the synthesis conditions used for **6a**, compound **6c** was synthesized from **7c**. Purification by silica gel column chromatography (DCM/hexane:1/10) to give the product a purple colour solid (103 mg, 50%). ^{1}H NMR (300 MHz, $CDCl_3$) δ 7.22 – 7.01 (m, 16H), 2.69 – 2.57 (m, 4H), 1.67 – 1.54 (m, 4H), 1.26 (s, 18H), 1.13 (t, J = 7.2 Hz, 4H), 0.95 (d, J = 23.8 Hz, 12H), 0.75 (d, J = 7.6 Hz, 4H). ^{13}C NMR (75 MHz, $CDCl_3$) δ 200.3, 152.81, 151.34, 141.82, 130.45, 130.42,

130.20, 129.28, 129.12, 129.10, 128.42, 128.39, 126.63, 124.54, 124.49, 35.93, 34.62, 31.18, 30.99, 30.38, 30.12, 29.57, 29.41, 27.91.

Synthesis of 2^2,2^3,2^5,2^6-tetrakis(4-(tert-butyl)phenyl)-1,2,3(1,4)-tribenzenacycloheptadecaphane (**3c**)

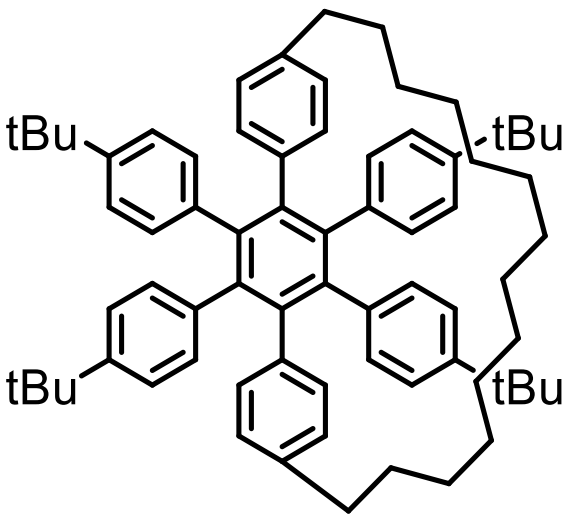

Following the synthesis conditions used for **3a**, compound **3c** was synthesized from **6c**. Purification by analytical GPC to afford the desired target as a colourless solid (46 mg, 68%). ^{1}H NMR (300 MHz, $CDCl_3$) δ 6.75 (q, J = 8.4 Hz, 20H), 6.53 (d, J = 8.7 Hz, 4H), 2.40 – 2.24 (m, 4H), 1.34 – 1.24 (m, 4H), 1.20 – 1.12 (m, 4H), 1.05 (s, 16H), 1.04 (s, 36H). ^{13}C NMR (75 MHz, $CDCl_3$) δ 147.55, 140.47, 138.67, 138.12, 137.81, 131.38, 131.31, 126.70, 123.02, 34.50, 34.09, 34.06, 31.93, 31.26, 31.23, 30.96, 30.83, 29.89, 29.82, 27.25. HR-MALDI-TOF: Calcd for $C_{72}H_{88}^+$ $[M]^+$ 952.6881; observed 952.6890.

Synthesis of 1^5,1^8,1^{14},1^{17}-tetra-*tert*-butyl-1(2,11)-hexabenzo[*bc*,*ef*,*hi*,*kl*,*no*,*qr*]-coronenacyclopentadecaphane (**2c**)

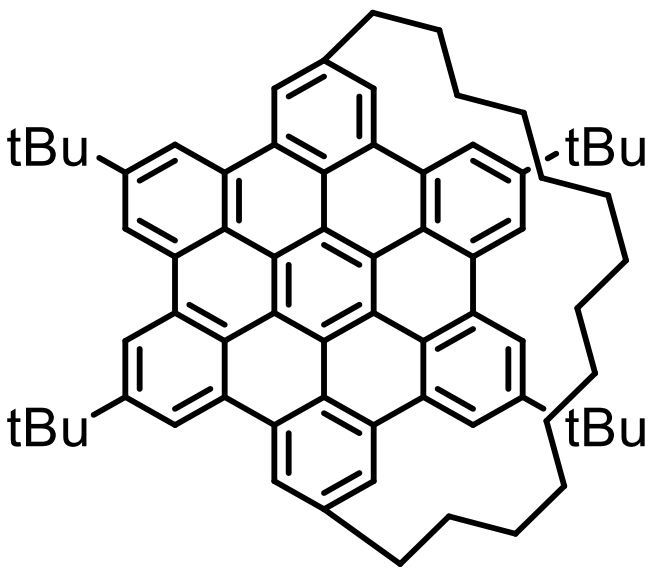

Following the synthesis conditions used for **2a**, compound **2c** was synthesized from **3c**. Purification by silica gel column chromatography (DCM/hexane:1/10) to provide the product as a pale-yellow solid (18 mg, 92%), single crystals suitable

for measurements were grown by diffusing n-hexane vapor into its DCM solution. ^{1}H NMR (300 MHz, $CDCl_3$) δ 9.31 (d, J = 1.7 Hz, 4H), 9.13 (d, J = 1.5 Hz, 4H), 8.79 (s, 4H), 3.31 (t, J = 6.2 Hz, 4H), 1.83 (s, 36H), 1.74 (s, 4H), 1.06 (s, 4H), -0.43 (s, 8H), -0.98 (s, 4H), -1.20 (s, 4H). ^{13}C NMR (75 MHz, $CDCl_3$) δ 149.2, 140.9, 131.0, 130.7, 130.1, 124.9, 123.8, 120.0, 119.6, 118.6, 35.8, 32.1, 30.4, 30.0, 29.8, 29.70. HR-MALDI-TOF: Calcd for $C_{72}H_{76}^{+}$ $[M]^{+}$ 940.5942; observed 940.5941.

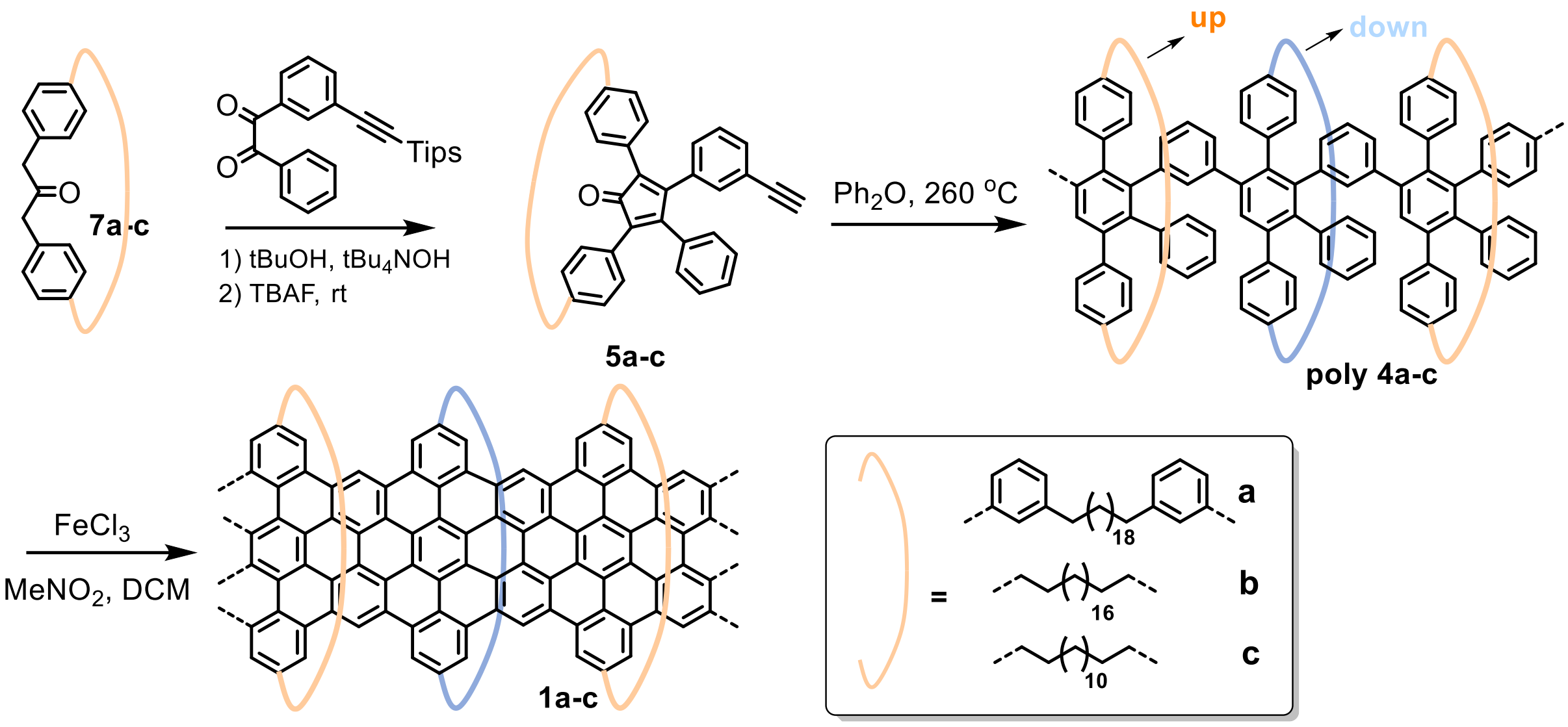

Supplementary Scheme 2. The synthesis route towards CsGNRs **1a-c**.

Synthesis of 3[4]-(3-ethynylphenyl)-3[5]-phenyl-1,5(1,3),2,4(1,4)-tetrabenzena-3(1,3)-cyclopentanacyclopentacosaphane-3[1](3[5]),3[3]-dien-3[2]-one (**5a**)

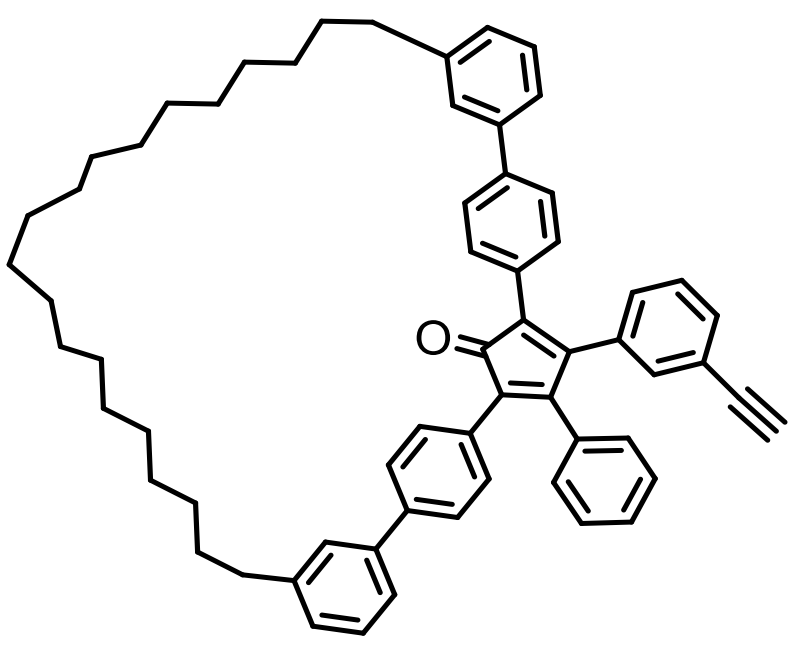

To a solution of **7a** (150 mg, 0.234 mmol) and 1-phenyl-2-(3-((triisopropylsilyl)ethynyl)phenyl)ethane-1,2-dione (110 mg, 0.281 mmol) in tert-

butanol (5 mL) was added a solution of tetrabutylammonium hydroxide (1.0 M, 0.2 mL, 0.2 mmol) at 90 °C. After stirring for 40 min the reaction was quenched by the addition of hydrochloric acid (2 M, 20 mL), and the reaction mixture was concentrated under a vacuum and then extracted twice with DCM. The combined organic layers were washed twice with water, dried over magnesium sulphate, and evaporated. The crude was purified by a flash column to give the product as purple colour solid. Then, the obtained solid was dissolved in anhydrous THF (10 mL), and tetra-n-butylammonium fluoride (1 M in THF, 0.1 mL) was added dropwise. After stirring at room temperature for 1 h, methanol was added to the reaction mixture and solvent was removed under vacuum. Purification by silica gel column chromatography (eluent: hexane/DCM = 3/1) yielded compound **5a** as a purple powder (80 mg, 50%). ^{1}H NMR (300 MHz, CD_2Cl_2) δ 7.45 (ddd, J = 8.6, 4.4, 2.0 Hz, 4H), 7.40 – 7.13 (m, 15H), 7.13 – 7.03 (m, 3H), 6.97 (ddd, J = 7.0, 4.3, 1.5 Hz, 3H), 2.99 (d, J = 1.5 Hz, 1H), 2.60 (t, J = 7.2 Hz, 4H), 1.65 – 1.51 (m, 4H), 1.28 – 1.17 (m, 32H). ^{13}C NMR (75 MHz, CD_2Cl_2) δ 200.71, 143.85, 140.61, 132.89, 132.51, 130.86, 130.13, 129.60, 129.03, 128.68, 128.55, 128.27, 127.76, 127.11, 127.05, 124.21, 122.44, 78.04, 35.77, 31.16, 30.66, 30.60, 30.45, 30.02, 29.84, 29.71, 28.88. HRMS (APCI, positive): Calcd for $C_{63}H_{67}O^+$ $[M+H]^+$ 839.5186; observed 839.5170.

Synthesis of 2^4-(3-ethynylphenyl)-2^5-phenyl-1,3(1,4)-dibenzena-2(1,3)-cyclopentanacyclohenicosaphane-2^1(2^5),2^3-dien-2^2-one (**5b**)

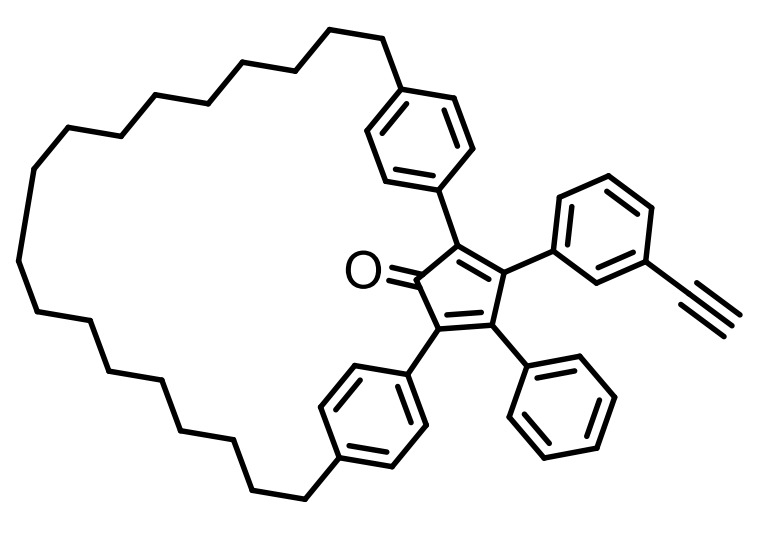

Following the synthesis conditions used for **5a**, compound **5b** was synthesized from **7b**. Purification by silica gel column chromatography (eluent: hexane/DCM = 3/1) yielded compound **5b** as a purple powder (65 mg, 47% in two steps). ^{1}H

NMR (300 MHz, CD_2Cl_2) δ 7.41 – 7.01 (m, 17H), 3.05 (s, 1H), 2.69 – 2.57 (m, 4H), 1.62 (dd, J = 7.8, 4.8 Hz, 4H), 1.14 (dd, J = 8.3, 6.4 Hz, 4H), 1.02 – 0.88 (m, 12H), 0.81 – 0.59 (m, 4H). ^{13}C NMR (75 MHz, CD_2Cl_2) δ 142.9, 142.8, 134.3, 133.5, 132.9, 132.3, 130.4, 130.4, 130.1, 129.6, 129.0, 128.9, 128.3, 128.2, 122.1, 83.2, 77.9, 36.2, 31.5, 30.8, 30.6, 30.0, 29.8, 28.3. HRMS (APCI, positive): Calcd for $C_{49}H_{55}O^+$ [M+H]$^+$ 659.4247; observed 659.4240.

Synthesis of 2^4-(3-ethynylphenyl)-2^5-phenyl-1,3(1,4)-dibenzena-2(1,3)-cyclopentanacycloheptadecaphane-2^1(2^5),2^3-dien-2^2-one (**5c**)

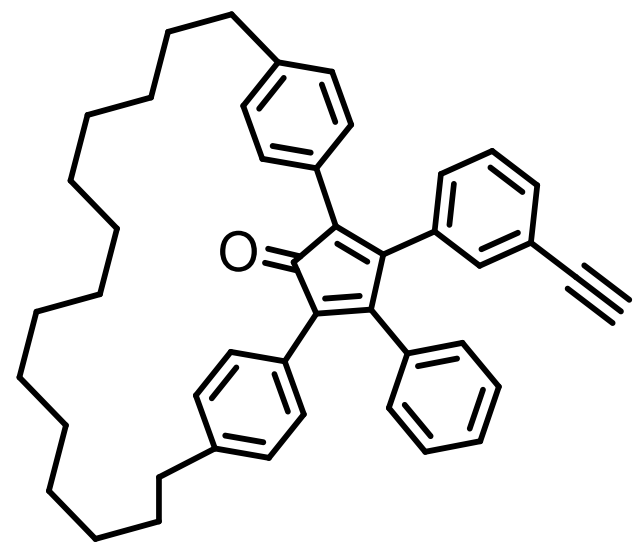

Following the synthesis conditions used for **5a**, compound **5c** was synthesized from **7c**. Purification by silica gel column chromatography (eluent: hexane/DCM = 3/1) yielded compound **5c** as a purple powder (30 mg, 10% in two steps). ^{1}H NMR (300 MHz, CD_2Cl_2) δ 7.41 – 7.01 (m, 17H), 3.06 (s, 1H), 2.69 – 2.57 (m, 4H), 1.62 (dd, J = 7.8, 4.8 Hz, 4H), 1.14 (dd, J = 8.3, 6.4 Hz, 4H), 1.02 – 0.88 (m, 12H), 0.81 – 0.59 (m, 4H). ^{13}C NMR (75 MHz, CD_2Cl_2) δ 142.9, 142.8, 134.3, 133.5, 132.9, 132.3, 130.4, 130.4, 130.1, 129.6, 129.0, 128.9, 128.3, 128.2, 122.1, 83.2, 77.9, 36.2, 31.5, 30.8, 30.6, 30.0, 29.8, 28.3. HRMS (APCI, positive): Calcd for $C_{45}H_{47}O^+$ [M+H]$^+$ 603.3621; observed 603.3605.

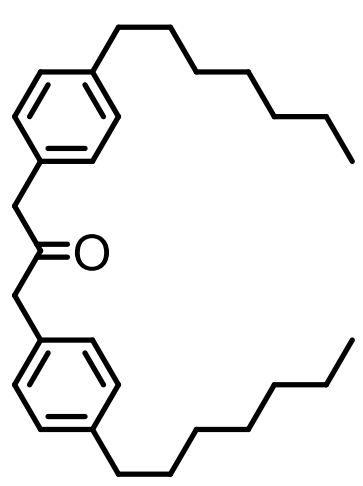

Following the synthesis conditions used for **9a**, compound **9d** was synthesized from oct-7-en-1-ylzin bromide and 1,3-bis(4-bromophenyl)propan-2-one.

Purification by silica gel column chromatography (DCM/hexane: 1/2) to give the title compound as a white solid (1.2 g, 70% yield).

^{1}H NMR (300 MHz, $CDCl_3$) δ 7.12 (d, J = 8.2 Hz, 4H), 7.05 (d, J = 8.1 Hz, 4H), 3.67 (s, 4H), 2.58 (dd, J = 8.8, 6.7 Hz, 4H), 1.66 – 1.55 (m, 4H), 1.30 (dq, J = 14.2, 5.7, 4.6 Hz, 16H), 0.92 – 0.84 (m, 6H). ^{13}C NMR (76 MHz, $CDCl_3$) δ 206.25, 141.71, 131.17, 129.33, 128.72, 48.66, 35.57, 31.81, 31.45, 29.28, 29.17, 22.66, 14.09. HR-MALDI-TOF: Calcd for $C_{29}H_{42}O^+$ $[M]^+$ 406.3230; observed 406.3218.

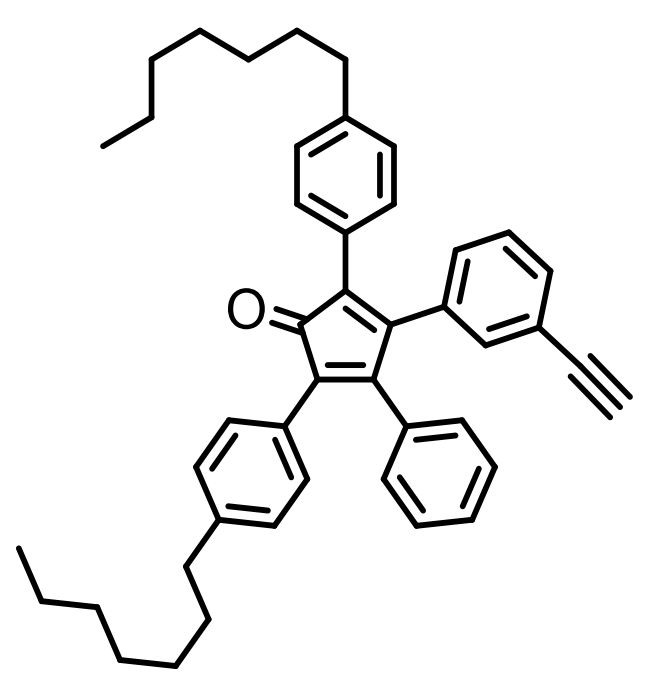

Following the synthesis conditions used for **5a**, compound **5d** was synthesized from **7d**. Purification by silica gel column chromatography (eluent: hexane/DCM = 3/1) yielded compound **5d** as a purple powder (120 mg, 52% in two steps).

^{1}H NMR (300 MHz, $CDCl_3$) δ 7.41 – 7.27 (m, 2H), 7.26 – 7.02 (m, 12H), 6.97 – 6.87 (m, 3H), 2.97 (s, 1H), 2.56 (td, J = 7.9, 2.5 Hz, 4H), 1.60 (d, J = 7.0 Hz, 4H), 1.33 – 1.24 (m, 16H), 0.91 – 0.86 (m, 6H). ^{13}C NMR (76 MHz,$CDCl_3$) δ 152.53, 142.59, 142.41, 132.74, 131.94, 129.94, 129.92, 129.77, 129.25, 128.49, 128.24, 128.14, 128.08, 127.90, 127.63, 35.79, 31.82, 31.20, 29.34, 29.31, 29.18, 22.68, 14.12. HR-MALDI-TOF: Calcd for $C_{45}H_{48}O^+$ $[M]^+$ 604.3700; observed 604.3680.

A typical procedure for the synthesis of **poly 4a-c and poly Ref**

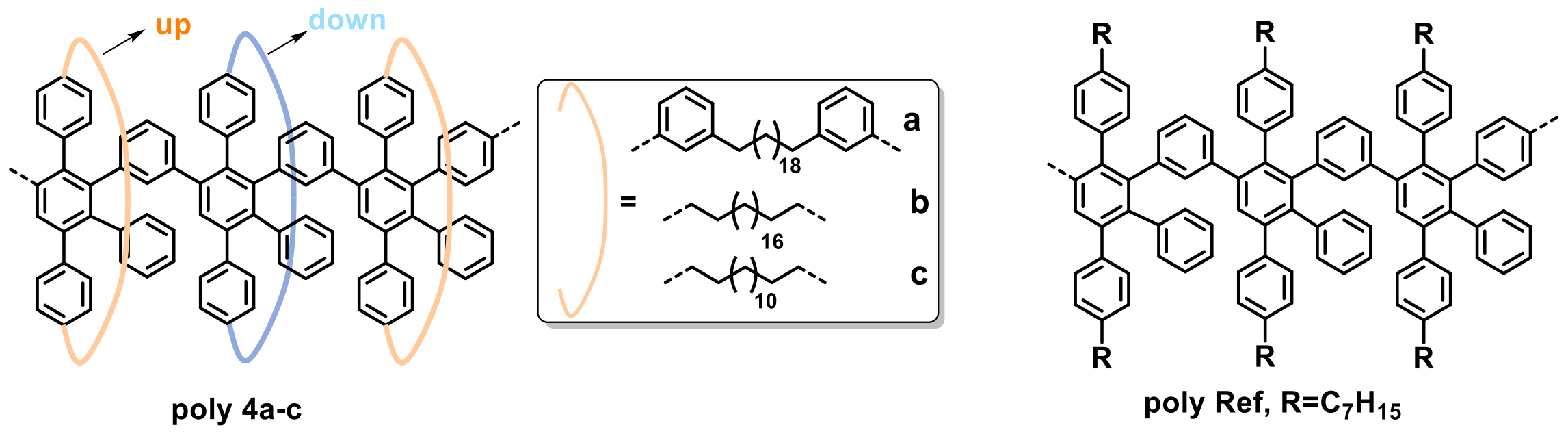

A degassed solution of **5a-c** (0.033 mmol) in diphenyl ether (0.2 mL, 170 mM) was refluxed for 24 h using sand heating. The purple color disappeared and the solution turned pale yellow, indicating the completion of the polymerization. After cooling down to room temperature, methanol was added. The precipitates were filtrated with a membrane filter to obtain a crude polymer as a grey-white solid. The crude polymer was fractionated by using recycling preparative SEC system to remove low-molecular-weight fractions, leading to poly **4a** with M_n = 75 kDa (PDI=1.2), poly **4b** with M_n = 50 kDa (PDI=1.3), and poly **4c** with M_n = 48 kDa, (PDI=1.2), respectively. It should be noted that poly **4a–c** are likely obtained as a mixture of regioisomers and conformational isomers, given the unsymmetrical monomer backbone and the two possible orientations of the cyclic alkyl chains.

A typical procedure for the synthesis of **CsGNRs 1a-c, Ref4**

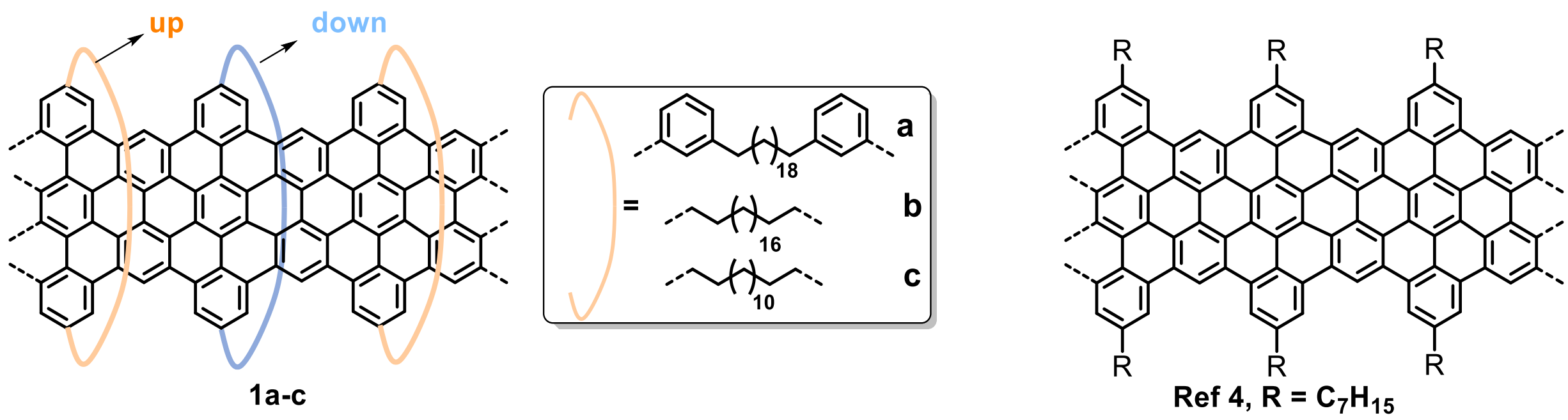

A solution of polyphenylene precursor **poly4a-c** (10 mg) in DCM (15 mL) was degassed by argon bubbling for 15 min. To the degassed solution was added a suspension of $FeCl_3$ (7 eq. for one hydrogen to be removed) in nitromethane (0.5

mL). After stirring at room temperature for 3-5 days under continuous bubbling with argon, the reaction was quenched by adding methanol to form dark purple precipitates. Filtration by suction using a membrane filter and washing intensively with methanol and THF gave the CsGNRs as dark purple powder.

2. Concentration-dependent NMR measurements

To investigate the intermolecular interaction of model compounds **2a-c** in solution state, concentration-dependent NMR study was performed at 25 °C in $C_2D_2Cl_4$ from 0.85 mmol L^{-1} to 10.2 mmol L^{-1} for **2a**, 0.98 mmol L^{-1} to 7.8 mmol L^{-1} for **2b**, 1 mmol L^{-1} to 7.6 mmol L^{-1} for **2c**, respectively (Supplementary Figure 3, 7, and 10). Evidence for the self-association behavior of the HBC backbone was observed in compounds **2a** and **2b**. The proton resonances assigned to the HBC units shifted downfield by approximately 0.01–0.02 ppm as the concentration decreased, which is attributed to the reduced shielding effect resulting from π–π stacking interactions between HBC units (Supplementary Figures 4 and 8). The slight chemical shifts indicate the presence of very weak π–π stacking interactions in both systems. Besides, the resonances corresponding to the tethered chains and the freely rotating peripheral benzene rings remained largely unchanged, suggesting their minimal involvement in the aggregation process (Supplementary Figures 5, 6, and 9). These observations are consistent with previous reports on HBC systems, which have demonstrated concentration-dependent aggregation effects[3,4].

In stark contrast, compound **2c** displayed concentration-independent behavior, with all NMR signals remaining nearly constant as the concentration was reduced from 7.6 mmol L^{-1} to 1.0 mmol L^{-1} (Supplementary Figure 11-12). This suggests that **2c** exists predominantly as monomeric species, even at relatively high concentrations. Given that only one side of the π-surface in **2c** is sterically shielded by a tethered alkyl chain, this result implies that the C_{14} chain may induce a bent geometry in the HBC backbone. Such distortion likely prevents aggregation of the unshielded π-face, thereby maintaining monodispersity.

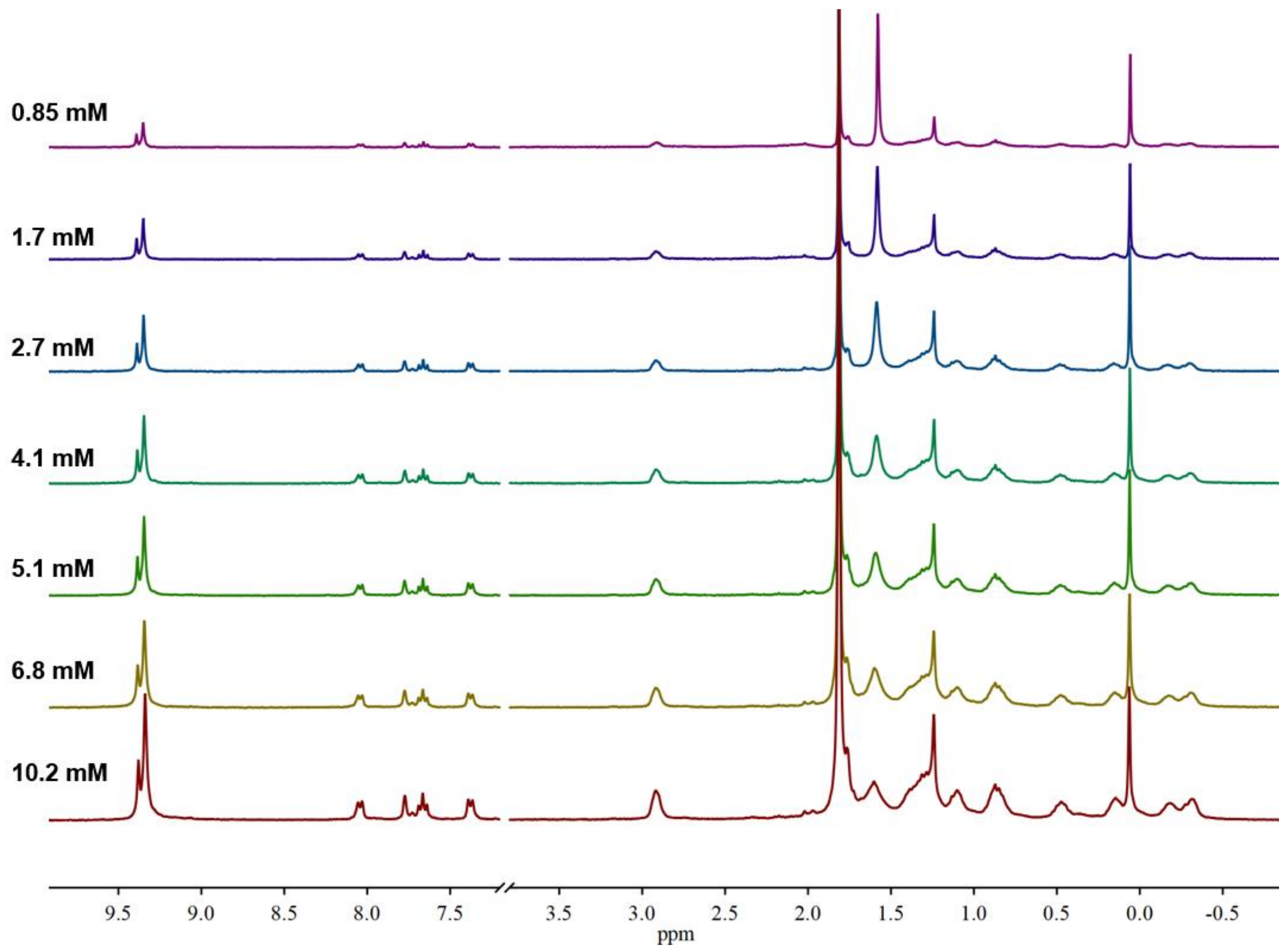

Supplementary Figure 1. Concentration-dependent ^1H NMR spectrum (300 MHz) of **2a** in $C_2D_2Cl_4$ at room temperature.

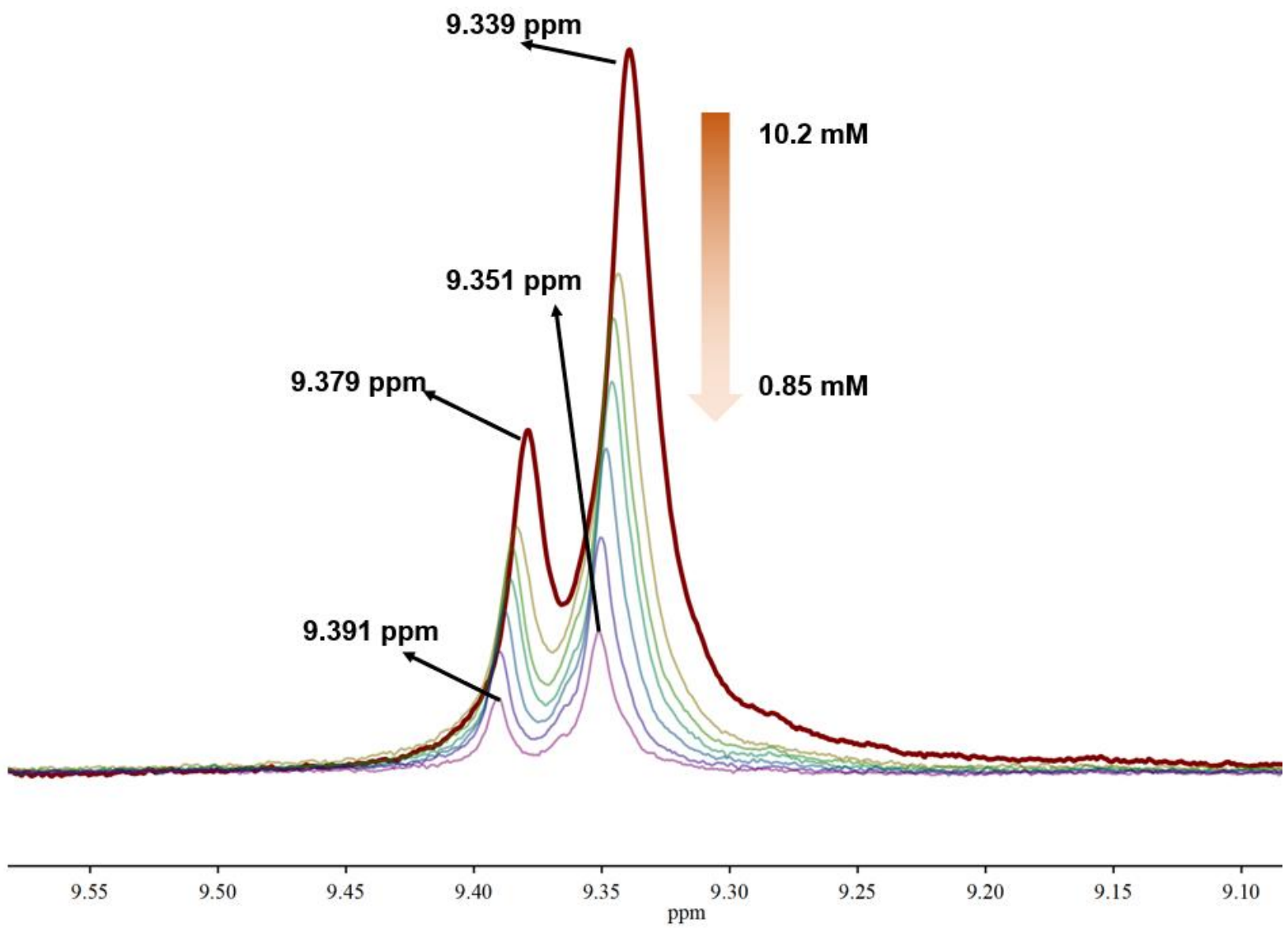

Supplementary Figure 2. Concentration-dependent ^1H NMR spectrum (300 MHz) of **2a** in the range of 9.1-9.5 ppm, record in $C_2D_2Cl_4$ at room temperature.

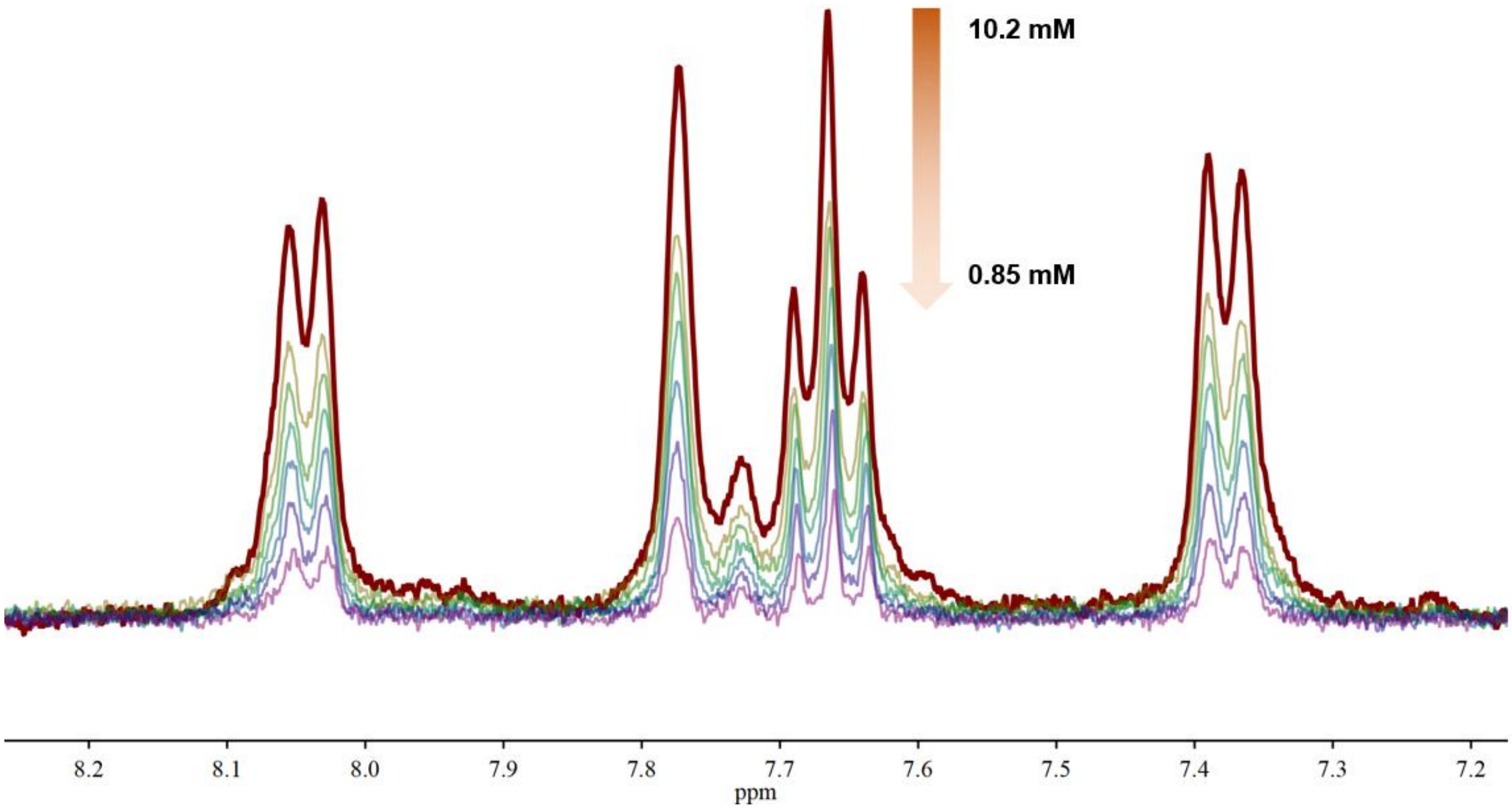

Supplementary Figure 3. Concentration-dependent ^{1}H NMR spectrum (300 MHz) of **2a** in the range of 7.2-8.2 ppm, record in $C_2D_2Cl_4$ at room temperature.

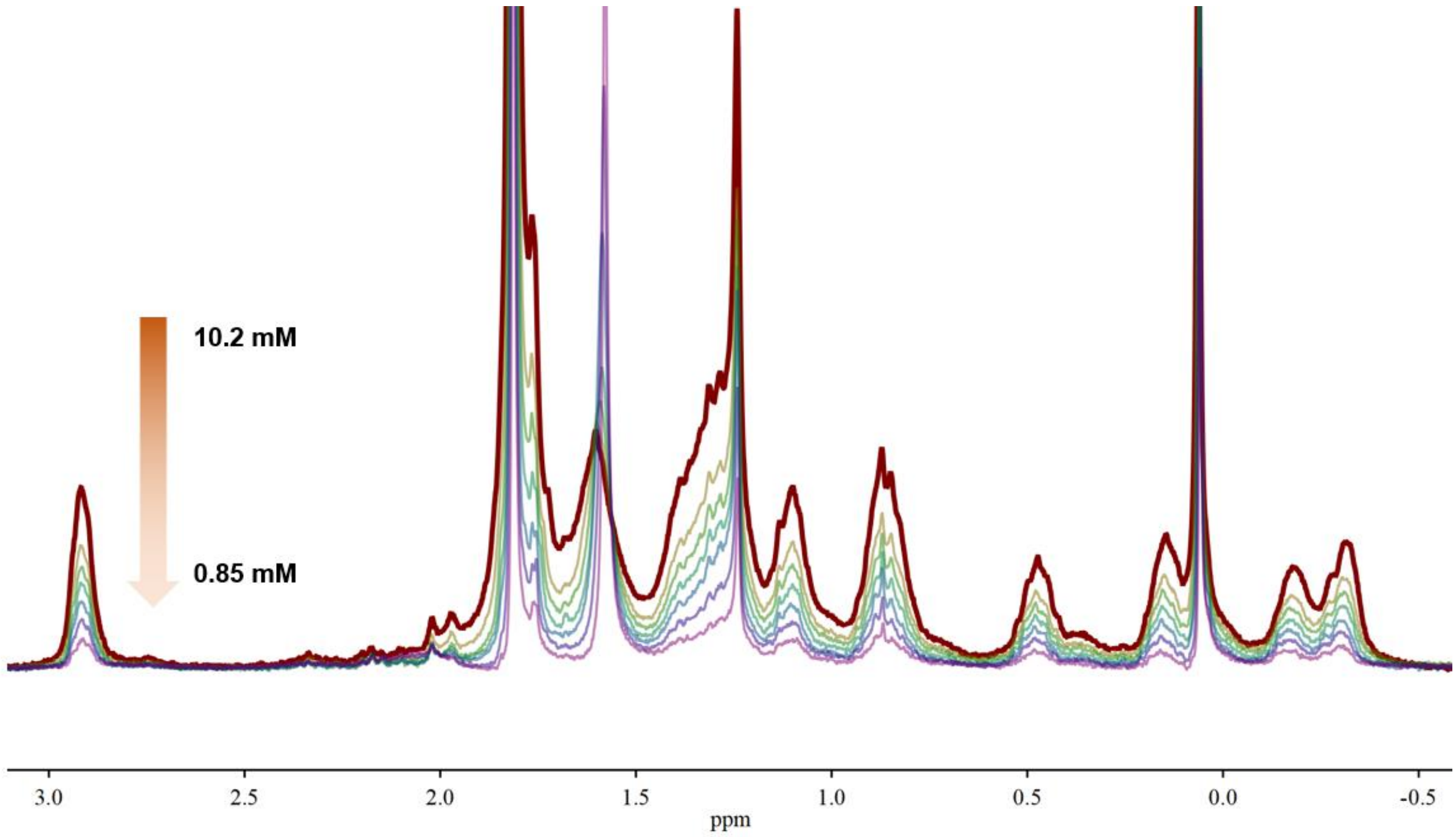

Supplementary Figure 4. Concentration-dependent ^{1}H NMR spectrum (300 MHz) of **2a** in the range of -0.5-3.0 ppm, record in $C_2D_2Cl_4$ at room temperature.

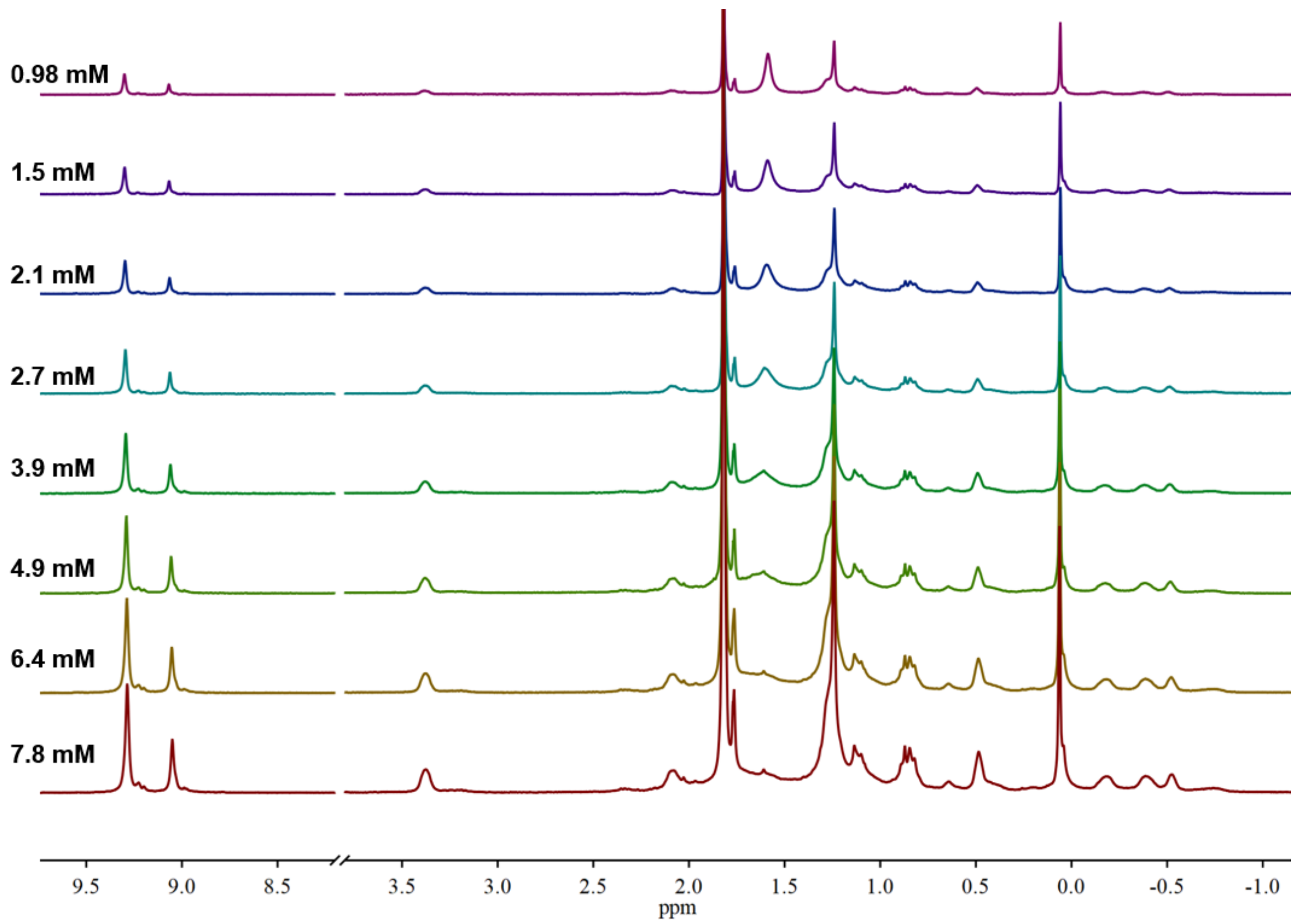

Supplementary Figure 5. Concentration-dependent ^{1}H NMR spectrum (300 MHz) of **2b** in $C_2D_2Cl_4$ at room temperature.

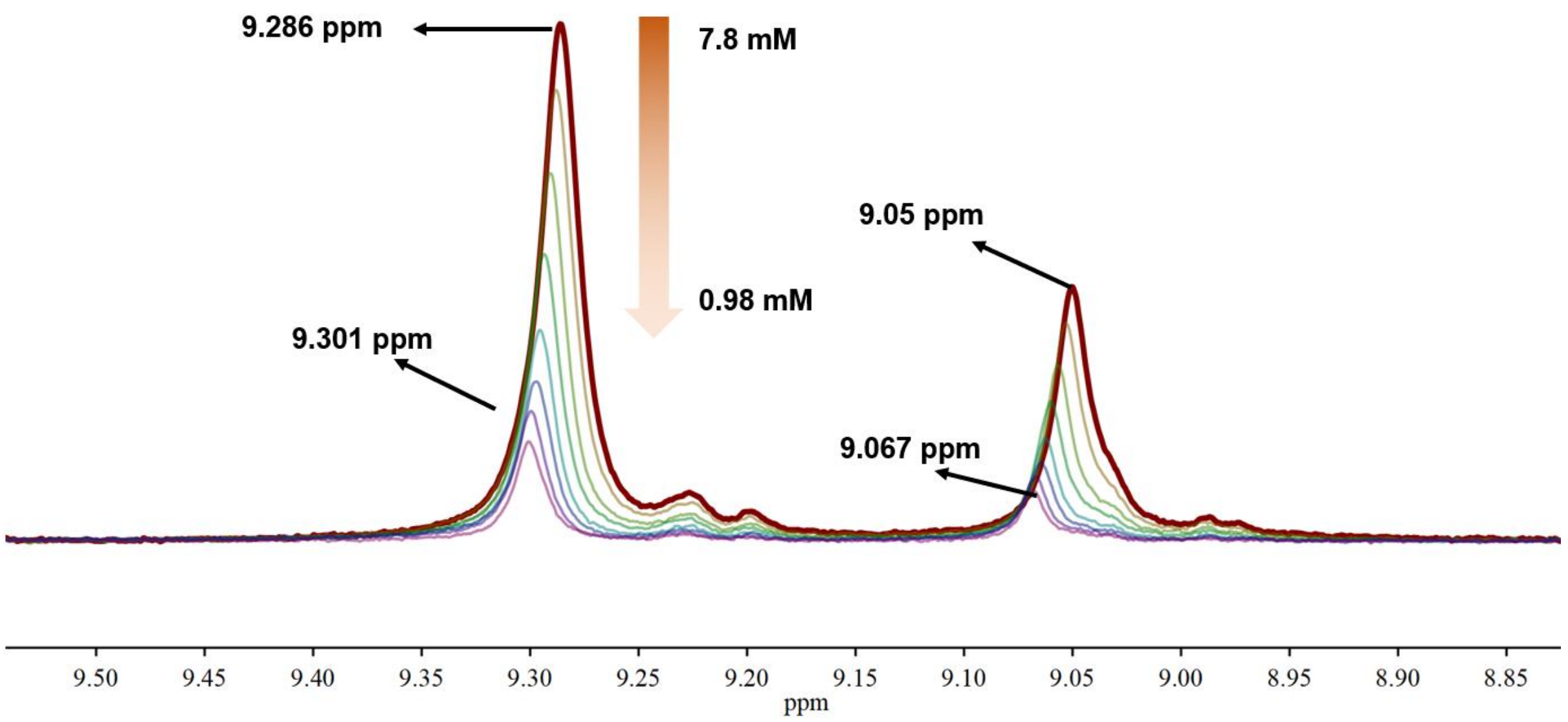

Supplementary Figure 6. Concentration-dependent ^{1}H NMR spectrum (300 MHz) of **2b** in the range of 8.9-9.5 ppm, record in $C_2D_2Cl_4$ at room temperature.

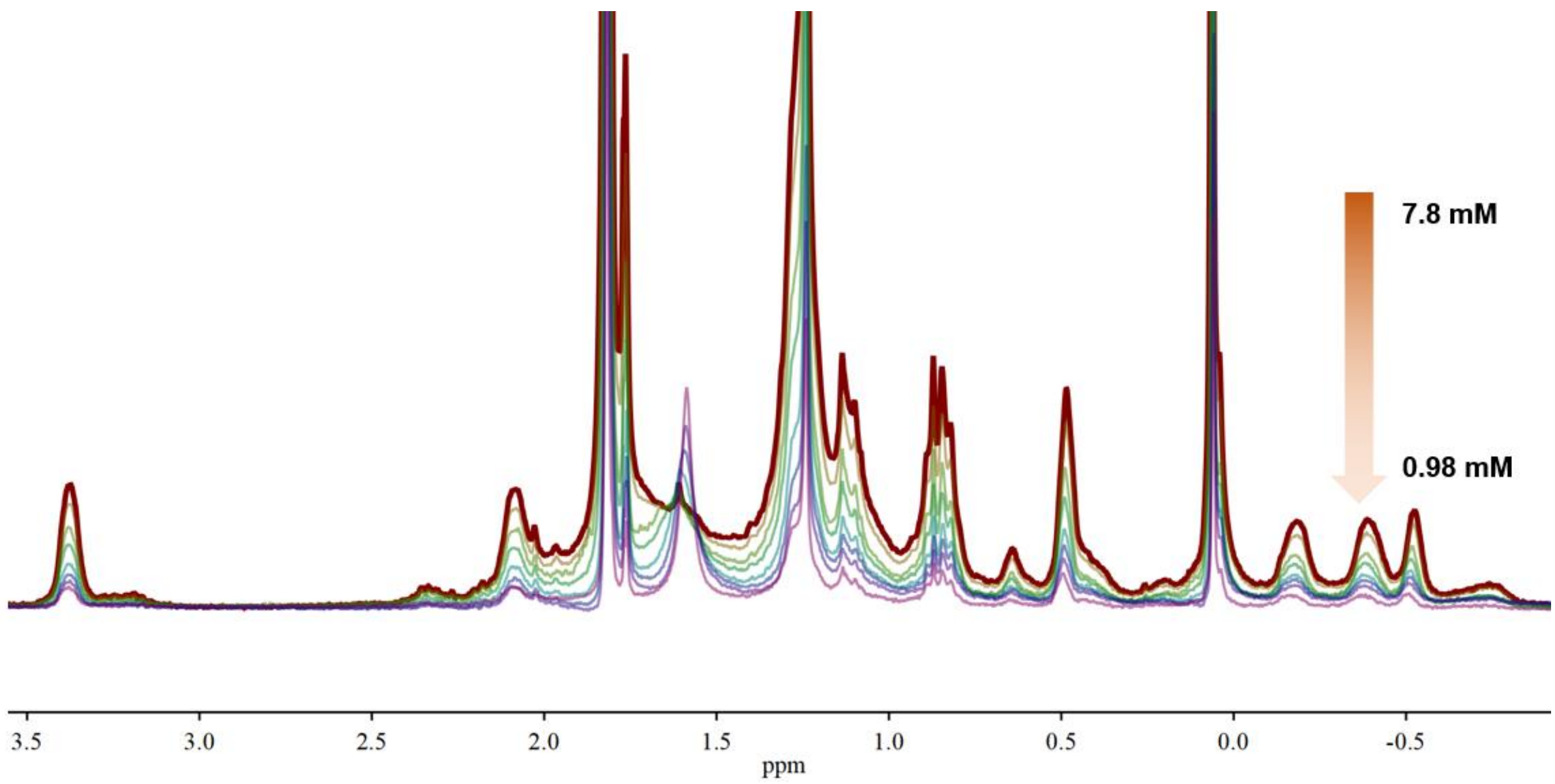

Supplementary Figure 7. Concentration-dependent ^{1}H NMR spectrum (300 MHz) of **2b** in the range of -1-3.5 ppm, recorded in $C_2D_2Cl_4$ at room temperature.

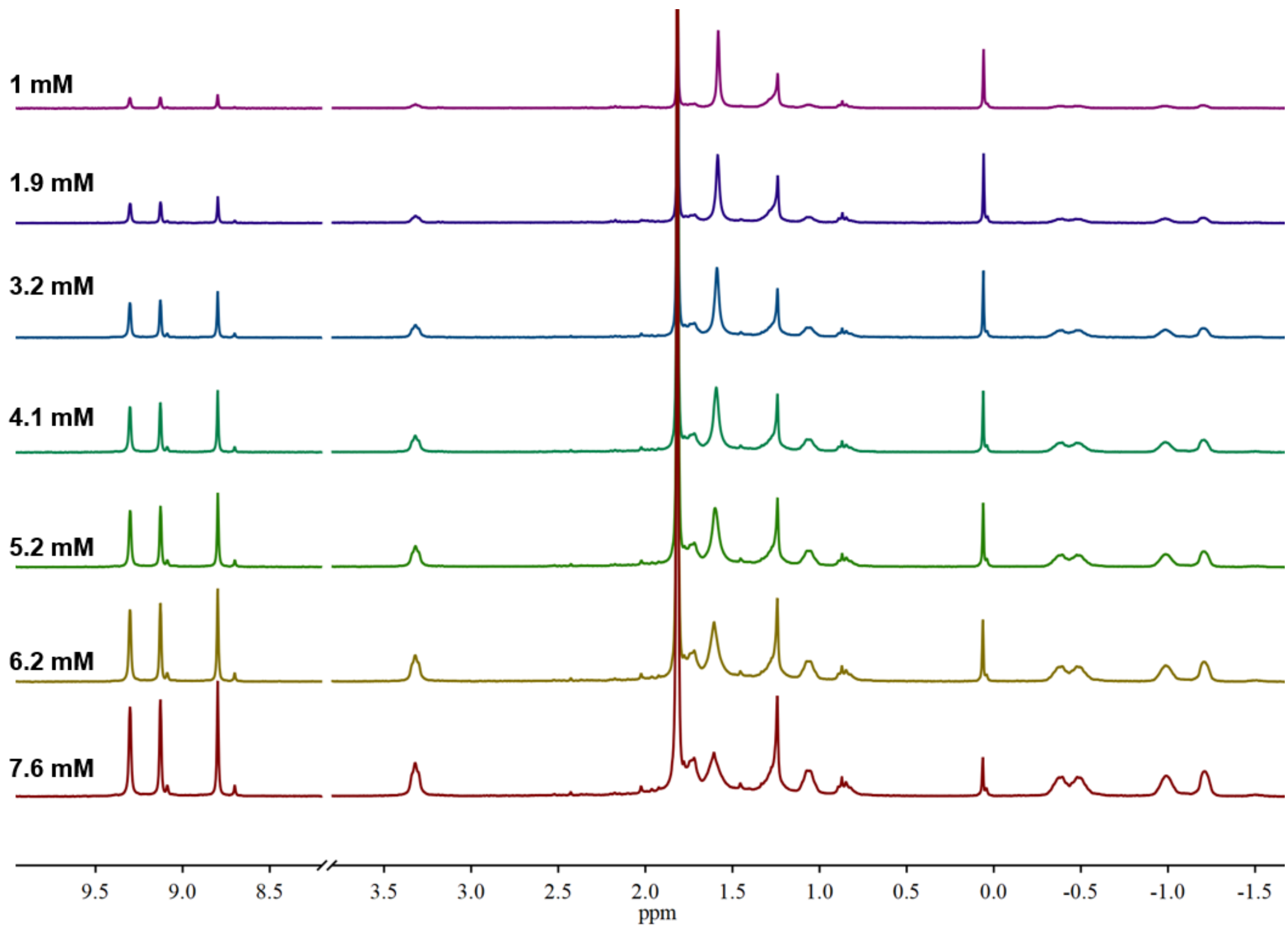

Supplementary Figure 8. Concentration-dependent ^{1}H NMR spectrum (300 MHz) of **2c** in $C_2D_2Cl_4$ at room temperature.

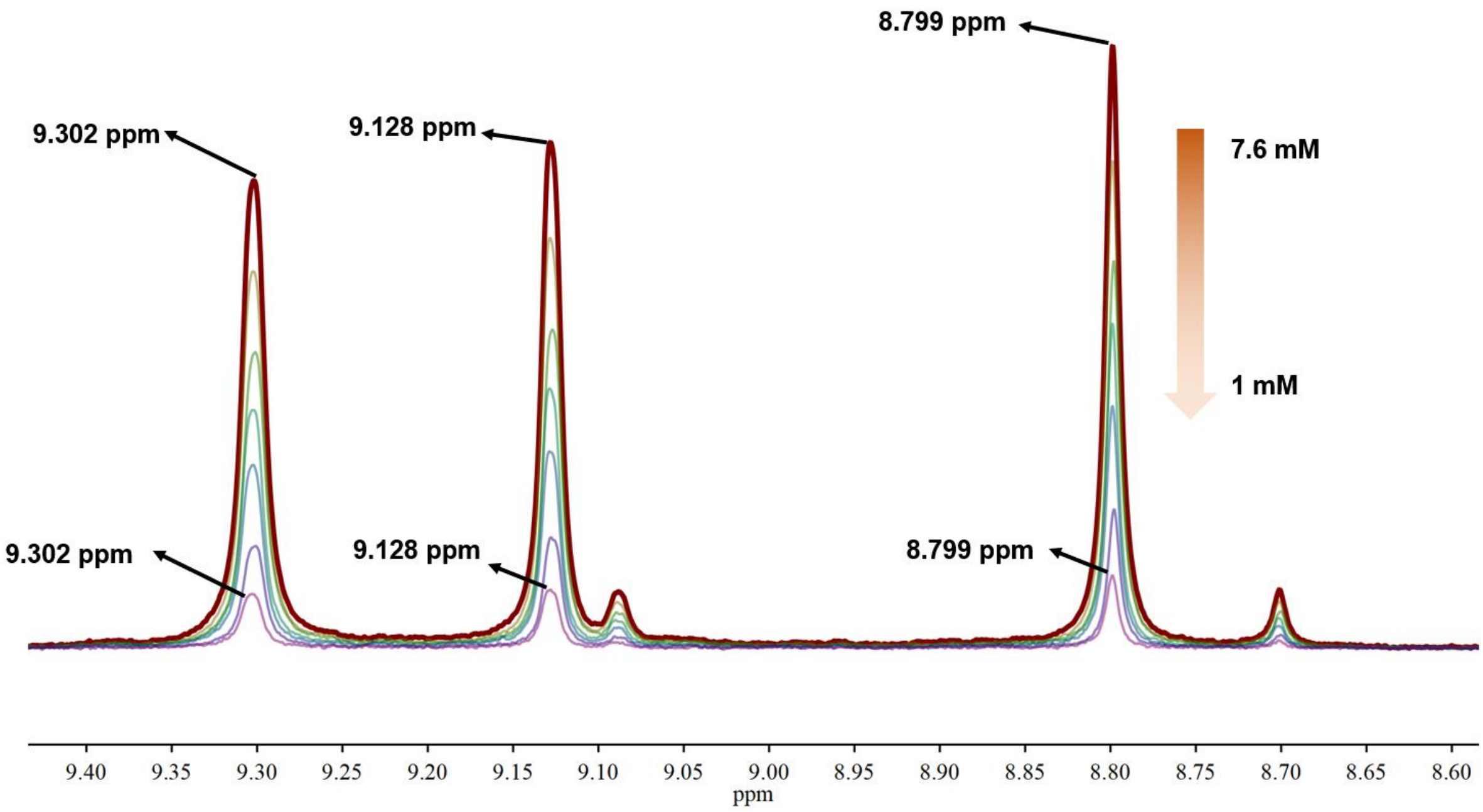

Supplementary Figure 9. Concentration-dependent ^{1}H NMR spectrum (300 MHz) of **2c** in the range of 8.6-9.4 ppm, recorded in $C_2D_2Cl_4$ at room temperature.

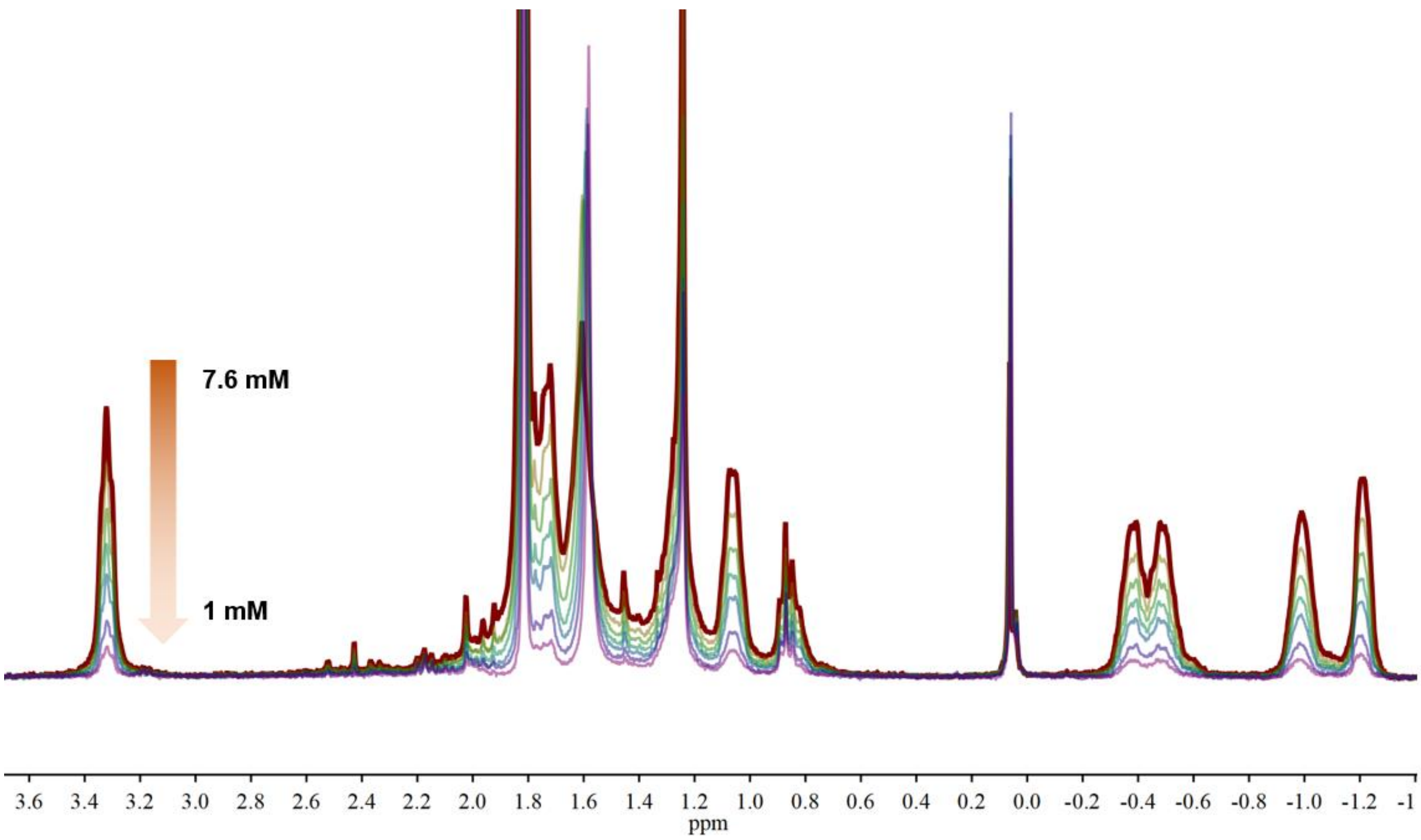

Supplementary Figure 10. Concentration-dependent ^{1}H NMR spectrum (300 MHz) of **2c** in the range of -1.4-3.6 ppm, recorded in $C_2D_2Cl_4$ at room temperature.

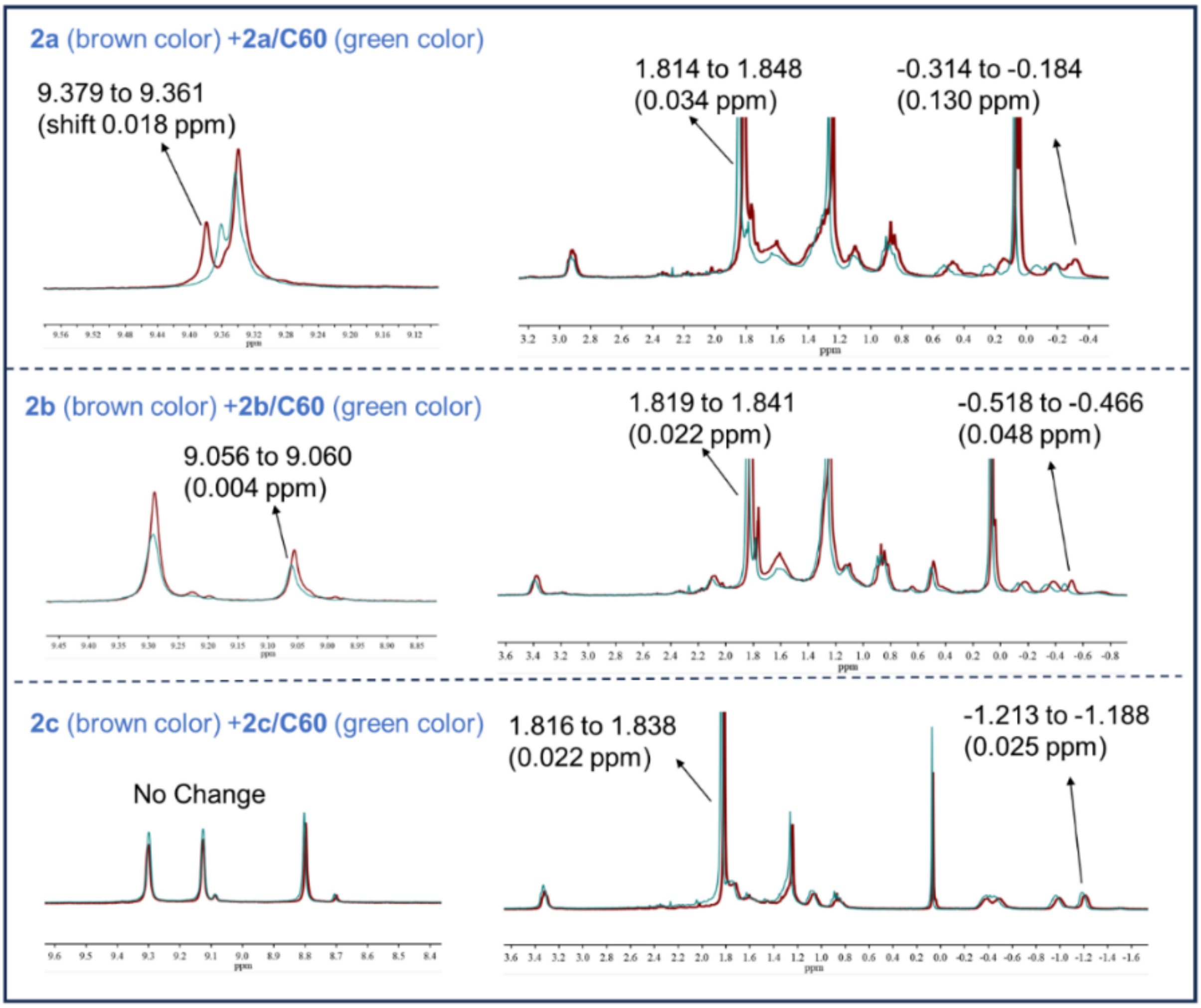

Supplementary Figure 11. Comparison of ^{1}HNMR spectra of **2a-c** and **2a-c**/C60 (1:1) in $C_2D_2Cl_4$ at room temperature.

3. UV-Vis absorption spectrum of 2a-c

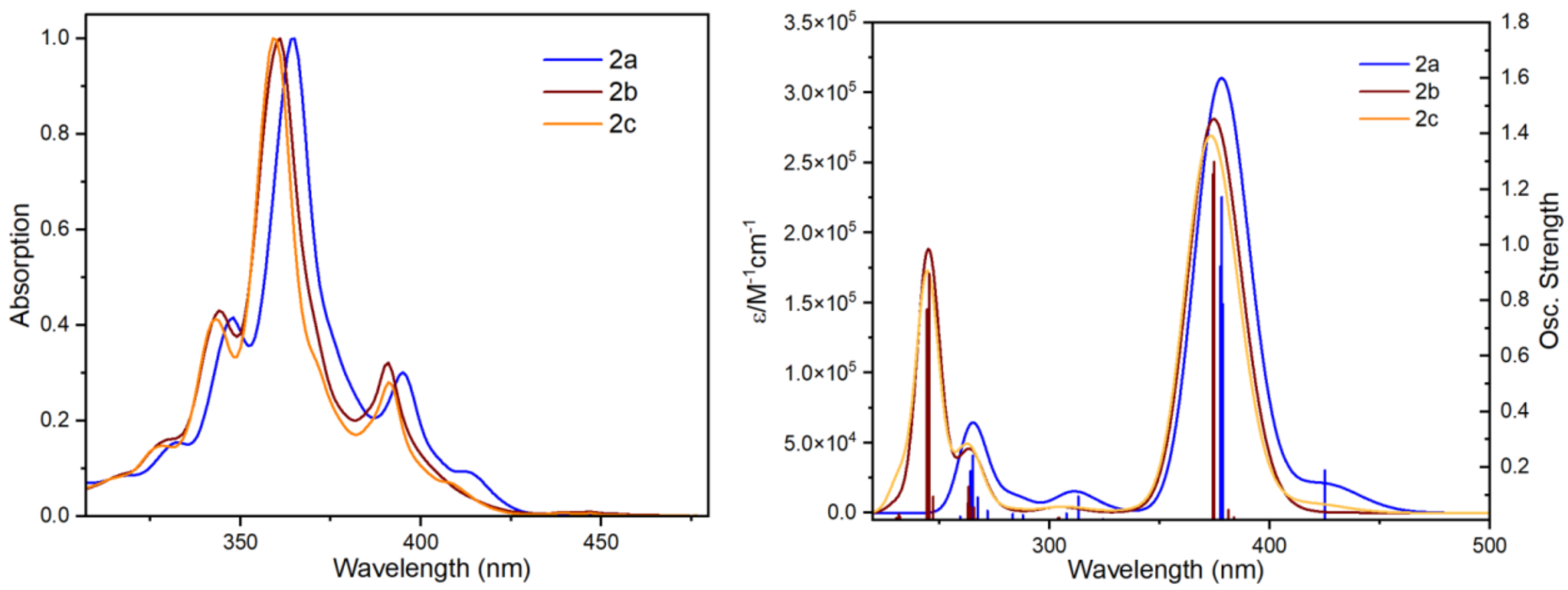

Supplementary Figure 12. UV-Vis absorption spectra of **2a-c** measured in DCM (~10^{-5} M) (left) and simulated results by DFT calculation (right).

4. X-ray structure

The Single crystal of **2a** suitable for measurements was grown in its chlorobenzene solution, and **2c** was grown by diffusing n-hexane vapor into its DCM solution. The crystals were measured on a Rigaku Oxford SuperNova Diffractometer while the temperature of the crystal was controlled by Oxford Cryostream 700. Using Olex2, all the initial structures were solved with the SHELX-XT structure solution program using the direct method and refined with the XL refinement package using Least Squares minimization. Crystallographic data were deposited in the Cambridge Crystallographic Data Centre (CCDC 2529326 for **2a** and CCDC 2447470 for **2c**). The data can be obtained free of charge from the Cambridge Crystallographic Data Centre via www.ccdc.cam.ac.uk/data_request/cif.

Supplementary Table 1. Crystal data and structure refinement for compound **2a** and **2c**.

CCDC	2529326 (**2a**)	2447470 (**2c**)
Empirical formula	$C_{90}H_{96}$	$C_{72}H_{76}$
Formula weight	1177.66	941.32
Temperature/K	100(1)	100.01(10)
Crystal system	triclinic	monoclinic
Space group	P-1	C2/c
a/Å	11.8996(12)	27.2802(5)
b/Å	17.2537(8)	20.3840(4)
c/Å	17.4306(12)	20.0183(5)
α/°	100.593(5)	90
β/°	96.529(7)	102.813(2)
γ/°	94.848(6)	90

Volume/Å^3	3474.5(5)	10854.6(4)
Z	2	8
ρ_{calc}g/cm^3	1.126	1.152
μ/mm^{-1}	0.469	0.481
F(000)	1272	4064
Crystal size/mm^3	0.5 × 0.5 × 0.1	0.5 × 0.1 × 0.1
Radiation	CuKα (λ = 1.54184)	CuKα (λ = 1.54184)
2Θ range for data collection/°	6.626 to 125.654	5.462 to 154.224
Index ranges	-13 ≤ h ≤ 13, -18 ≤ k ≤ 19, -20 ≤ l ≤ 18	-34 ≤ h ≤ 34, -19 ≤ k ≤ 25, -25 ≤ l ≤ 24
Reflections collected	31003	39295
Independent reflections	10770 [R_{int} = 0.0728, R_{sigma} = 0.0870]	10938 [R_{int} = 0.0638, R_{sigma} = 0.0601]
Data/restraints/parameters	10770/0/823	10938/570/692
Goodness-of-fit on F^2	1.005	1.016
Final R indexes [I>=2σ (I)]	R_1 = 0.0905, wR_2 = 0.2501	R_1 = 0.0637, wR_2 = 0.1675
Final R indexes [all data]	R_1 = 0.1440, wR_2 = 0.2933	R_1 = 0.0962, wR_2 = 0.1892
Largest diff. peak/hole / e Å^{-3}	0.42/-0.26	0.34/-0.24

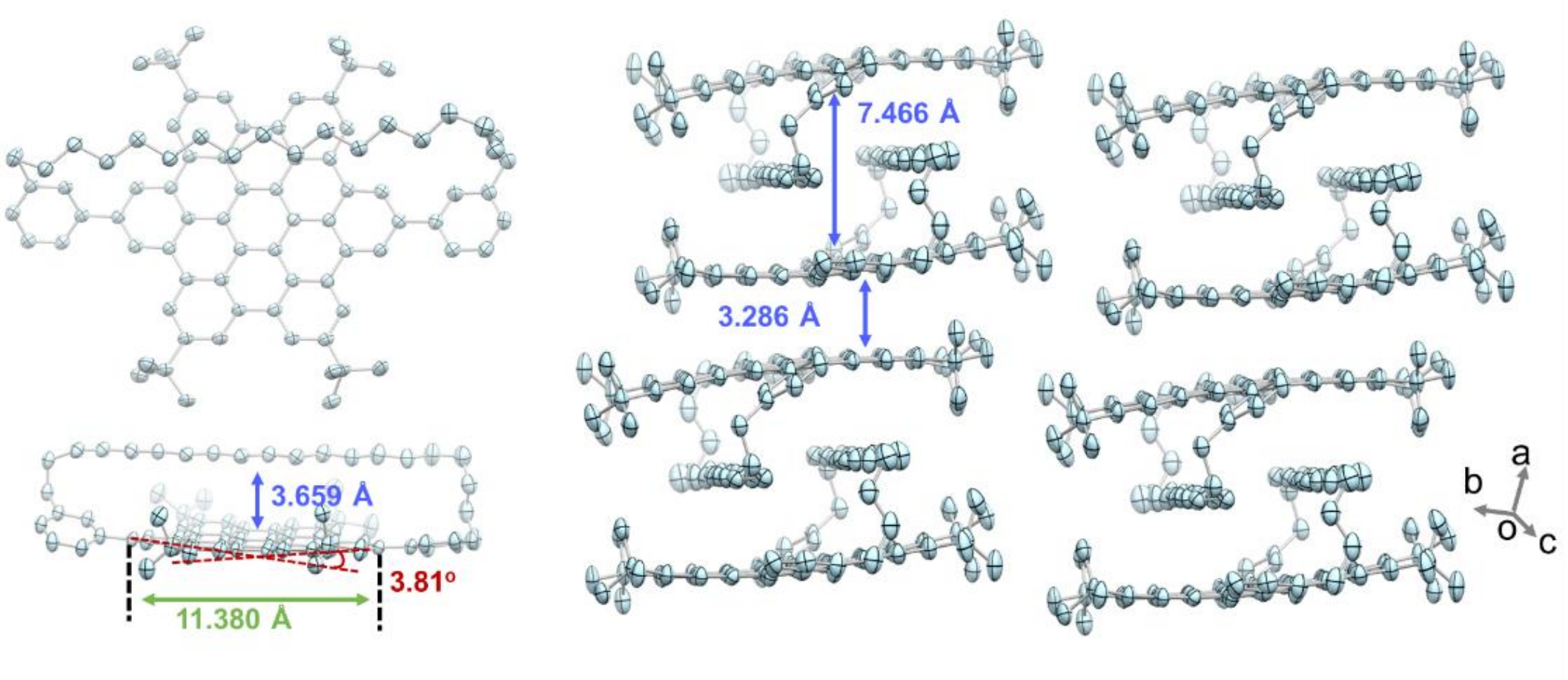

Supplementary Figure 13. Single crystal structure analysis of **2a**.

5. Polymers characterization

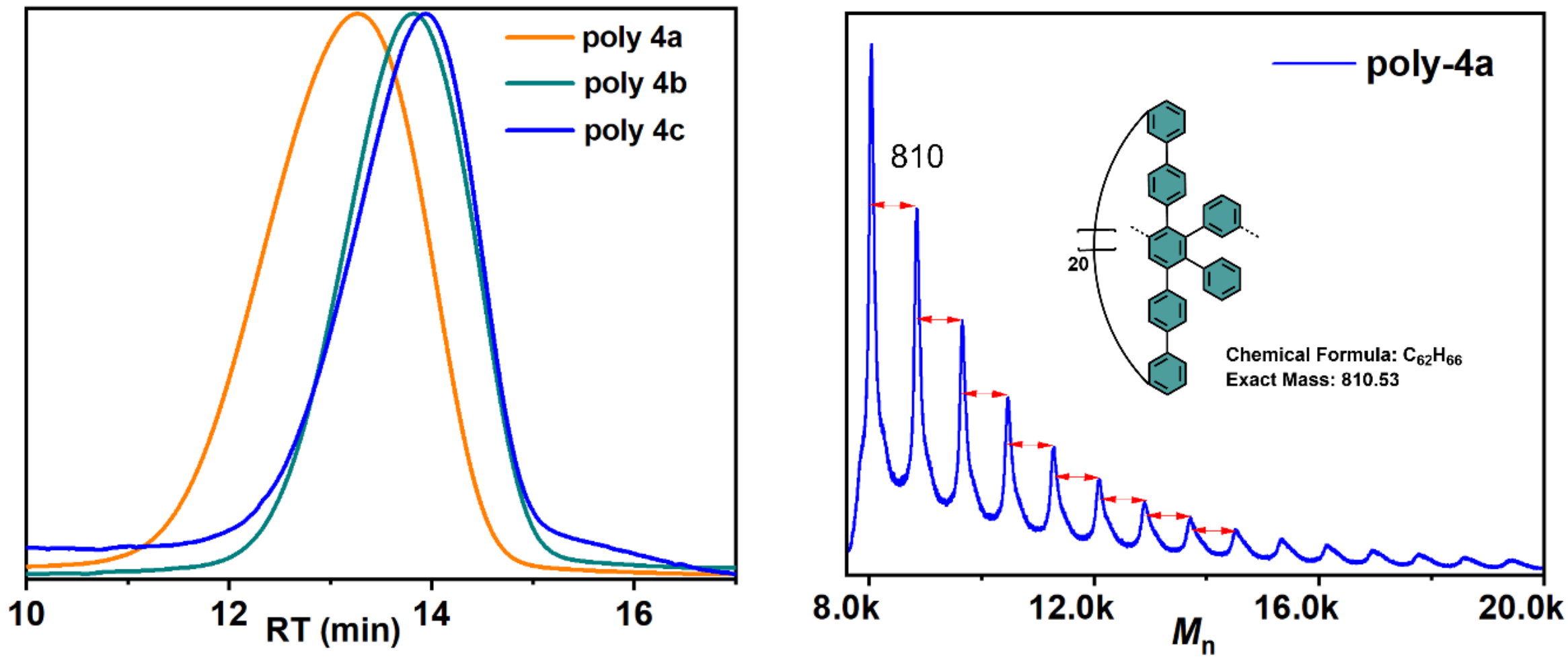

Supplementary Figure 14. GPC curve of **poly 4a-c** (left), and the MALDI-TOF measurement of **poly-4a**.

The analysis results of analytical size exclusion chromatography (SEC) calibrated by polystyrene (PS) standards revealed a close number-average molar mass (M_n) of 75 kDa for **10a**, 50 kDa for **10b**, and 48 kDa for **10c**, with a low polydispersity index (*Đ*) range from 1.2 to 1.3 for the first fraction of these polymers.

6. Solid-state NMR analysis

Since the investigated GNRs and their respective precursors consist exclusively of hydrogen and carbon, ^{1}H and ^{13}C solid-state MAS NMR are useful tools to investigate their composition. Due to the strong expected direct dipolar couplings, either homonuclear between different ^{1}H spins or heteronuclear between ^{13}C and ^{1}H, high MAS frequencies were used to achieve reasonable resolution although the small sample volume limits the achievable signal-to-noise ratio from the ^{13}C experiments. For ^{1}H experiments, onepulse spectra suffer from an intense ^{1}H probe background that might obscure the spectra. To overcome this, Hahn echo spectra were acquired additionally, where the broad background signal is almost completely suppressed. These spectra, however, might give skewed intensity ratios because of the T_2 weighing of the signals.

The resulting ^{1}H MAS NMR spectra for the **poly 4a-c** and the **CsGNRs 1a-c** are shown in Supplementary Figure 15, 16 and 17, respectively. Because of their structural similarity, all spectra of the precursors and the GNRs look qualitatively identical. For the precursors, good resolution can be achieved between the signal of the aliphatic protons of the alkyl chains and the signal of the aromatic protons attached to the ribbon's backbone. Signal maxima are located at about 1.0 ppm to 1.1 ppm and 6.8 ppm to 6.9 ppm, respectively. For the corresponding GNRs, the resolution is greatly reduced due to the much broader linewidths. The broader lines are attributed to the higher effective homonuclear ^{1}H-^{1}H dipolar couplings that result from the reduced mobility of the GNR backbone. Nevertheless, spectral deconvolution can be achieved. From these, signal ratios of aliphatic to aromatic protons can be obtained and compared to the expected ratios from the structure. The results are summarized in Supplementary Table 2. First, it is noticed that the ratios obtained from the onepulse and Hahn echo spectra correlate well and only differ in a few percent, which is well within the expected error of a few percent that results from some ambiguity in the deconvolution. From this it can be concluded that T_2 effects do not play a significant role. Furthermore, the

experimental ratios obtained match the expected ratios well, usually within a few percent. For example, the expected signal intensity ratio of aliphatic to aromatic protons for **poly 4a** is 60.6 : 39.4, while the experimental ratios are 59.9 : 40.1 and 61.5 : 38.5 from the onepulse and Hahn echo experiment, respectively. The greatest deviation is observed for **CsGNR 1c**, where the experimental ratios are 78.8 : 21.2 and 74.6 : 25.4 while the expected one is 82.4 : 17.6. As expected, the signal ratio of aliphatic to aromatic protons increases upon graphitization due to the loss of aromatic hydrogen, which is qualitatively and quantitatively reflected in the experimental ratios, which match well within the expected error.

$^{13}C\{^{1}H\}$ spectra were also acquired to further confirm the results given above. Usually, ^{13}C T_1 relaxation times are much longer (tens of seconds to minutes) than those of ^{1}H (a few seconds). This and the low natural abundance of ^{13}C hinder the acquisition of quantitative spectra in a reasonable amount of time. While this holds for the precursors, in GNRs the T_1 relaxation times are often greatly reduced so that quantitative results can be expected even for short recycle delays.[5] To also observe the effect of T_1 relaxation to some extent, spectra with different recycle delays of 3.5 s and 7 s were acquired. The spectra for the **poly 4a-c** and the **CsGNRs 1a-c** are shown in Supplementary Figures 18, 19, and 20, respectively.

The $^{13}C\{^{1}H\}$ spectra for **poly 4a-c** as well as **CsGNRs 1a-c** also show a signal for the aliphatic carbon spins (maxima at around 31.0 to 31.5 ppm for the precursors and 29.0 ppm to 29.5 ppm for the GNRs) and a more complex signal (mainly two maxima at around 127.5 ppm to 128.5 ppm and 140.0 ppm to 141.6 ppm for **poly 4a-c** and three maxima around 122.5 ppm to 123.5 ppm, 128.0 ppm to 129.5 ppm and 139.4 ppm to 140.5 ppm for the **CsGNRs 1a-c**) for the aromatic carbon spins which reflect the inner and edge carbon atoms. Again, signal linewidths increase significantly upon graphitization because of the lower mobility resulting in stronger effective ^{13}C-^{1}H heteronuclear dipolar couplings. The obtained aliphatic to aromatic carbon signal intensity ratios from the spectra with a 3.5 s recycle delay for aliphatic to aromatic carbon signals are skewed greatly towards the

aliphatic fraction for **poly 4a-c**. This is due to the incomplete T_1 relaxation, especially of the aromatic carbon spins that appear to have substantially longer T_1 relaxation times than the aliphatic carbon spins. This is evident from the ratios from the spectra with the 7 s recycle delay, which are already closer to the expected values, although the aliphatic fraction is still overpronounced. For the **CsGNRs**, however, the ratios for the 3.5 s and 7.0 s spectra are already very close to each other and to the expected ones. This indicates that a recycle delay of 3.5 s or at least 7.0 s is enough to obtain quantitative results.

The connectivity of the proton spin network can be analyzed using ^{1}H-^{1}H 2D double-quantum/single-quantum (DQ/SQ) correlation spectroscopy. Double quanta are excited via direct dipolar coupling of ^{1}H spins directly through space. Two spectra for each sample with different excitation times are shown in Supplementary Figure 21. The same trends as in the 1D spectra can be observed regarding the linewidths. Even for short DQ excitation times of 4 rotor periods (64 μs), all auto- and cross-correlations are visible reflecting the strongly coupled network. For longer excitation times of 12 rotor periods (192 μs) signal intensity decreases due to T_2 relaxation. Especially for the GNRs, the auto-correlation signals appear to be stretched along the DQ/SQ auto-correlation line. This hints towards a distribution of chemical shifts as can be expected for the packed and rather immobile nanoribbons offering a broad range of slightly different chemical environments for the protons.

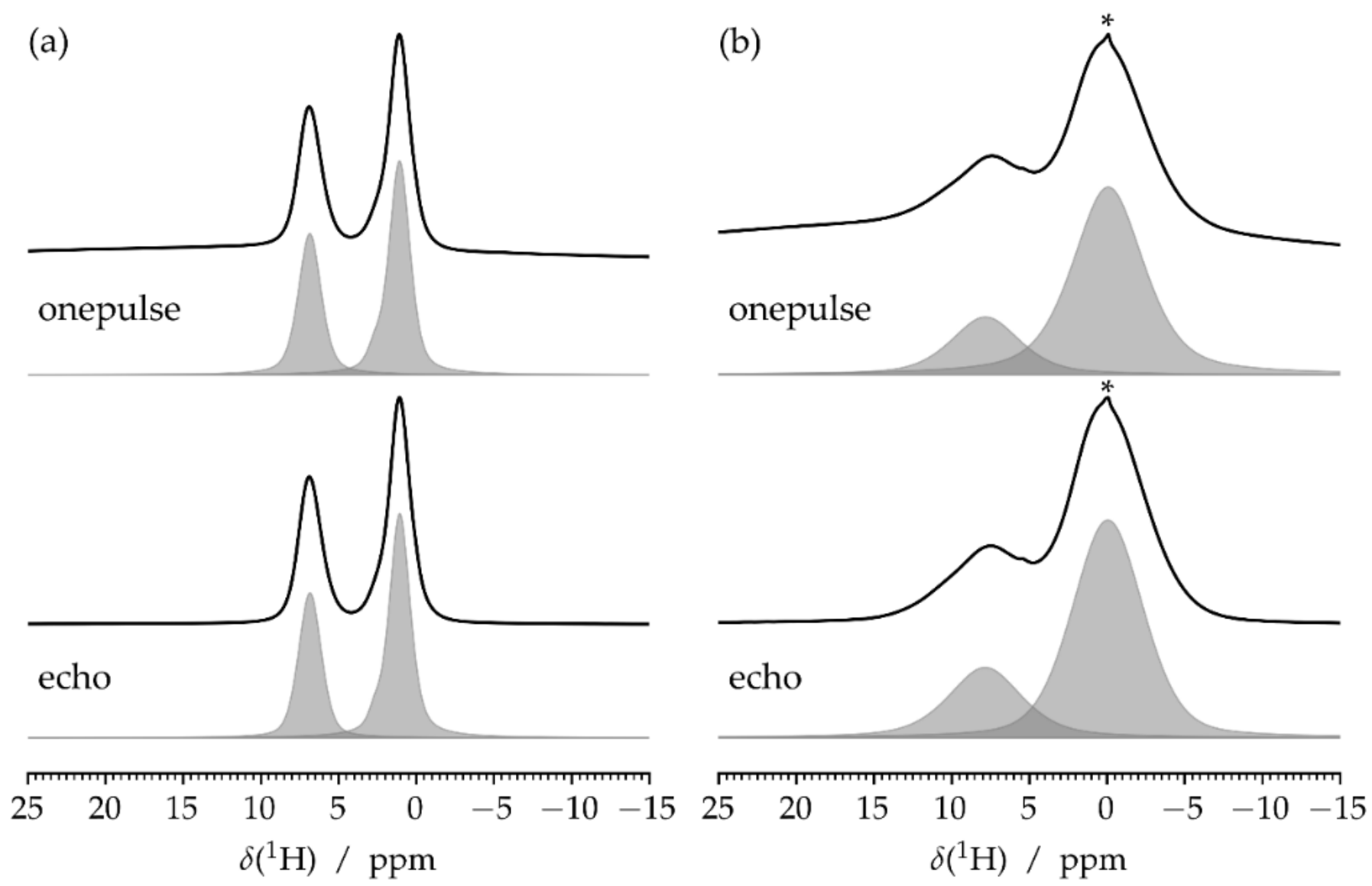

Supplementary Figure 15. ^{1}H solid-state onepulse and Hahn echo MAS NMR spectra of (a) **poly 4a** and (b) **CsGNR 1a** spinning at 62.5 kHz MAS and B_0 = 11.75 T and ambient temperature. Spinning sidebands are not shown. Each spectrum was acquired with 16 scans and a 64 s recycle delay. The fitted signals from the deconvolution for the aromatic and aliphatic signal regions are shown in gray below the spectra. The asterisks (*) indicate the remaining solvent signal.

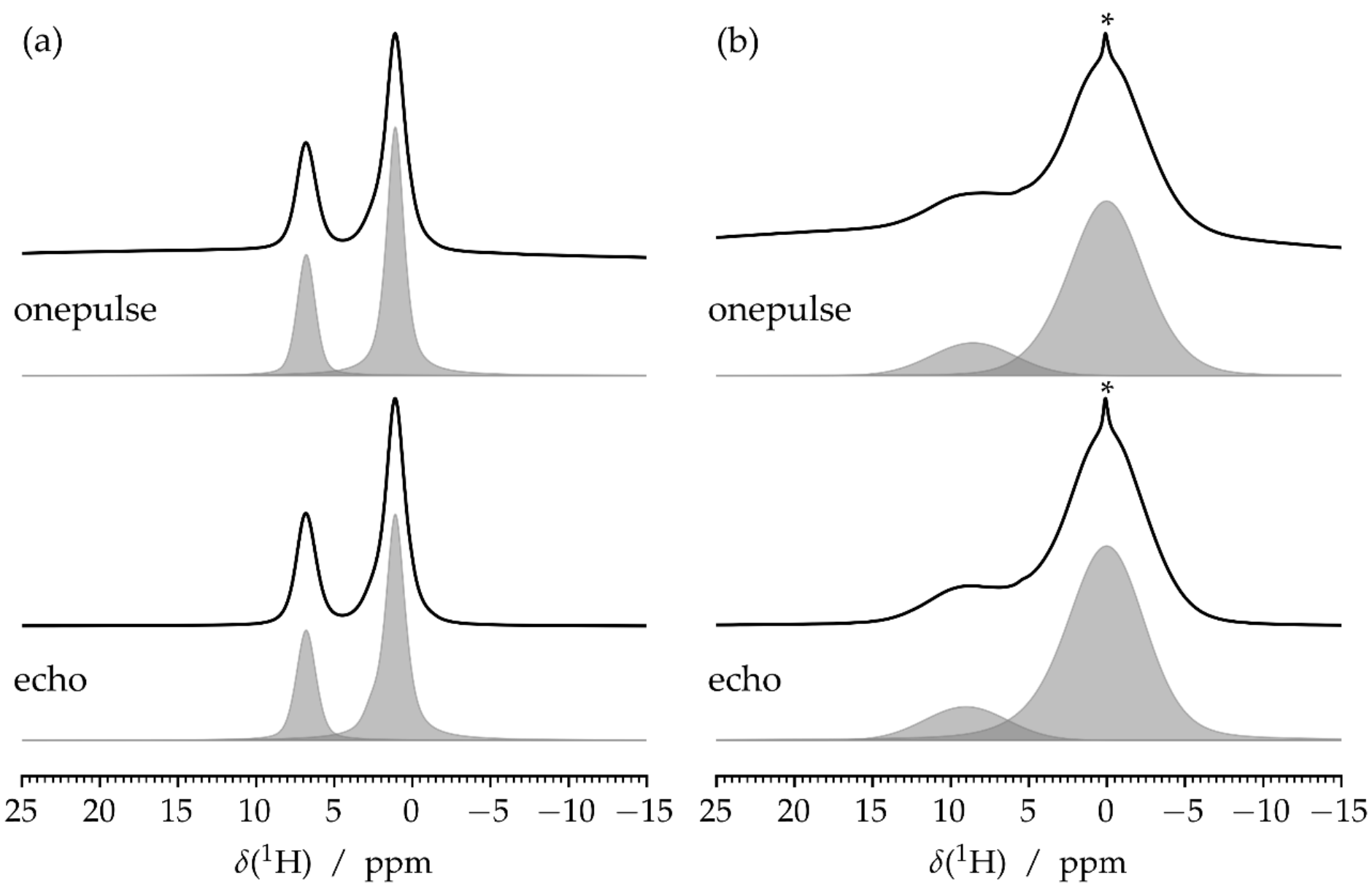

Supplementary Figure 16. ^{1}H solid-state onepulse and Hahn echo MAS NMR spectra of (a) **poly 4b** and (b) **CsGNR 1b** spinning at 62.5 kHz MAS and B_0 = 11.75 T and ambient temperature. Spinning sidebands are not shown. Each

spectrum was acquired with 16 scans and a 64 s recycle delay. The fitted signals from the deconvolution for the aromatic and aliphatic signal regions are shown in gray below the spectra. The asterisks (*) indicate the remaining solvent signal.

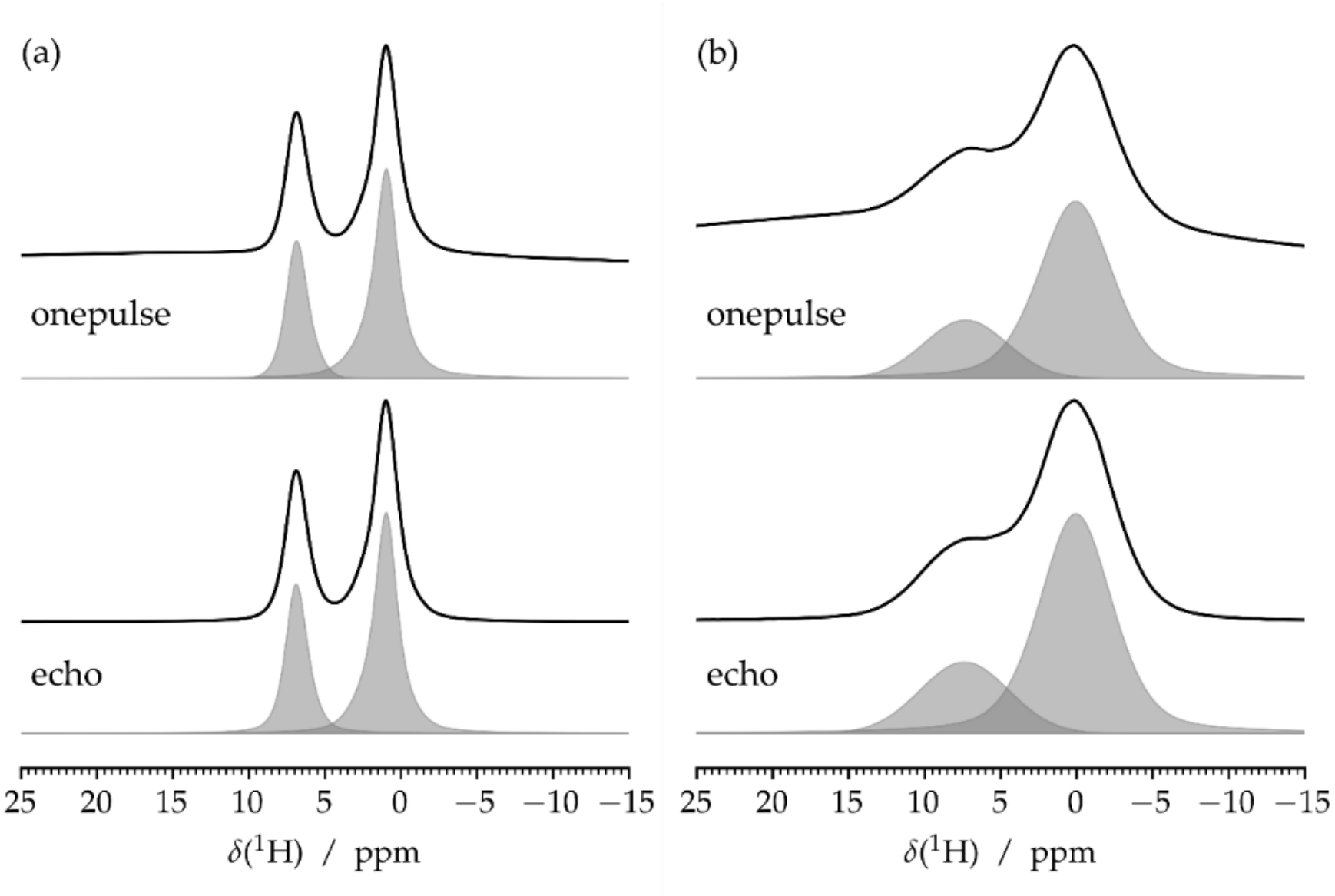

Supplementary Figure 17. ^{1}H solid-state onpulse and Hahn echo MAS NMR spectra of (a) **poly 4c** and (b) **CsGNR 1c** spinning at 62.5 kHz MAS and $B_0 = 11.75$ T and ambient temperature. Spinning sidebands are not shown. Each spectrum was acquired with 16 scans and a 64 s recycle delay. The fitted signals from the deconvolution for the aromatic and aliphatic signal regions are shown in gray below the spectra.

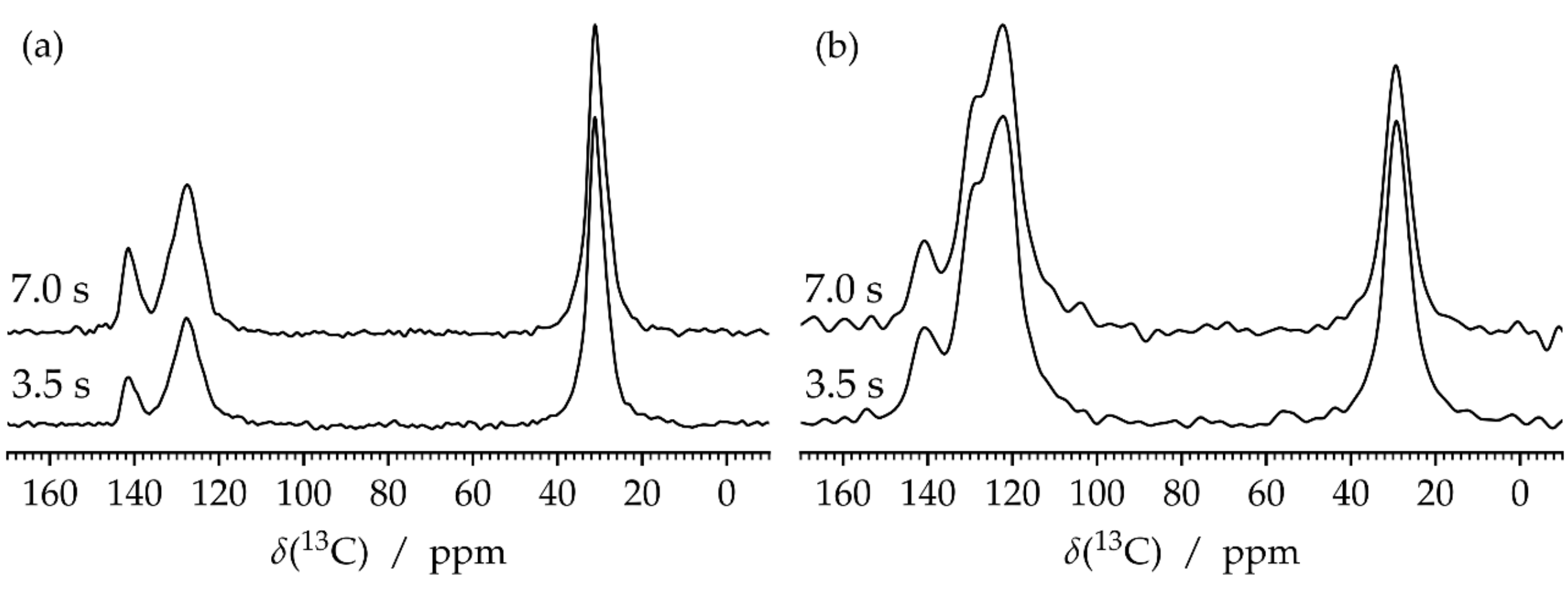

Supplementary Figure 18. $^{13}C\{^{1}H\}$ solid-state MAS NMR spectra of (a) **poly 4a** and (b) **CsGNR 1a** spinning at 62.5 kHz MAS and B_0 = 11.75 T and ambient temperature. A recycle delay of 3.5 s or 7.0 s was used. During acquisition, 28 kHz low-power ^{1}H CW decoupling was applied.

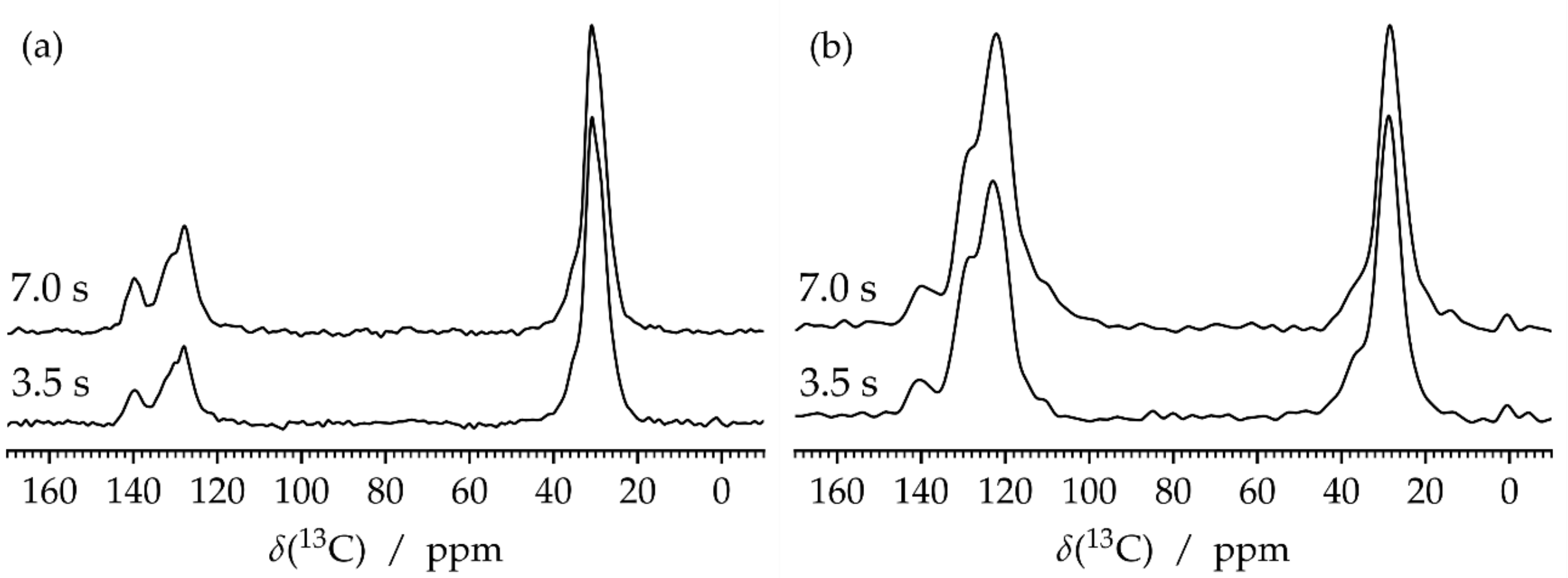

Supplementary Figure 19. $^{13}C\{^{1}H\}$ solid-state MAS NMR spectra of (a) **poly 4b** and (b) **CsGNR 1b** spinning at 62.5 kHz MAS and B_0 = 11.75 T and ambient temperature. A recycle delay of 3.5 s or 7.0 s was used. During acquisition, 28 kHz low-power ^{1}H CW decoupling was applied.

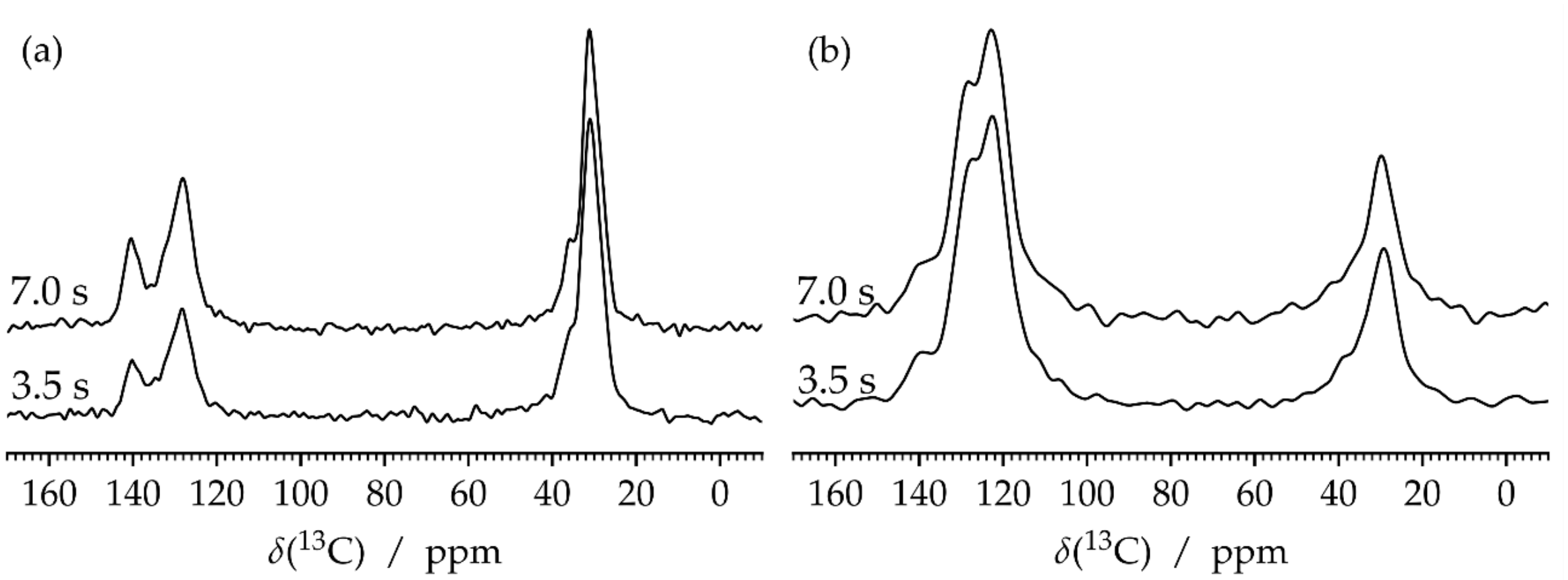

Supplementary Figure 20. $^{13}C\{^{1}H\}$ solid-state MAS NMR spectra of (a) **poly 4c** and (b) **CsGNR 1c** spinning at 62.5 kHz MAS and B_0 = 11.75 T and ambient temperature. A recycle delay of 3.5 s or 7.0 s was used. During acquisition, 28 kHz low-power ^{1}H CW decoupling was applied.

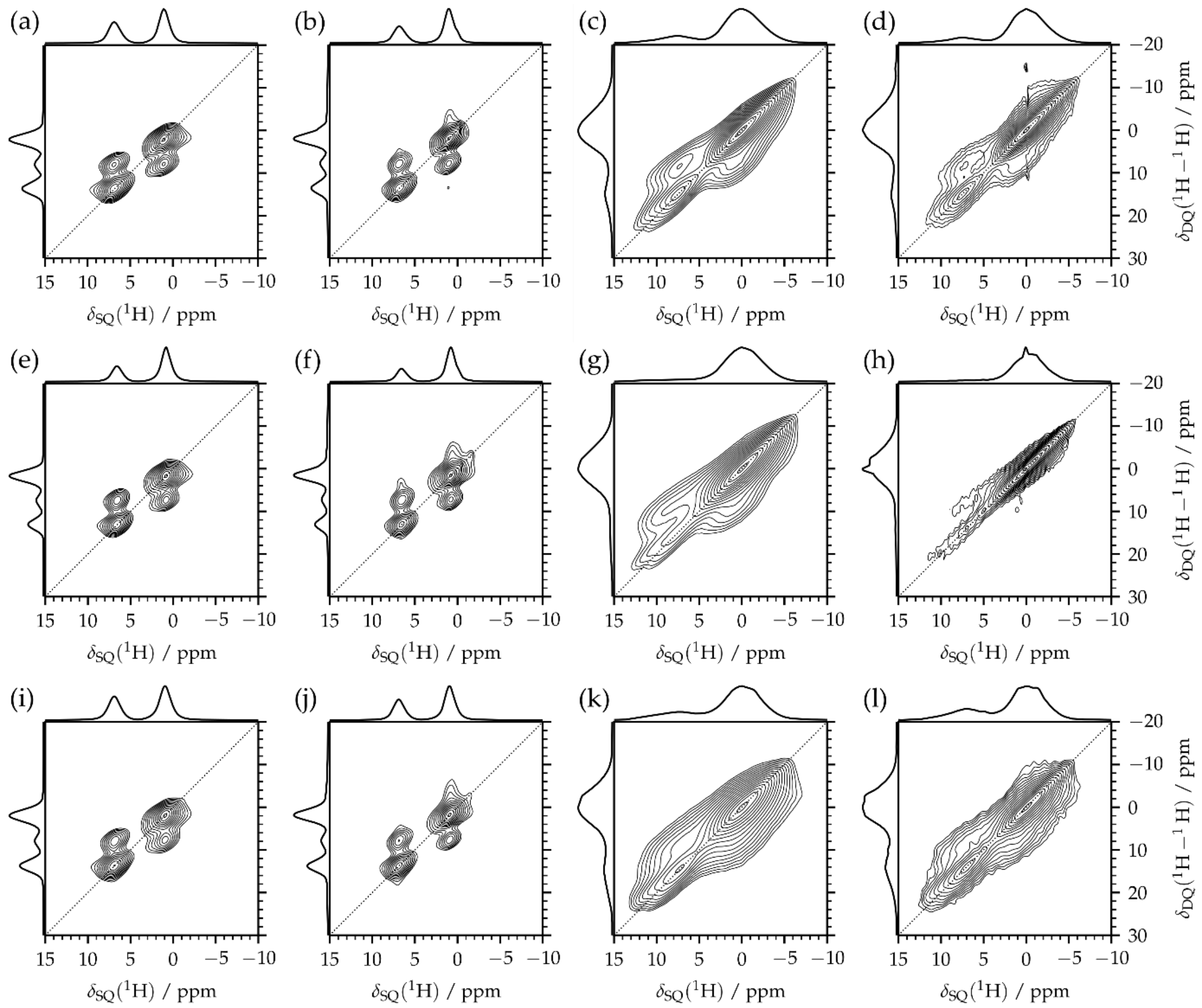

Supplementary Figure 21. ^{1}H-^{1}H 2D single-quantum/double-quantum MAS NMR correlation spectra of (a, b) **poly 4a** (c, d) **CsGNR 1a**, (e, f) **poly 4b**, (g, h) **CsGNR 1b**, (i, j) **poly 4c** and (k, l) **CsGNR 1c** at 65.0 kHz MAS and B_0 = 11.75 T at ambient temperature. The BaBaXY16 scheme was used for DQ excitation and reconversion with (a,c,e,g,i,k) 4 and (b,d,f,h,j,l) 12 rotor periods of excitation time.

Supplementary Table 2. Ratios of the aliphatic to aromatic signal intensities of the ^{1}H and $^{13}C\{^{1}H\}$ spectra and the expected ratios from the structures. Ratios are normalized such that the sum of both parts adds to 100.

**sample**	**^{1}H integral ratios**			**^{13}C integral ratios**		
	**expected**	**onepulse**	**echo**	**expected**	**3.5 s**	**7 s**
**poly 4a**	60.6 : 39.4	59.9: 40.1	61.5 : 38.5	32.3 : 67.7	60.1 : 39.9	50.7 : 49.3
**CsGNR 1a**	74.1 : 25.9	77.0 : 23.0	73.1 : 26.9	32.3 : 67.7	35.4 : 64.6	32:7 : 67.3
**poly 4b**	69.0 : 31.0	70.1 : 29.9	70.6 : 29.4	40.0 : 60.0	73.4 : 26.6	64.9 : 35.1
**CsGNR 1b**	87.0 : 13.0	83.7 : 16.3	85.9 : 14.1	40.0 : 60.0	46.8 : 53.2	38.8 : 61.2
**poly 4c**	60.9 : 39.1	67.7 : 32.3	63.5 : 36.5	31.8 : 68.2	64.2 : 35.8	53.6 : 46.4
**CsGNR 1c**	82.4 : 17.6	78.8 : 21.2	74.6 : 25.4	31.8 : 68.2	27.7 : 72.3	29.3 : 70.7

7. DFT calculation

7.1 Model compounds calculation

All density functional theory (DFT) calculation for the model compounds **2a-c** was performed using the Gaussian 16 program[6]. The B3LYP functional with Grimme's D3 correction (Becke–Johnson damping)[7] was used for geometry optimization in the ground state. The 6-31G(d) basis set was used. To analyze the weak interactions between the layers in the molecules, an independent gradient model based on the Hirshfeld partition (IGMH) method in Multiwfn was performed.[8,9] The isosurface maps were rendered by the VMD program.[10]

Geometry optimization

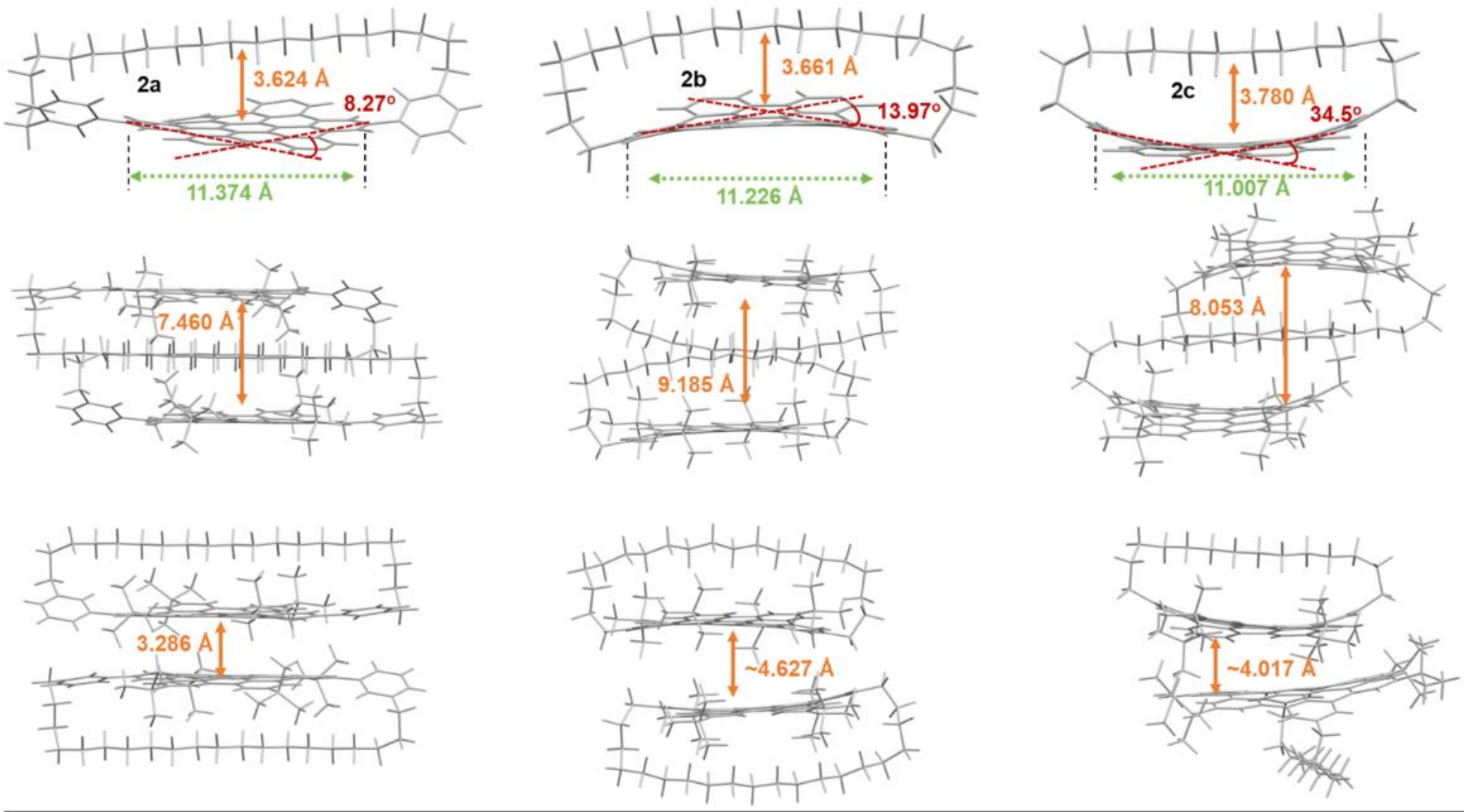

Sample	chain-to-HBC distance/Å	bridge-head C-C distance/Å	bending angle/°	F-F packing distance/Å	B-B packing distance/Å
**2a**	3.62	11.37	8.3	3.29	7.46
**2b**	3.66	11.23	14.0	4.63	9.19
**2c**	3.80	11.08	34.5	4.02	8.05

Supplementary Figure 22. Geometry optimization of model compounds **2a-c**.

As the strain increases from 2a to 2c, the bending angle increases from 8.3° to 34.5°, the chain-to-HBC distance increases from 3.62 to 3.80 Å, and the bridgehead C–C distance decreases from 11.37 to 11.01 Å. In addition, both the face-to-face and back-to-back packing distances of **2a**–**c** were calculated

(Supplementary Figure 22). The dimer models of **2a** and **2c** were extracted from their respective single-crystal structures, whereas the dimer model of **2b** was constructed based on the packing motif of **2c**, given their similar molecular structures. As expected, both the face-to-face and back-to-back packing distances increase from **2a** to **2c**. However, a slight deviation is observed for **2b**, whose intermolecular distances are slightly larger than those of **2c**. This deviation can be attributed to the larger *van der Waals* volume of the longer alkyl chains in **2b**. When constrained within the lattice of **2c** bearing a smaller van der Waals volume, the increased steric demand of these chains induces "lattice strain," forcing the HBC cores further apart. This discrepancy between the calculated and experimental results highlights the limitations of the constrained modeling approach in capturing the packing behavior of **2b**, indicating that further refinement, such as full lattice relaxation, would be required for a more accurate description.

Strain for **2a-c** was estimated by the reactions depicted in Supplementary Figure 22. The structures were optimized at the B3LYP/6-31G(d) level of theory. Then, the single point energies were calculated at the B3LYP/def2TZVP level of theory. Finally, the strain energy was estimated as the reaction energy of the following reactions:

$E_{Strain}(\mathbf{2a}) = E(\mathbf{2a}) + E(H_2) - E(\mathbf{2a'})$

$E_{Strain}(\mathbf{2b}) = E(\mathbf{2b}) + E(H_2) - E(\mathbf{2b'})$

$E_{Strain}(\mathbf{2c}) = E(\mathbf{2c}) + E(H_2) - E(\mathbf{2c'})$

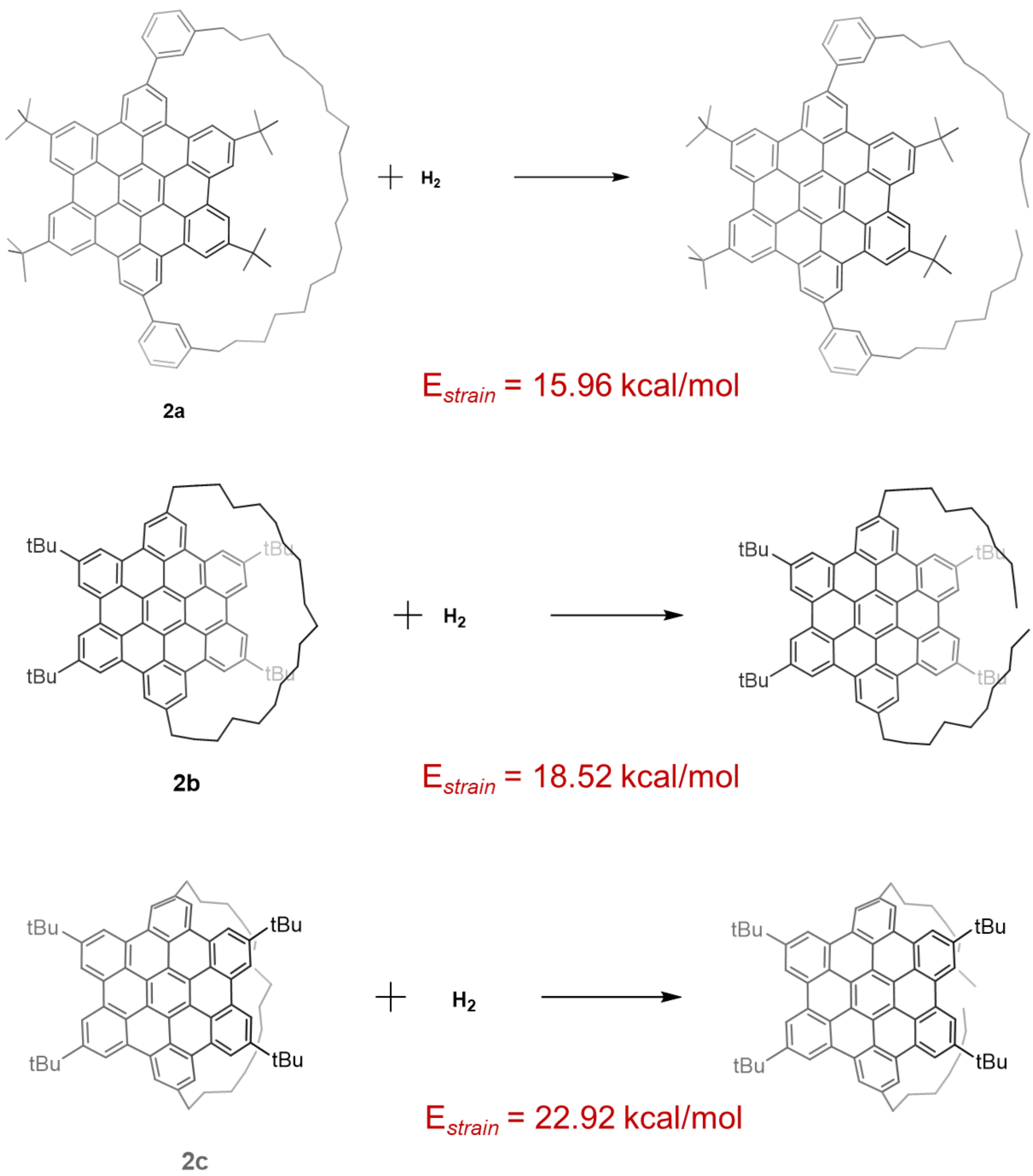

Supplementary Figure 23. Reactions used to estimate the strain energy in **2a-c**.

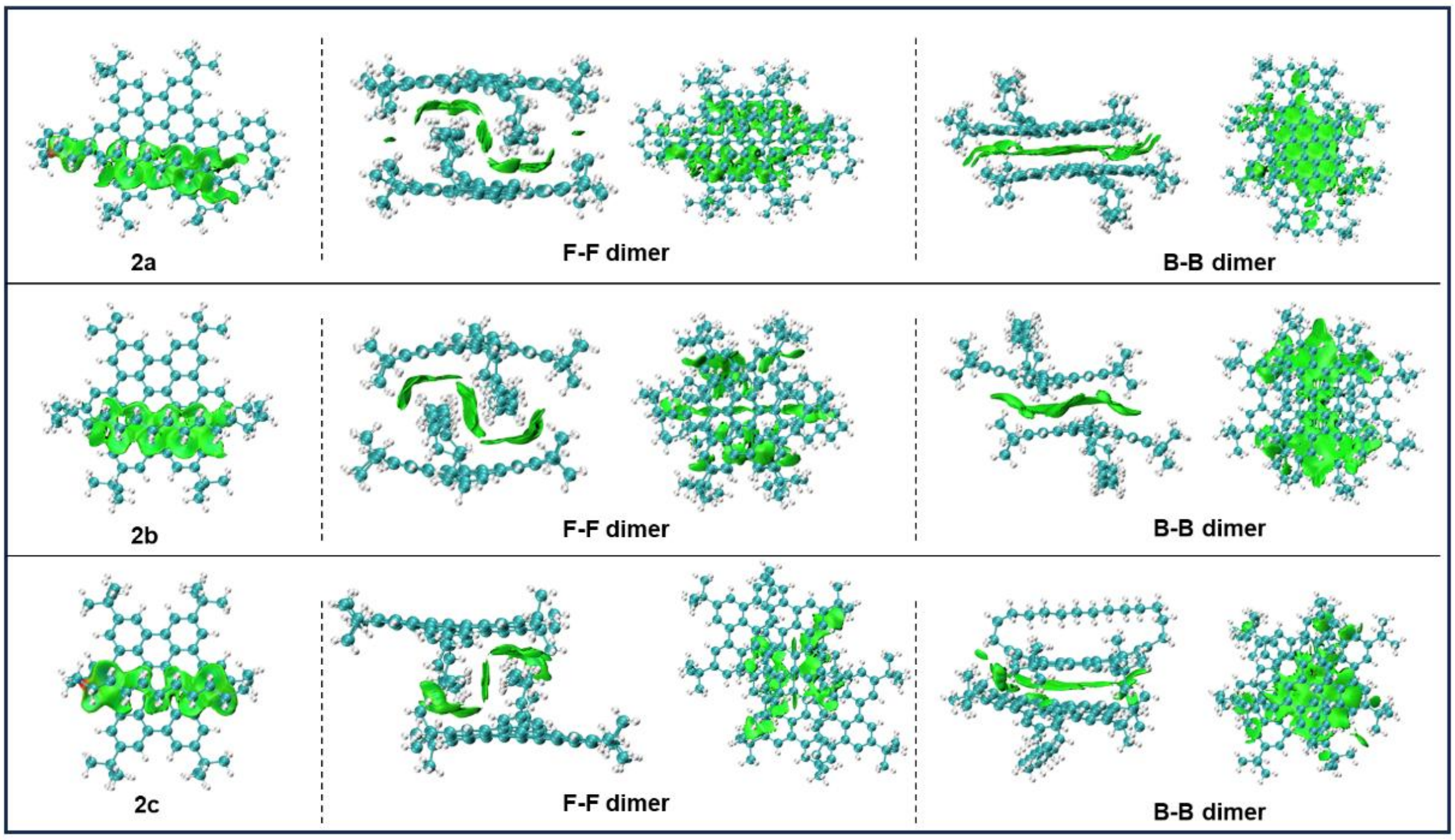

Supplementary Figure 24. Contour plots of IGMH iso-surfaces for **2a**-**c**, with green surfaces indicating the noncovalent interactions between the tether chain and main backbone.

IGMH iso-surface calculations were also conducted to evaluate the intermolecular non-covalent interactions (Supplementary Figure 24), which indicates a progressive decrease of the *van der Waals* interactions from **2a** to **2c** for both face-to-face packing dimer and back-to-back packing dimer.

7.2 CsGNRs calculations

7.2.1 Cyclophane chains on opposite sides of the GNR

The coordinates of the model structure, short oligomers, and infinite GNR (using PBC) have been optimized at the DFT level of theory with the HSE functional and 6-31G(d) basis set, using the Gaussian software. The VASP software was used to compute the phonon dispersion energy and the relative density of states (DOS). A cell of 1.48x2x3 nm was considered, in order to reproduce the transition vector of the ribbon length (x-axis) and at the same time to avoid spurious interaction with the images on the other two dimensions. The projected augmented wave (PAW) pseudopotential together with the Perdew-Burke-

Emzerhof (PBE) functional with a plane-wave cutoff energy of 500 eV was used. The convergence criteria the electronic structure energy convergence was $1x10^{-5}$ eV. To account for Van der Waals interactions, we employed the Grimme D3 method with Becke-Johnson parameters[11]. The vibrational modes were calculated at 298 K to obtain the phonon dispersion along the Gamma-X high symmetry points, with a 4x1x1 k-points grid based on the Monkhorst-Pack scheme centered at Gamma.

We define the structure with cyclic chains alternately positioned on opposite sides of the main backbone as the UD isomer, whereas the structure with all cyclic chains positioned on the same side of the backbone is defined as the UU isomer.

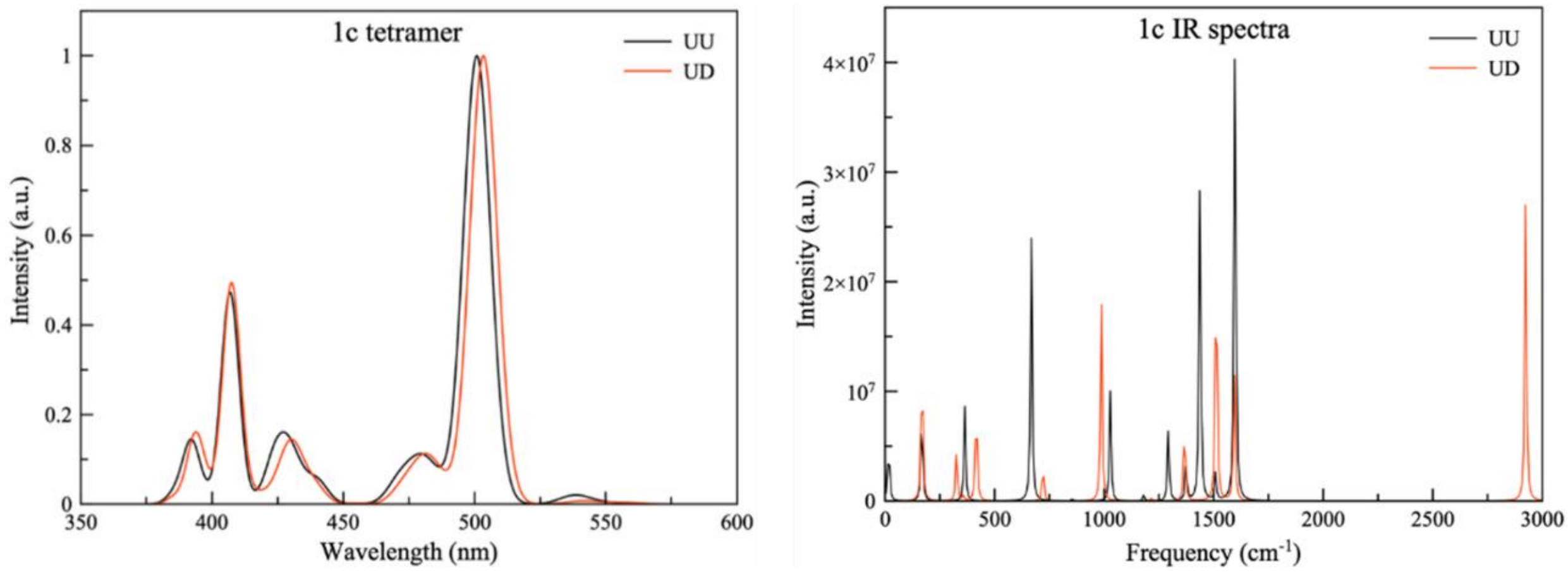

Supplementary Figure 25. The calculated absorption spectra (left) and IR spectra (right) of two isomers of the tetramer compound.

We performed theoretical calculations of the absorption and IR spectra for both regioisomers of structure **1c**. To reduce computational complexity while retaining the essential structural features, tetramer models of both regioisomers were employed to represent the corresponding GNR structures and validate the experimental results (Supplementary Figure 25). (The frequencies in the IR spectra were scaled by 0.9510 to compensate the inherent error of the used HSE functional).

The absorption spectrum of the UD regioisomer exhibits a spectral shape similar to that of the UU regioisomer, although it is slightly red-shifted by approximately 4 nm, which is in excellent agreement with the experimental spectrum shown in main text Fig. 3c. The absorption peak located at around 540 nm corresponds to a HOMO-to-LUMO transition (experimental value: 535 nm), whereas the main intensity peak at 501 nm arises from mixed HOMO−1→LUMO and HOMO→LUMO+1 transitions. These results indicate that the presence of cyclophane chains does influence the optical properties of structure **1c**; however, the magnitude of this effect is insufficient to unambiguously distinguish which regioisomer is dominant based solely on the absorption spectra.

In contrast to the absorption spectra, the IR spectra of the two regioisomers show clear differences. The most striking distinction is the absence of a peak around 3000 cm^{-1} for the UU isomer, whereas this feature is present at 2924 cm^{-1} for the UD isomer. This observation is consistent with the experimental IR spectra, in which a peak is clearly detected in this spectral region. Furthermore, the IR spectrum of the UD isomer exhibits two distinct peaks in the fingerprint region between 500 and 1000 cm^{-1}, centered at 724 and 988 cm^{-1}, respectively. These values are in good agreement with the experimentally observed peaks at 711 and 966 cm^{-1}.

Collectively, these evidences strongly suggests that, under the experimental conditions, the UD isomer predominates over the UU isomer.

Geometry of the optimized structures

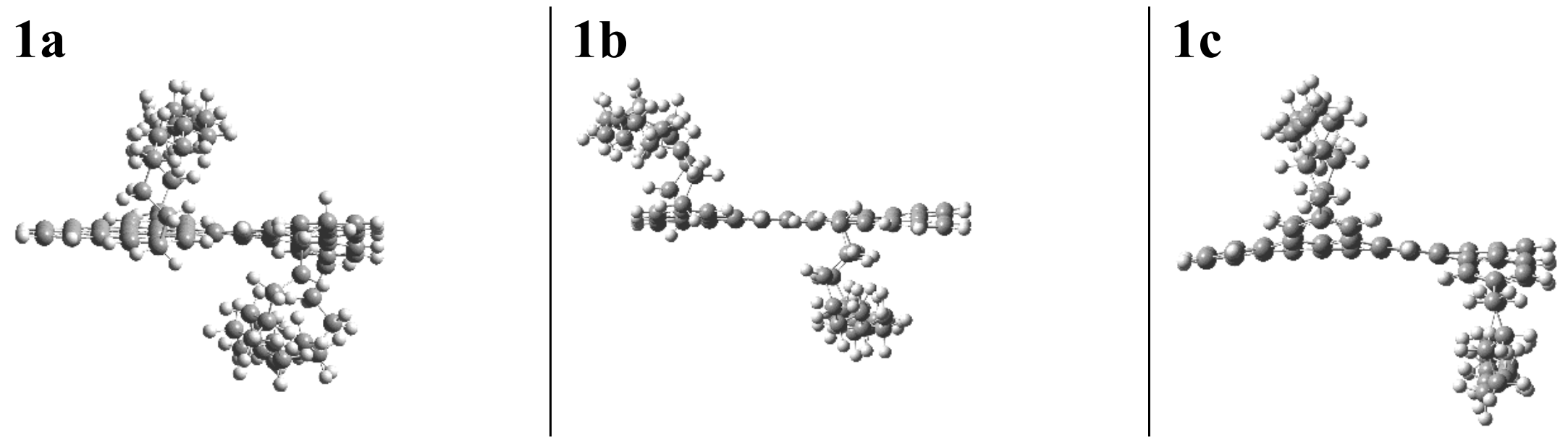

Supplementary Figure 26. The GNRs structures are reported, in which we consider the cyclophane chains on opposite sides of the GNR. A minimal tilting

in the direction along the GNR elongation axis is present, but there is a more marked bending of the edges of the structures. While **1c** shows the highest degree of distortion, both other structures also show bending along the edges.

Electronic properties

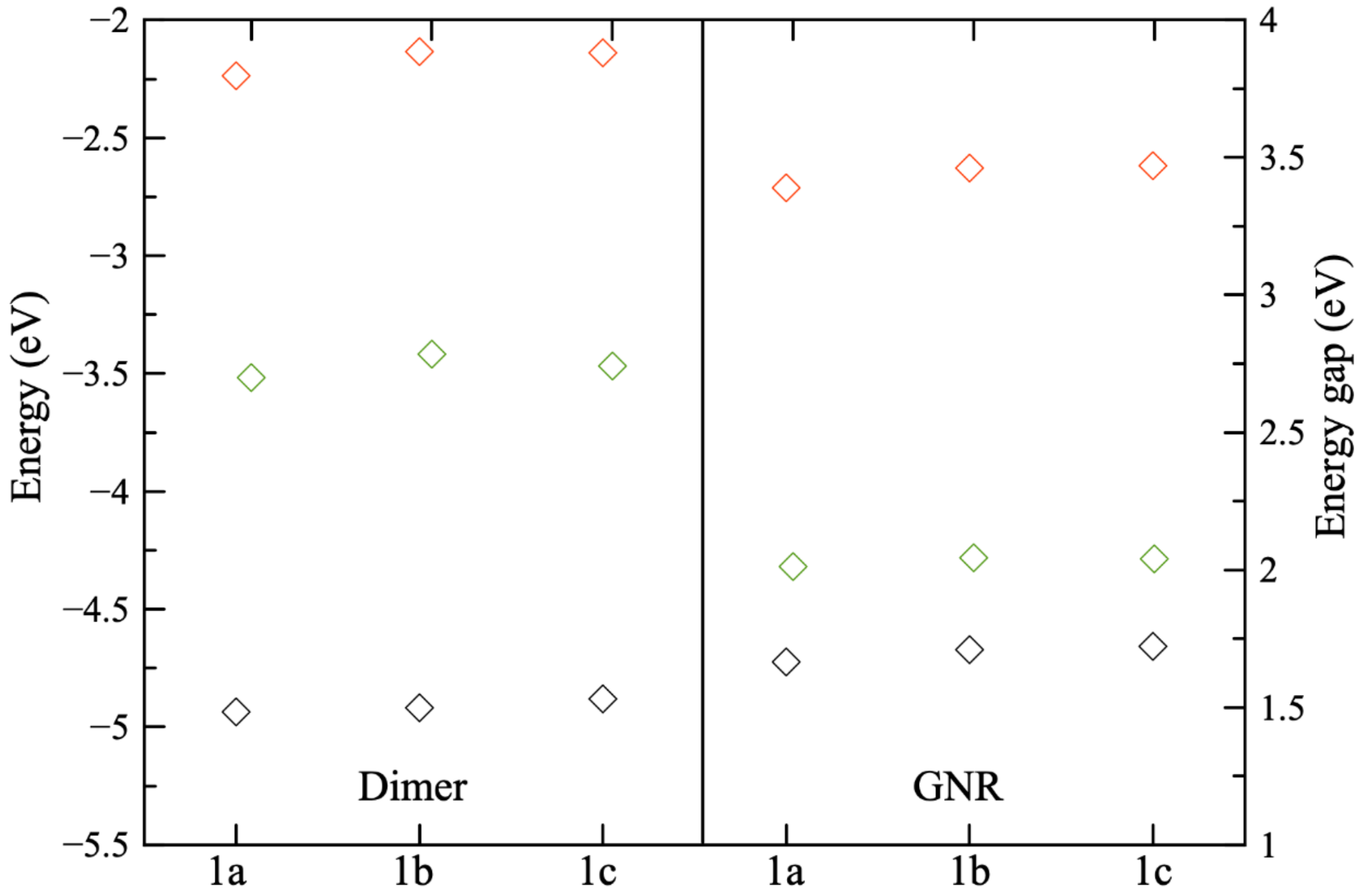

Supplementary Figure 27. Calculated energy level of the dimer and infinite CsGNRs **1a, 1b,** and **1c**. black dots refer to HOMO (VMB) values, red to LUMO (CBM) values and green dots to the energy (band) gap, respectively.

	HOMO	LUMO	VBM	CBM
**1a**	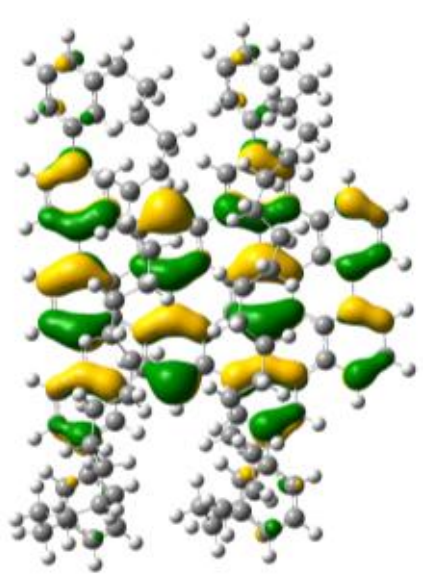	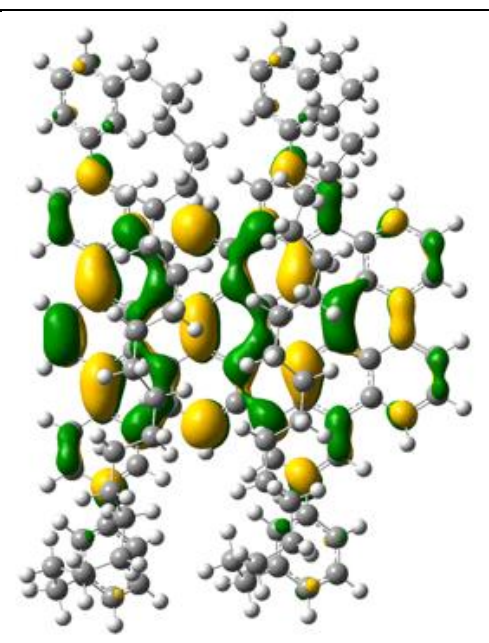	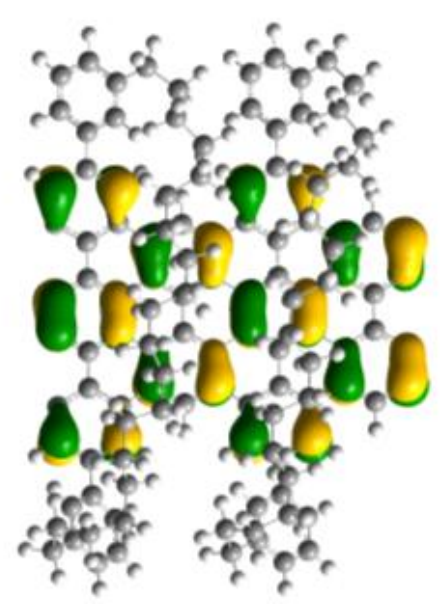	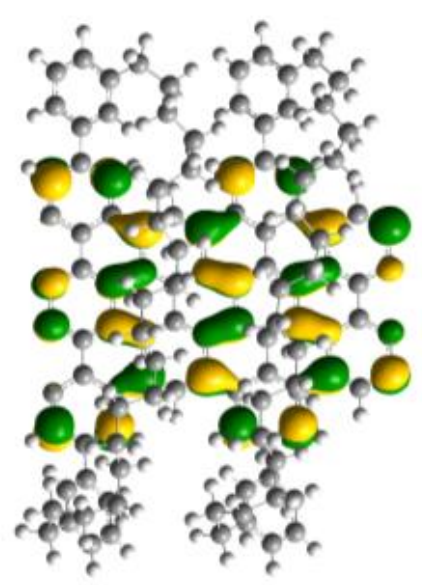

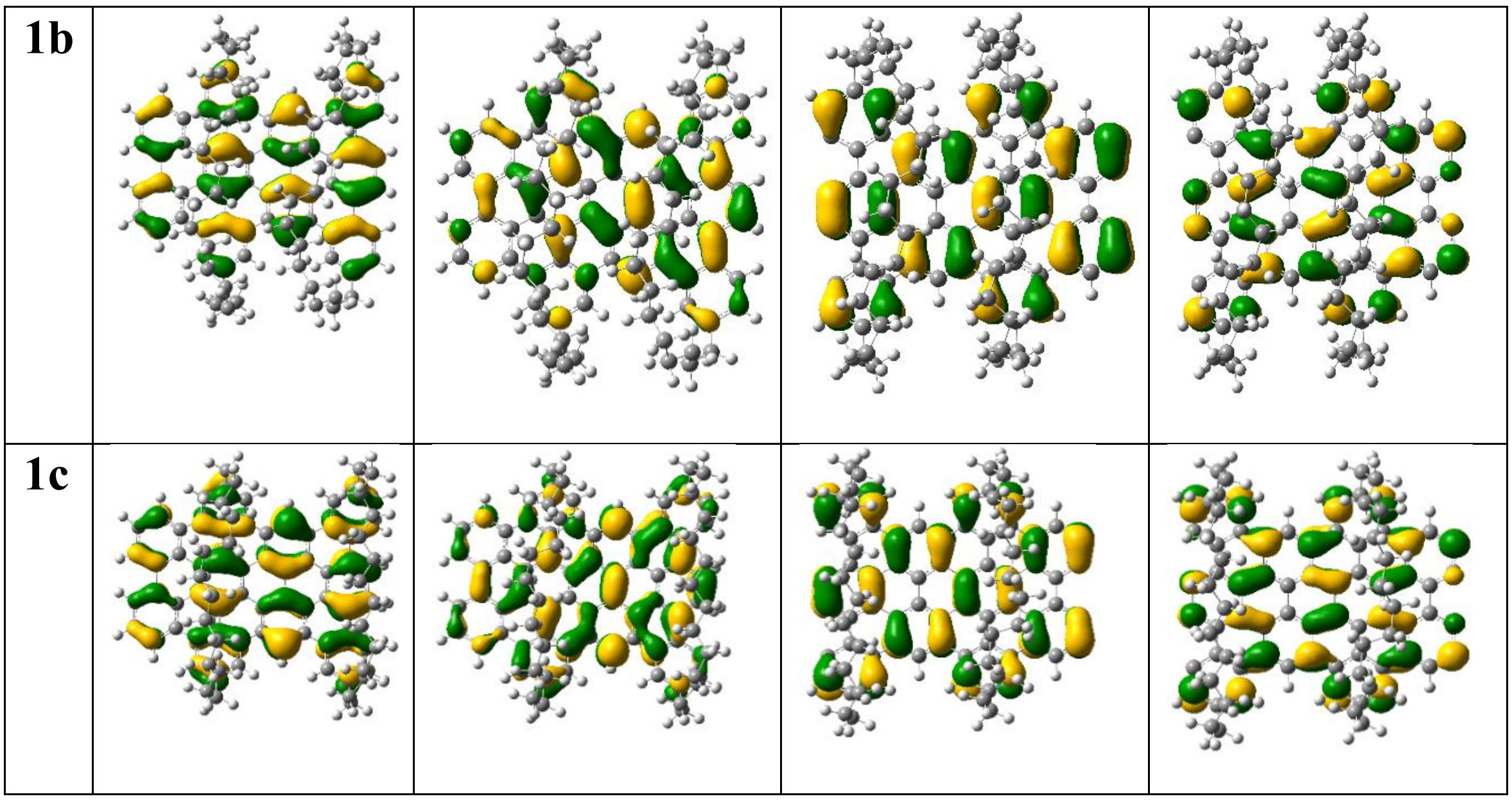

Supplementary Figure 28. Frontier molecular orbitals' shape of the three studied systems.

The frontier molecular orbitals show a distinct orientation when we consider either the dimer or the infinite GNRs. In fact, the HOMO of all three dimers has nodal planes along the direction of the dimer elongation, while for the LUMO the orbitals are parallel to the dimer elongation direction. The opposite is true for the GNRs.

For the dimers, the differences in chain length of the cyclophanes have only a minimum impact, as both frontier orbitals are slightly destabilized, leading to an overall energy gap of around 2.8 eV. The presence of the cyclophane chains at opposite sides has a minor impact in the energetic of the frontier molecular orbitals and energy gap, while the distortion they cause in the GNR structures (especially for **1b** and **1c**) lead to a significant destabilization of the BVM energy, resulting in a decrease of the bandgap, which is now comparable among the three GNRs and has value of 2.04 eV.

Reduced mass analysis

When the cyclophane chains are on the opposite side of the basal GNR, the structures are less distorted, but they have an impact on the reduced masses. For **1a** featuring phen-C20-phen as cyclic chains and **1b** with C20 as tethered chains,

they have the same value regarding the reduced masses, which are electrons and holes are 0.61 for electrons and 0.45 for holes, respectively, leading to m_0*=0.259. The strongest difference is observed for the **1c** with C14 as tethered chains, which exhibits the lowest reduced masses of m^*_h=0.46 and m^*_e=0.48 among three samples, leading to m_0*=0.23.

7.2.2 Cyclophane chains on same sides of the GNR

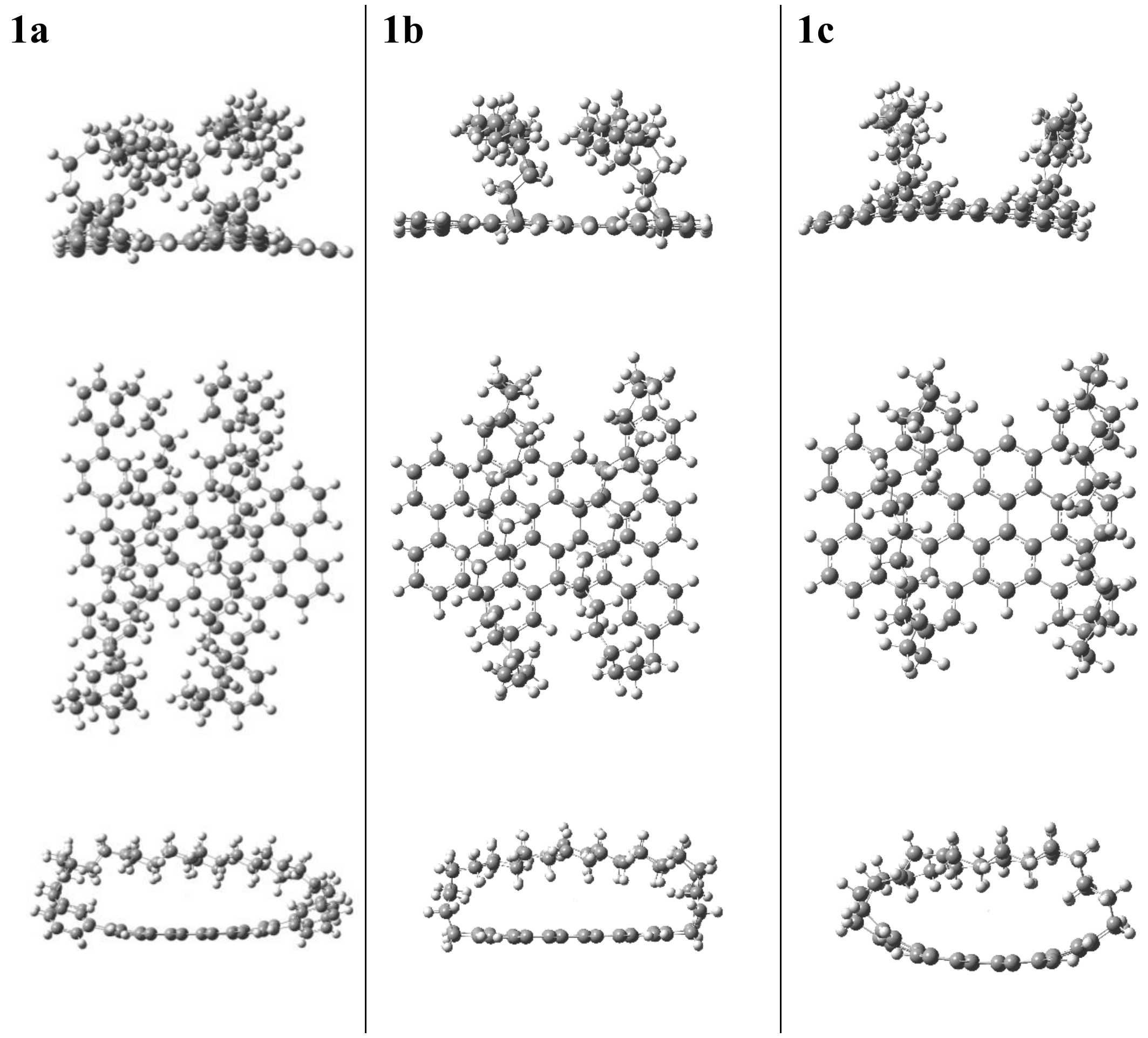

Supplementary Figure 29. The dimers and GNRs structures are reported, in which we consider the cyclic chains on the same side of the GNR plane. It is clearly visible that the tilting effect of the cyclophane chains on the structures of both dimers and polymers. This strong tilting observed in **1c** is responsible for both the differences in electronic properties and band dispersions.

Electronic properties

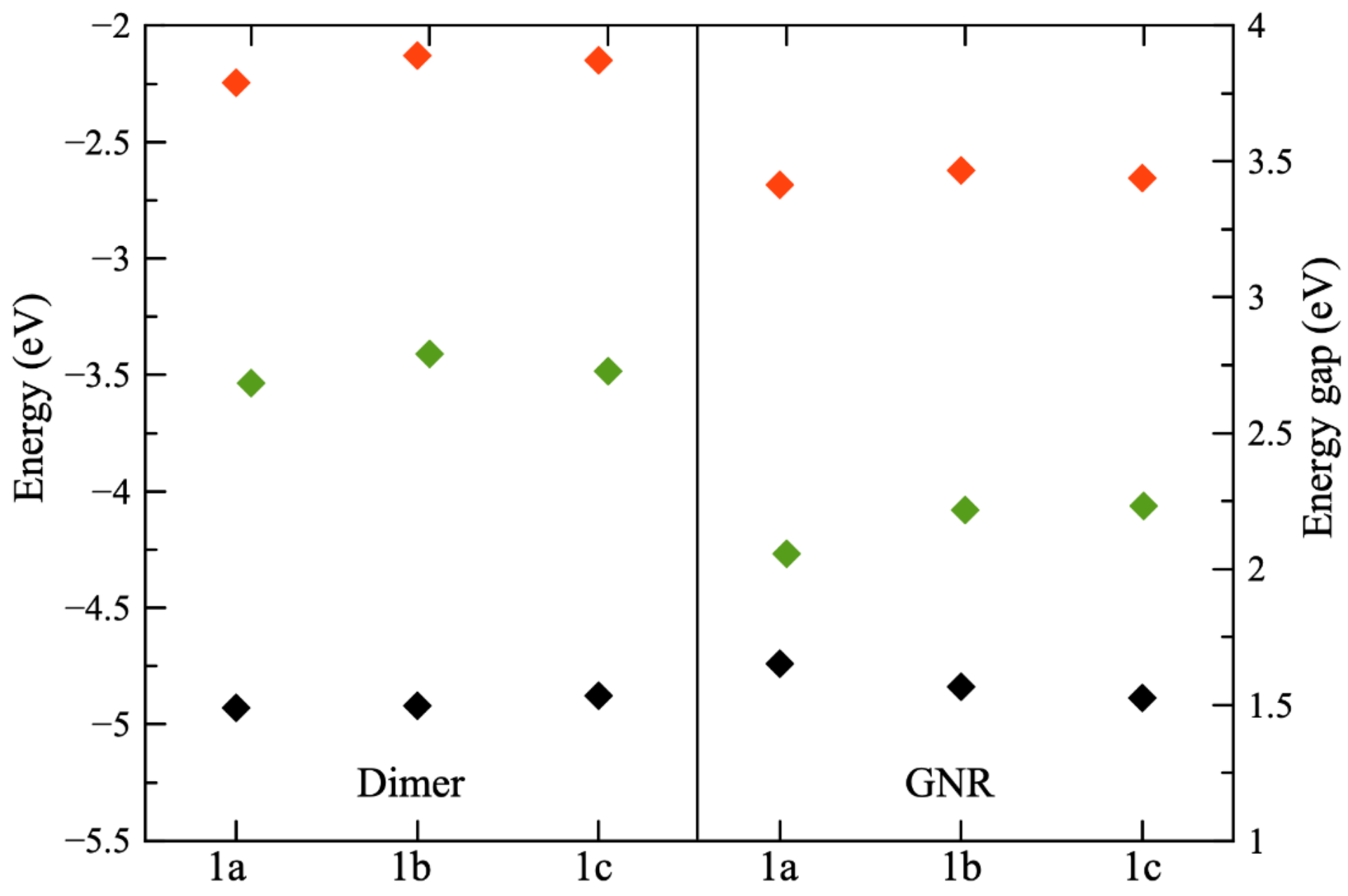

Supplementary Figure 30. Calculated energy level of the dimer and infinite CsGNRs **1a, 1b,** and **1c** when considering tethered chains on the same side of GNR plane. Black dots refer to HOMO (VMB) values, red to LUMO (CBM) values and green dots to the energy (band) gap, respectively.

For the dimers, the differences in chain length of the cyclophanes have only a minimum impact, as both frontier orbitals are slightly destabilized, leading to an overall energy gap of around 2.8 eV. Moreover, the presence of the cyclophane chains on the same side of the GNR plane has a stronger impact on the GNRs. In fact, now the presence of the phenyl group in the cyclophane chains when on the same side of the GNR plane strongly stabilizes the **1a** structure, while both its absence (**1b**) and the shortening of the chain (**1c**) lead to a destabilization of the orbitals and an increase in the bandgap, which is increased from 2 to 2.2 eV going from **1a** to **1c** GNRs.

Reduced mass analysis

When the cyclophane chains are on one side of the basal GNR, the presence of the different cyclophane lengths has a strong impact on the band dispersion of the three GNRs, which, in turn, affects the reduced mass and the mobility. For **1a**, we

compute the smallest bandgap of 2.05 eV, and m^*_h=0.45 while m^*_e= 0.51, leading to a m_0*=0.24. Decreasing the chain length leads to a steady increase of the reduced masses for both electrons and vacancies, leading to an overall decrease in mobility. The bandgap for **1b** and **1c** has the same value of 2.22 eV. The reduced masses have values of m^*_h=0.51 while m^*_e= 0.60, leading to a m_0*=0.28. For **1c** these values increase up to m^*_h=0.89 while m^*_e= 0.61, leading to a m_0*=0.36.

Electronic and phonon bands and DOS

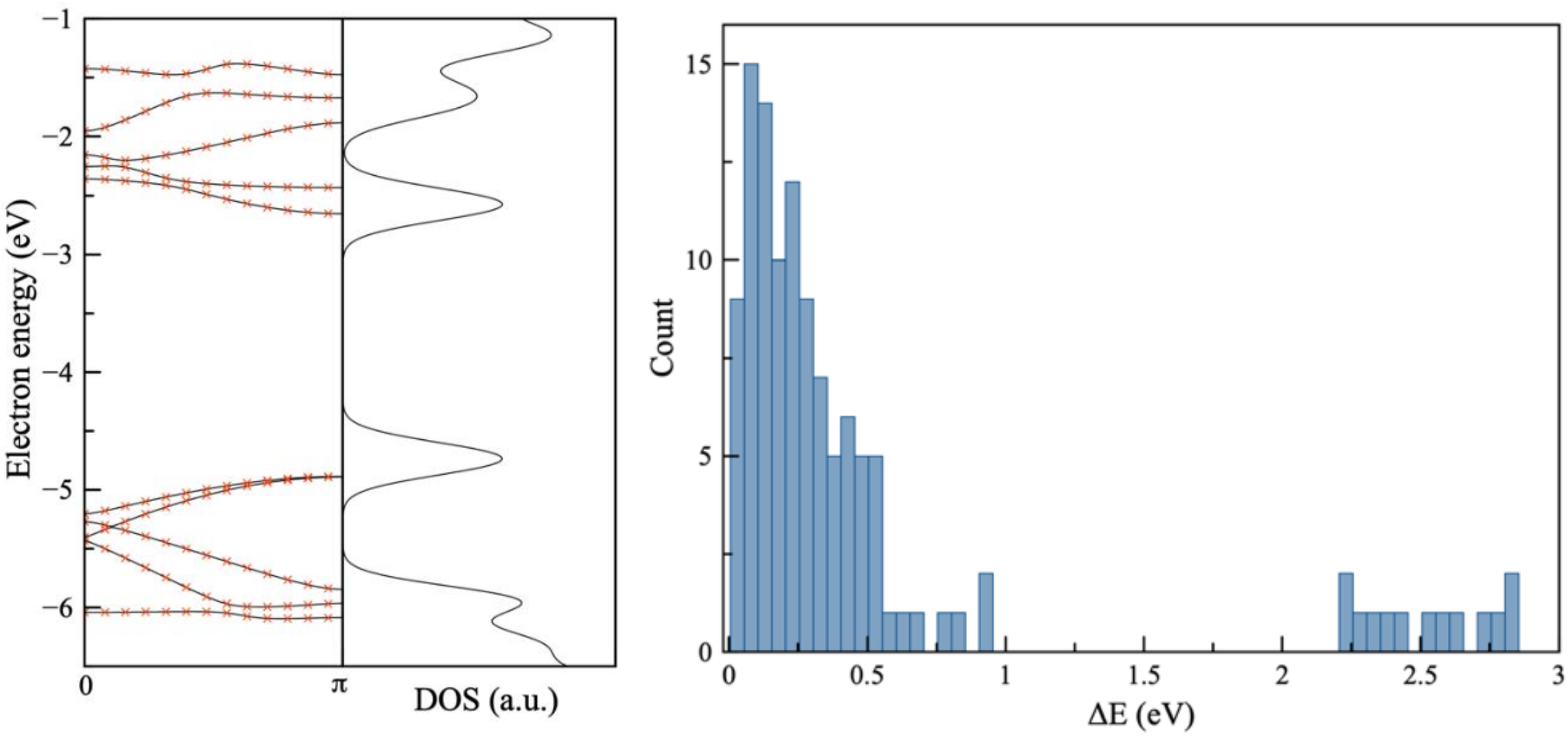

Supplementary Figure 31. Electron band structure of the **1c** with both cyclic chains on the same side of the GNR plane. The histogram of electron energy level spacing of the GNR is determined by the energy differences of neighbouring bands at various ka values (red cross in the band dispersion plot).

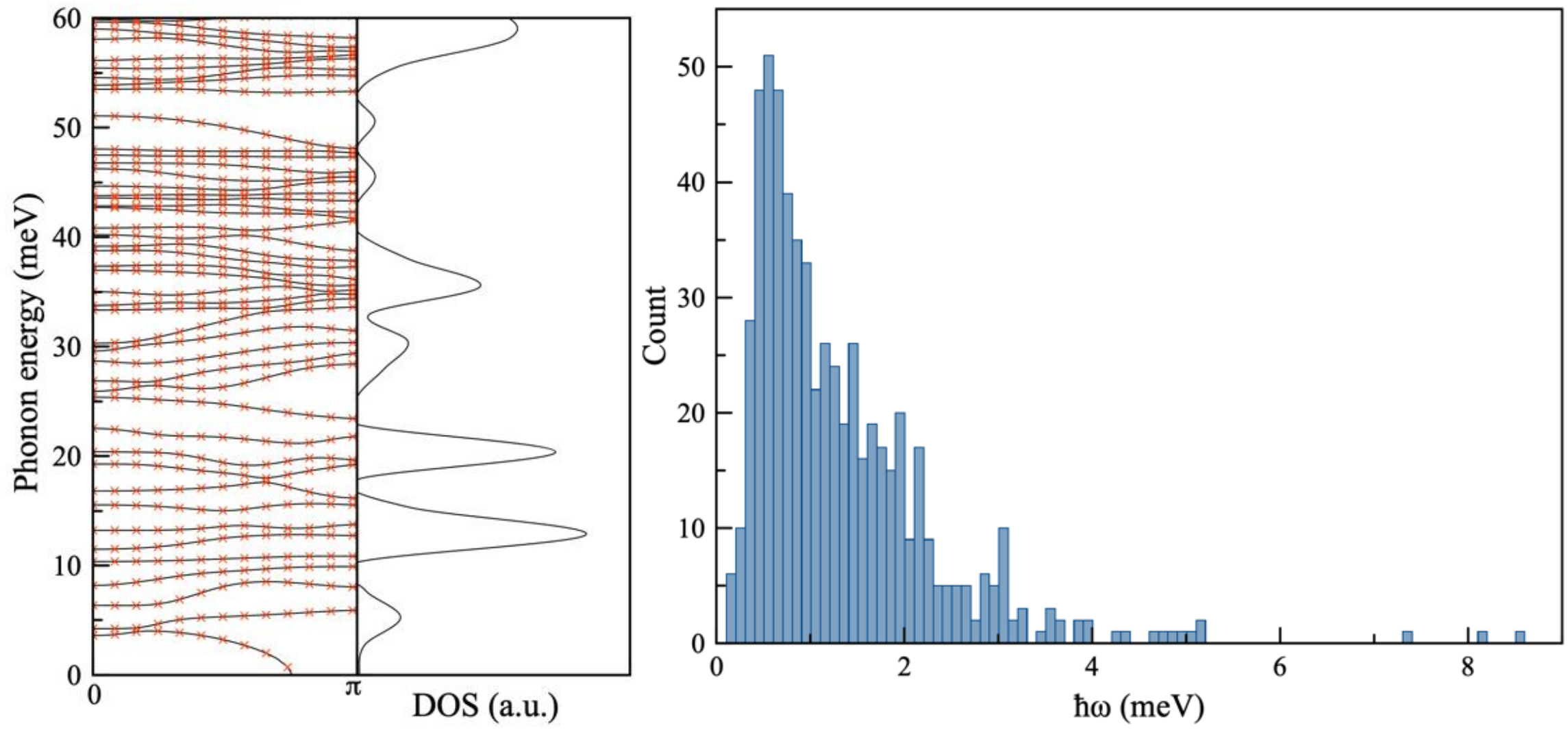

Supplementary Figure 32. Phonon band dispersion of the **1c** with both cyclic chains on the same side of the GNR plane. The histogram of electron energy level spacing of the GNR is determined by the energy differences of neighbouring bands at various ka values (red cross in the band dispersion plot). Phonon bands and DOS have been obtained without considering the cyclophane chains, to reduce the number of bands to consider and increase the clarity of the plots.

Electronic properties of reference structures

Three reference structures, consisting of the same basal GNR but different edge group substitutions, were considered and compared with the sterically shielded GNRs **1a-c**. In particular, we consider the literature reported structures with long alkyl chains (Ref1)[12], OMe substitutions (Ref2)[13], and more bulky substituents (Ref3)[14]. All these substituents introduce a certain degree of tilting in the basal structure, which increases with the size of the substituent, and in turn, affects the electronic properties.

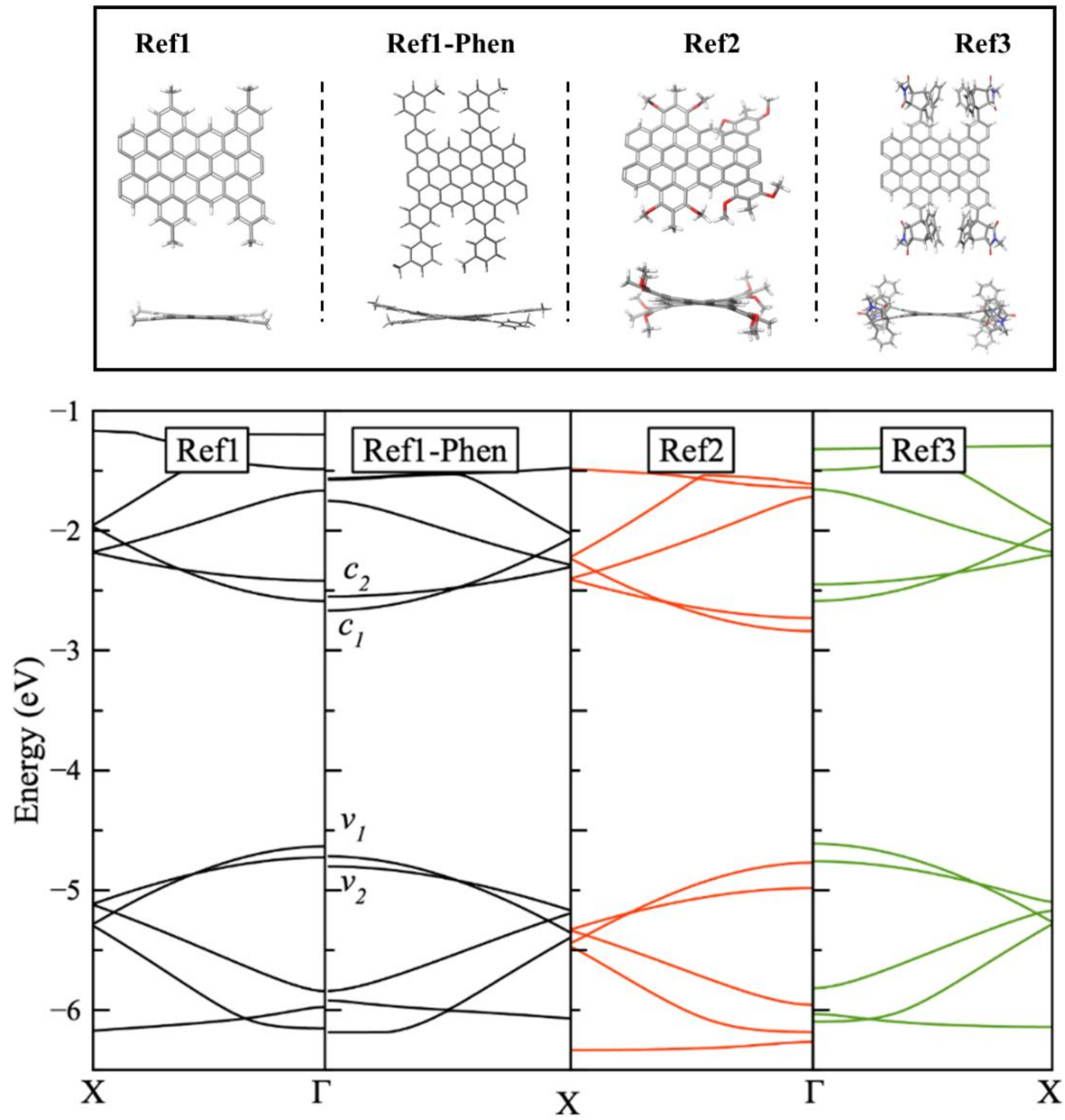

Supplementary Figure 33. Geometrical structures of the optimized repeating unit considered for the calculations and band dispersion along the elongation direction of the GNR for the three reference structures.

we performed more detailed computational analyses to elucidate the influence of cyclic chains on reduced effective mass, considering that it primarily dominates the charge carrier mobility of GNRs. As shown in Supplementary Figure 33, our calculations indicate that the presence of non-cyclic alkyl chains of different lengths (C12, C10, and C7) has a negligible effect on the electronic properties of the corresponding GNRs, as all exhibit identical band structures, consistent with those of **Ref1**. In contrast, the introduction of phenyl rings modifies the band dispersion, leading to a decrease in the reduced effective mass from 0.259 m_0 (**Ref1**) to 0.232 m_0 (**Ref1-phen**). When comparing **Ref1-phen** with **CsGNR 1a**,

only a subtle change in the reduced effective mass is observed (from 0.232 to 0.238 m_0). Likewise, for the comparison between **Ref1** and **CsGNR 1b**, no discernible changes in either the bandgap or the reduced effective mass are found. These results indicate that when the cyclic alkyl chains are sufficiently long, they do not induce significant backbone distortion and therefore exert little influence on the band structure, rendering them effectively equivalent to non-cyclic alkyl chains.

To further support this conclusion, we synthesized a new reference GNR (**Ref4**) with linear C7 alkyl chains as a representative example to compare its photoconductivity with that of CsGNR **1b** and **1c**. Specifically, **Ref4** enables a direct comparison with CsGNR **1c** that bears C14 cyclic chains and exhibits the greatest internal bending strain among all CsGNRs. The photoconductivity of **Ref4** is also evaluated using THz spectroscopy. As shown in Supplementary Figure 34, **Ref4** exhibits a pronounced reduction in photoconductivity compared with **1c**. Drude–Smith model fitting of the frequency-resolved photoconductivity reveals a significantly shortened charge scattering time, decreasing from 42 fs for **1c** to 28 fs for **Ref4**. Meanwhile, DFT calculations indicate a non-negligible increase in the reduced effective mass from 0.235 m_0 for **1c** to 0.259 m_0 for **Ref4**. The combined effects of reduced scattering time and increased effective mass account for the markedly lower charge carrier mobility observed for **Ref4**.

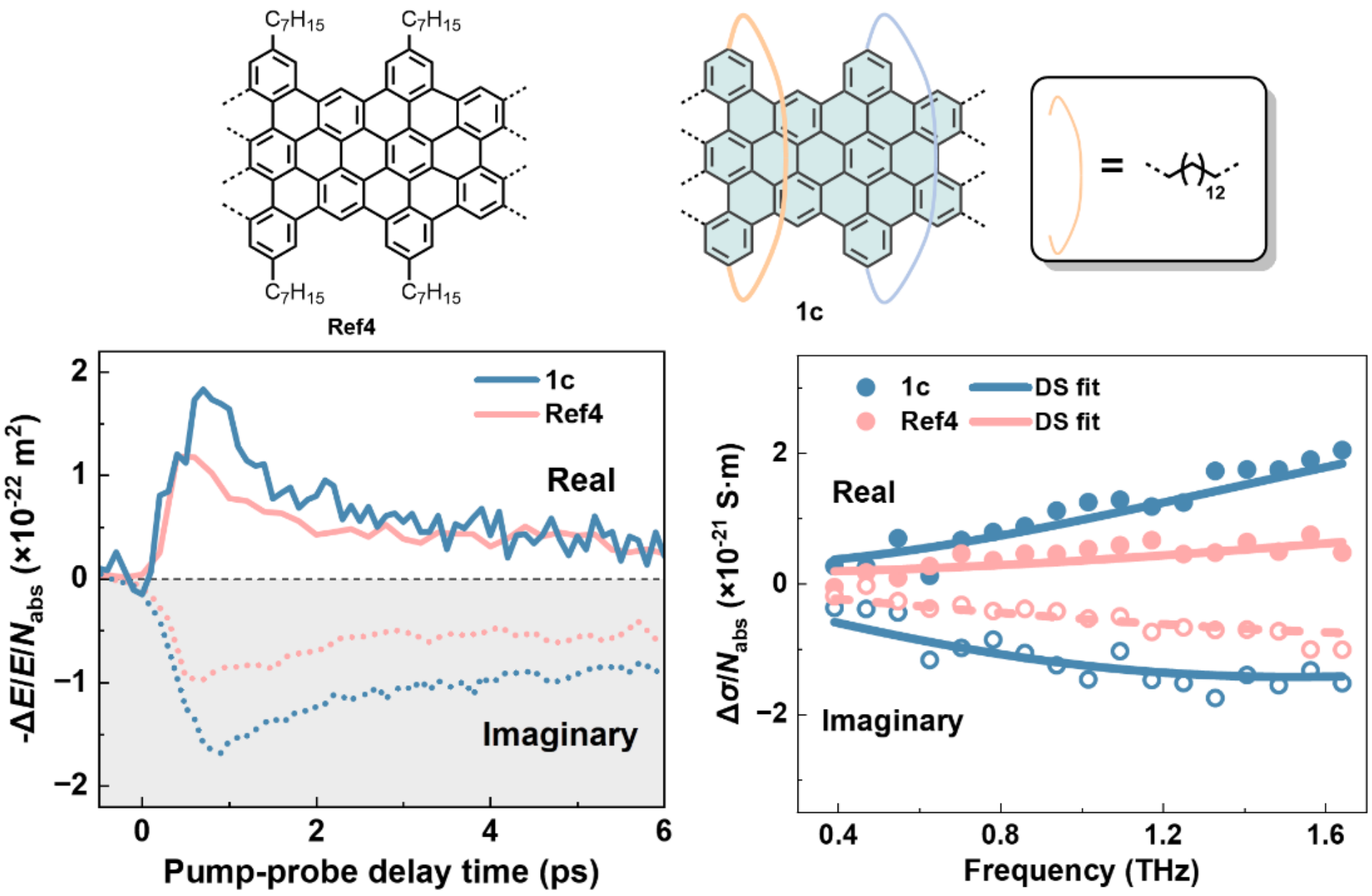

Supplementary Figure 34. Comparison of the terahertz transient photoconductivity of **Ref4** and **1c**.

Moreover, the THz result of **Ref4** was also compared with that of CsGNR **1b** bearing a C20 cyclic alkyl chain, and both samples exhibit nearly identical photoconductivity profiles (Supplementary Figure 35), suggesting that the cyclic chains in **1b** exert a similar effect to the noncyclic chains in **Ref4.**

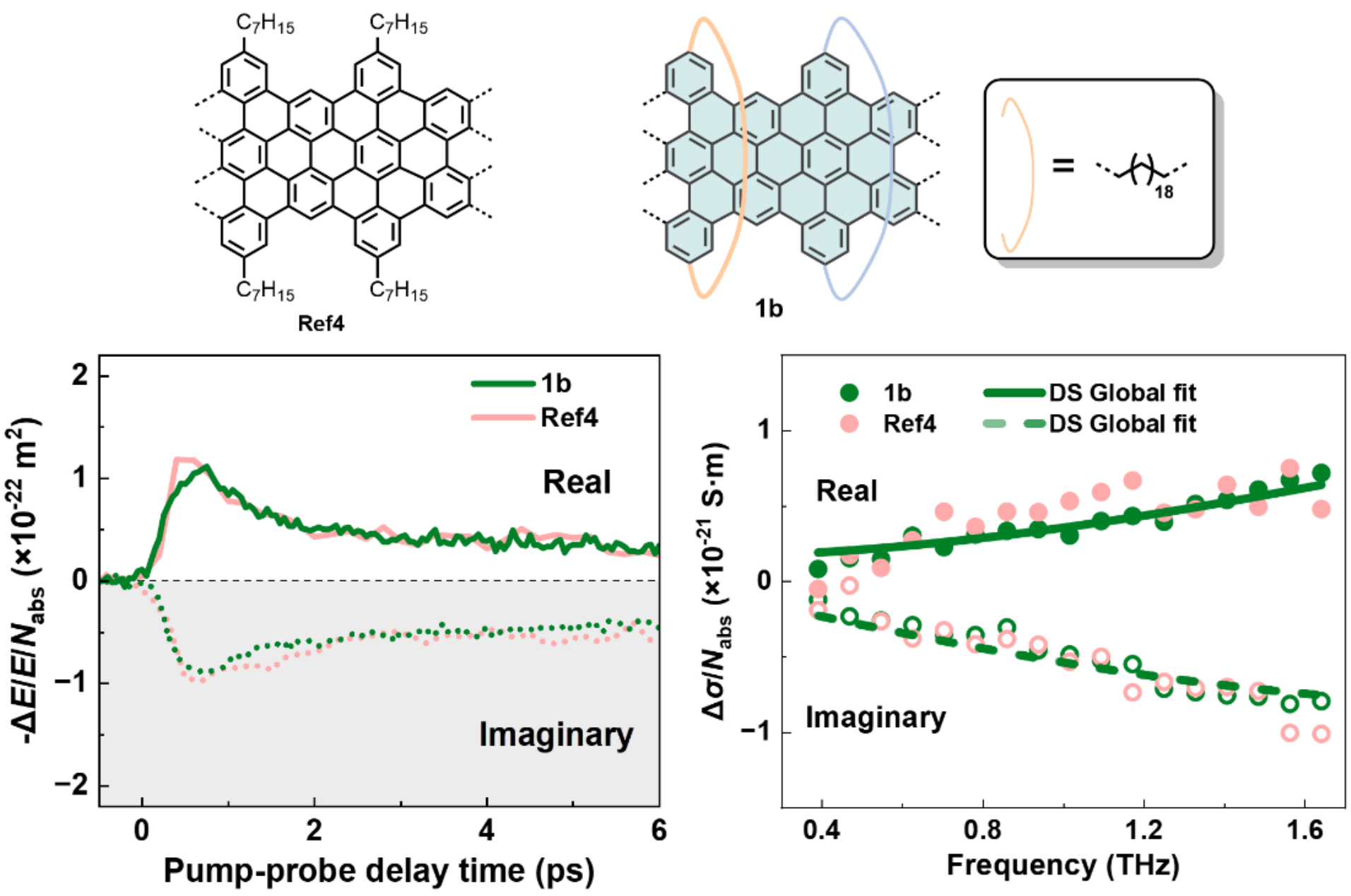

Supplementary Figure 35. Comparison of the terahertz transient photoconductivity of **Ref4** and **1b**.

Collectively, these results demonstrate that cyclophane chains influence charge carrier mobility only when the internal bending strain, modulated by the length of the cyclic chains, is sufficiently large to induce noticeable geometric distortion of the GNR backbone.

By changing the type of substituents, it not only reduces the bandgap, which decreases from 2.05 to 1.93 eV from **Ref1** to **Ref3**, but is also responsible for the different band dispersion observed. This leads to different reduced rest mass values of 0.26 m_0 for **Ref1** to 0.22 and 0.21 m_0 for **Ref2** and **Ref3**, respectively. Measured charge scattering times τ are 27 fs for **Ref2** and 23 fs for **Ref3**, respectively. Therefore, the estimated short-range charge carrier mobility μ_{int} (= $e\tau/m^*$) is 216 for **Ref2** and 187 $cm^2\ V^{-1}\ s^{-1}$ for **Ref3**, respectively.

8. THZ spectroscopy

As a contact-free technique, THz spectroscopy enables characterization of short-range charge transport in GNRs, benefiting from the transient single-cycle electrical pulse nature of the THz field with ~1 ps duration.

Time-resolved terahertz (THz) measurements were powered by a commercial mode-locked Ti:sapphire femtosecond laser (central wavelength: 800 nm; pulse duration: 50 fs; repetition rate: 1 kHz). The laser output was split into three branches for THz generation, electro-optic sampling, and optical pumping. The THz pulses were generated via optical rectification in a (110)-cut ZnTe crystal driven by the 800 nm fundamental pulse. The emitted THz radiation was collimated and focused through a 1 mm-thick quartz cuvette containing the CsGNR solution, using a pair of off-axis parabolic mirrors. After transmission, the THz field was re-collimated and refocused onto a second ZnTe crystal for electro-optic detection, where it was sampled by a variably delayed weak 800 nm probe pulse via the Pockels effect. The optical pump pulse at 400 nm was produced by frequency-doubling the 800 nm fundamental in a β-barium borate (BBO) crystal. Temporal overlap between the THz probe and the optical pump was controlled by a motorized delay stage. All measurements were conducted under dry nitrogen atmosphere to minimize absorption by ambient water vapor.

The Drude-Smith (DS) model, a phenomenological modification from the classical Drude model, has been widely employed to evaluate the charge transport properties in nanostructured semiconductors. The frequency-dependent conductivity spectra of CsGNRs are fitted by the DS model using the expression:

$$\sigma(\omega) = \frac{\omega_{\mathrm{p}}^2 \varepsilon_0 \tau_{DS}}{1 - i\omega\tau_{DS}}\left(1 + \frac{c}{1 - i\omega\tau_{DS}}\right)$$

where τ, ω_p and ε_0 are the Drude-Smith charge scattering time, the plasma frequency, and vacuum permittivity, respectively. In the DS model, the parameter

c represents the backscattering probability for carrier transport, and the typical values for c ranges from 0 (free charge) to -1 (preferential backscattering).

9. Single-Electron Transistor Devices

The device is fabricated as follows. First, a 150-nm-wide local gate is patterned on SiO_2. These gates consist of 5/15 nm of Cr/Pt and are covered by a 30 nm Al_2O_3 layer deposited using atomic layer deposition, acting as the gate dielectric. Polycrystalline graphene was synthesized via chemical vapor deposition, transferred, and pre-patterned. After that, graphene nanogap (3-10 nm) were formed via electrical breakdown. The graphene gaps were electrically characterized to ensure a clear separation between the electrodes. Devices with currents greater than 20 pA at $V_{Bias} = 1$ V were excluded from further characterization.

The **GNRs** were deposited into the junctions from a ~5 µl suspension prepared by sonicating powders in toluene (0.01 mg ml^{-1}) for 30 min. *IV* characteristics were recorded for all devices immediately after drop-casting of **GNRs**; the presence of **GNR** was detected by an increase of the current from a few pA (noise level) to more than 1 nA.

The preselected GNR devices were measured at a low temperature under vacuum conditions (<10^{-6} mbar). The devices were measured in a commercially available ^{3}He refrigerator (Oxford Instruments, model HelioxVL) at a base temperature of 255 mK and a vacuum condition (<10^{-6} mbar). A data acquisition board (ADwin-Gold II, Jäger Computergesteuerte Messtechnik) is used to apply the bias and gate voltages and read the voltage output of the current–voltage converter (DDPCA-300, Femto Messtechnik). All the devices were measured in a two-terminal setup, where we applied a bias voltage and measured the current, from which the differential conductance was calculated by taking the numerical derivative.

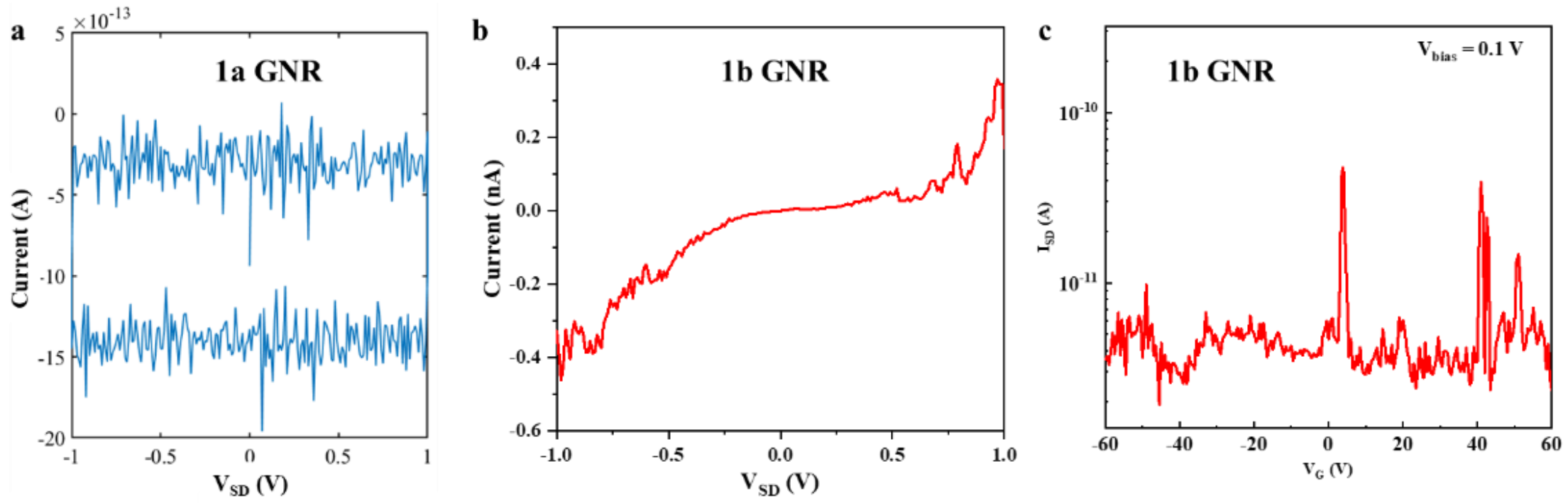

Supplementary Figure 36. Electrical characterization of CsGNR **1a** and **1b** devices. (a) Representative I-V curve for a device fabricated from 1a, showing only noise. (b, c) I-V and transfer curves of the sole functional device from batch 1b, measured at room temperature.

To compare the device performance of CsGNR **1c** against **1a** and **1b**, we prepared two additional control samples, each containing 100 nanogap devices. The nanogap fabrication and GNR deposition methods were identical to those used for **1c**. For sample **1a**, no devices exhibited a detectable signal at room temperature; only noise was observed at bias voltages up to 1 V (see representative IV, Supplementary Figure 36a). For molecule **1b**, only one device showed an

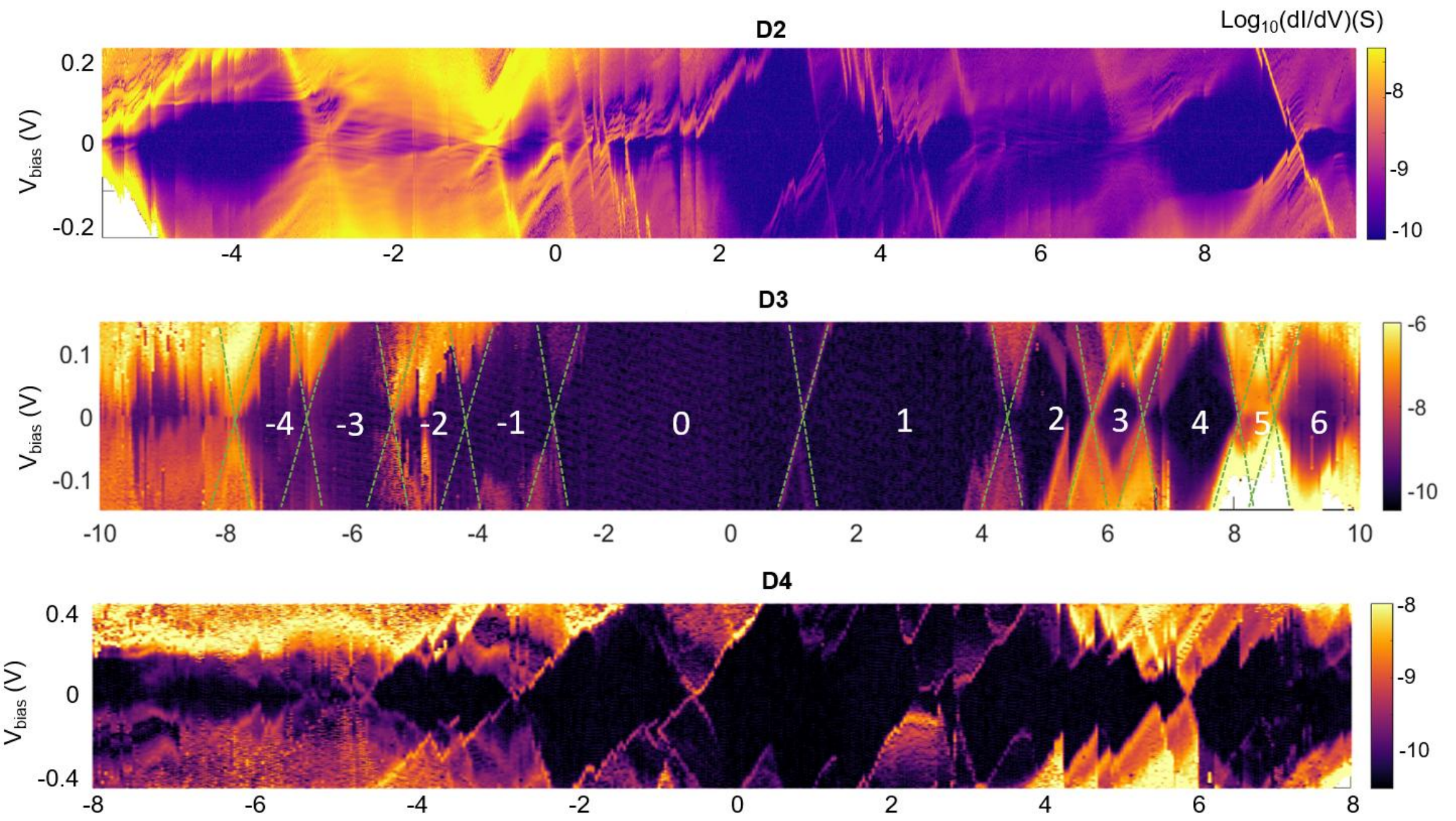

Supplementary Figure 37. Overview of transport characteristics (stability diagrams) at low temperature for Devices 2-4.

appreciable current at room temperature (0.4 nA at 1 V, Supplementary Figure 36b). However, transfer curve measurements at a 0.1 V bias revealed negligible gate modulation (Supplementary Figure 36c), with only a few peaks visible. Subsequent cooling to 10 K for low-temperature measurement yielded no observable signal. We attribute the near-zero yield and poor performance of **1a** and **1b** to the insufficient dispersibility of their respective GNRs.

The full stability diagrams of D2, D3, and D4 can be found in Supplementary Figure 37. To quantitatively address the coupling strength in our devices, we extracted the total tunnel coupling (Γ) between the GNR and the leads by applying the Breit-Wigner model to the resonant transport features on devices D1-D4, as detailed in Supplementary Figure 38 below. The extracted Γ values range from 0.18 to 2.35 meV, with an average value of 0.93 meV and a median of 0.75 meV.

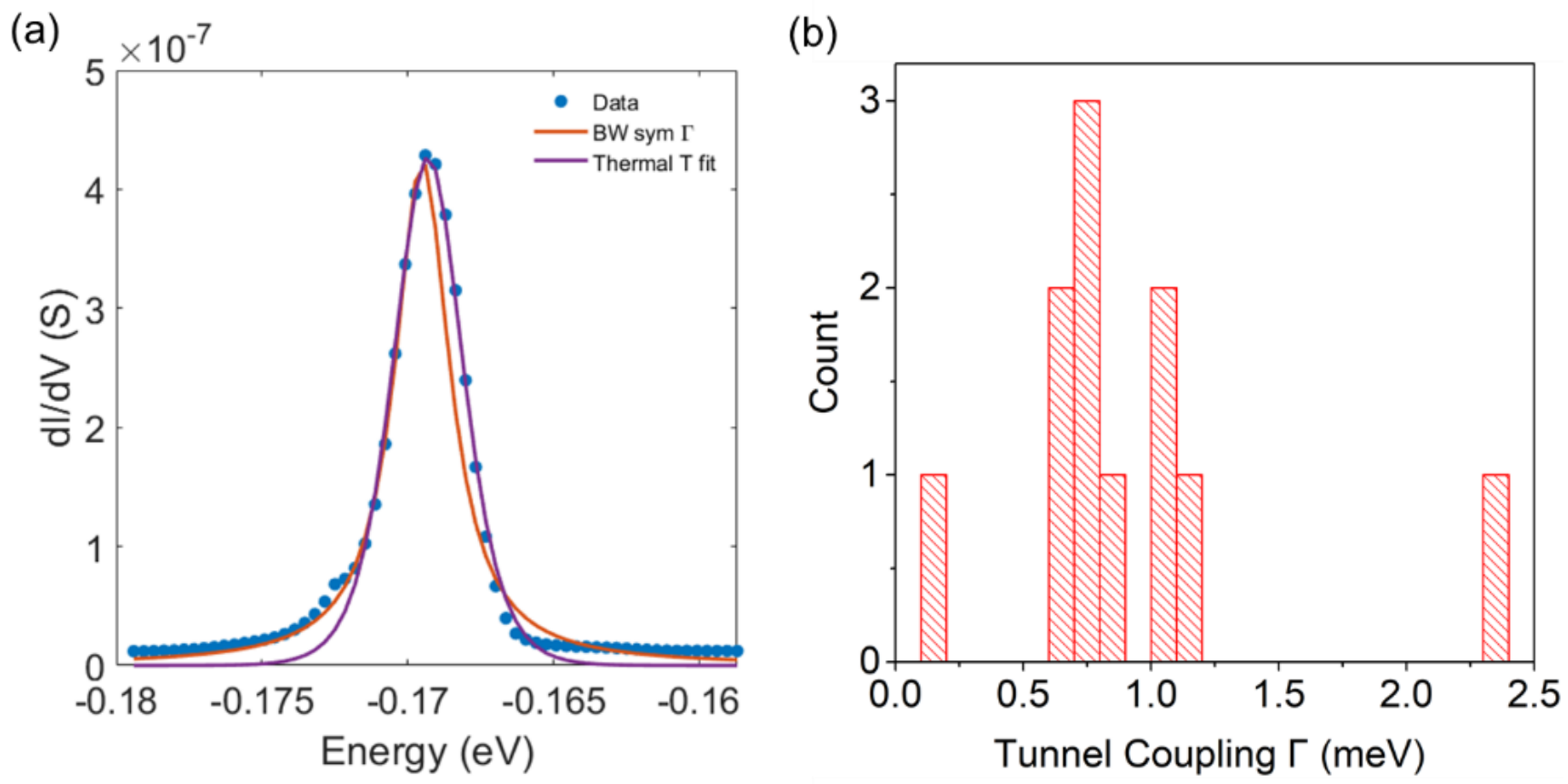

Supplementary Figure 38. Tunnel coupling extraction for GNR-graphene contacts. (a) Example fitting showing Γ = 1.14 meV. (b) Statistical distribution of Γ across all devices.

The performance variability in our current devices stems from several sources. While the width and edge structure of the GNRs are well-defined by synthesis, their length remains not well-controlled. Furthermore, the graphene nanogap electrodes formed by electrical breakdown are not atomically precise, with their position, shape, and size (typically 3-10 nm) varying stochastically. This, combined with the random position and binding geometry of a GNR within the

gap, leads to significant variability in the contact resistance and gate coupling. Additional factors include charge puddles and dangling bonds on the device substrate (Al_2O_3 in our case), as well as polymer residues. These geometric and fabrication-level issues are the primary cause of operational failures. As a supplementary note, stress during wire-bonding and cooldown could lead to signal degradation or complete device failure.

To address these challenges, key improvements must focus on deterministic fabrication. First, synthesizing longer GNRs with single dispersibility would ensure a sufficient contact length, improving stability and electronic coupling. More robust solutions involve engineering the graphene nanogap with a precise shape and performing chemical modifications at the electrode or GNR terminations to form covalent contacts, while contacting only a single GNR. Furthermore, device stability can be significantly enhanced by using encapsulation layers, such as hexagonal boron nitride or aluminum oxide. By implementing these engineering solutions, we can advance towards creating reliable and predictable nanoelectronic devices.

10. NMR and Maldi-TOF Mass Spectra

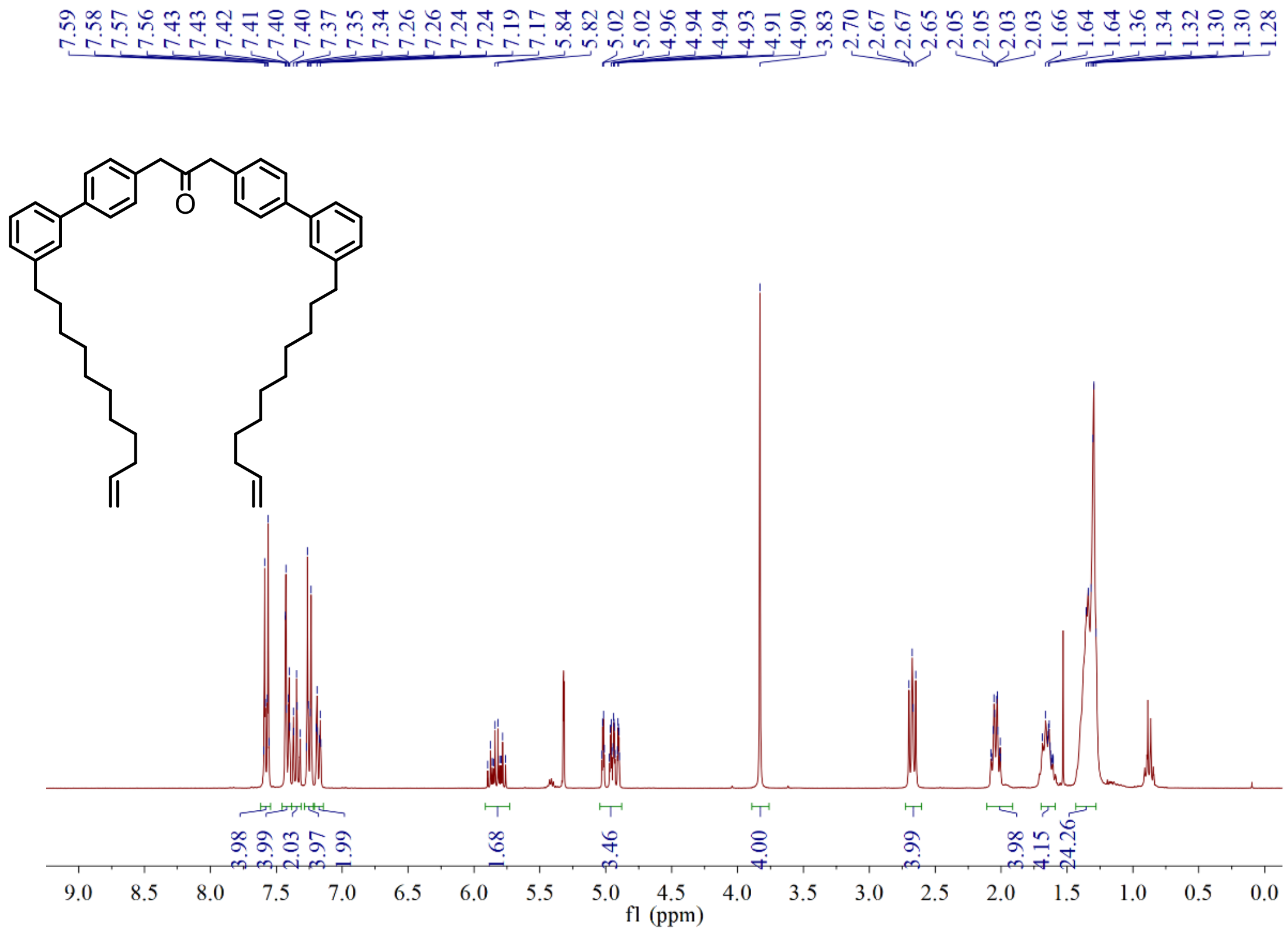

Supplementary Figure 39. ^{1}H NMR spectrum (300 MHz) of **9a** in CD_2Cl_2 at room temperature.

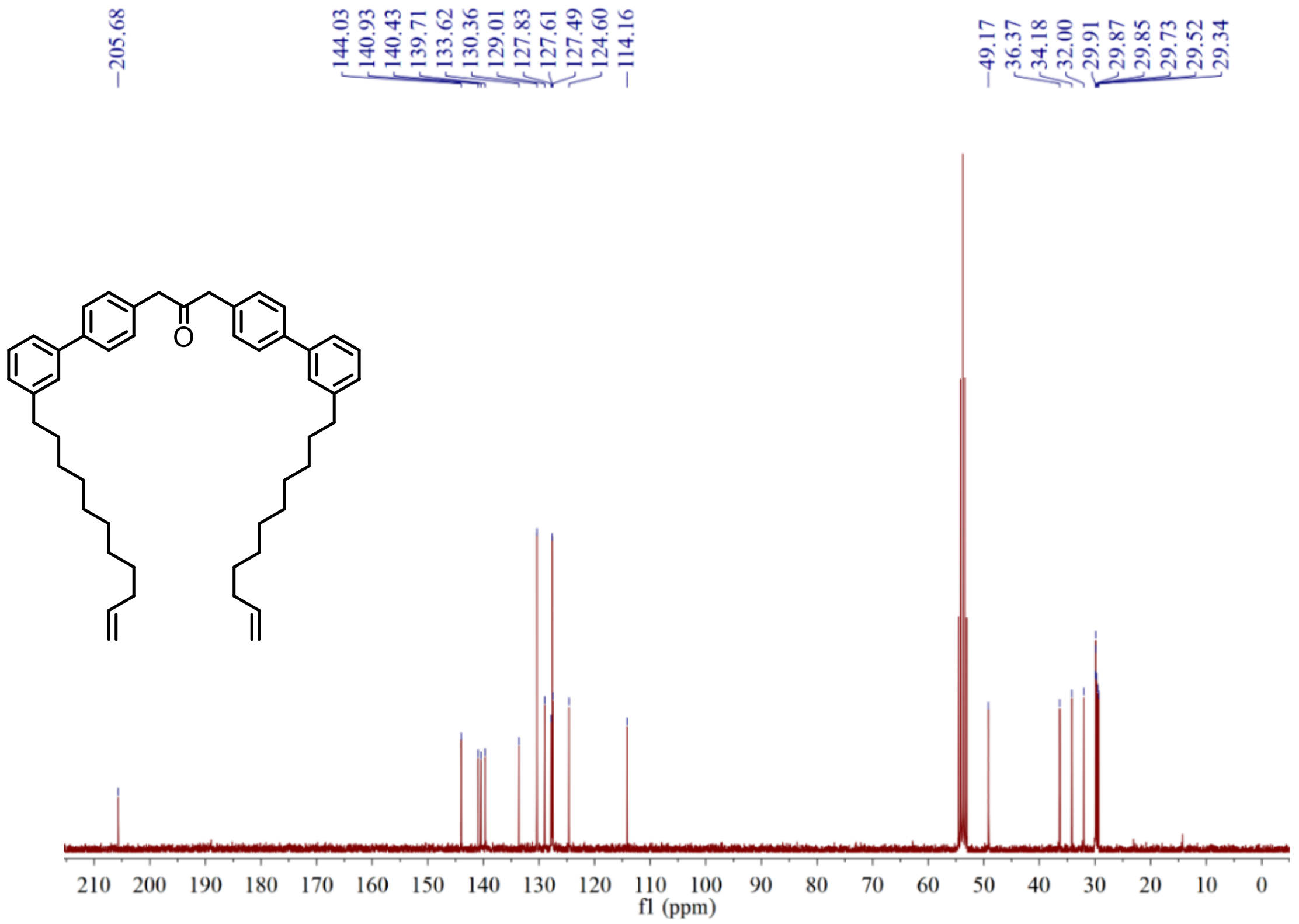

Supplementary Figure 40. ^{13}C NMR spectrum (75 MHz) of **9a** in CD_2Cl_2 at room temperature.

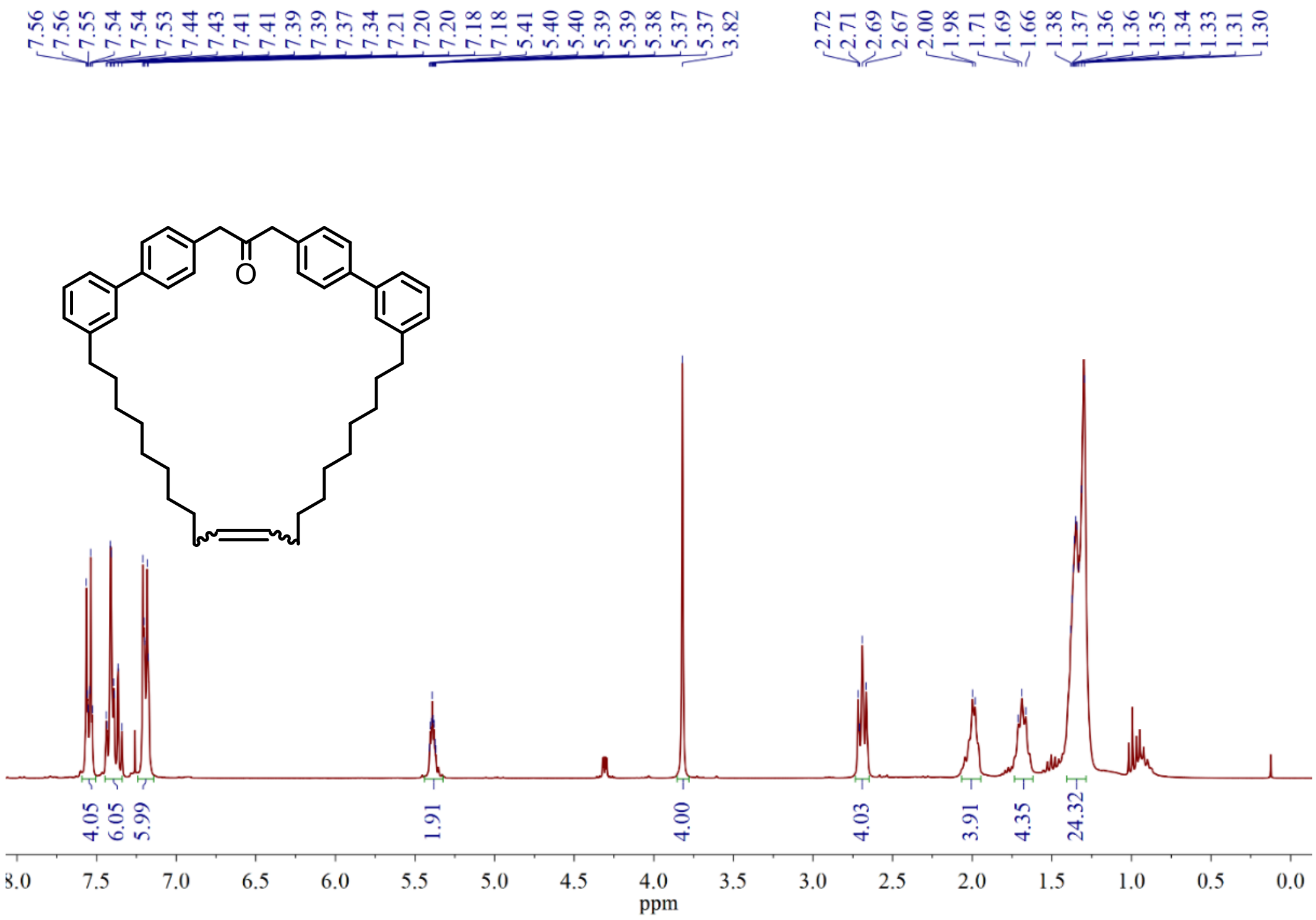

Supplementary Figure 41. ^{1}H NMR spectrum (300 MHz) of **8a** in $CDCl_3$ at room temperature.

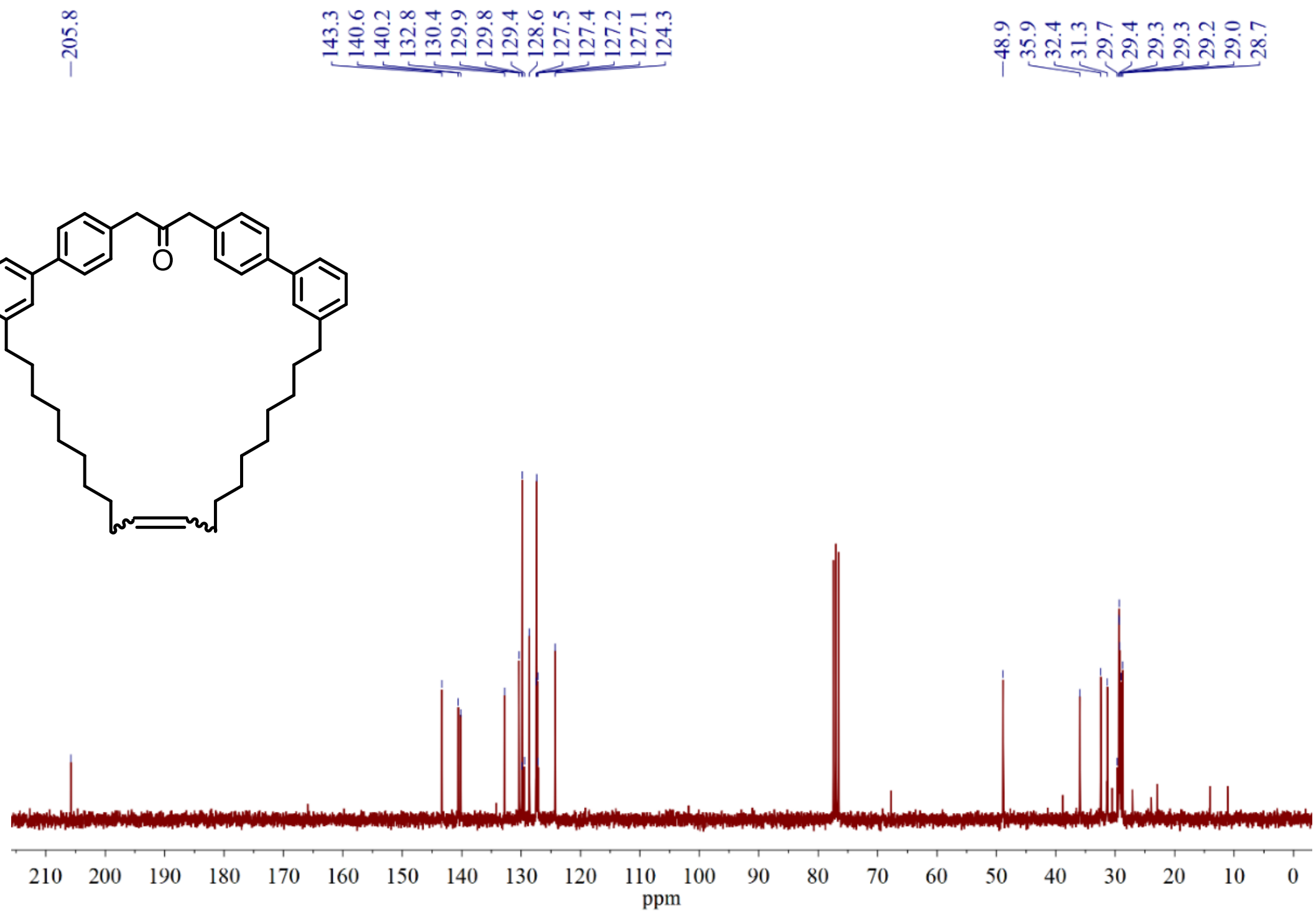

Supplementary Figure 42. ^{13}C NMR spectrum (75 MHz) of **8a** in $CDCl_3$ at room temperature.

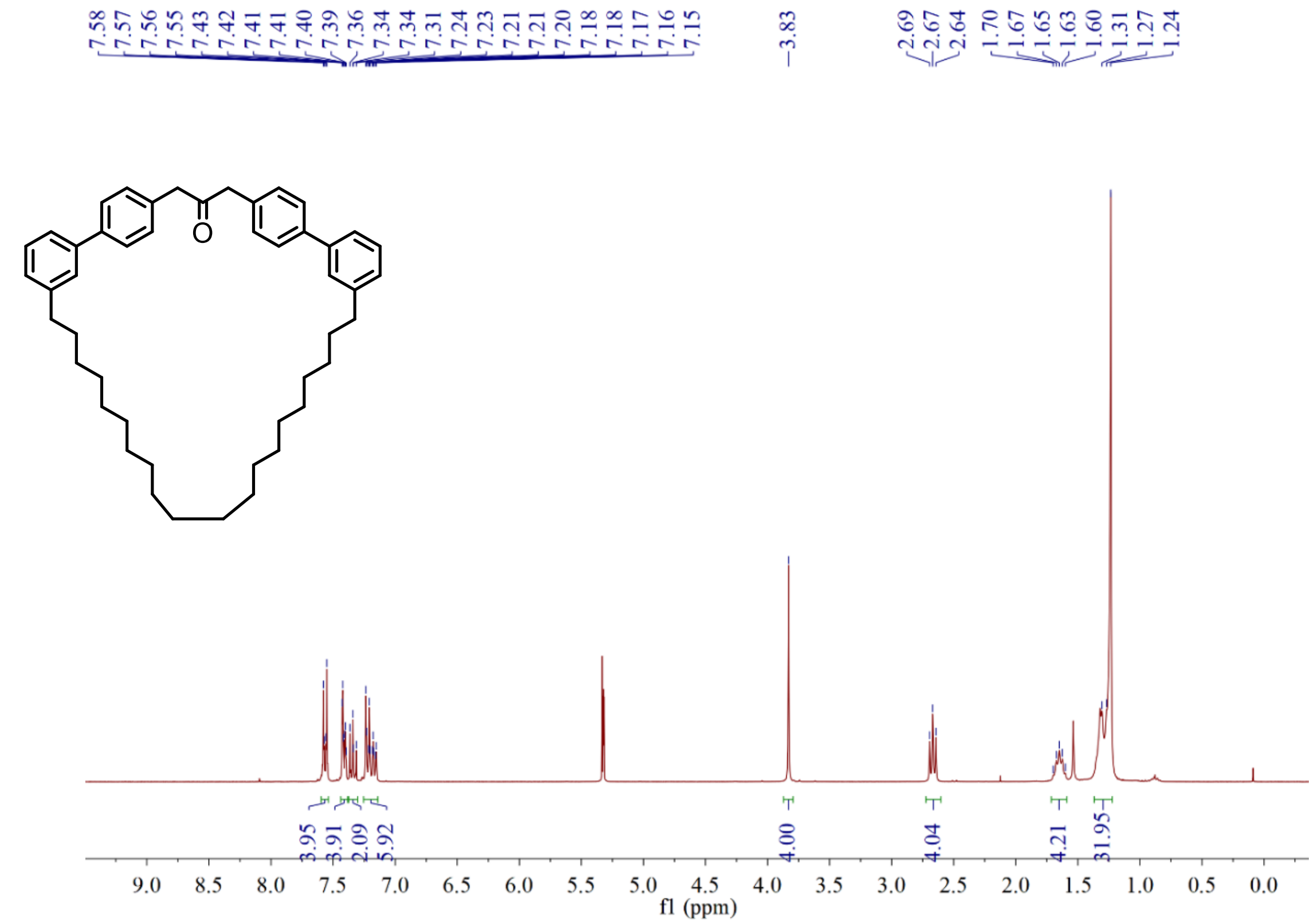

Supplementary Figure 43. ^{1}H NMR spectrum (300 MHz) of **7a** in CD_2Cl_2 at room temperature.

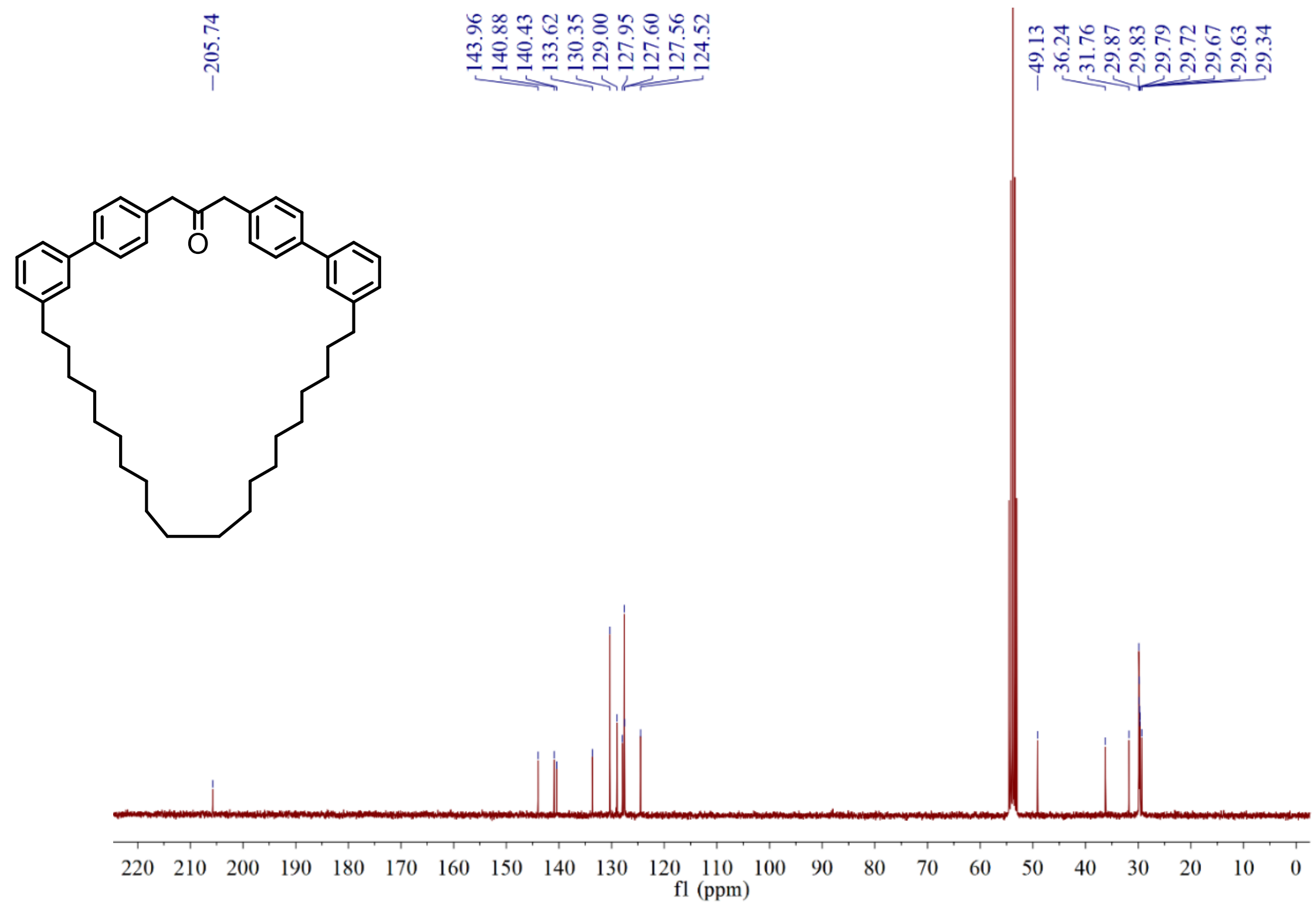

Supplementary Figure 44. ^{13}C NMR spectrum (75 MHz) of **7a** in CD_2Cl_2 at room temperature.

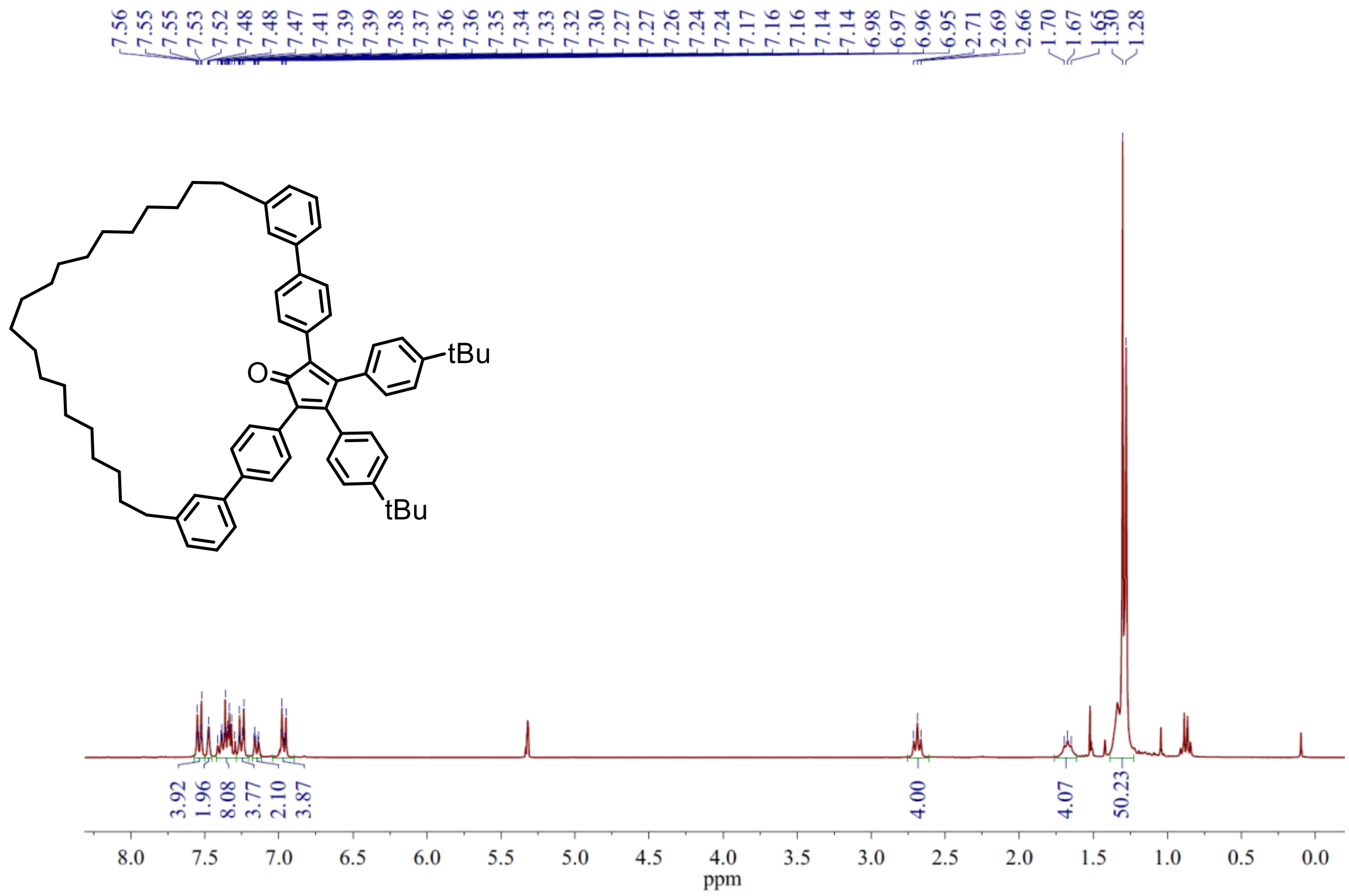

Supplementary Figure 45. ^{1}H NMR spectrum (300 MHz) of **6a** in CD_2Cl_2 at room temperature.

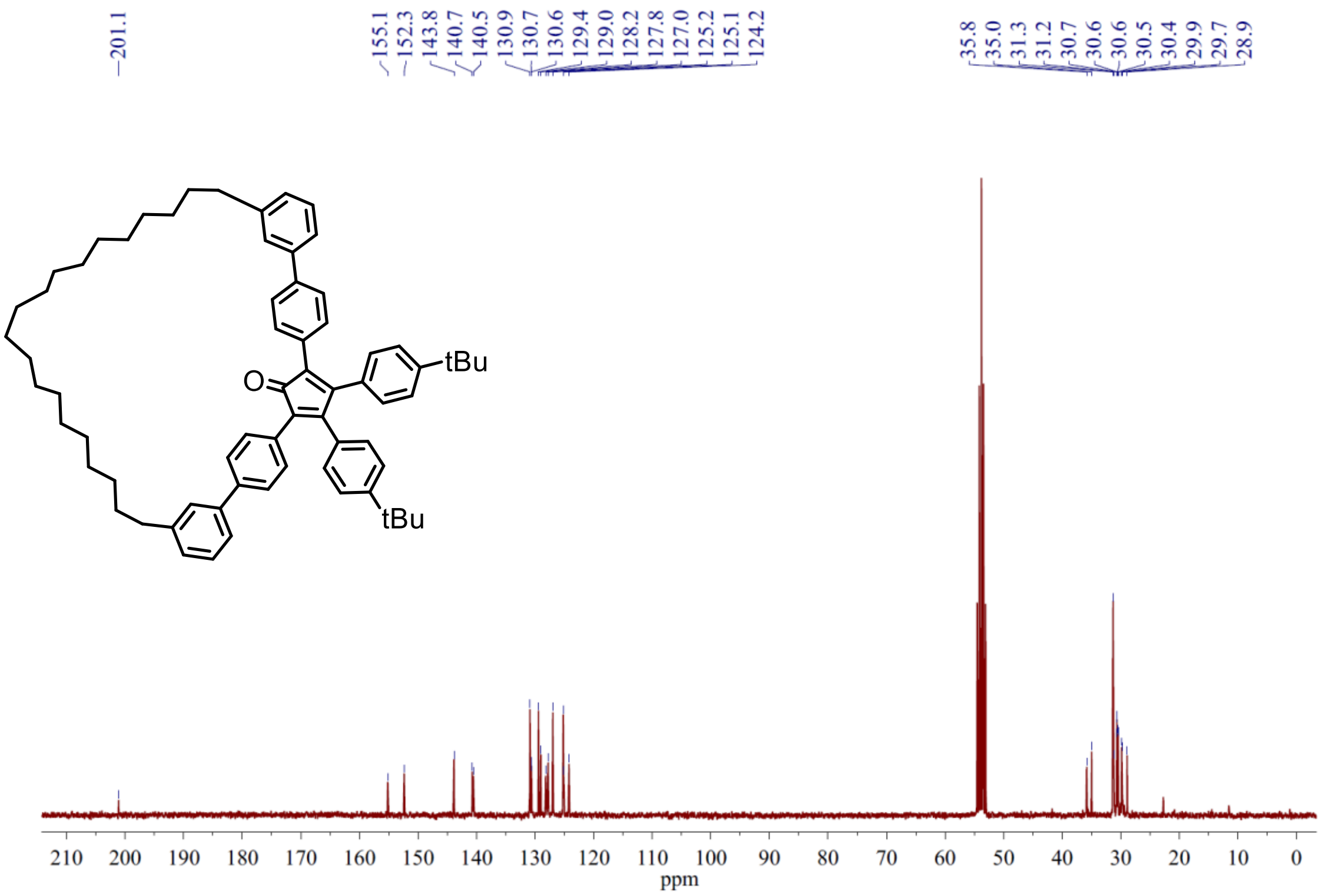

Supplementary Figure 46. ^{13}C NMR spectrum (75 MHz) of **6a** in CD_2Cl_2 at room temperature.

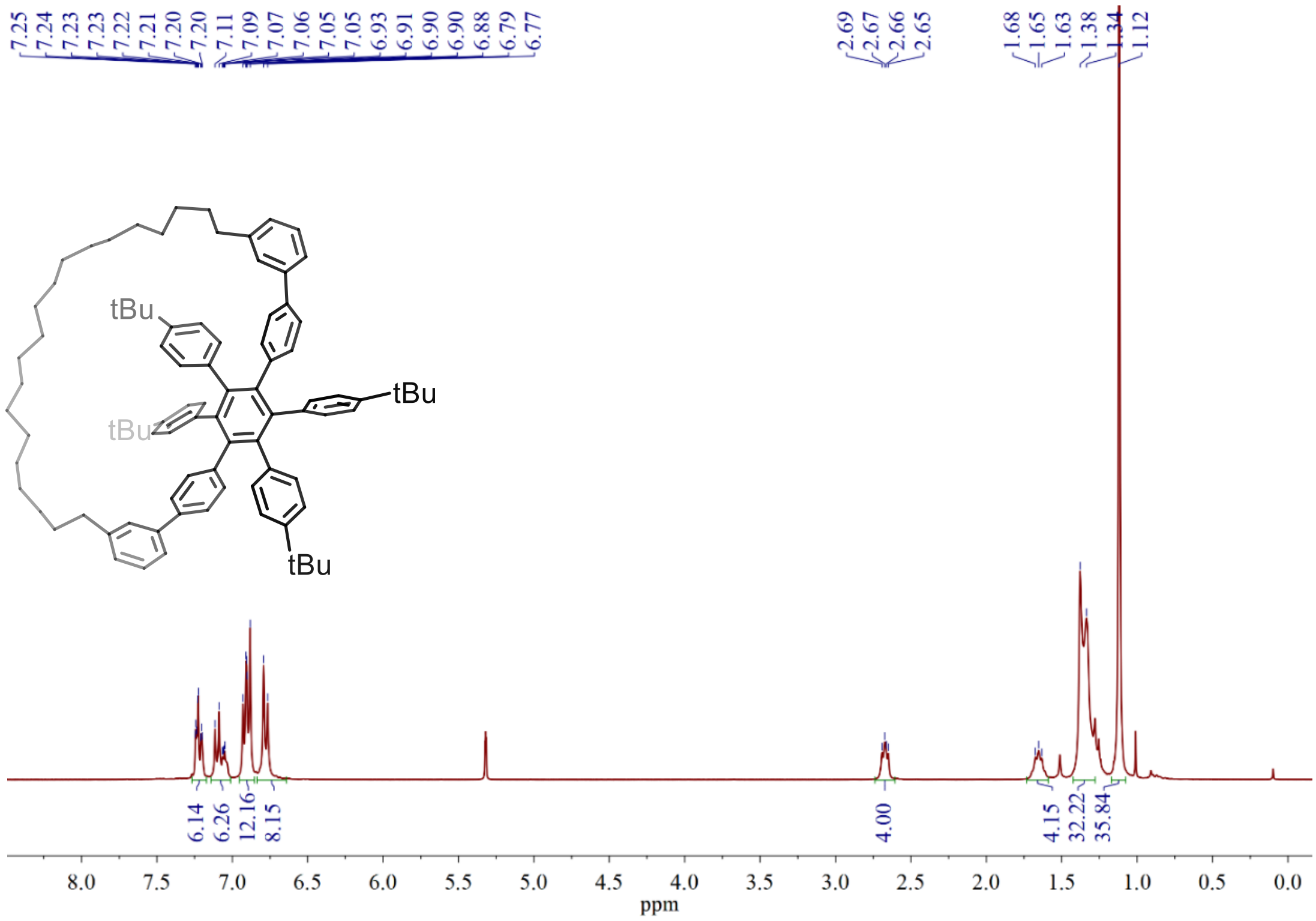

Supplementary Figure 47. ^{1}H NMR spectrum (300 MHz) of **3a** in CD_2Cl_2 at room temperature.

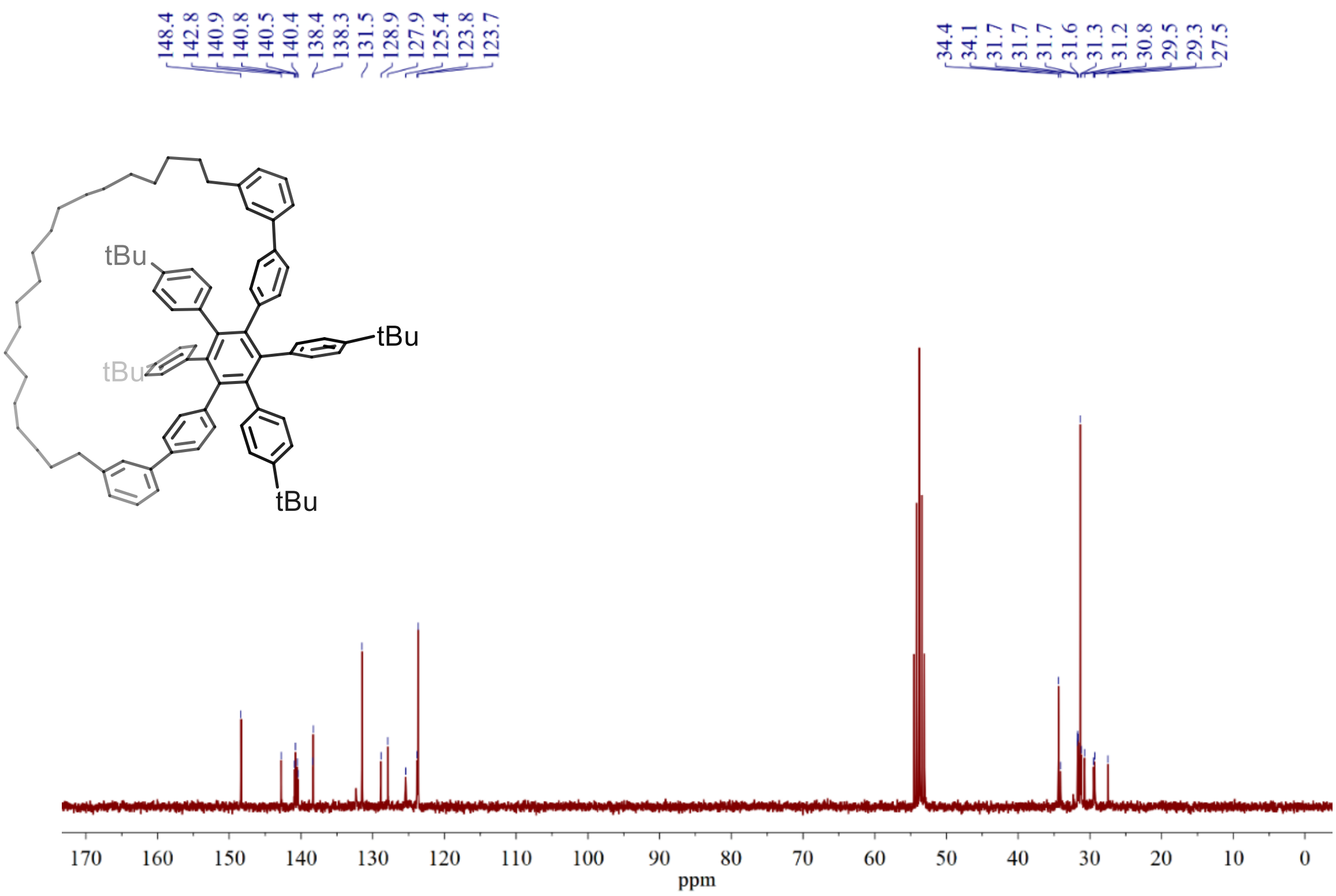

Supplementary Figure 48. ^{13}C NMR spectrum (75 MHz) of **3a** in CD_2Cl_2 at room temperature.

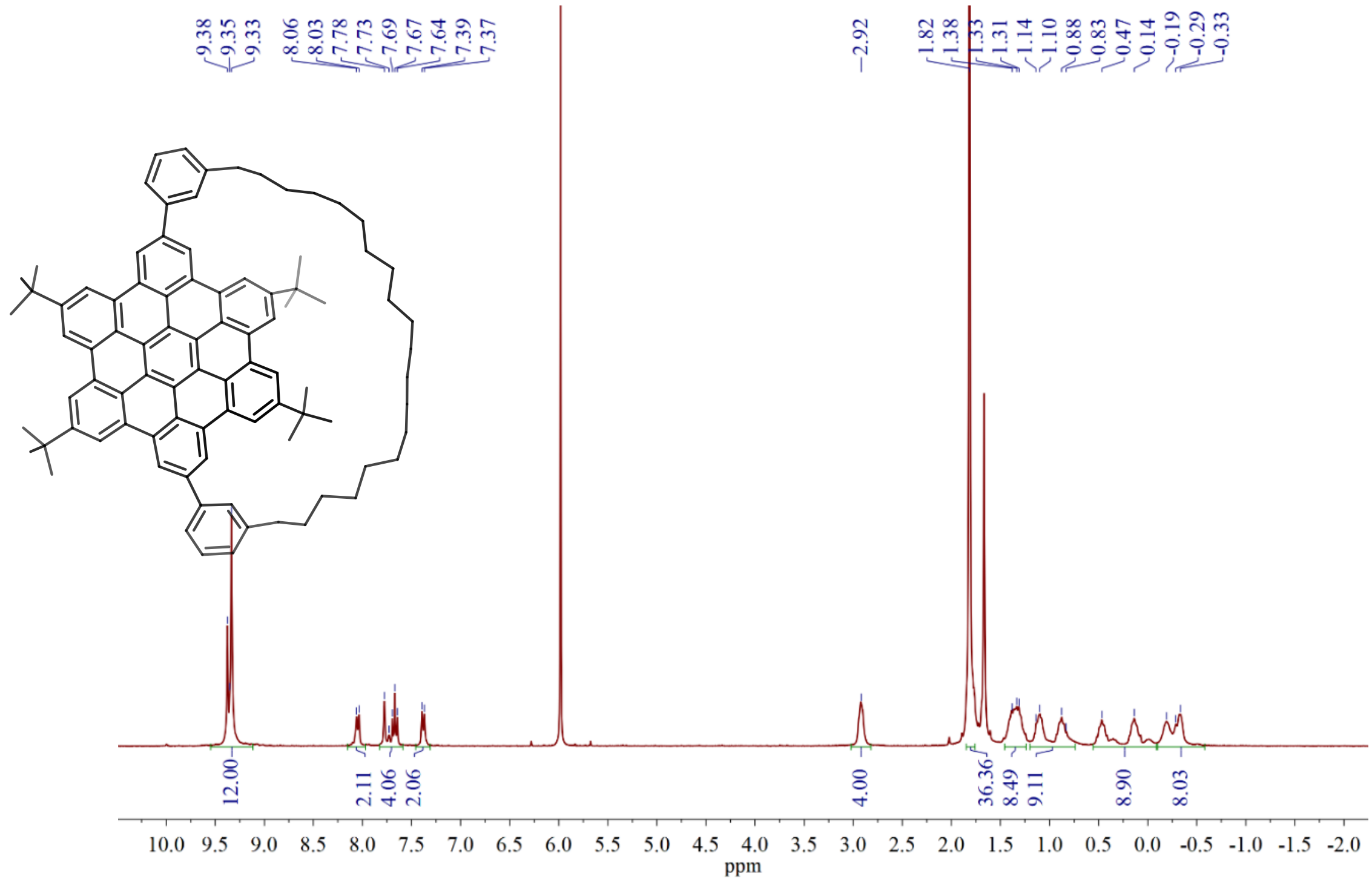

Supplementary Figure 49. ^{1}H NMR spectrum (300 MHz) of **2a** in $C_2D_2Cl_4$ at room temperature.

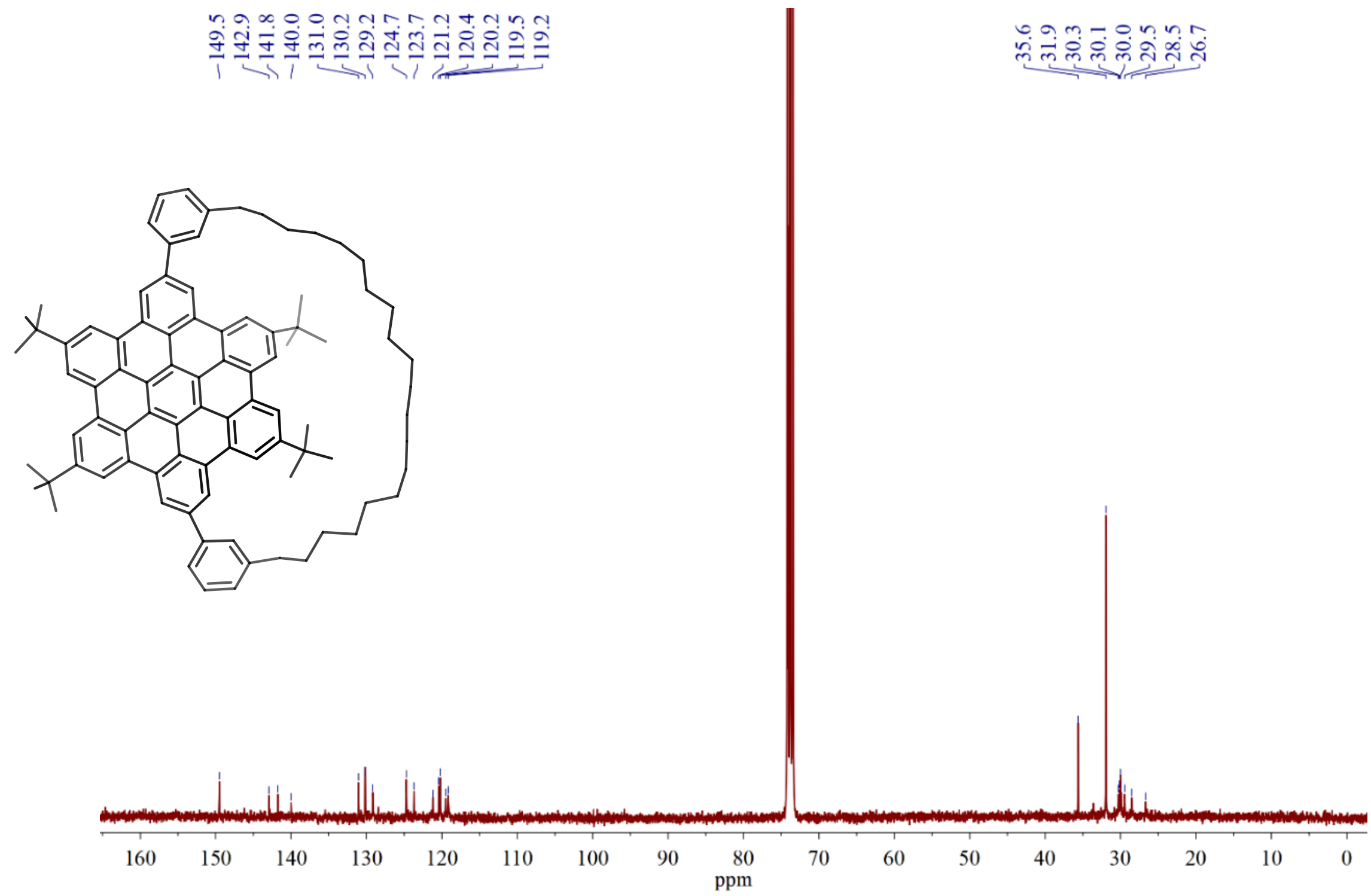

Supplementary Figure 50. ^{13}C NMR spectrum (75 MHz) of **2a** in $C_2D_2Cl_4$ at room temperature.

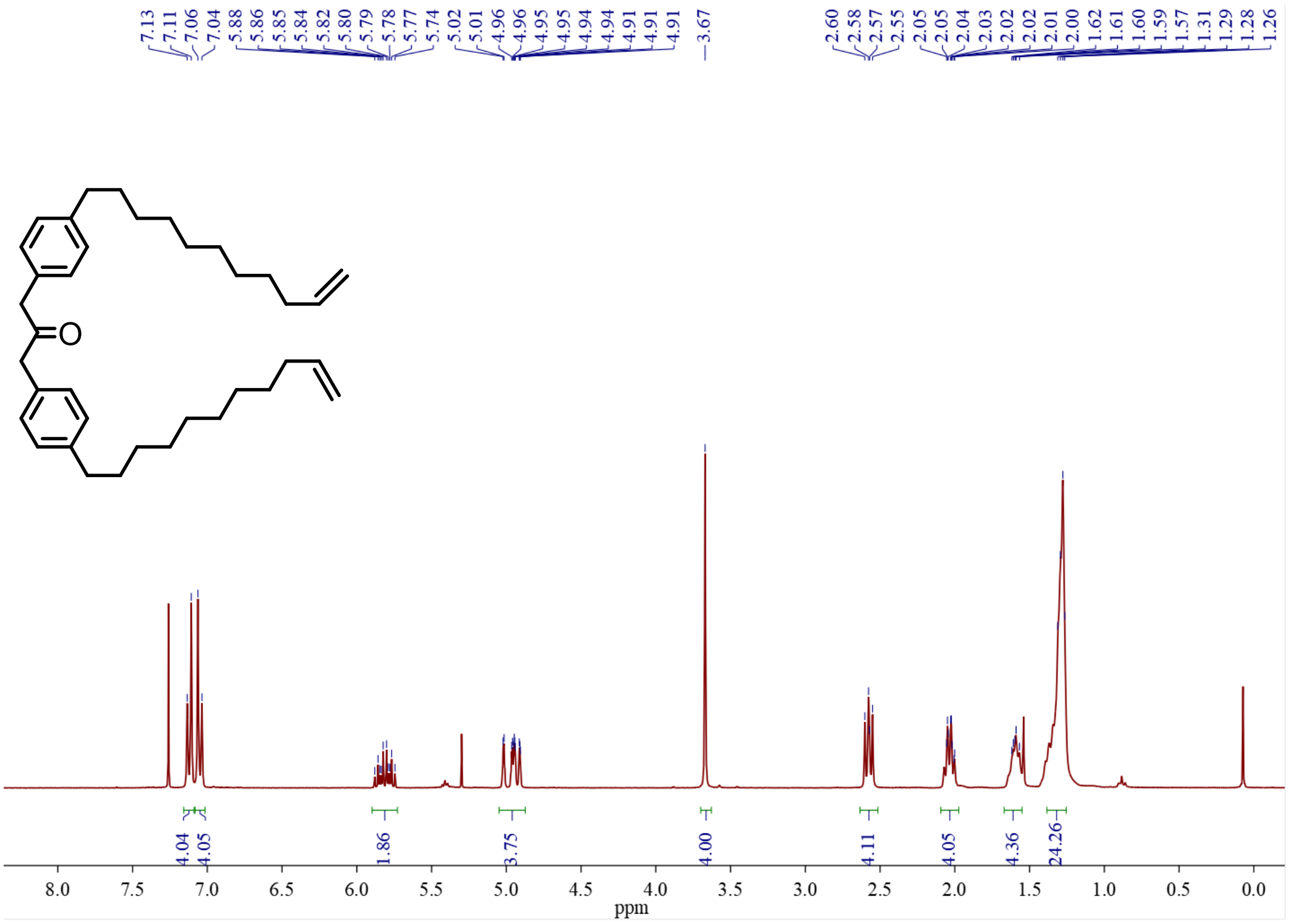

Supplementary Figure 51. ^{1}H NMR spectrum (300 MHz) of **9b** in $CDCl_3$ at room temperature.

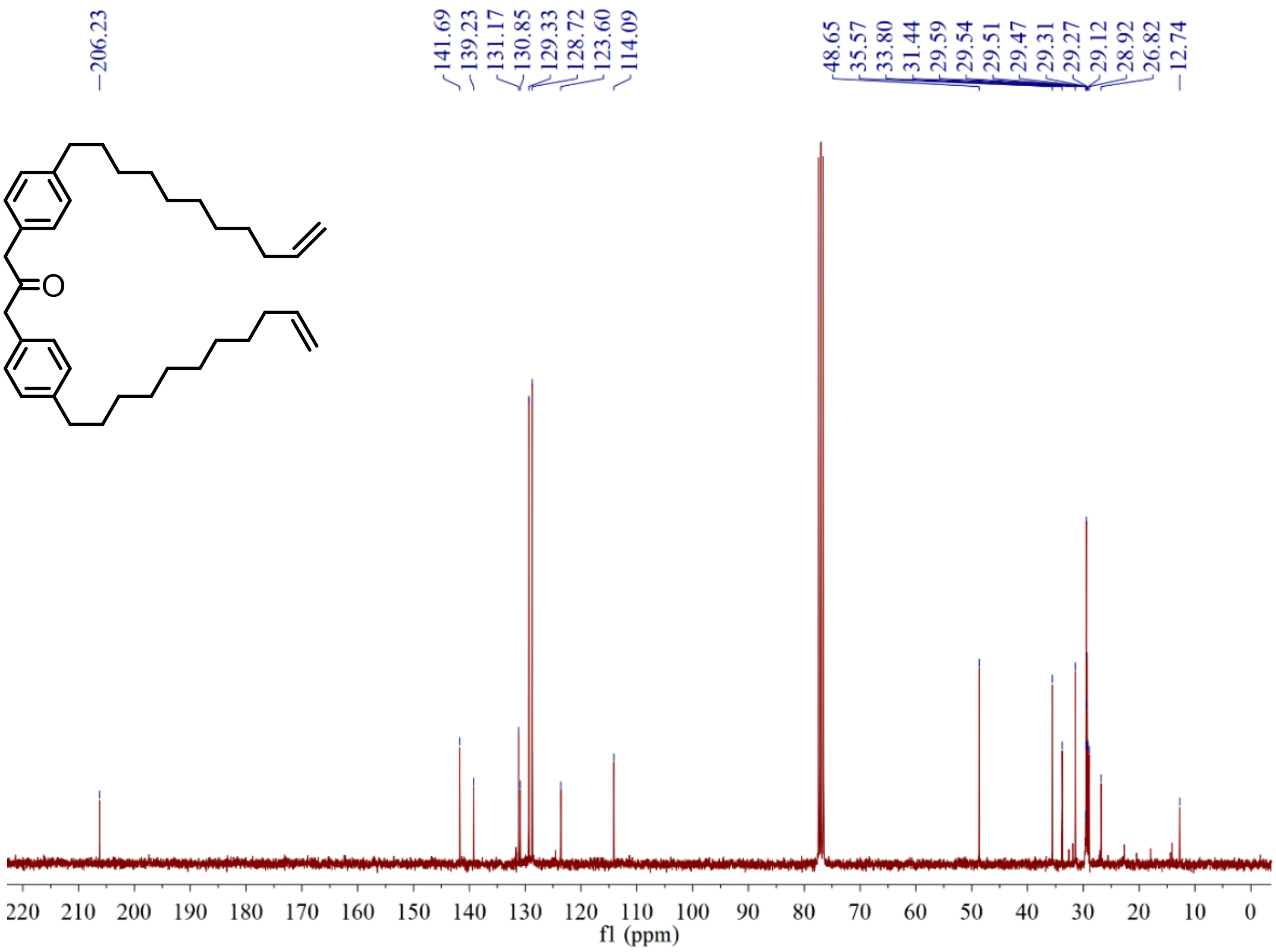

Supplementary Figure 52. ^{13}C NMR spectrum (75 MHz) of **9b** in $CDCl_3$ at room temperature.

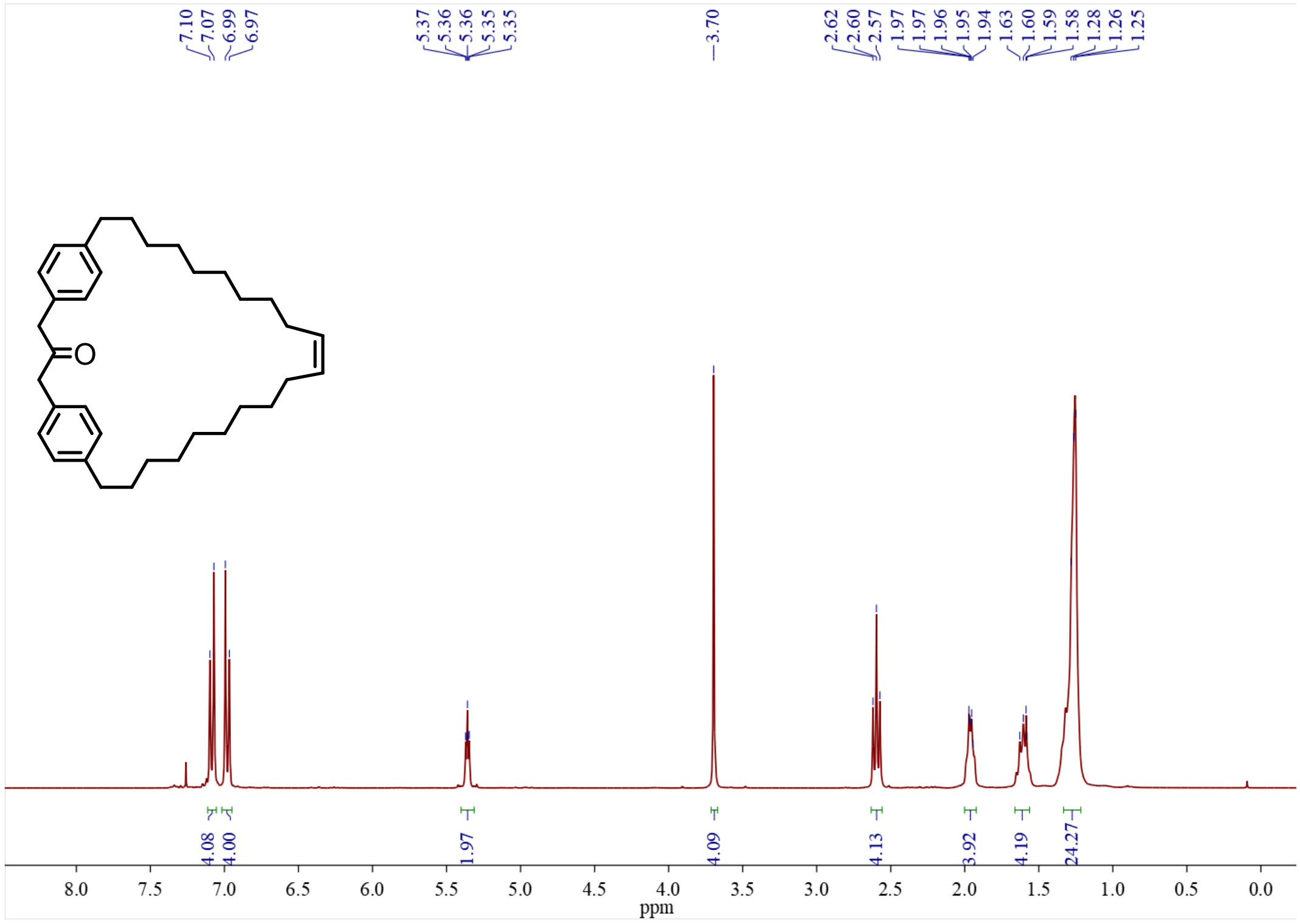

Supplementary Figure 53. ^{1}H NMR spectrum (300 MHz) of **8b** in $CDCl_3$ at room temperature.

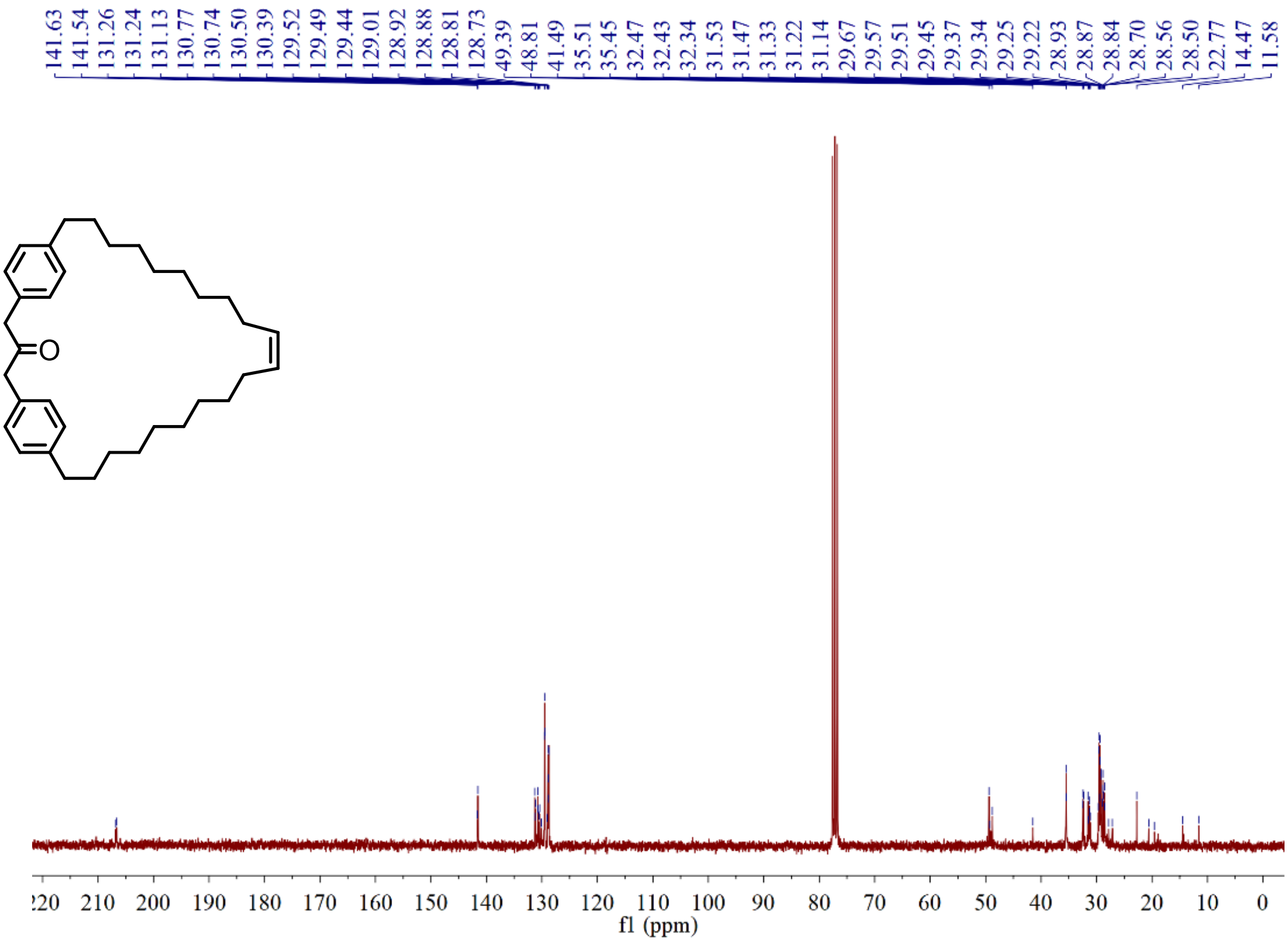

Supplementary Figure 54. ^{1}H NMR spectrum (300 MHz) of **8b** in $CDCl_3$ at room temperature.

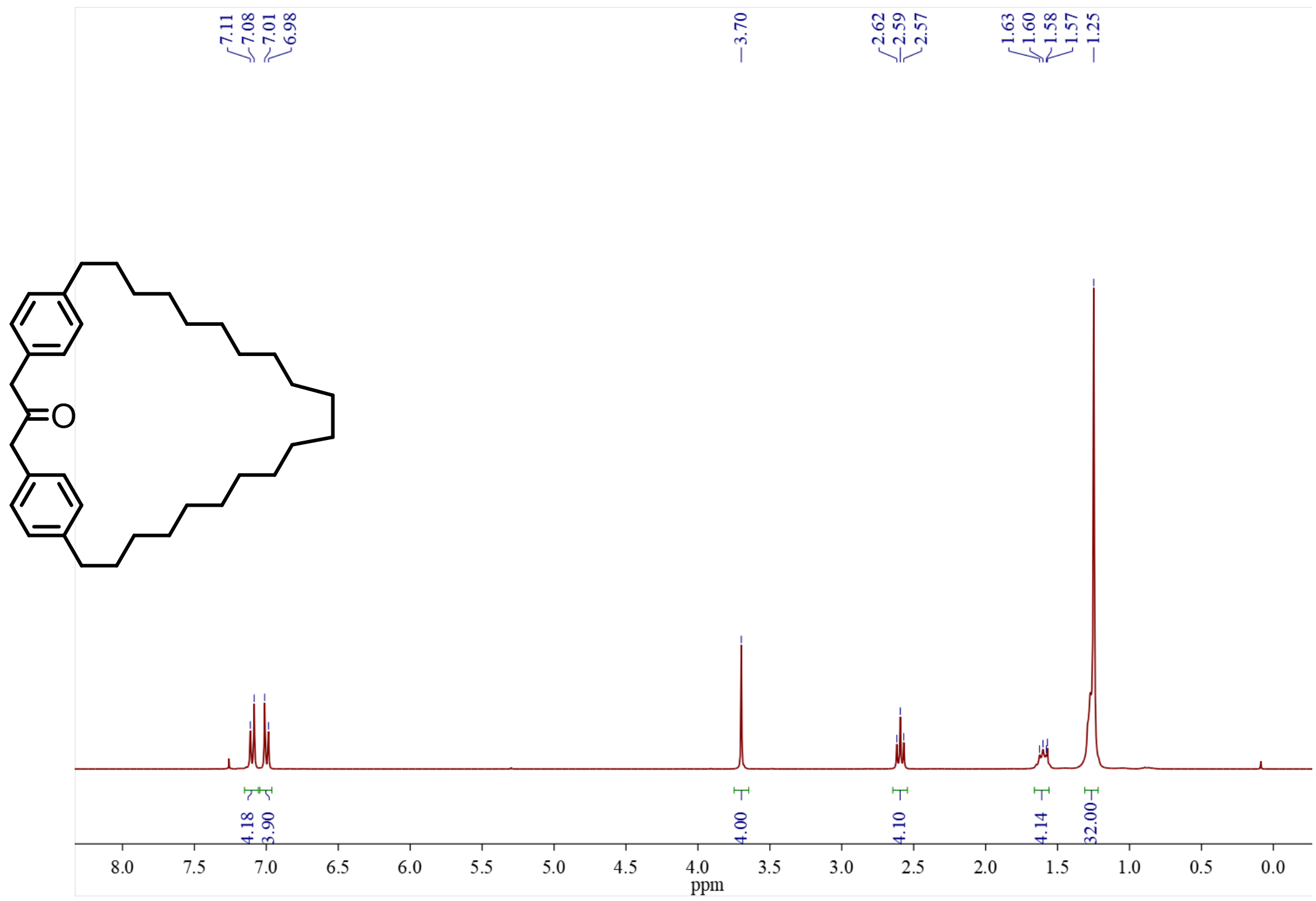

Supplementary Figure 55. ^{1}H NMR spectrum (300 MHz) of **7b** in $CDCl_3$ at room temperature.

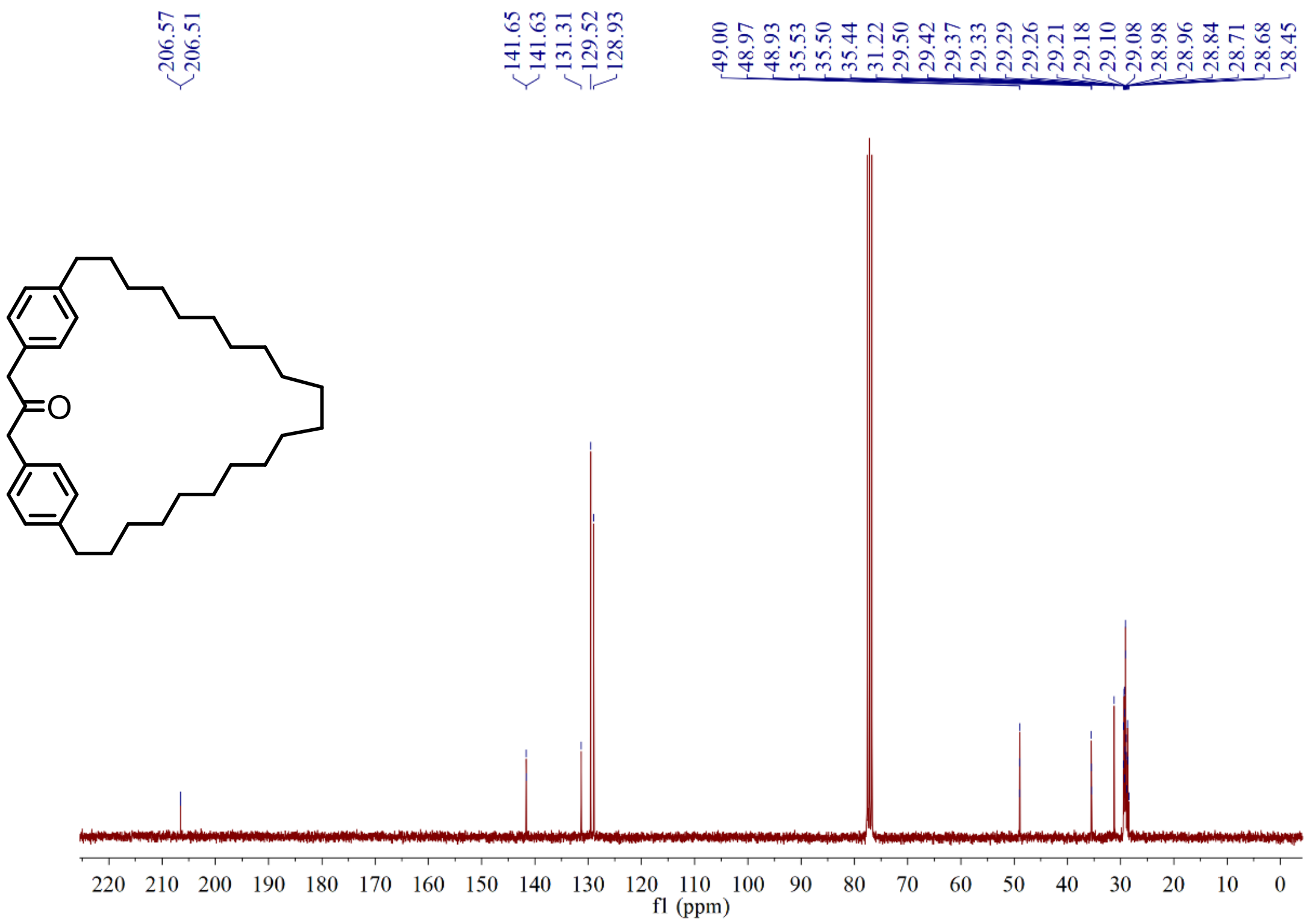

Supplementary Figure 56. ^{1}H NMR spectrum (300 MHz) of **7b** in $CDCl_3$ at room temperature.

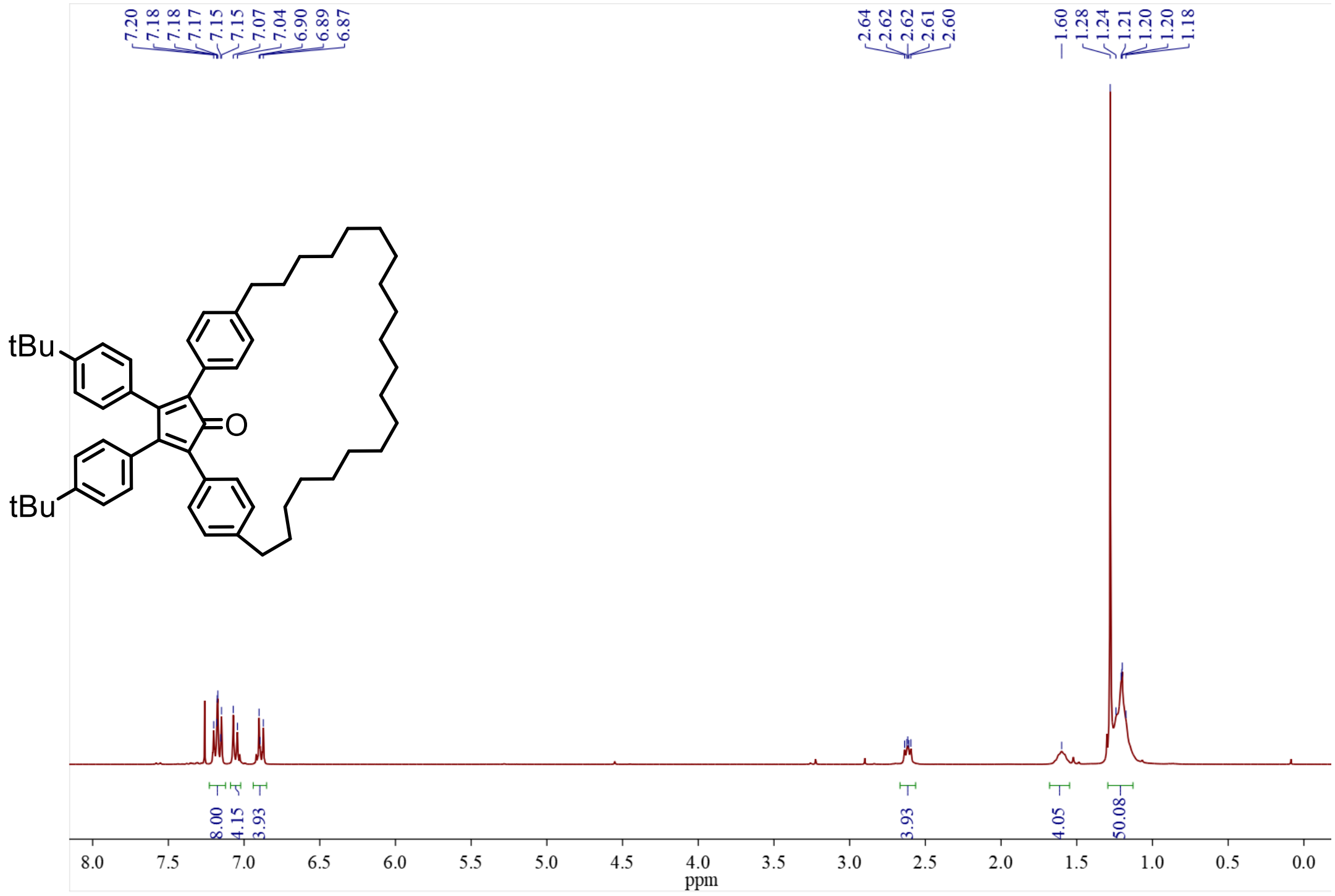

Supplementary Figure 57. ^{1}H NMR spectrum (300 MHz) of **6b** in $CDCl_3$ at room temperature.

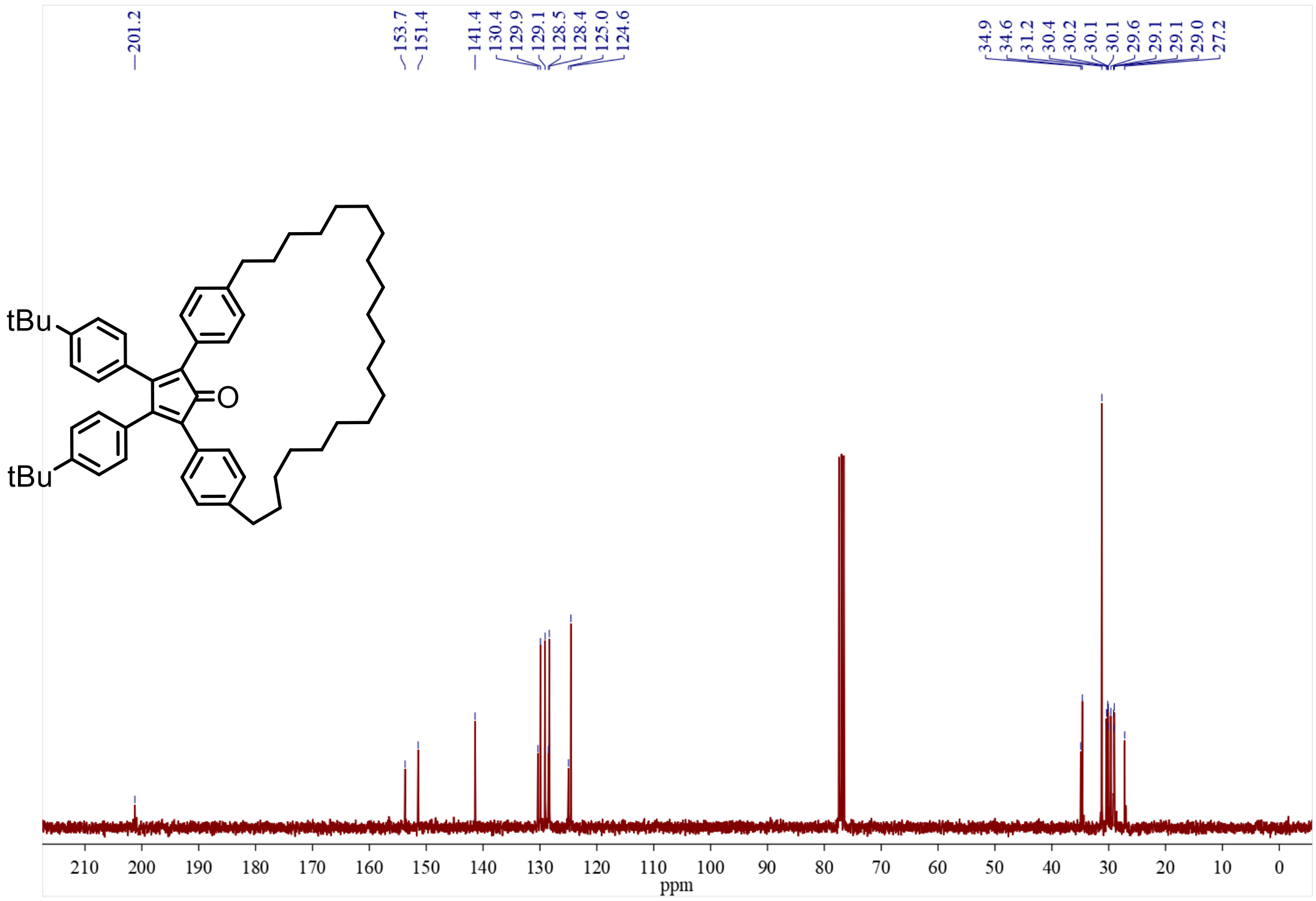

Supplementary Figure 58. ^{13}C NMR spectrum (75 MHz) of **6b** in $CDCl_3$ at room temperature.

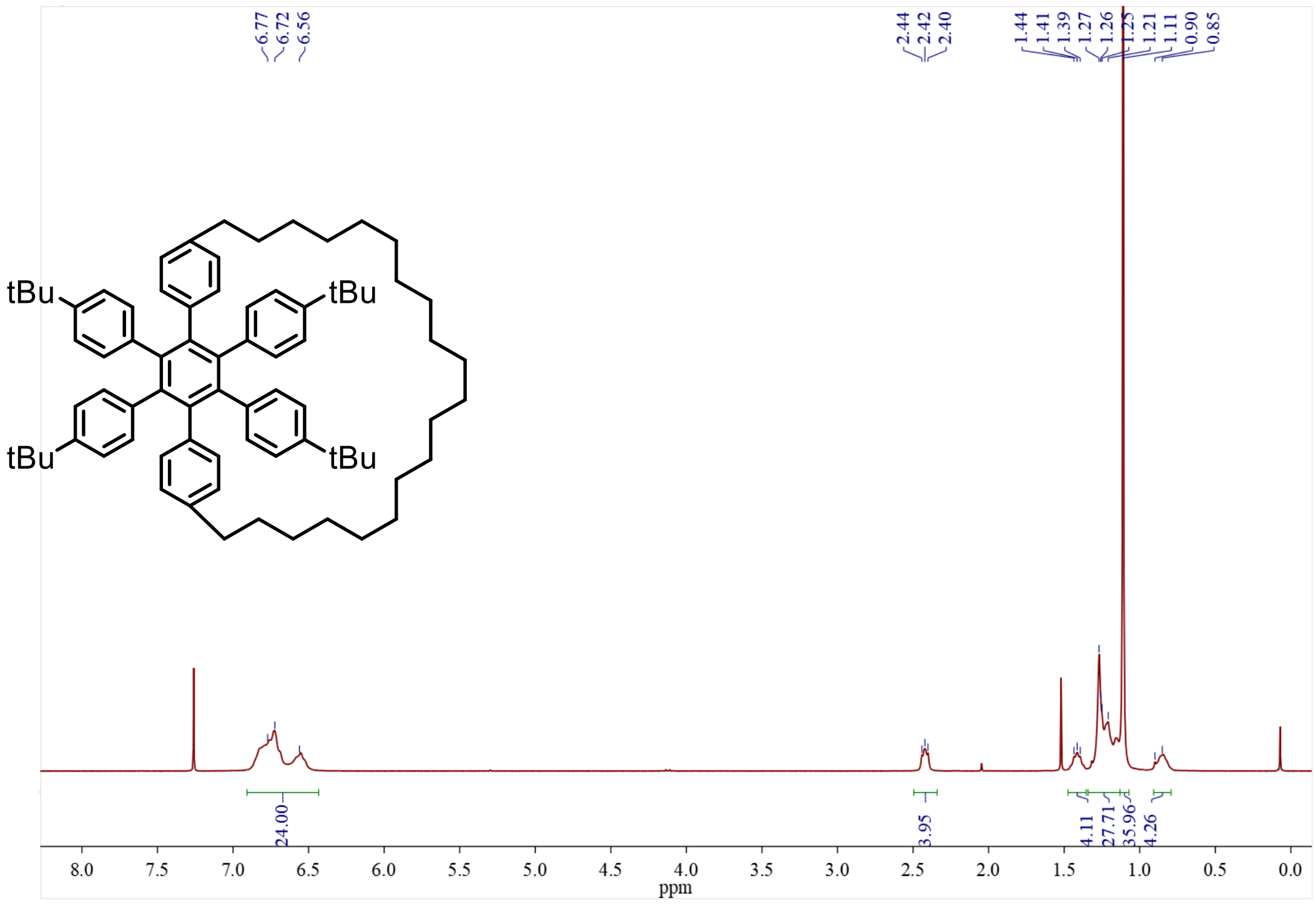

Supplementary Figure 59. ^{1}H NMR spectrum (300 MHz) of **3b** in $CDCl_3$ at room temperature.

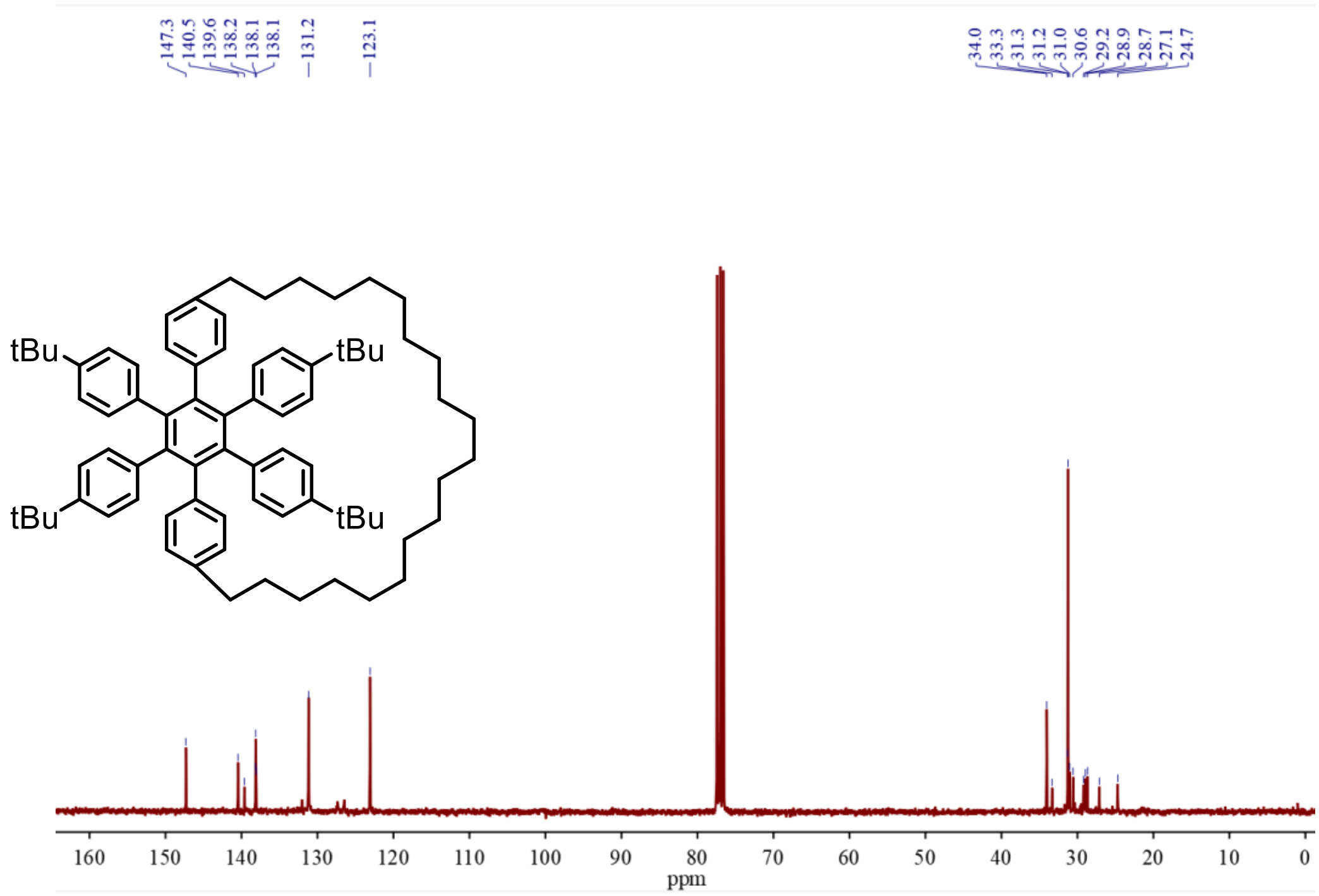

Supplementary Figure 60. ^{13}C NMR spectrum (75 MHz) of **3b** in $CDCl_3$ at room temperature.

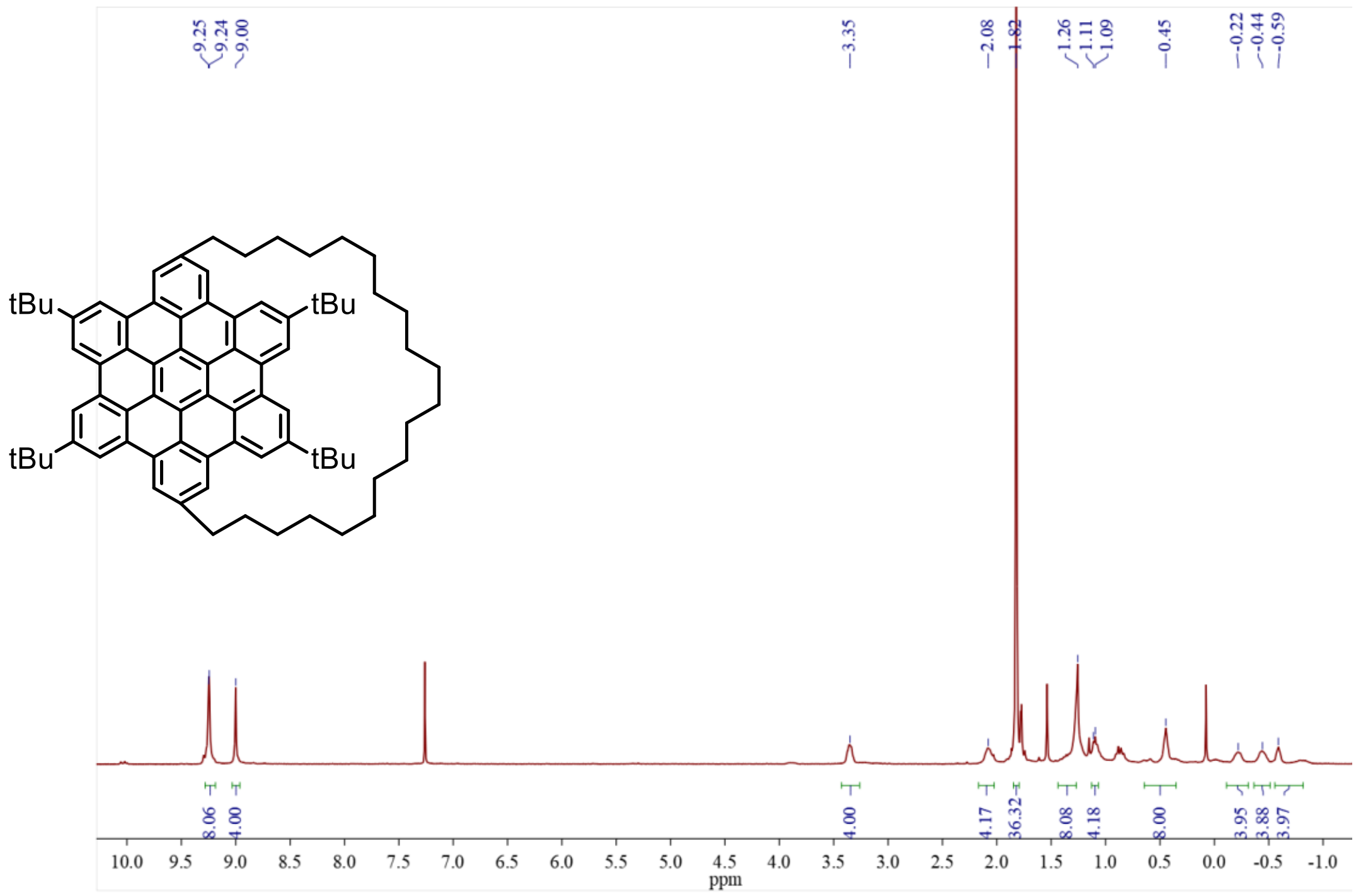

Supplementary Figure 61. ^{1}H NMR spectrum (300 MHz) of **2b** in $CDCl_3$ at room temperature.

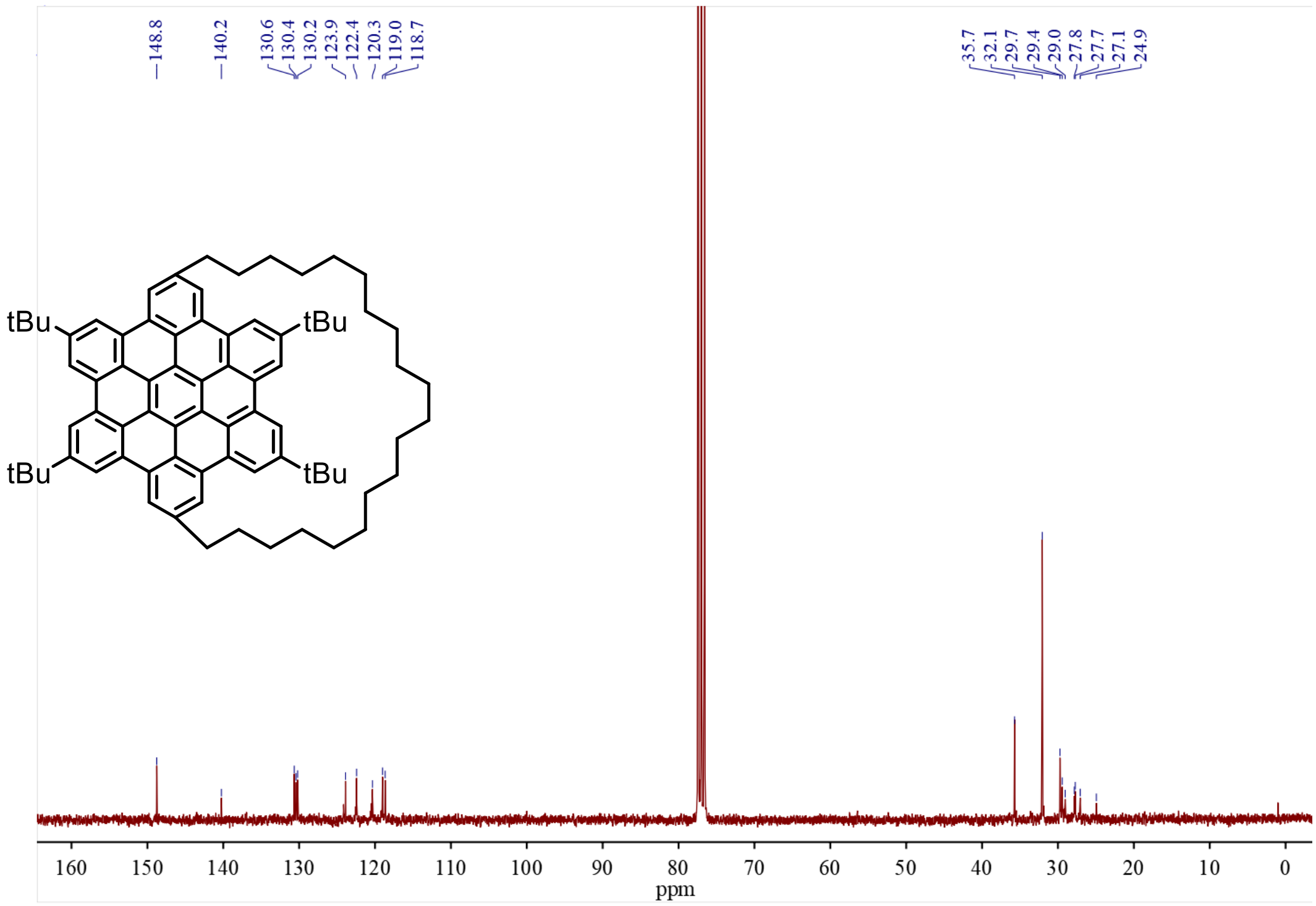

Supplementary Figure 62. ^{13}C NMR spectrum (75 MHz) of **2b** in $CDCl_3$ at room temperature.

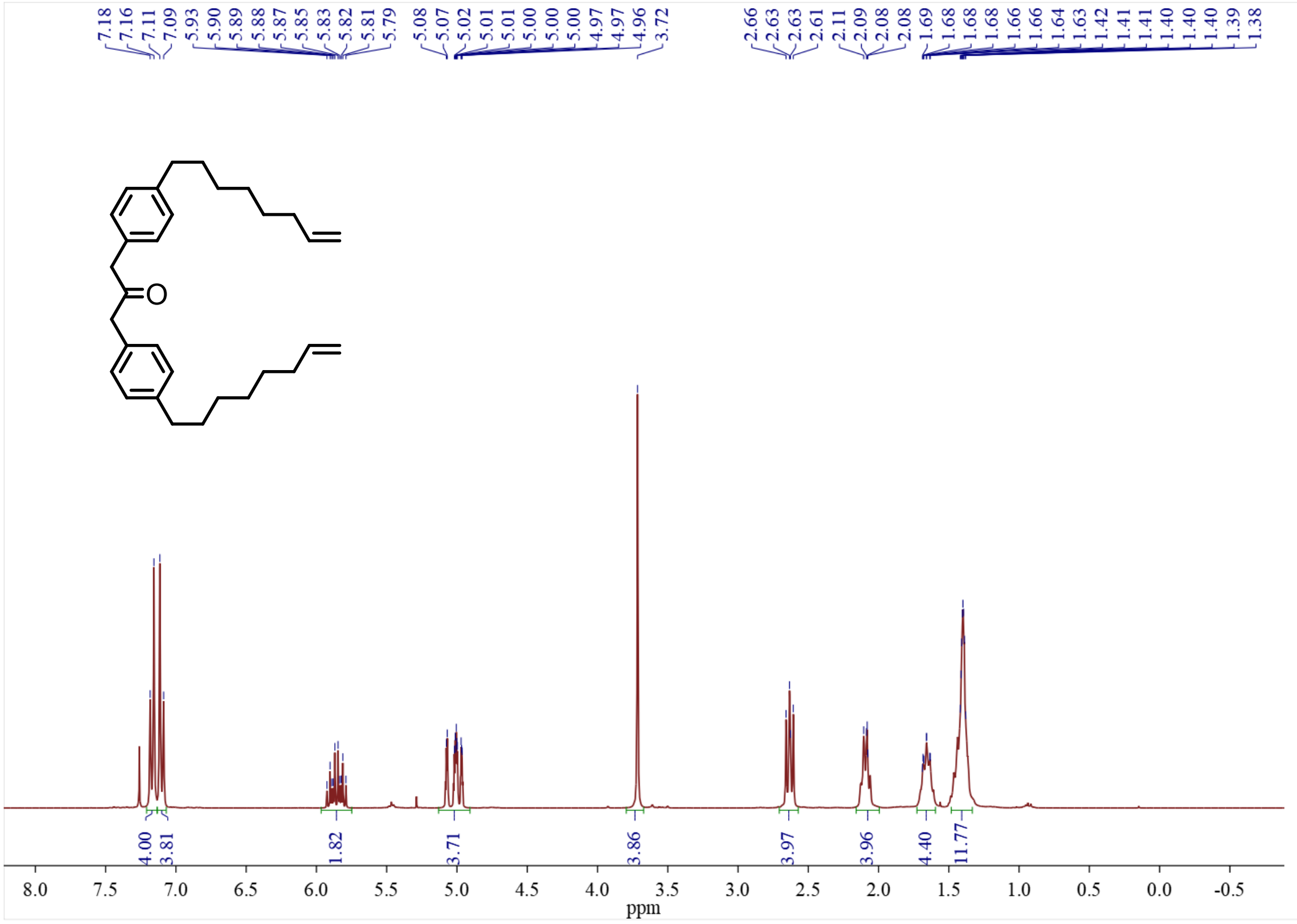

Supplementary Figure 63. ^{1}H NMR spectrum (300 MHz) of **9c** in $CDCl_3$ at room temperature.

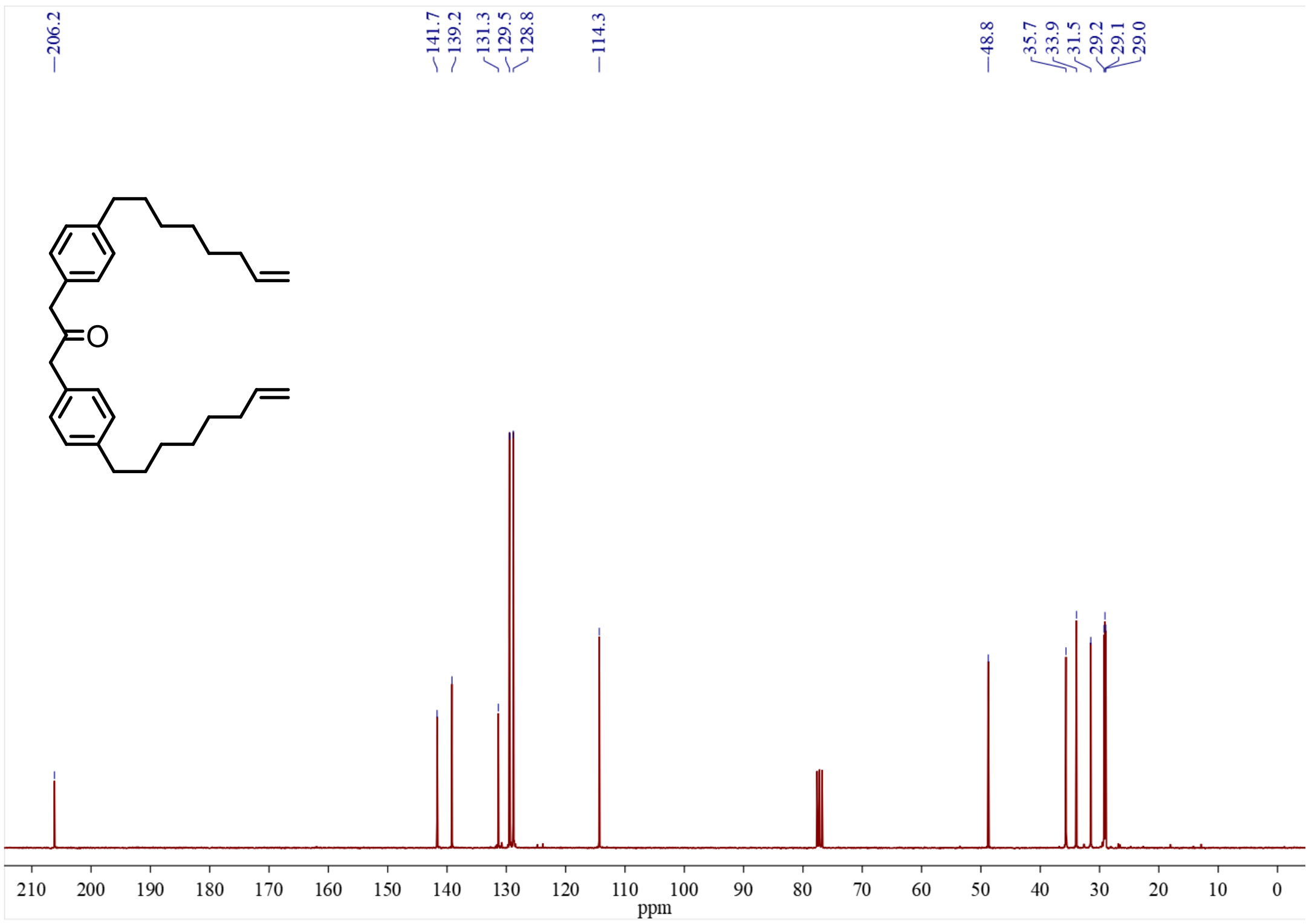

Supplementary Figure 64. ^{13}C NMR spectrum (75 MHz) of **9c** in $CDCl_3$ at room temperature.

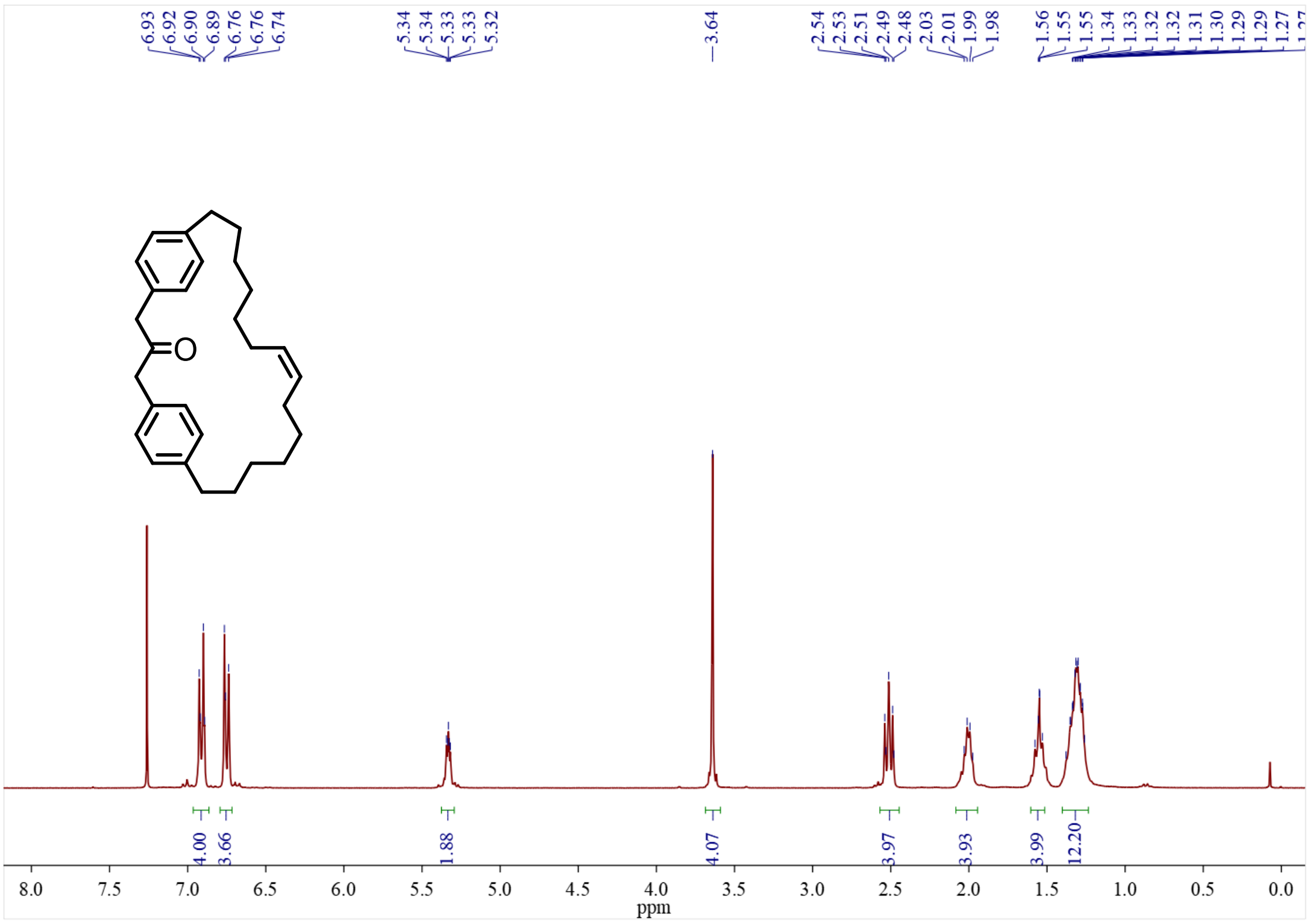

Supplementary Figure 65. ^{1}H NMR spectrum (300 MHz) of **8c** in $CDCl_3$ at room temperature.

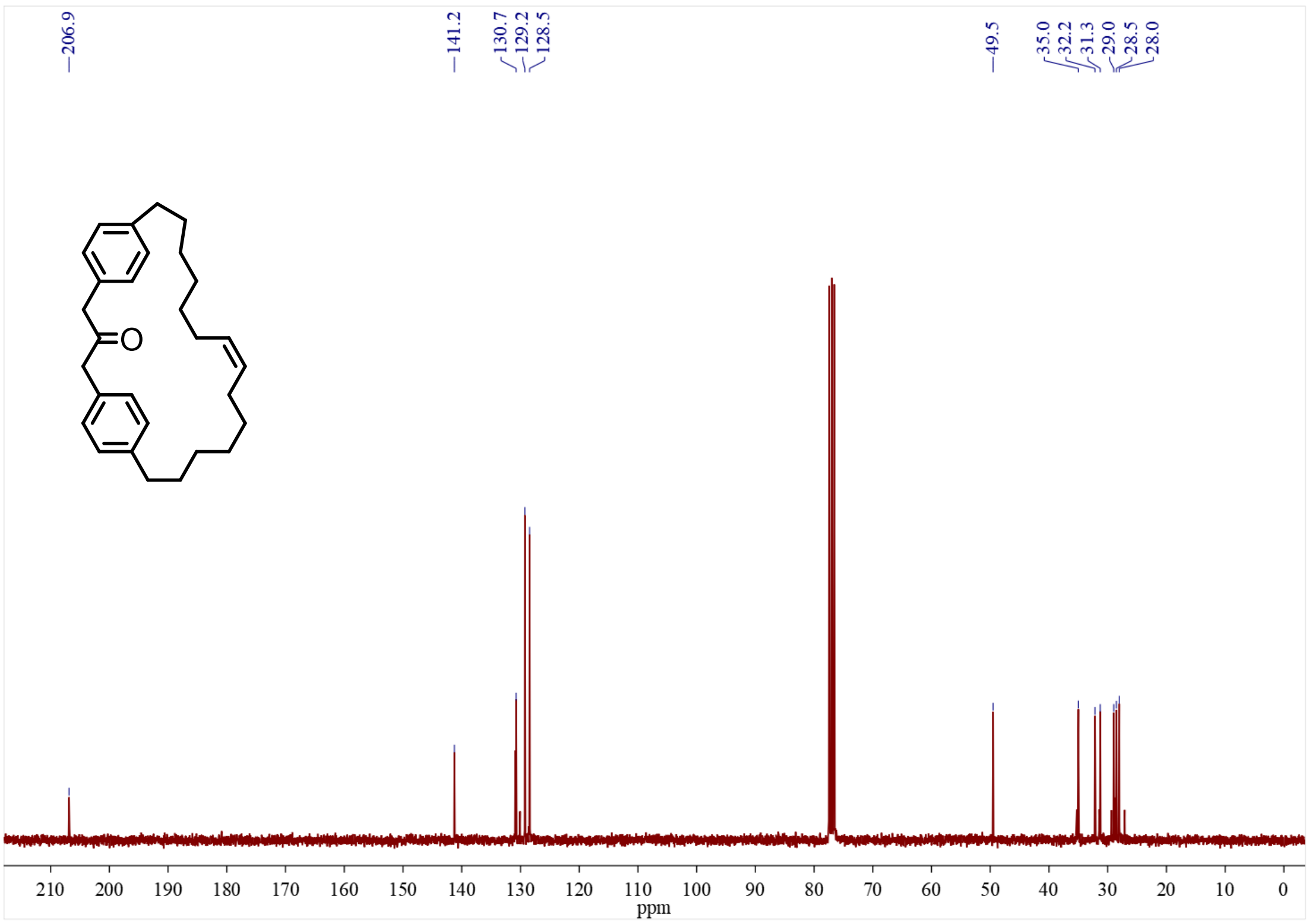

Supplementary Figure 66. ^{13}C NMR spectrum (75 MHz) of **8c** in $CDCl_3$ at room temperature.

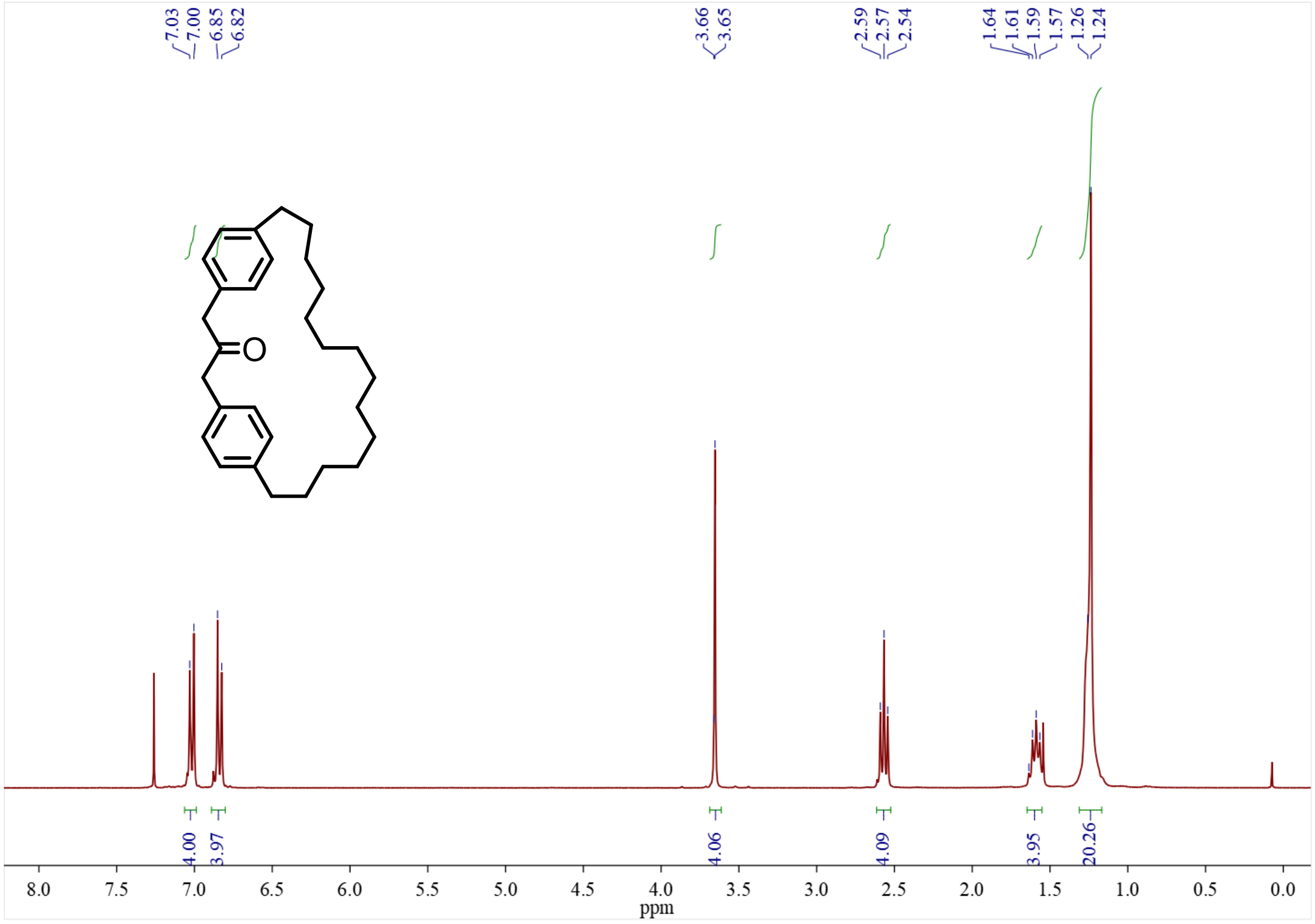

Supplementary Figure 67. ^{1}H NMR spectrum (300 MHz) of **7c** in $CDCl_3$ at room temperature.

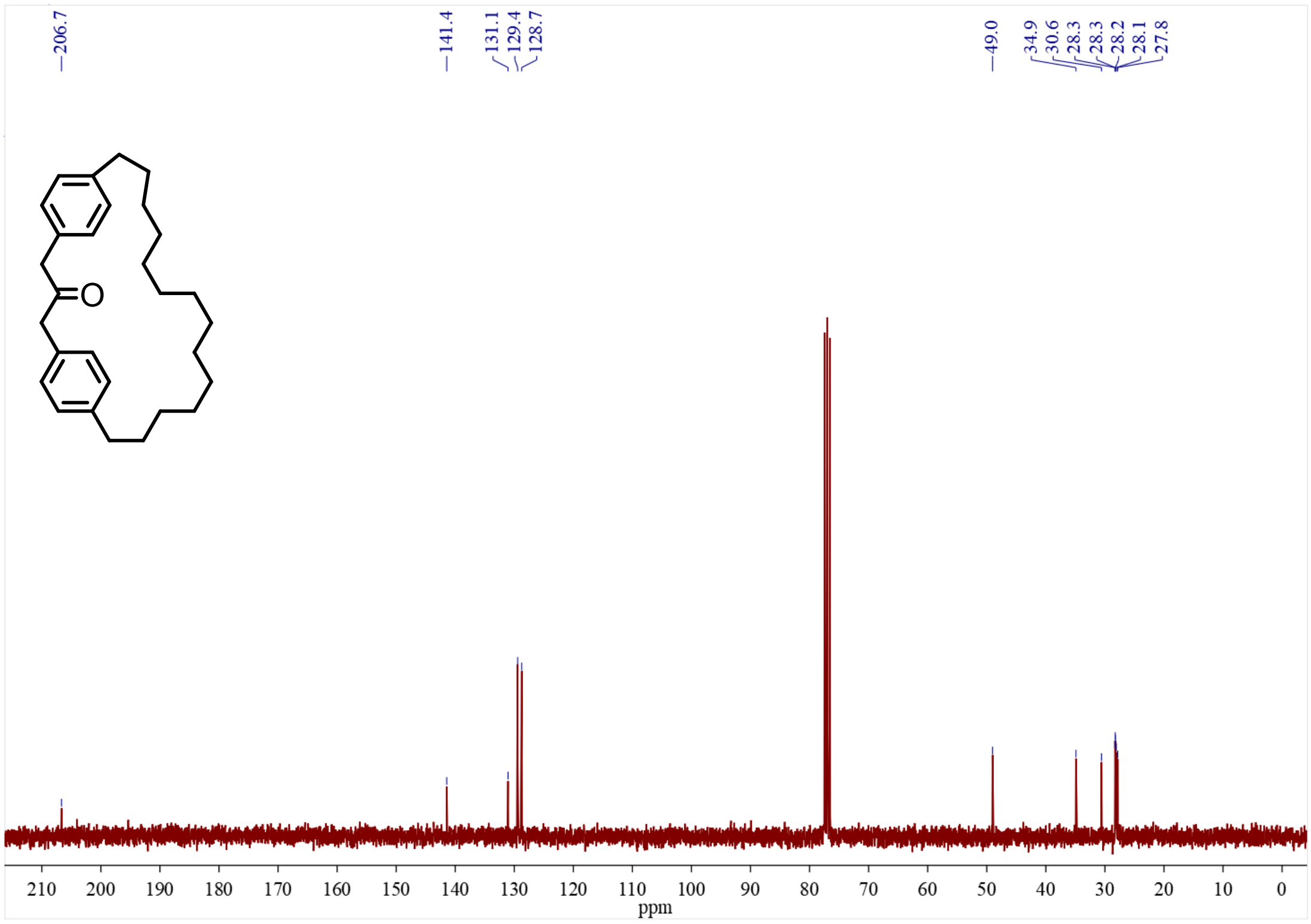

Supplementary Figure 68. ^{13}C NMR spectrum (75 MHz) of **7c** in $CDCl_3$ at room temperature.

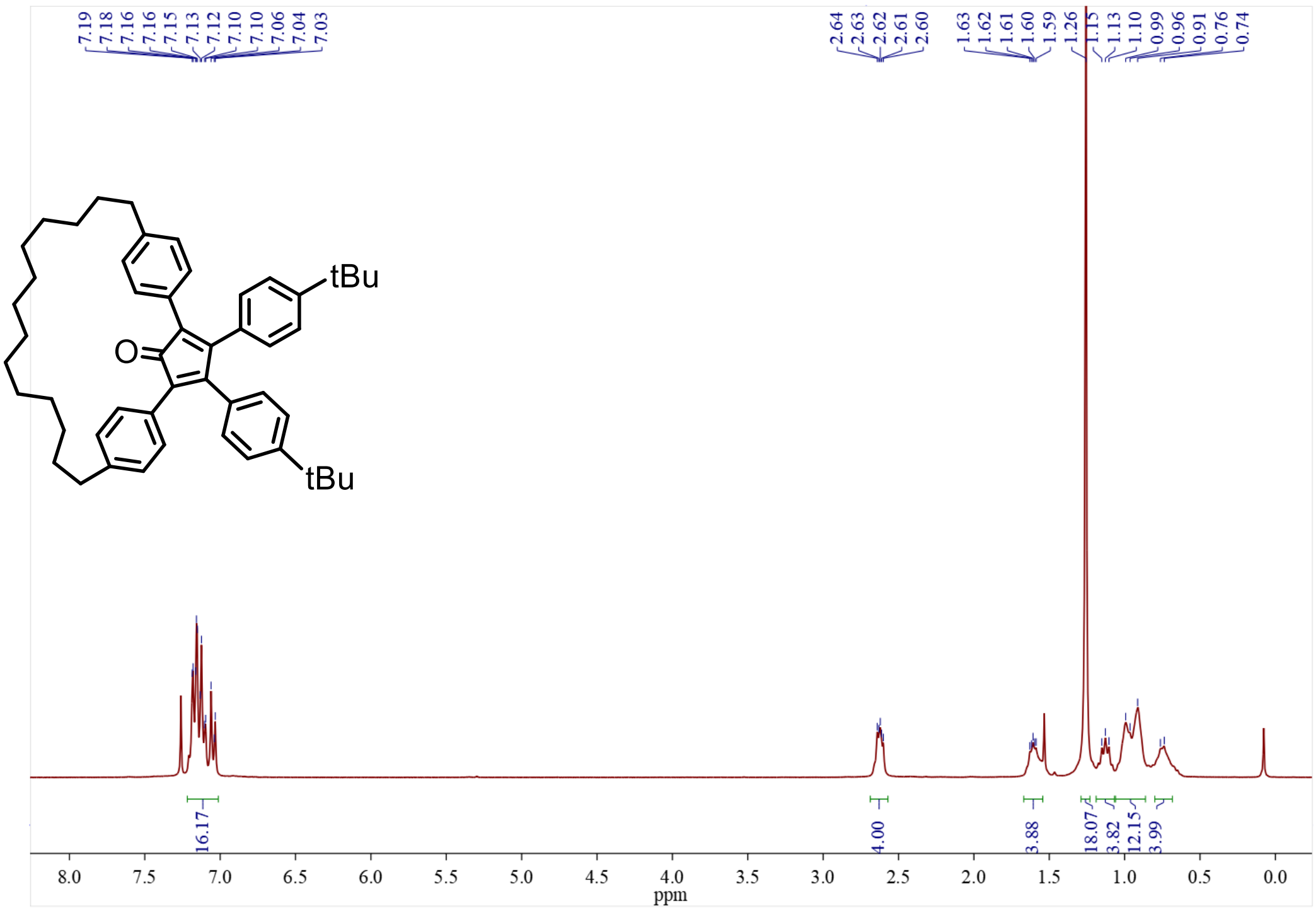

Supplementary Figure 69. ^{1}H NMR spectrum (300 MHz) of **6c** in $CDCl_3$ at room temperature.

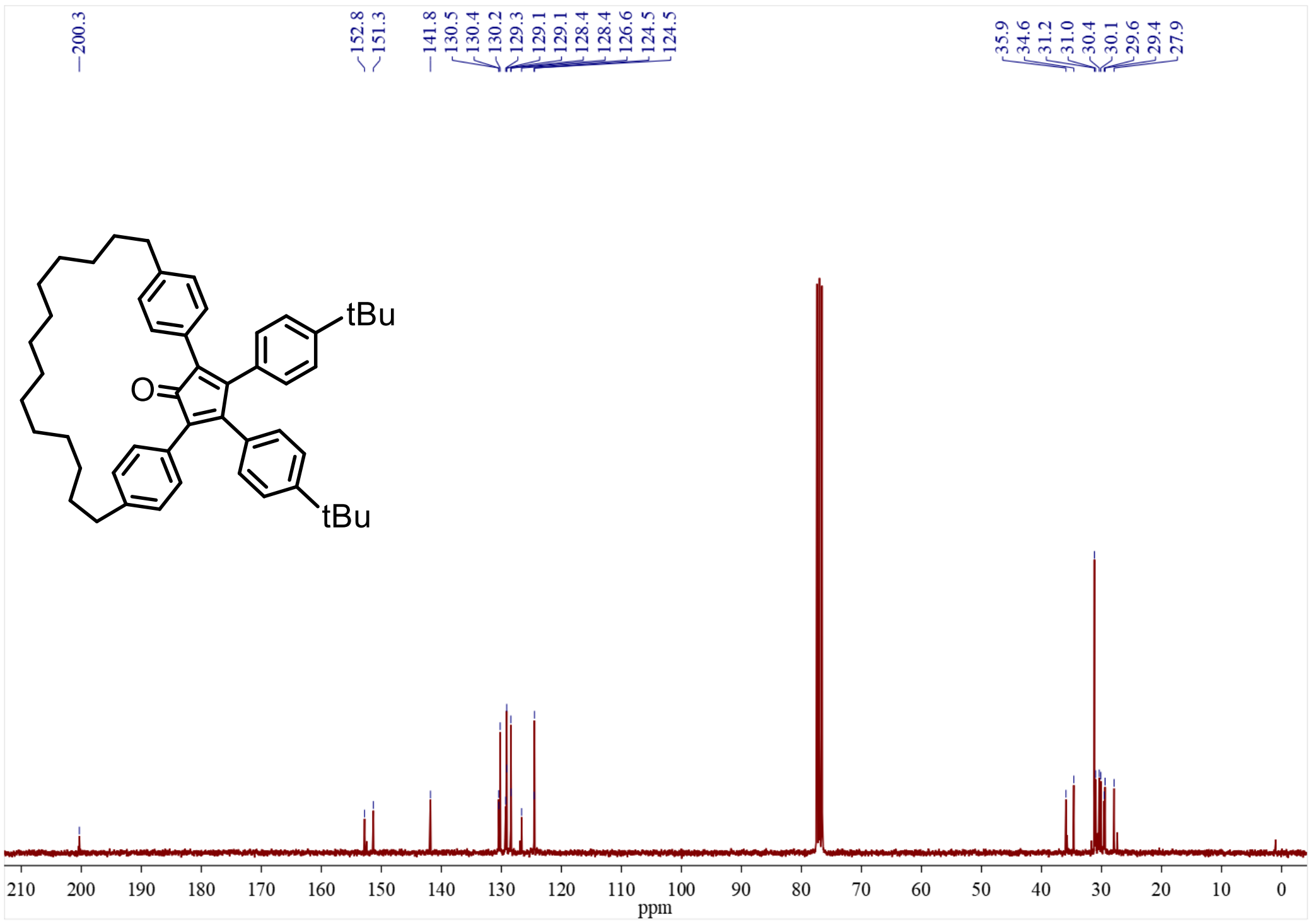

Supplementary Figure 70. ^{13}C NMR spectrum (75 MHz) of **6c** in $CDCl_3$ at room temperature.

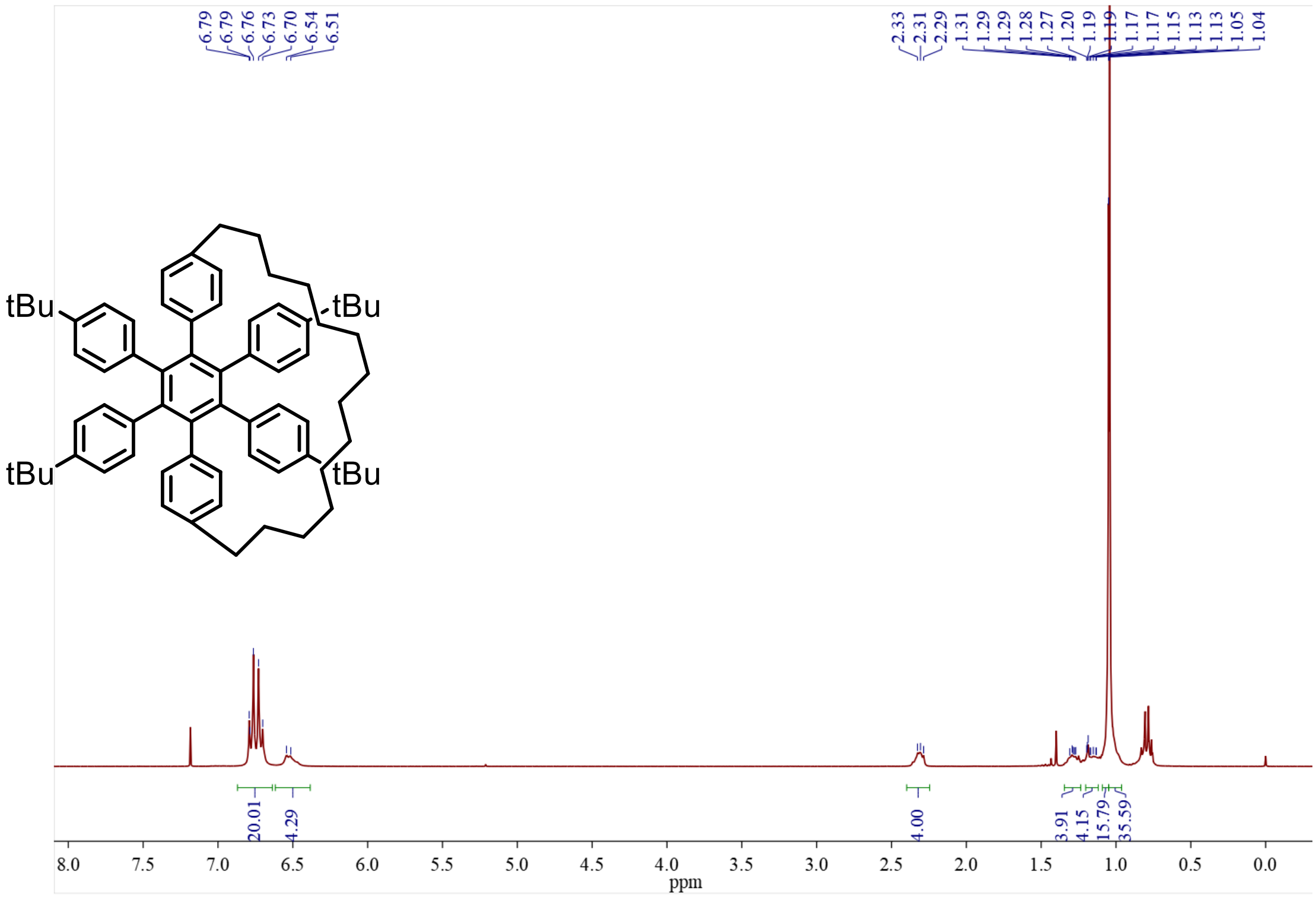

Supplementary Figure 71. ^{1}H NMR spectrum (300 MHz) of **3c** in $CDCl_3$ at room temperature.

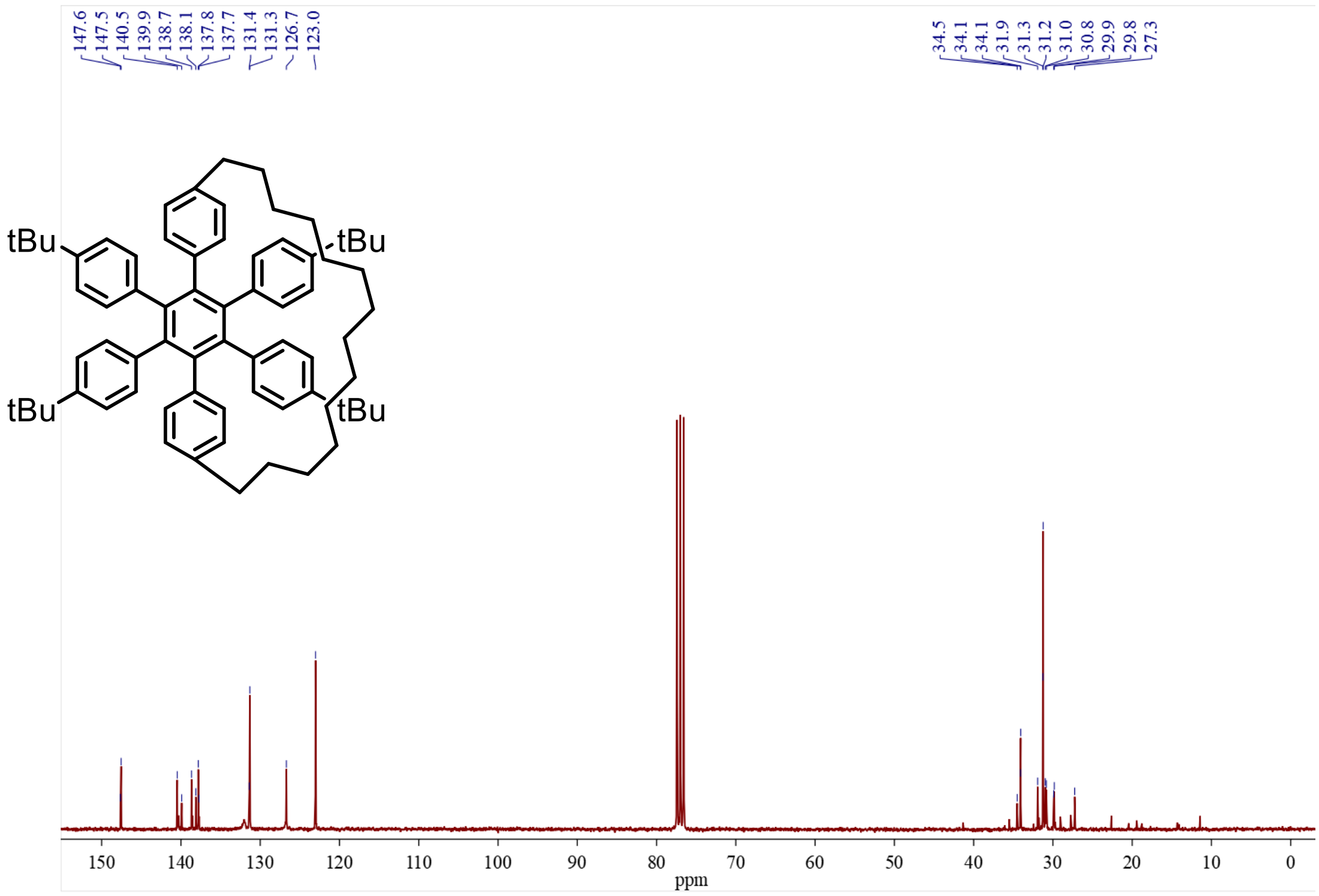

Supplementary Figure 72. ^{13}C NMR spectrum (75 MHz) of **3c** in $CDCl_3$ at room temperature.

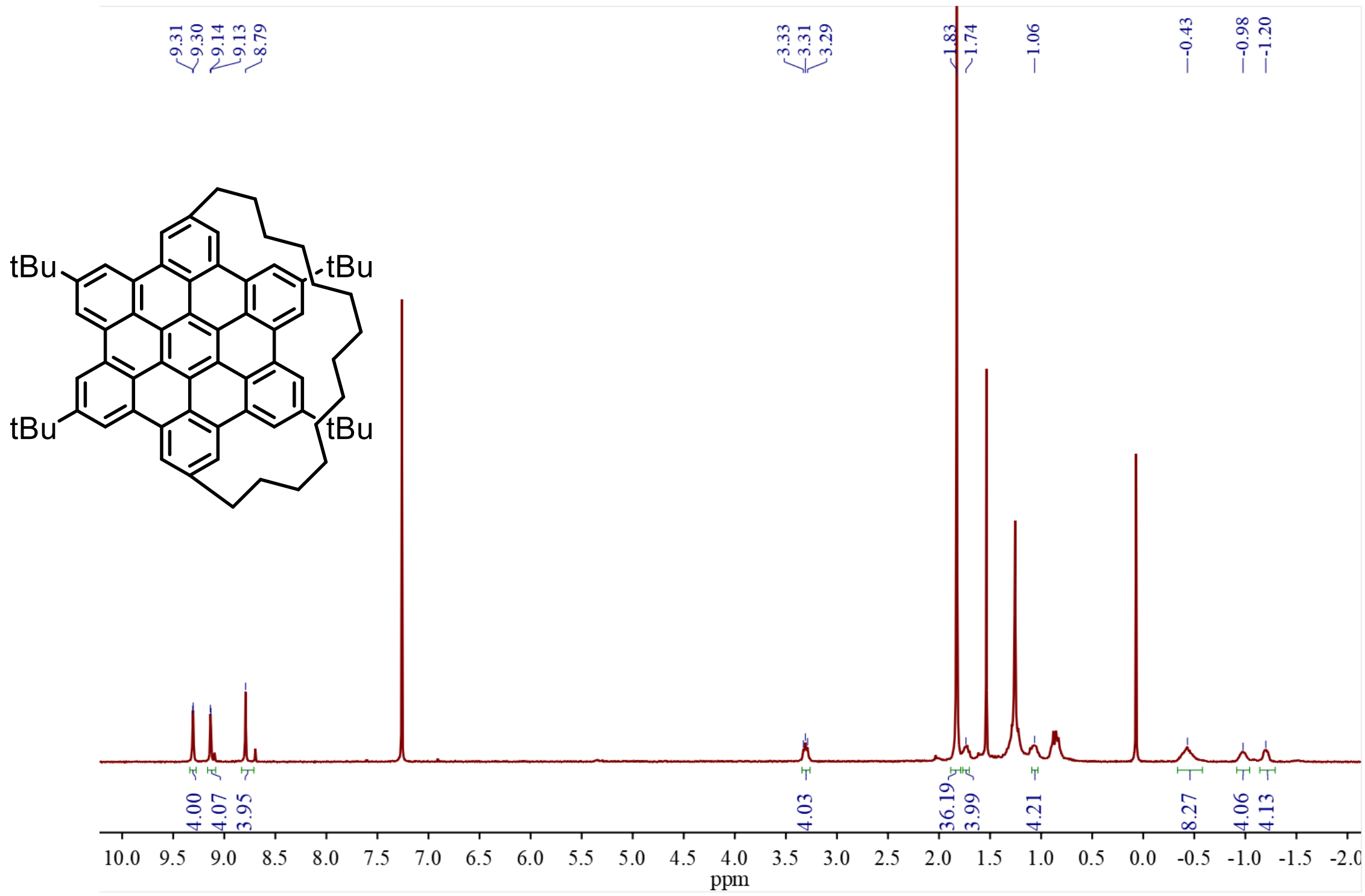

Supplementary Figure 73. ^{1}H NMR spectrum (300 MHz) of **2c** in $CDCl_3$ at room temperature.

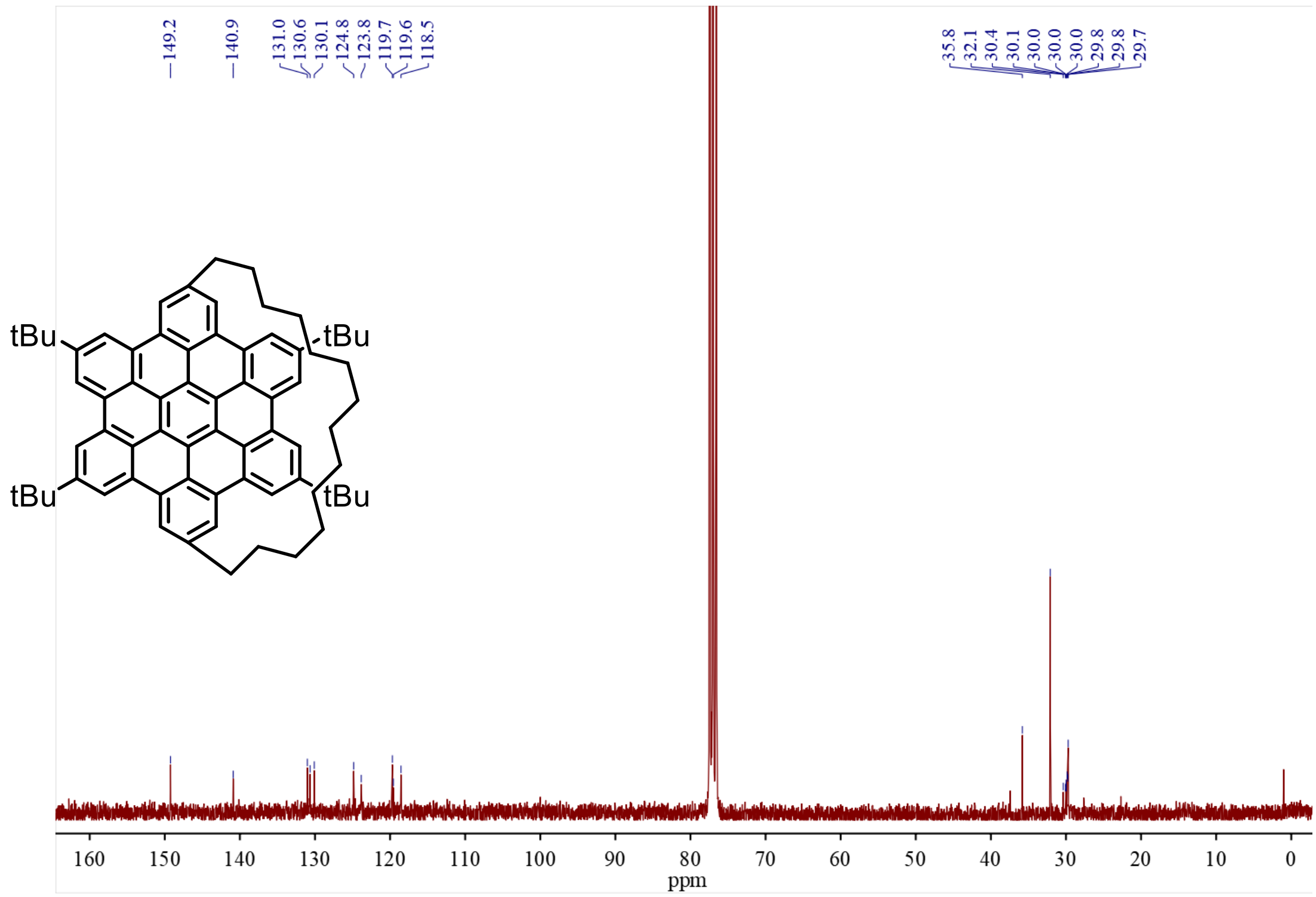

Supplementary Figure 74. ^{13}C NMR spectrum (75 MHz) of **2c** in $CDCl_3$ at room temperature.

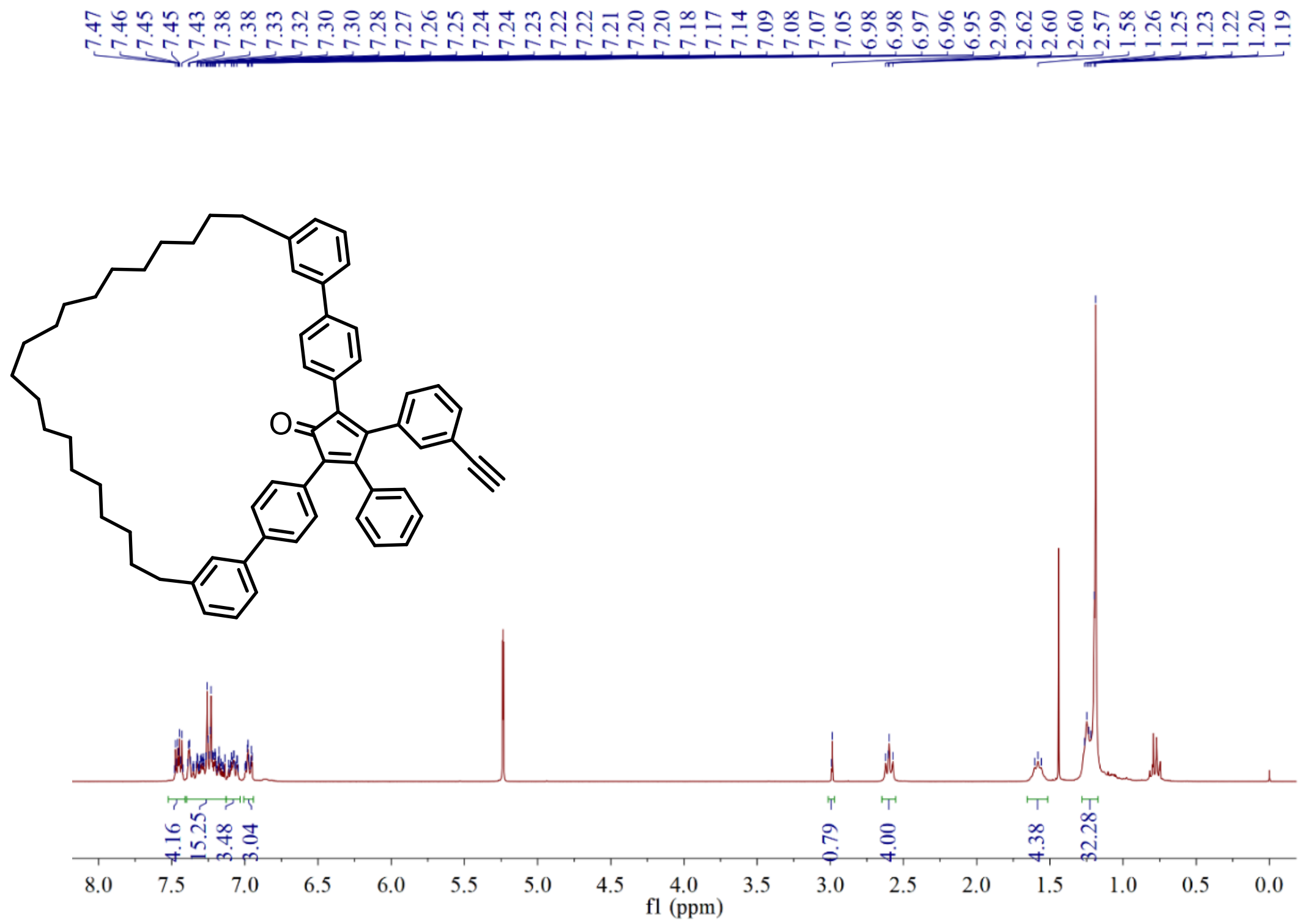

Supplementary Figure 75. ^{1}H NMR spectrum (300 MHz) of **5a** in CD_2Cl_2 at room temperature.

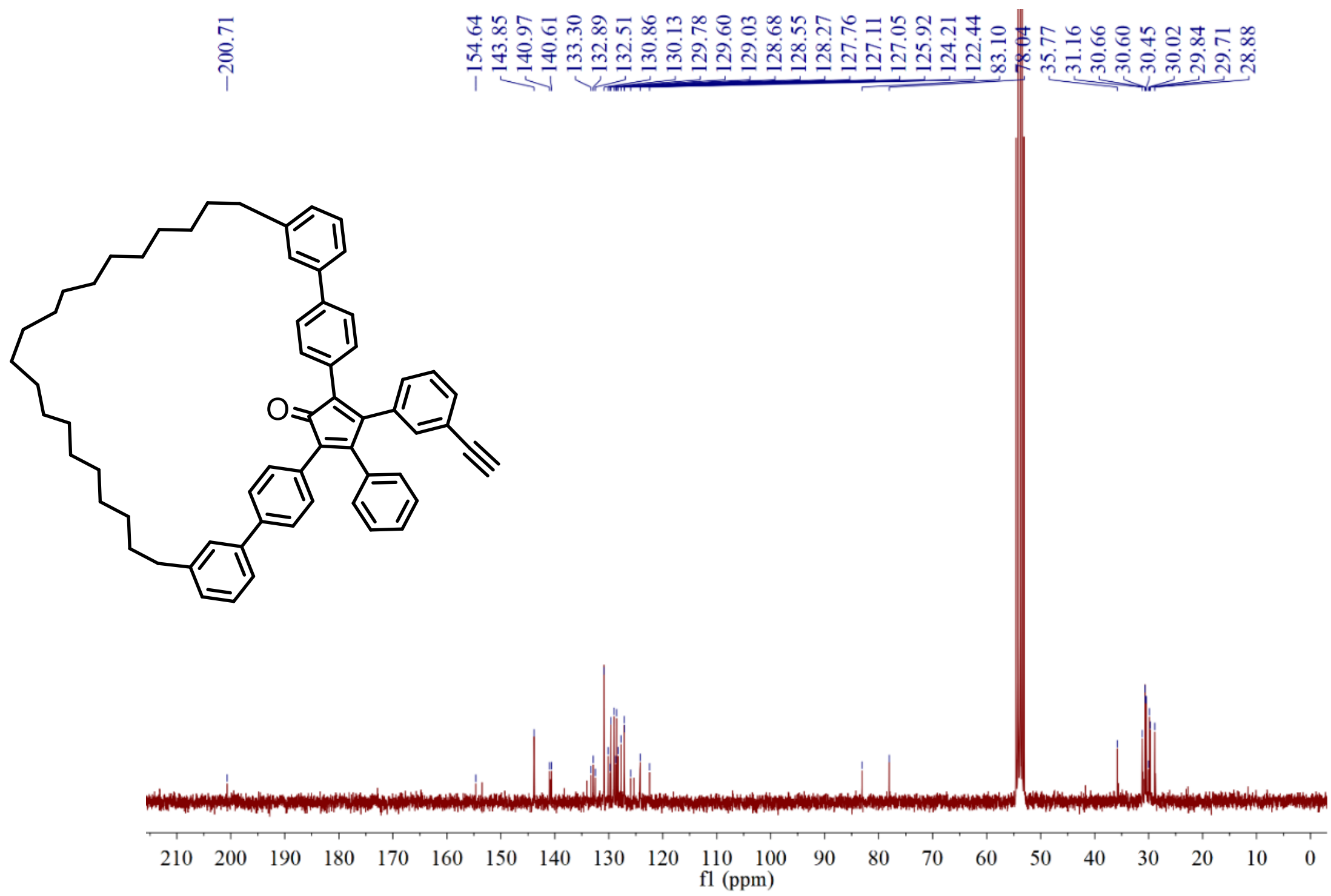

Supplementary Figure 76. ^{13}C NMR spectrum (75 MHz) of **5a** in CD_2Cl_2 at room temperature.

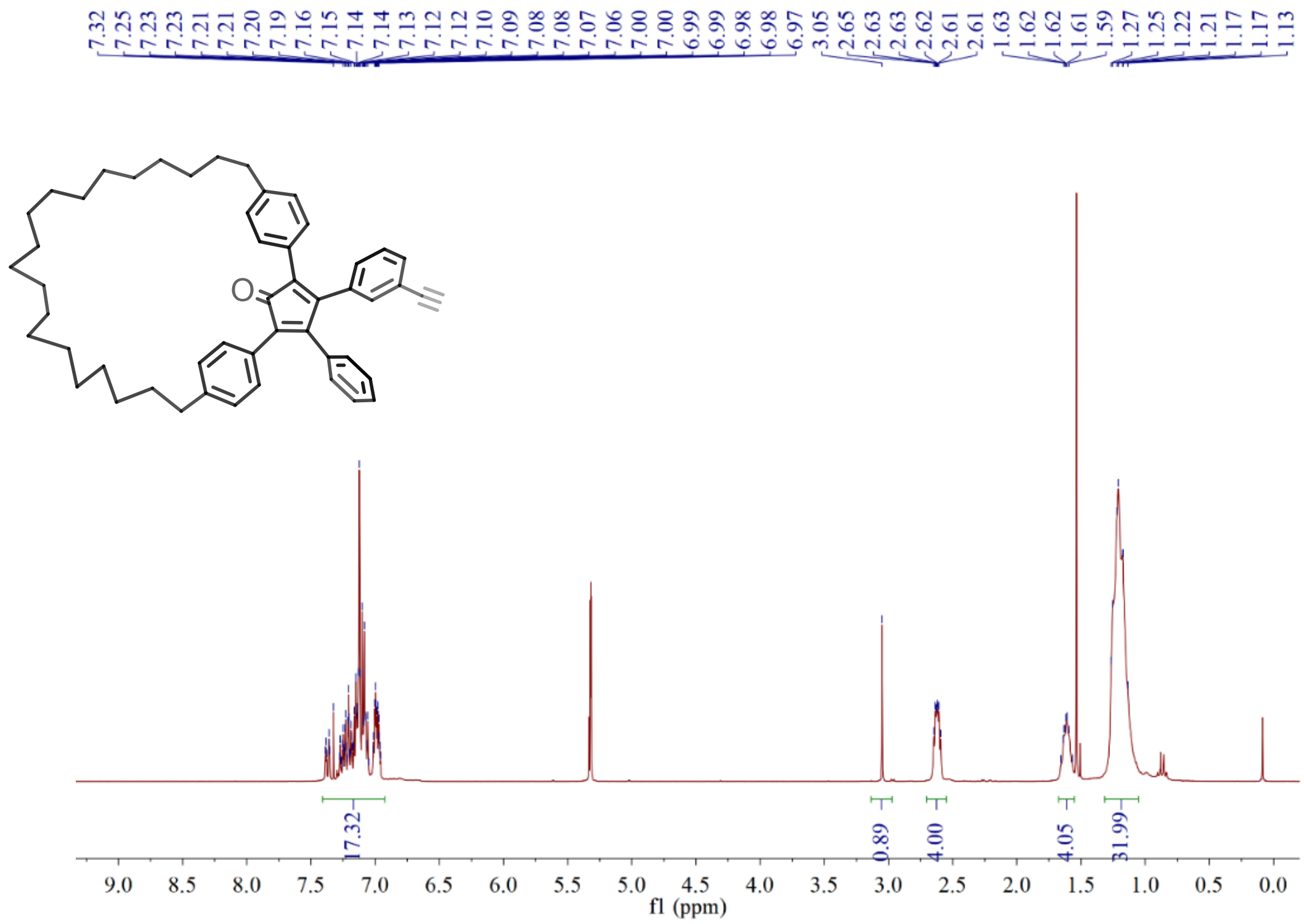

Supplementary Figure 77. ^{1}H NMR spectrum (300 MHz) of **5b** in CD_2Cl_2 at room temperature.

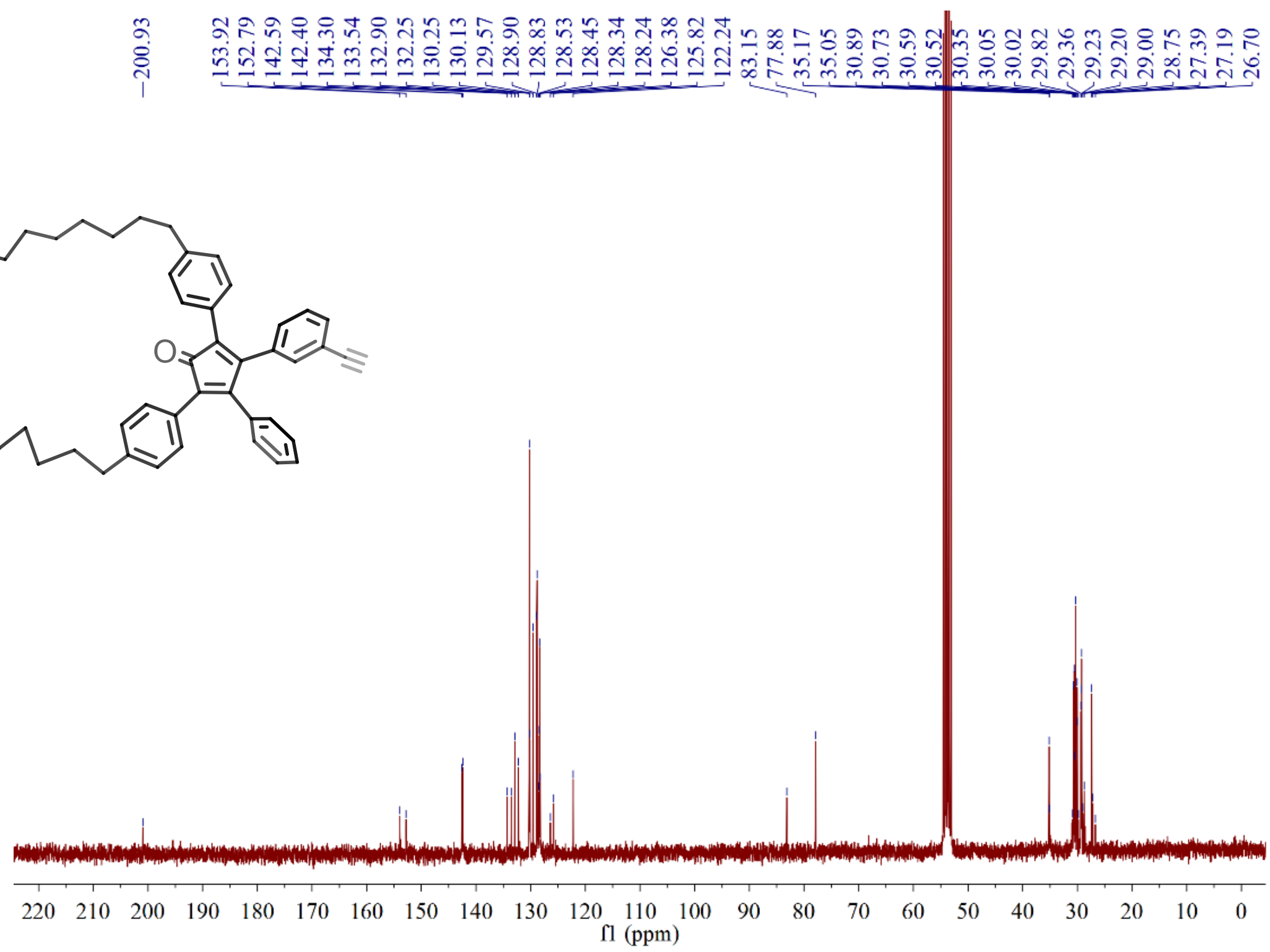

Supplementary Figure 78. ^{13}C NMR spectrum (75 MHz) of **5b** in CD_2Cl_2 at room temperature.

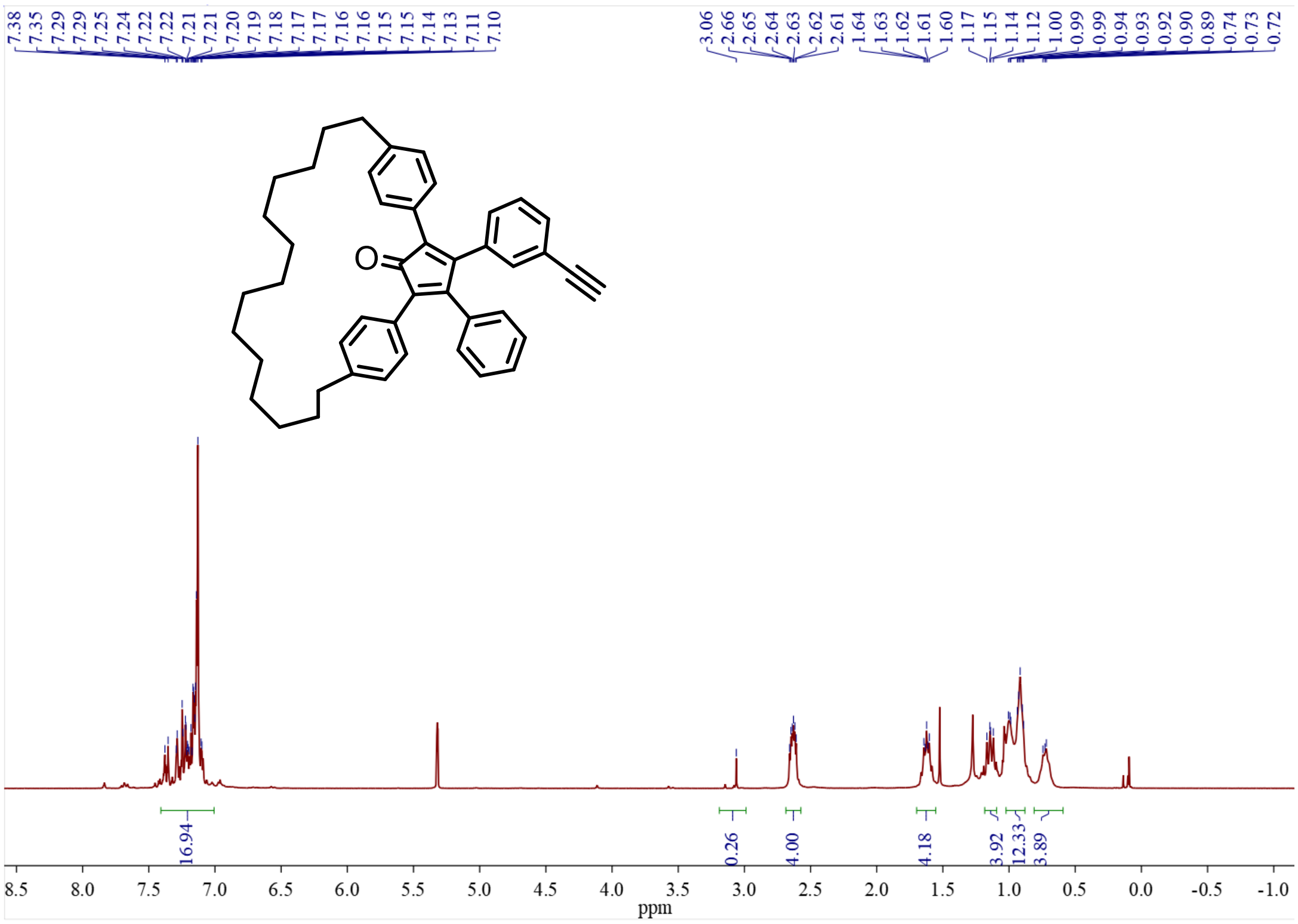

Supplementary Figure 79. ^{1}H NMR spectrum (300 MHz) of **5c** in CD_2Cl_2 at room temperature.

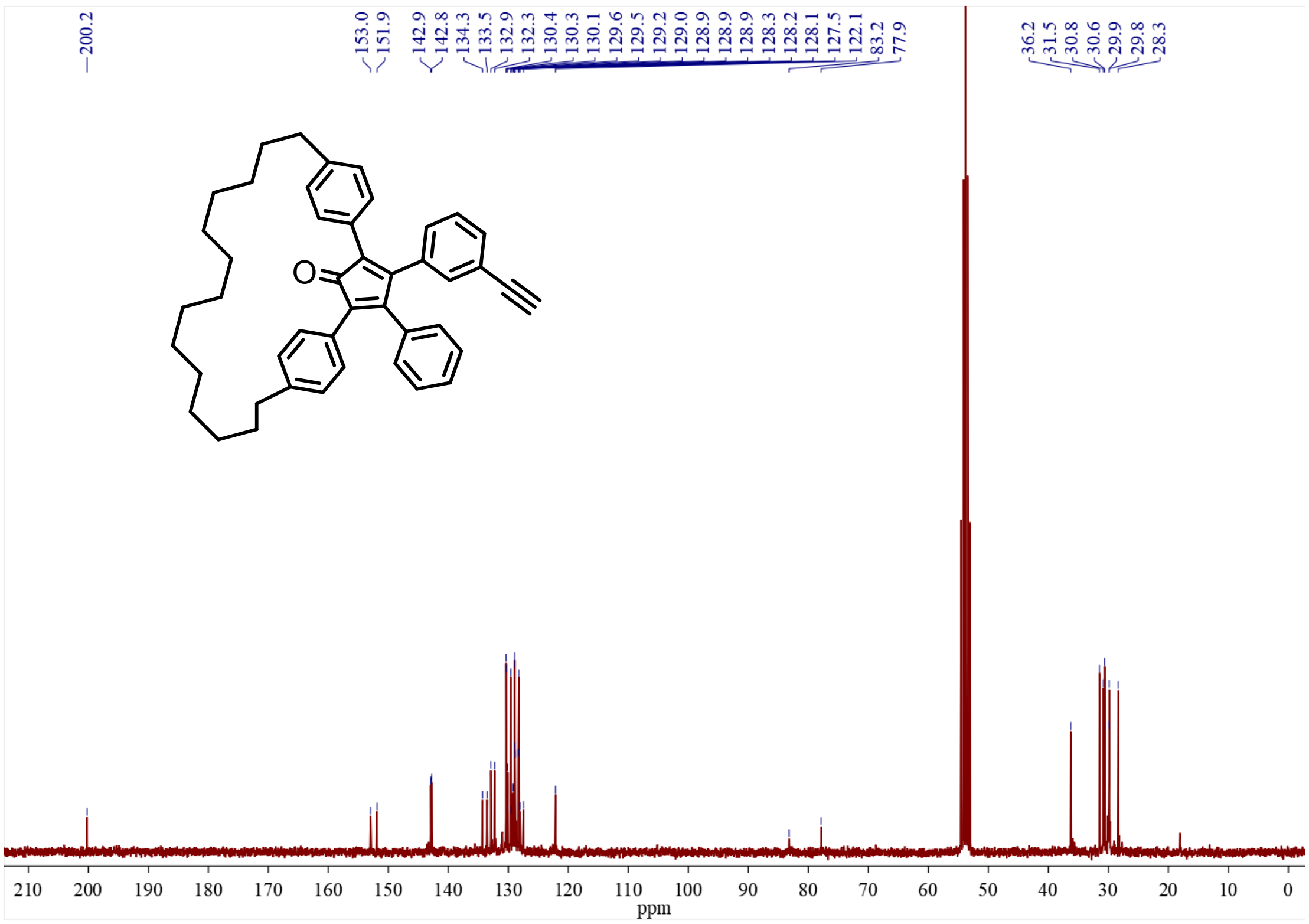

Supplementary Figure 80. ^{13}C NMR spectrum (75 MHz) of **5c** in CD_2Cl_2 at room temperature.

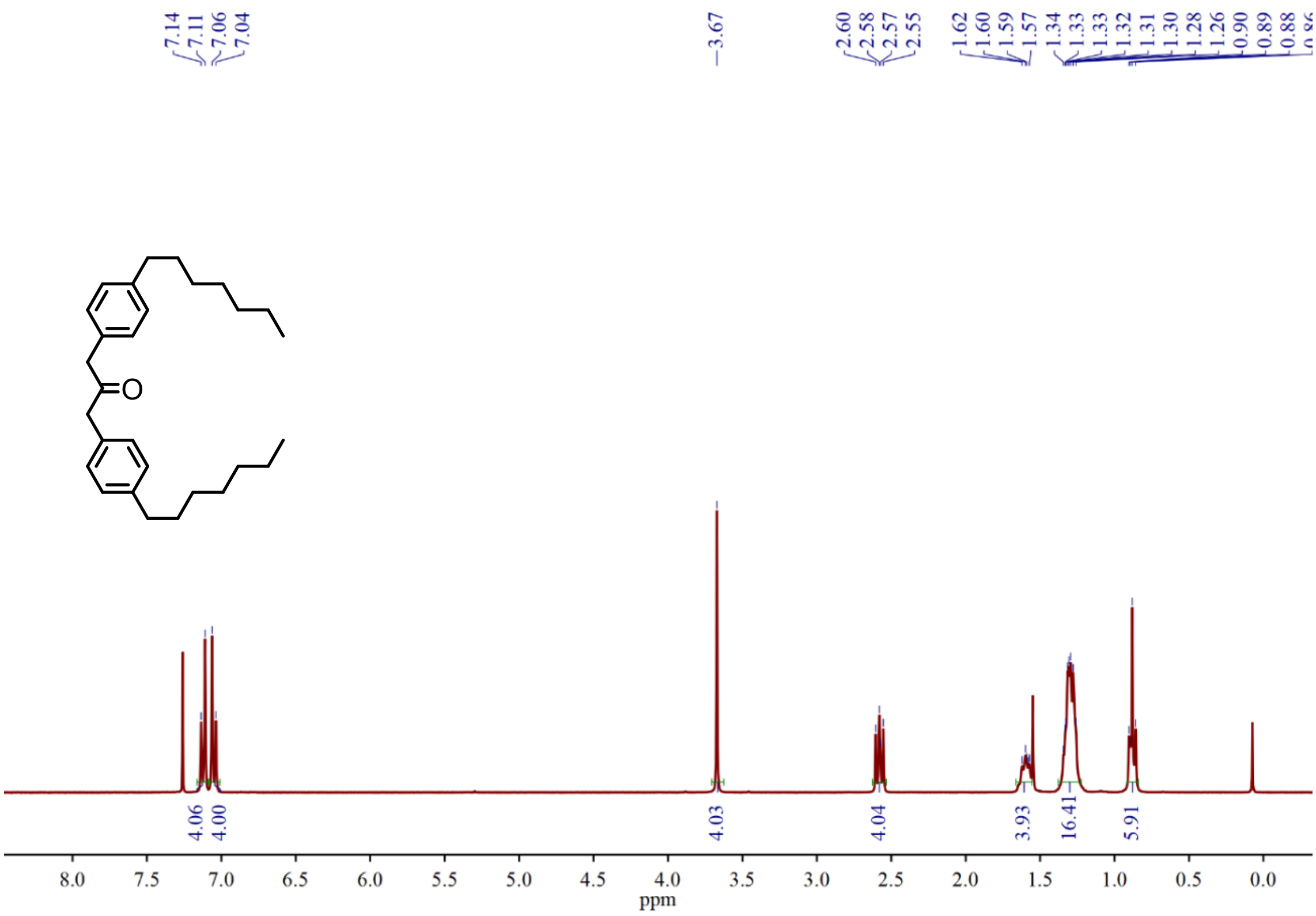

Supplementary Figure 81. ^{1}H NMR spectrum (300 MHz) of **9d** in CD_2Cl_2 at room temperature.

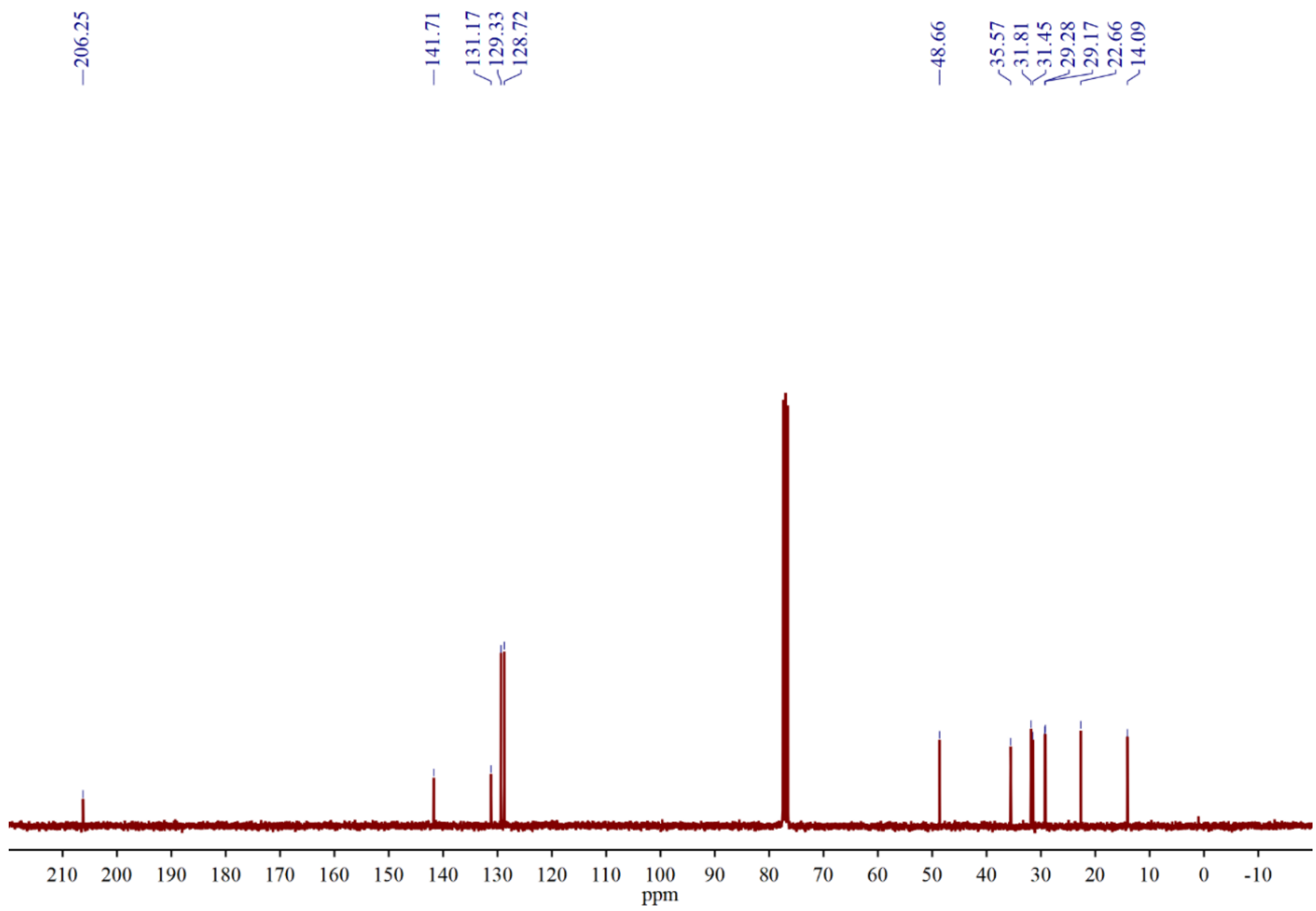

Supplementary Figure 82. ^{13}C NMR spectrum (75 MHz) of **9d** in CD_2Cl_2 at room temperature.

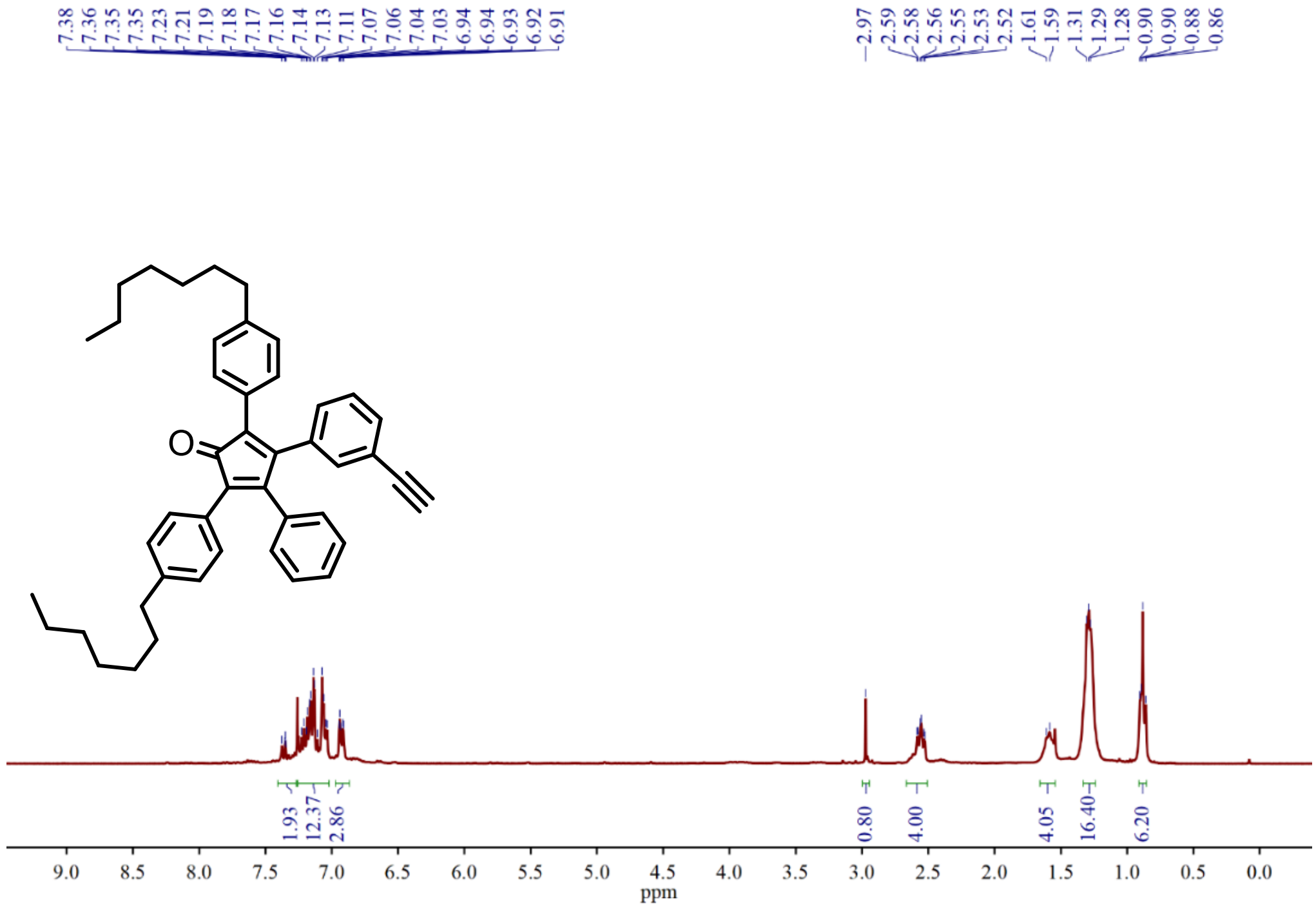

Supplementary Figure 83. ^{1}H NMR spectrum (300 MHz) of **5d** in CD_2Cl_2 at room temperature.

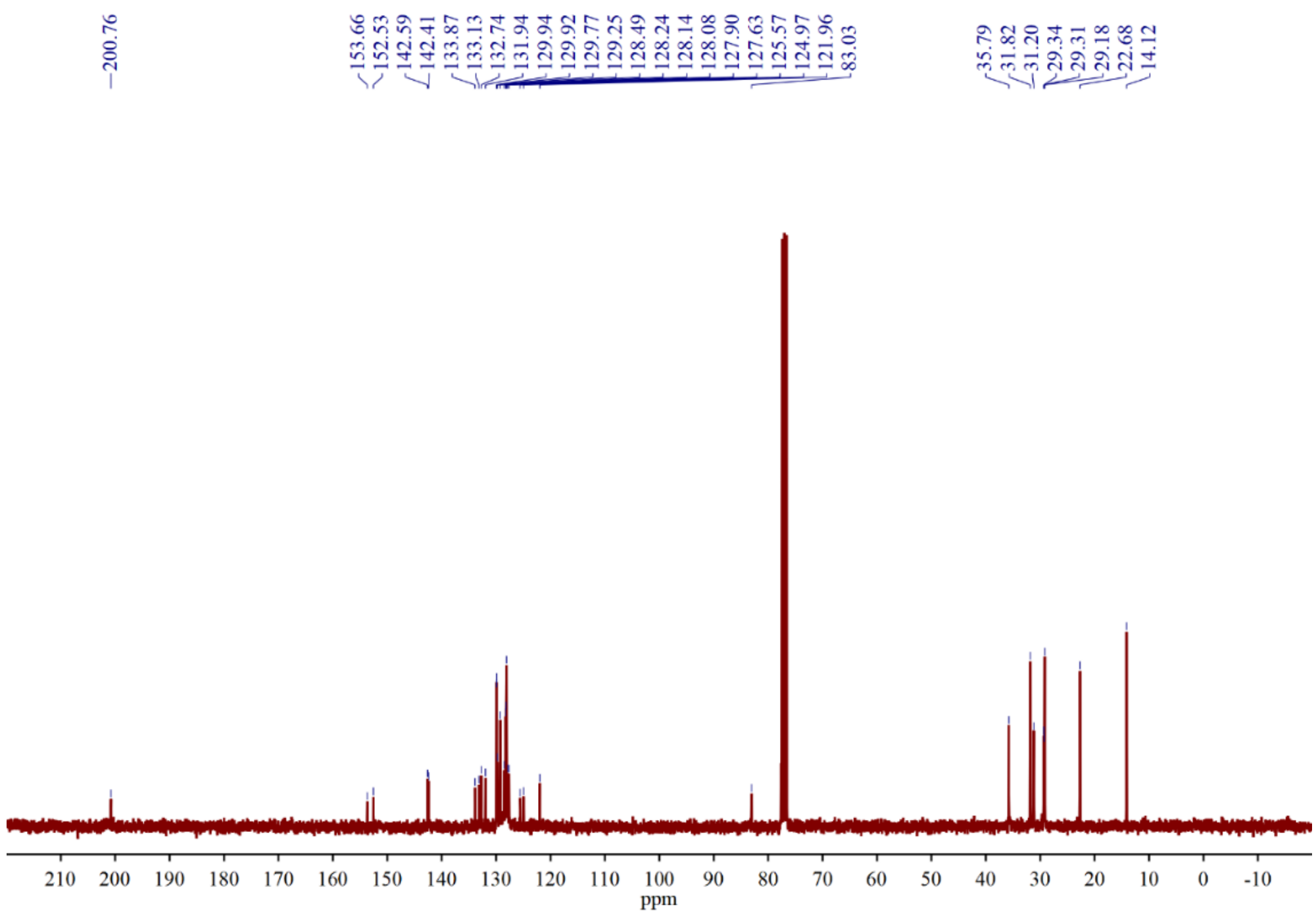

Supplementary Figure 84. ^{13}C NMR spectrum (75 MHz) of **5d** in CD_2Cl_2 at room temperature.

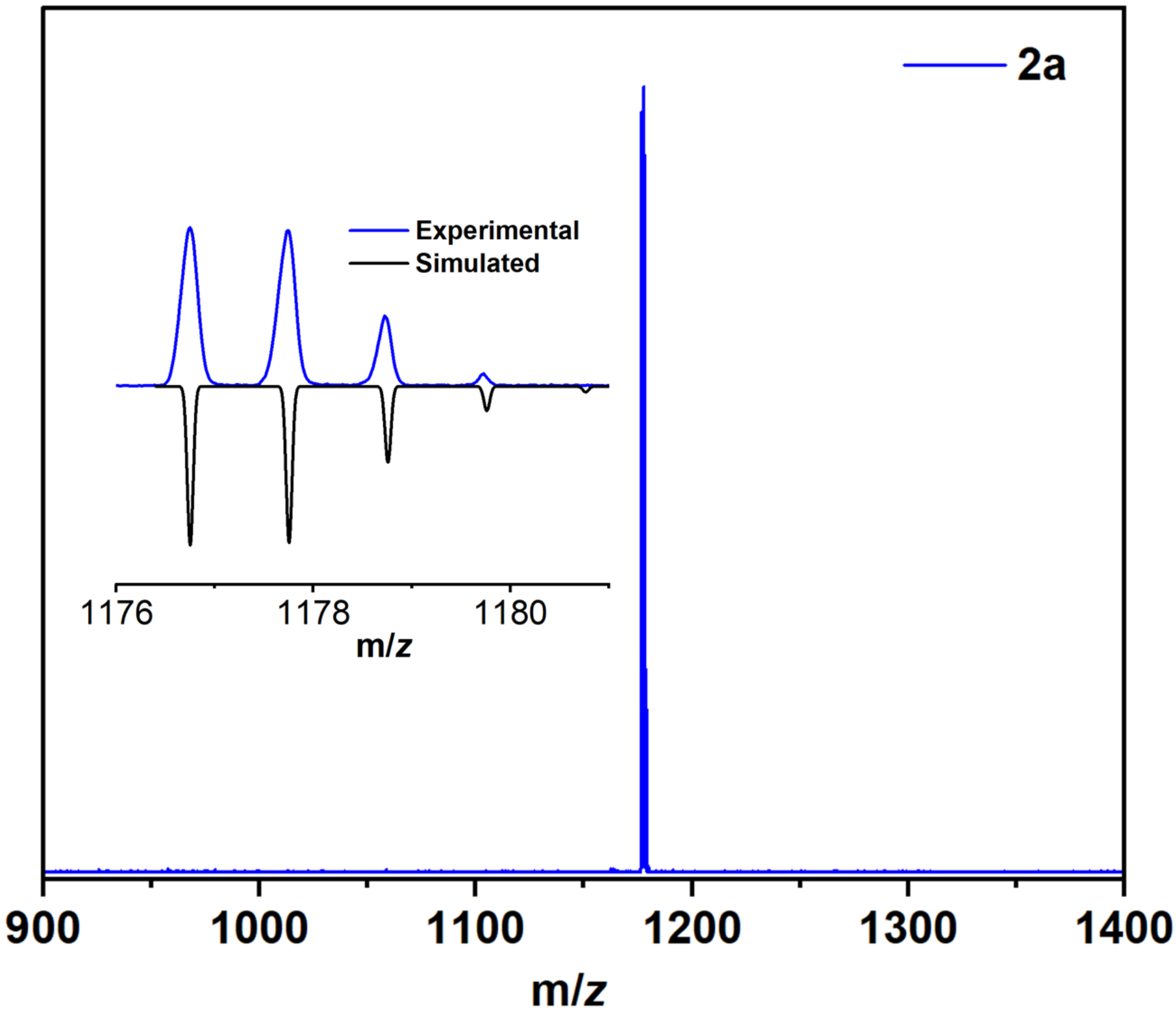

Supplementary Figure 85. HR-MALDI-TOF of compound **2a**.

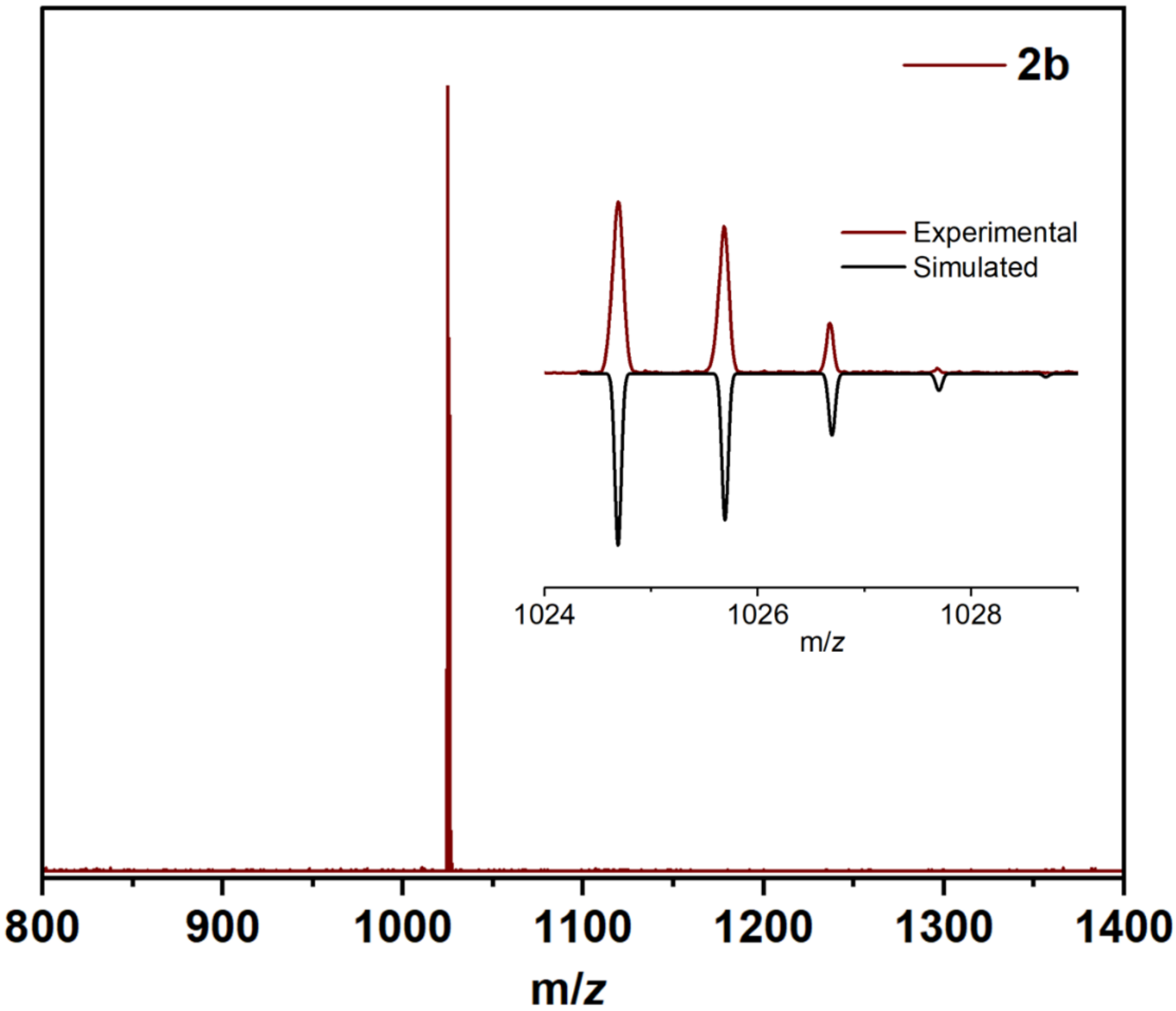

Supplementary Figure 86. HR-MALDI-TOF of compound **2b**.

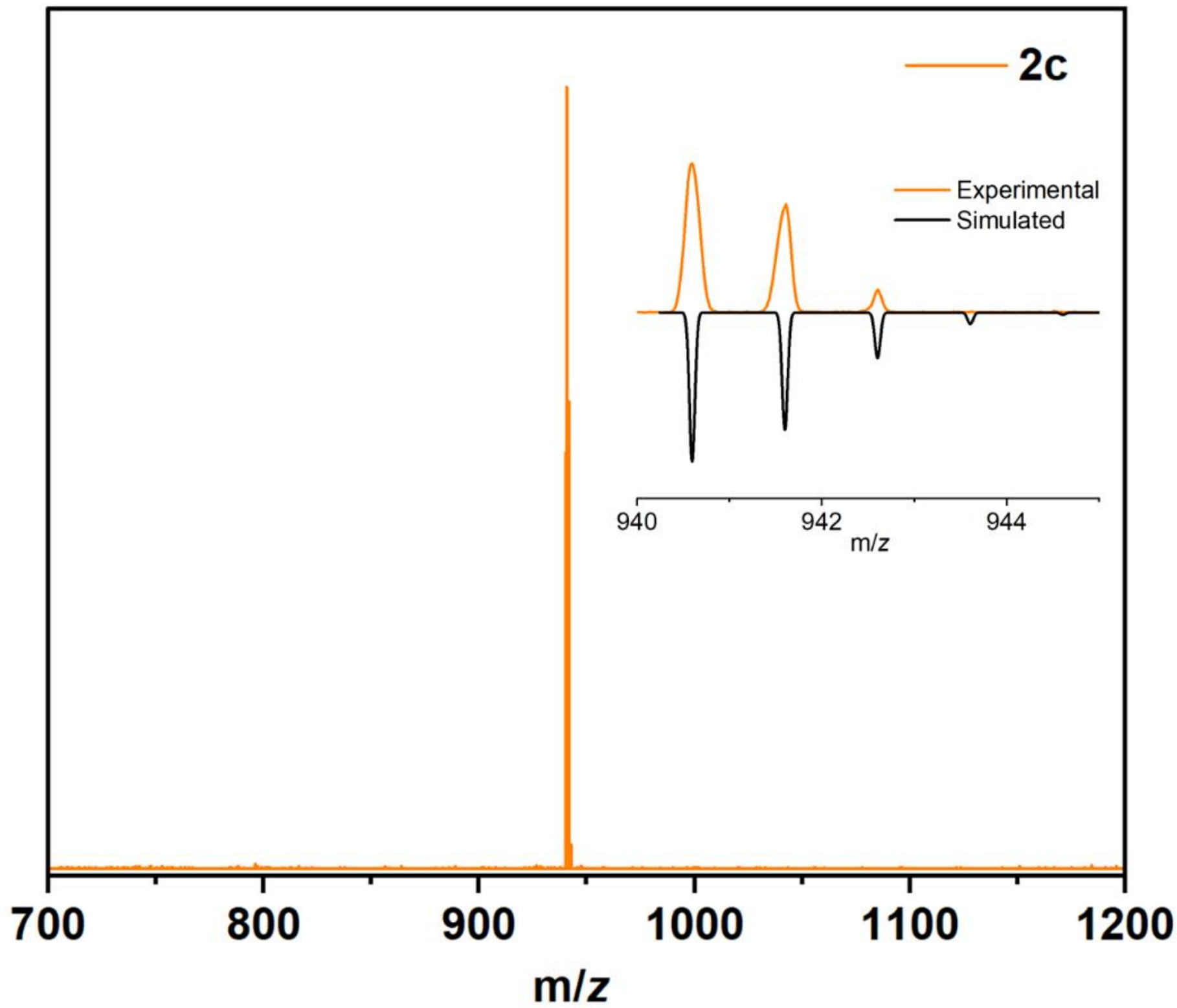

Supplementary Figure 87. HR-MALDI-TOF of compound **2c**.

11. Supplementary References

1. Hayashi, S. & Hayamizu, K. Chemical shift standards in high-resolution solid-state NMR (1) 13C, 29Si, and 1H nuclei. *Bull. Chem. Soc. Jpn.* **64**, 685-687 (1991).
2. Saalwächter, K., Lange, F., Matyjaszewski, K., Huang, C.-F. & Graf, R. BaBa-xy16: Robust and broadband homonuclear DQ recoupling for applications in rigid and soft solids up to the highest MAS frequencies. *Journal of Magnetic Resonance* **212**, 204-215 (2011).
3. Wong, W. W. H. *et al.* Solution Processable Fluorenyl Hexa-peri-hexabenzocoronenes in Organic Field-Effect Transistors and Solar Cells. *Adv. Funct. Mater.* **20**, 927-938 (2010).
4. Wong, W. W. H. *et al.* Self-Assembling Thiophene Dendrimers with a Hexa-peri-hexabenzocoronene Core−Synthesis, Characterization and Performance in Bulk Heterojunction Solar Cells. *Chem. Mater.* **22**, 457-466 (2009).
5. Narita, A. *et al.* Bottom-up synthesis of liquid-phase-processable graphene nanoribbons with near-infrared absorption. *Acs Nano* **8**, 11622-11630 (2014).
6. Gaussian 09, R. D., M. J. Frisch, G. W. Trucks, H. B. Schlegel, G. E. Scuseria, M. A. Robb, J. R. Cheeseman, G. Scalmani, V. Barone, B. Mennucci, G. A. Petersson, H. Nakatsuji, M. Caricato, X. Li, H. P. Hratchian, A. F. Izmaylov, J. Bloino, G. Zheng, J. L. Sonnenberg, M. Hada, M. Ehara, K. Toyota, R. Fukuda, J. Hasegawa, M. Ishida, T. Nakajima, Y. Honda, O. Kitao, H. Nakai, T. Vreven, J. A. Montgomery, Jr., J. E. Peralta, F. Ogliaro, M. Bearpark, J. J. Heyd, E. Brothers, K. N. Kudin, V. N. Staroverov, R. Kobayashi, J. Normand, K. Raghavachari, A. Rendell, J. C. Burant, S. S. Iyengar, J. Tomasi, M. Cossi, N. Rega, J. M. Millam, M. Klene, J. E. Knox, J. B. Cross, V. Bakken, C. Adamo, J. Jaramillo, R. Gomperts, R. E. Stratmann, O. Yazyev, A. J. Austin, R. Cammi, C. Pomelli, J. W. Ochterski, R. L. Martin, K. Morokuma, V. G. Zakrzewski, G. A. Voth, P. Salvador, J. J. Dannenberg, S. Dapprich, A. D. Daniels, Ö. Farkas, J. B. Foresman, J. V. Ortiz, J. Cioslowski, and D. J. Fox, Gaussian, Inc., Wallingford CT, 2009.
7. Grimme, S., Ehrlich, S. & Goerigk, L. Effect of the damping function in dispersion corrected density functional theory. *J. Comput. Chem.* **32**, 1456-1465 (2011).
8. Lu, T. & Chen, F. Multiwfn: A multifunctional wavefunction analyzer. *J. Comput. Chem.* **33**, 580-592 (2012).

9. Lu, T. & Chen, Q. Independent gradient model based on Hirshfeld partition: A new method for visual study of interactions in chemical systems. *J. Comput. Chem.* **43**, 539-555 (2022).
10. Humphrey, W., Dalke, A. & Schulten, K. VMD: visual molecular dynamics. *Journal of molecular graphics* **14**, 33-38 (1996).
11. Grimme, S., Ehrlich, S. & Goerigk, L. Effect of the damping function in dispersion corrected density functional theory. *J. Comput. Chem.* **32**, 1456-1465 (2011).
12. Narita, A. *et al.* Synthesis of structurally well-defined and liquid-phase-processable graphene nanoribbons. *Nat. Chem.* **6**, 126-132 (2014).
13. Götz, A. *et al.* Band structure modulation by methoxy-functionalization of graphene nanoribbons. *J. Mater. Chem. C* **10**, 4173-4181 (2022).
14. Niu, W. *et al.* Exceptionally clean single-electron transistors from solutions of molecular graphene nanoribbons. *Nat. Mater.* **22**, 180-185 (2023).
